# Stereodivergent Synthesis of 6,12-Guaianolide C1 Epimers via a Rationally Designed Oxy-Cope/Ene Reaction Cascade

**DOI:** 10.1021/acs.orglett.4c03504

**Published:** 2024-11-04

**Authors:** Kalliopi Mazaraki, Christos Zangelidis, Antonis Kelesidis, Alexandros L. Zografos

**Affiliations:** Laboratory of Organic Chemistry, Department of Chemistry, Aristotle University of Thessaloniki, Thessaloniki 54124, Greece

## Abstract

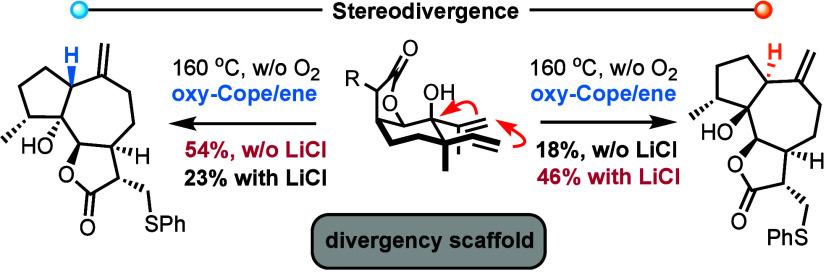

Nature synthesizes epimeric C1 guaianolide congeners, key components of major natural product classes, through a single structurally flexible macrocyclic germacranolide core. Our rationally designed elemanolide-type scaffold (**5**) now mimics this natural process, enabling the stereodivergent synthesis of both C1 epimers of 6,12-guaianolide lactone motifs. An oxy-Cope/ene cascade acts as the key step of this process, generating two distinct conformers of an intermediate germacranolide, each leading to a specific C1 epimer. Highly stereoselective redox manipulations follow, culminating in the efficient syntheses of diverse osmitopsin-type guaianolides.

The total synthesis of guaianolide sesquiterpenoid lactones (SQLs) commonly relies on semisynthesis. This process utilizes a set of well-established transformations applied to a chiral pool of starting materials, to address the inherent structural complexity of guaianolide sesquiterpenoid lactones.^1^ While this strategy has undoubtedly been successful, the fixed stereochemistry of the limited number of readily available starting materials restricts their potential for diversification. Instead, designing and building appropriate divergency scaffolds that mimic biosynthetic routes would theoretically enable the synthesis of a broader spectrum of natural-product-like complexity, eliminating the necessity for a targeted retrosynthetic analysis tailored to specific products.^2^ Following this rationale, our group has developed divergency scaffolds to address furosesquiterpenoid, Apiaceae-type sesquiterpenoid, and recently 8,12-sesquiterpenoid lactone complexity.^3^ The highly abundant regioisomeric 6,12-sesquiterpenoid lactones represent the most biologically active congeners in the family of SQLs (Scheme 1).^4^ Keen to integrate them into our divergent toolkit, we report our initial endeavors to explore their synthesis. In this letter, we report a highly adaptable synthetic strategy for the total synthesis of guaianolide congeners of both α- and β-H at the C1 position of the carbocyclic core, utilizing easily scaled-up elemanolide **5** as a divergency scaffold (Scheme 1).

**Scheme 1:**
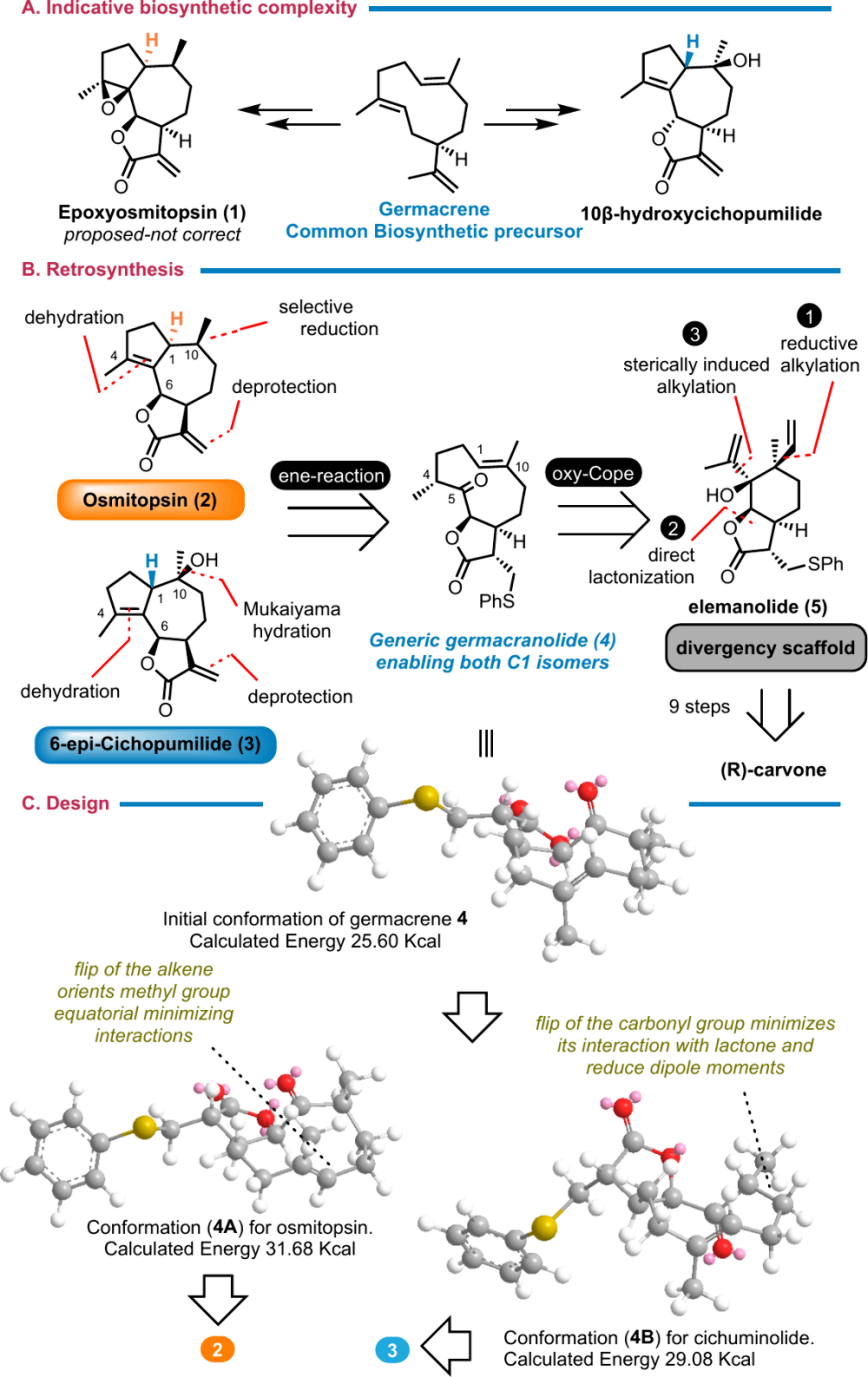
Divergent Indicative Retrosynthetic Analysis for Osmitopsin (**2**) and 6-*epi*-Cichopumilide (**3**): Rational Design of Elemanolide **5** To Achieve C1 Epimer Divergency Energy calculations were performed with Chem3D 18.0 software.

Osmitopsin (**2**) and 4,5-epoxy-osmitopsin (**1**), which were initially isolated from the aerial parts of *Osmitopsis asteriscoides* in 1975, represented the first known guaianolides bearing a *cis*-6,12-α-methylene-γ-butenolide moiety.^5^ Enantioselective total syntheses of compounds **2** and **1** by Metz’s group revealed significant differences in the spectral data of the latter compared to the originally reported data.^6^ This disparity has left its structure and biological profile unexplored, while the total synthesis and biological investigation of the structurally related racemic C10 epimer of 4,5-epoxy-osmitopsin showed significant activity against schistosomal cercariae, a cause of bilharziosis.^7^ To facilitate future investigations into the structure–activity relationship of osmitopsin-type compounds, the synthesis of diverse congeners at C1 and C10 is being targeted.^8^

Despite our extensive experience in the total synthesis of 8,12-congeners,^3^ 6,12-sesquiterpenoid lactones present several synthetic challenges. The indicative retrosyntheses of osmitopsin (**2**) and the related epimer at C1, 6-*epi*-cichopumilide (**3**), for example, require the development of the appropriate stereoselective redox transformations for their late-stage functionalization, but most importantly, it demands diastereoselective access to both epimers of C1 in the guaianolide carbocycle. This latter task has been surprisingly unprecedented when considering a common synthetic plan to access them, in contrast to their biosynthetic origin involving the stereoselective cyclization of a common germacranolide core.

Expanding on our previous efforts,^3^ the described diastereodivergency at C1 of guaianolides might be addressed by an oxy-Cope/ene reaction cascade of an appropriate elemane precursor **5** (Scheme 1B). This task becomes feasible if the 10-member germacranolide macrocycle **4**, formed by the initial oxy-Cope reaction of compound **5**, allows for the rotation of both the carbonyl group and the alkene bond in the subsequent ene reaction (conformations **4a** and **4b**; Scheme 1C). These rotations would permit the delivery of both epimeric forms of the guaianolide carbocycle. An additional advantage might arise from the projected equilibration between the potential germacranolide and guaianolide cores, further enabling the synthesis of one epimer over the other under appropriate conditions.^9^

Molecular modeling calculations on these conformationally active intermediates (**4a** and **4b**) reveal similar energies for both carbonyl and double-bond rotations, indicating the potential accessibility to both C1 α and β isomers. Elemanolide **5** can be further disconnected retrosynthetically to *R*-carvone using its reductive alkylation, a direct lactonization sequence, and a sterically induced alkylation as key steps.

The synthesis commenced with the known transformation of *R*-carvone to compound **6** (Scheme 2A). The four-step sequence involves the reductive alkylation of *R*-carvone with l-selectride and acetaldehyde,^3d,10^ the subsequent mesylation and dehydration of the obtained secondary alcohol, and finally the allylic chlorination with Ca(OCl)_2_ to provide compound **6** in 56% overall yield. The early introduction of the α-methylene-γ-butenolide core is envisioned to facilitate the stereoselective introduction of the remaining unsaturated alkyl chain toward the synthesis of the divergency scaffold **5**. Thus, obtained compound **6** was readily transformed to acrylic acid **7** by its treatment with sodium bicarbonate and sodium iodide in dimethyl sulfoxide (DMSO),^11^ followed by Lindgren’s protocol, before attempting its direct lactonization.^12^ The formation of the 6,12-lactone moiety for the synthesis of compound **8** posed a challenge due to the high reactivity of pendant alkene toward electrophiles. This issue was addressed through a radical bromination process, using *N*-bromosuccinimide (NBS) in the presence of ultraviolet (UV) light, yielding compound **8** in 53% yield, after treatment with NaHCO_3_. With α-methylene-γ-butenolide **8** available in gram-scale quantities, the next step involved protecting its notoriously nucleophile-sensitive site using thiophenol and 4-dimethylaminopyridine (DMAP), resulting in compound **9**. Attempts to alkylate compound **9** revealed its sensitivity to nucleophilic sources. Isopropenyl magnesium bromide led to a mixture of unidentified products, while isopropenyl lithium returned a low yield of the desired product **5** with non-selective diastereoselection, along with products from the addition to carbonyl of the lactone moiety (see the Supporting Information). To overcome these, isopropenyl cerium chloride was utilized providing compound **5** as a mixture of diastereoisomers [diastereomeric ratio (dr) = 4:1] favoring the anti-orientation between the alkenyl chains. Further optimization with the aid of LiCl as an additive yielded compound **5** in 66% yield in an excellent diastereocontrol (dr = 15:1) (Scheme 2A).

**Scheme 2:**
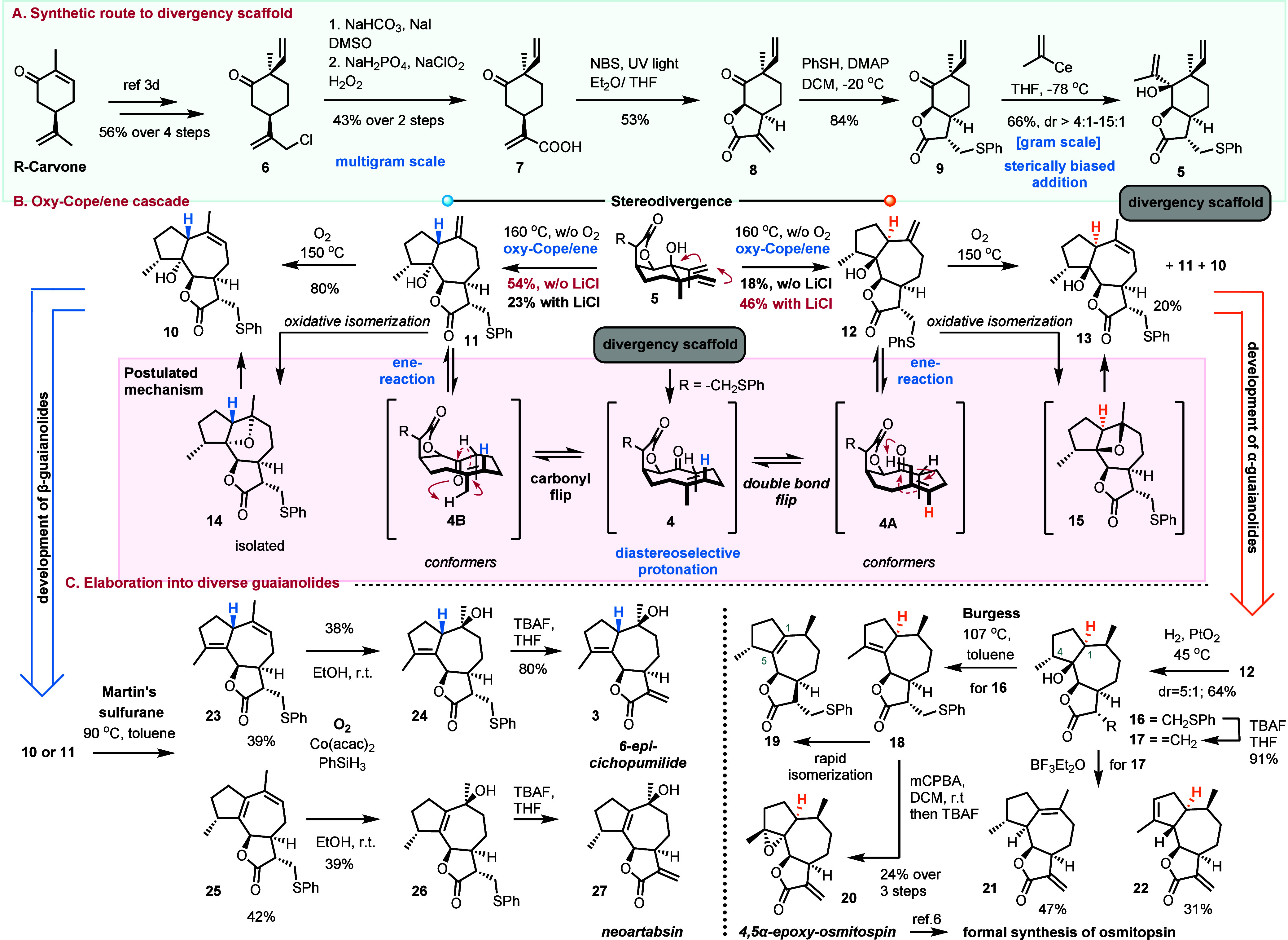
Synthesis of the Divergency Scaffold **5** and Its Elaboration to Diverse Guaianolide Scaffolds

In confirmation of our design and predictions, the thermal oxy-Cope reaction of compound **5** at 160 °C in deoxygenated toluene produced products **11** and **12** in an almost 3:1 ratio, with an overall yield of 72%, and, notably, without isolating the germacranolide **4** intermediate (Scheme 2B). Intriguingly, when the thermal oxy-Cope/ene reaction was conducted at 160 °C in the presence of dioxygen, only isomerized guaianolide **10** was obtained in 41% yield, suggesting an oxidative decomposition and/or transformation of compound **12** to compound **11** before the isomerization step (Scheme 2B). To substantiate our hypothesis, heating a pure sample of compound **11** in toluene in the presence of dioxygen at 150 °C led to the isolation of stable oxetane **14** and isomerized product **10**, indicating a thermal opening of oxetane to compound **10**. Indeed, heating oxetane **14** at 150 °C led exclusively to the formation of product **10** in 80% yield. A similar oxidative transformation was also evidenced when compound **12** was heated under dioxygen at 150 °C, providing isomerized compound **13** in 20% yield without isolating respective oxetane **15**, along with compounds **11** and **10**. This interesting unprecedented oxidative isomerization of alkenes will be further commented on below (Scheme 3). Heating the β-H isomer **11** under deoxygenated conditions at 150 °C led to a 3:1 mixture of compounds **11**/**12**, demonstrating the reversibility of the ene process. With the connection of every piece of information in attempting to postulate a mechanism, a non-reversible oxy-Cope/reversible ene reaction sequence, toward the thermodynamic production preference for compound **11**, bearing the β-H stereoisomer in the guaianolide junction, when dioxygen is absent, appears plausible. A closer look to germacranolide conformers can shed light to this preference as part of a dipole moment decrease in the system. Oxidative isomerization of compound **11** leads to the non-reversible production of compound **10** when dioxygen is present. Focusing on germacranolide conformers **4A** and **4B**, it is expected that an appropriate metal interaction between the carbonyl and lactone moieties might allow delayed rotation of the carbonyl group, thus enriching the production of α-H guaianolide stereoisomer **12**. Indeed, when LiCl was introduced in the oxy-Cope/ene reaction of compound **5** in deoxygenated toluene at 160 °C, compound **12** was obtained as a 2:1 mixture, indicating a clear preference. Integrating this result with ene reaction reversibility allows the interconversion of isomer **11** to isomer **12** in 60% yield. To the best of our knowledge, it is the first time a synthetic plan can diverge selectively between the two epimeric forms of C1 on the guaianolide carbocycles.

**Scheme 3:**
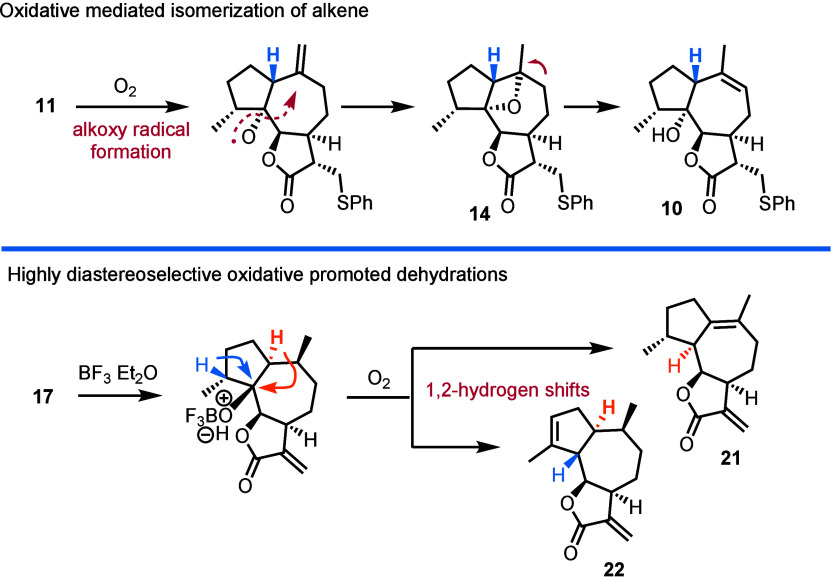
Highly Diastereoselective Oxidative Transformations Derived from Tertiary Guaianolide Hydroxyl at the 5 Position

With ample quantities of both compounds **11** and **12** at our disposal, we proceeded to complete the total synthesis of osmitopsin congeners with diversity at C1 and C10 (Scheme 1C).

Initially, the total synthesis of the osmitopsin natural product (**2**) was pursued (right side of Scheme 1C). α-H isomer **12** was reduced with hydrogen in the presence of Adams’ catalyst to deliver the desired β-methyl product **16** in good diastereocontrol (dr = 5:1). Unfortunately, despite the plethora of available methods, its dehydration to osmitopsin’s core posed a significant challenge. Screening commonly employed conditions for activating tertiary hydroxyl at C5 (COCl_2_, POCl_3_, Martin sulfurane, etc.) yielded inconsistent results, low yields, and inseparable mixtures of regioisomeric alkene products. Fortuitously, the reaction with Burgess reagent produced product **18** in moderate yield (55%); however, it proved sensitive to chromatographic purification and CDCl_3_ conditions and transformed acutely to the undesired regioisomer **19**. To address this sensitivity issue, the direct treatment of the Burgess reaction mixture with excess mCPBA resulted in the epoxidation of C4–C5 alkene and oxidation of sulfide to sulfone that was readily deprotected with tetrabutylammonium fluoride (TBAF) to provide the total synthesis of 4,5-epoxy-osmitopsin **20** in 23% over the three steps and the formal synthesis of osmitopsin.^6^ Attempts to overpass the observed sensitivity of protected osmitopsin by the initial deprotection of compound **16** with TBAF to compound **17** followed by dehydration with Burgess reagent led unexpectedly solely to the corresponding C1–C5 alkene. Additional attempts to dehydrate deprotected alcohol **17** using BF_3_·Et_2_O resulted in the stereoselective formation of alkene osmitopsin congeners **21** (C3–C4 alkene) and **22** (C1–C10 alkene). Interestingly, the latter reaction initiates only in the presence of dioxygen, pointing again toward an oxidative radical-based process (Scheme 3). The interesting behavior of 5-hydroxylated guaianolides under oxidative conditions (**11** to **10**) that was priorly witnessed in our attempts to synthesize Apiaceae congeners^3e^ advocates for an alkoxy radical formation from the tertiary hydroxyl group, in the presence of dioxygen. The latter subsequently reacts with alkene, leading to compound **14**. This result indicates the unprecedented generation of alkoxy radicals from tertiary alcohols in the presence of dioxygen without preactivation, metals, or light. Selective cleavage of oxetane **14** explains the thermodynamically contra generation of trisubstituted alkene **13** over its tetrasubstituted congeners. In the case of products **21** and **22**, the complete lack of products when dioxygen is absent and the unselective nature of the cations formed when protic acids were used indicate the formation of a radical cation. In this case, the reaction of tertiary alcohol with BF_3_·etherate is proposed to form easily oxidized boronate responsible for the highly diastereoselective dehydrations observed. Further investigations are underway to harness the full potential of these observations.

Focusing on exploring the chemical behavior of C1 epimer **11** (left side of Scheme 1C), we subjected it to a reaction with Martin’s sulfurane. This effort yielded dienes **23** and **25** in 39 and 42% yields, respectively. With the employment of Mukaiyama hydration with Co(acac)_2_ catalysis, phenylsilane and dioxygen in both alkenes resulted in the selective formation of β-hydroxy compounds **24** and **26**, which, after deprotection with TBAF, possess the correct stereochemistry for the total synthesis of 6-*epi*-10β-cichopumilide and an unnatural derivative of artabsin (named neoartabsin), with the former standing out as an unprecedented 1-*epi*-10β-hydroxy osmitopsin analogue.

In conclusion, the rationally designed synthesis of 6,12-elemanolide **5** allows the unprecedented divergent synthesis of both C1 epimers of 6,12-guaianolide through a unified synthetic plan, harnessing the versatility of a reversible oxy-Cope/ene reaction cascade. The effectiveness of this strategy is vividly demonstrated by the streamlined synthesis of C1 and C10 osmitopsin-type congeners, underscoring the elegance and efficiency of our approach. Important indications for alkoxy radical generation from tertiary alcohols in the presence of dioxygen have also been provided, highlighting for the first time the ability of tertiary alcohols to serve as alkoxy radical precursors under metal-free conditions.

## Data Availability

The data underlying this study are available in the published article and its Supporting Information.

## Supporting Information Available

Synthetic protocols, spectroscopic characterization, and ^1^H and ^13^C nuclear magnetic resonance (NMR) spectra of new compounds (PDF)
