# Supplementary material for: Stereodivergent Synthesis of 6,12-Guaianolide C1 Epimers via a Rationally Designed Oxy-Cope/Ene Reaction Cascade
Source: Org Lett. 2024 Nov 4;26(51):11085–9. doi: 10.1021/acs.orglett.4c03504 (PMC11686507; doi:10.1021/acs.orglett.4c03504)
Supplement: Supplementary file 1 — ol4c03504_si_001.pdf [file ol4c03504_si_001.pdf]

# Stereodivergent Synthesis of 6,12-Guaianolide C1 Epimers via a Rationally Designed Oxy-Cope/Ene Reaction Cascade

Kalliopi Mazaraki, Christos Zangelidis, Antonis Kelesidis and Alexandros L. Zografos*

Laboratory of Organic Chemistry, Department of Chemistry, Aristotle University of Thessaloniki, Main University Campus, 54124, Thessaloniki, Greece

**Corresponding Authors:**

E-mail: alzograf@chem.auth.gr

**Supporting Information**

## Table of Contents


1. Abbreviations ... 1

1. Material and methods ... 2

2. Synthetic plan synopsis ... 3

3. Experimental procedures ... 4

   1. Synthesis of compound S1 via introduction of the vinyl chain to commercially available R-carvone[1] ... 4

   2. Allylic chlorination and Kornblum oxidation[2,3] ... 6

      3. Pinnick oxidation[4], subsequent lactonization and protection of the Michael system ... 8

      4. Alkylation: Introduction of the isopropenyl chain ... 11

      5. oxy-Cope/ ene reaction ... 14

      4. Final steps towards epoxy-osmitopsin (20) ... 20

      5. Final steps towards 6-*epi*-cichopumilide (3) & neoartabsin (27) ... 26

5. Spectra ... 32

6. References ... 78

## 1. Abbreviations

| | |
|---|---|
| **Ac** | Acetyl- |
| **acac** | Acetylacetonate |
| **Bnz** | Benzene |
| **DCE** | Dichloroethane |
| **DCM** | Dichloromethane |
| **DMAP** | 4-Dimethylaminopyridine |
| **DMF** | Dimethylformamide |
| **DMSO** | Dimethylsulfoxide |
| **Et** | Ethyl- |
| **Hex** | Hexane |
| ***L*-selectride** | Lithium tri-sec-butylborohydride |
| ***m*-CPBA** | *meta*-Chloroperoxybenzoic acid |
| **MeCN** | Acetonitrile |
| **Ms** | Mehtanesulfonyl- (mesyl-) |
| **NBS** | *N*-bromosuccinimide |
| **NMR** | Nuclear magnetic reasonanse |
| **o/n** | Overnight |
| ***p*** | *para-* |
| **Ph** | Phenyl- |
| **ppm** | Parts per million |
| **PS** | Petroleum ether |
| ***p*-TSA** | *p*-toluenesulfonic acid |
| **$^{t}$Bu** | ($^{tert}$)butyl- |
| **TFA** | Trifluoroacetic acid |
| **THF** | Tetrahydrofuran |
| **TLC** | Thin layer chromatography |
| **Tol** | Toluene |
| **UV** | Ultraviolet |

## 1. Material and methods

All reactions were carried out under an argon (Ar) atmosphere with dry solvents under anhydrous conditions. Anhydrous solvents were either obtained from commercial sources or dried accordingly. Dry tetrahydrofuran (THF) was obtained by refluxing the solvents with sodium metal as drying agent and benzophenone as indicator for several hours, whereas methylene chloride (DCM) from $CaH_2$. The solvents were kept under Ar using molecular sieves 4Å in their bottles. Petroleum ether refers to the 40-60°C boiling fraction. Commercially available reagents were purchased at the highest commercial quality and used without further purification or where specified, purified by standard techniques.

Reactions were monitored by thin-layer chromatography (TLC) carried out on S-2 0.25 mm E. Merck silica gel plates (60F-254) using UV light as visualizing agent ($\lambda_{max}$= 254 nm or 360 nm) and ethanolic *p*-anisaldehyde as developing agent or by Seebach TLC stain solution, followed by heating. E. Merck silica gel (60, particle size 0.040–0.063 mm) was used for flash column chromatography. Preparative TLC plates (S-2 0.5mm E. Merck silica gel plates precoated with silica gel 60-F254) were used in cases where the separation with usual flash column chromatography were inadequate. Molecular mechanics (MM2) energy calculations were performed with Chem3D 18.0 chemical software. NMR spectra were recorded at 298 K using an Agilent Technologies DD2 500 spectrometer and calibrated by residual solvent peaks. $^1H$ NMR spectra were recorded at 500 MHz and residual solvent peaks were used as an internal reference ($CDCl_3$ δ 7.26). Data are reported as follows: chemical shift in ppm, multiplicity (s = singlet, brs = broad singlet, d = doublet, brd = broad doublet, t = triplet, brt = broad triplet, q = quartet, m = multiplet or overlap of nonequivalent resonances, coupling constants are reported in Hz, integration is included. $^{13}C$ NMR spectra were recorded at 125 MHz and residual solvent peaks were used as an internal reference ($CDCl_3$ δ 77.00). Data are reported as follows: chemical shift in ppm, multiplicity deduced. The assignment of $^1H$ and $^{13}C$ signals was assisted by COSY, HSQC, HMBC and NOESY experiments where necessary. High-resolution mass spectra (HRMS) were recorded on an Agilent ESI-TOF (time of light) mass spectrometer at a 4000V emitter voltage. Melting points were obtained by Stuart Melting Point Apparatus SMP3, Bibby Scientific. . Heating conditions were achieved with IKA magnetic stirring-heating plates in an oil bath.

## 2. Synthetic plan synopsis

Our goal was primarily the synthesis of a divergent elemanolide scaffold, to target consequently both *Apiaceae*- and *Asteraceae*-type sesquiterpenoids. This common scaffold **5** was envisioned to derive from commercially available *R*-carvone, via a series of reactions. The key step of this route involves a sequence of oxy-Cope/ ene reactions, yielding to the two desired C1 epimers **11** and **12**. Further transformations, such as hydration, reduction and dehydration, on these skeletons led to the final osmitopsin-type compounds 4,5*α*-epoxy-osmitopsin **20**, and 6-*epi*-cichopumilide **3**.

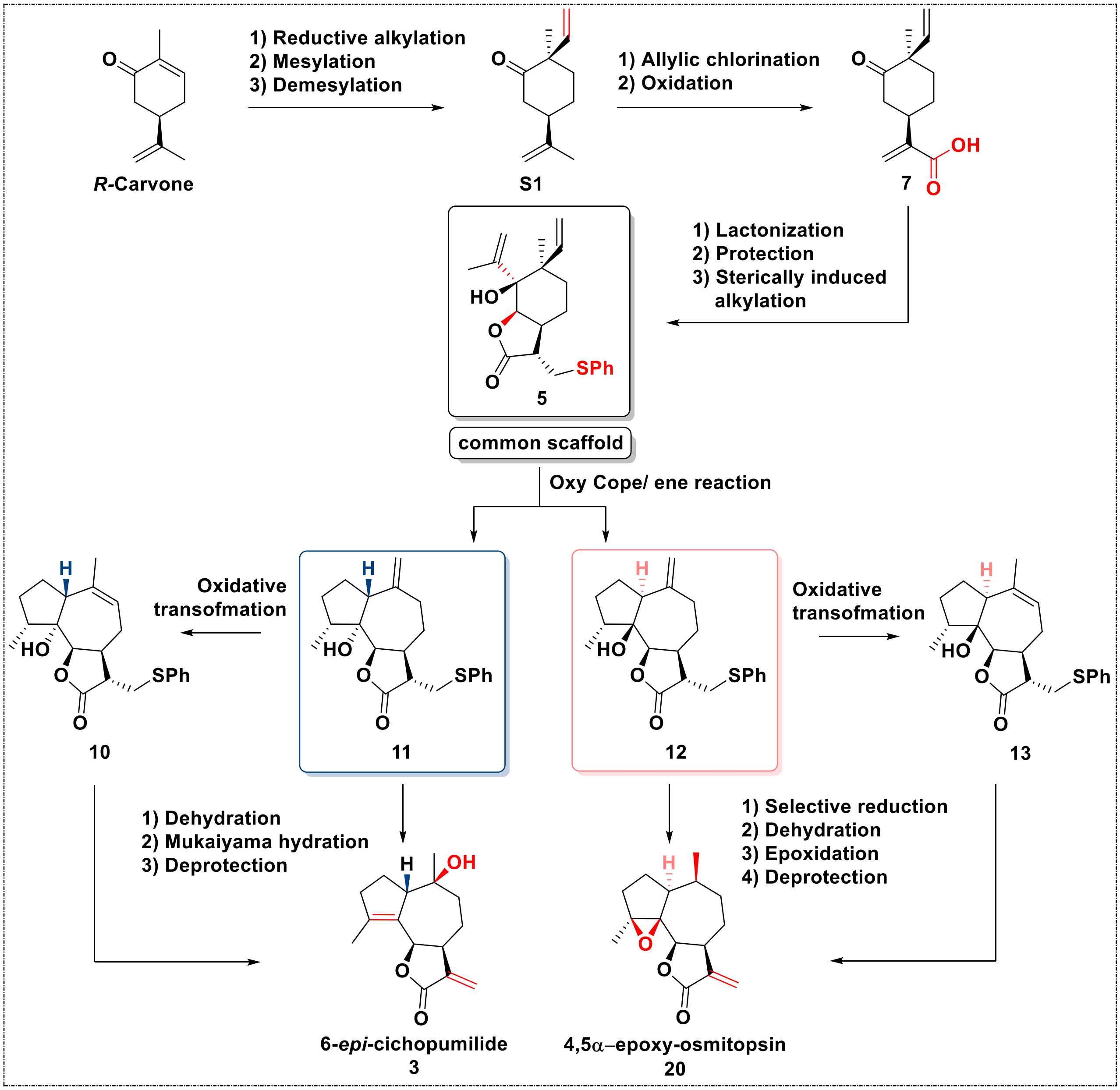

# 3. Experimental procedures

## 1. Synthesis of compound S1 via introduction of the vinyl chain to commercially available R-carvone[1]

The synthetic route begins with the conjugated addition of acetaldehyde to the commercially available *R*-carvone, followed by mesylation of the resulting alcohols. Elimination of the mesyl- group using lithium salts produces the desired alkene **SI1** in 10:1 diastereoselectivity favoring the *β*-vinyl isomer.[1]

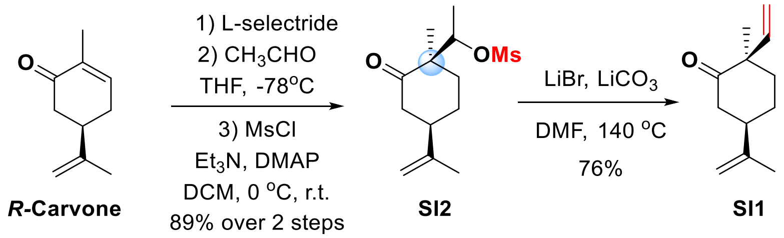


**Scheme SI1**. Introduction of the vinyl chain

### (S)-1-((1R,4R)-1-methyl-2-oxo-4-(prop-1-en-2-yl)cyclohexyl)ethyl methanesulfonate (SI2)[1]

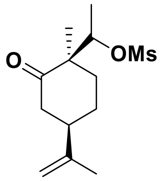


**SI2**

**MW**: 274.38 g/mol

**Molecular Formula**: $C_{13}H_{22}O_4S$

To a flame-dried round-bottom flask, (*R*)-carvone (4.0 g, 26.6 mmol, 1 eq) was dissolved in dry THF (50 mL) under argon atmosphere and was cooled to -78°C. *L*-selectride (1.1 eq, 29.3 mL, 1M in THF) was then added slowly and the resulting mixture was stirred at -78°C for 1h (the consumption of the starting material was monitored by TLC). Then, acetaldehyde (5 mL, 83.07 mmol, 4.0 eq) was added at -78°C. The resulting mixture was stirred for an additional hour at the same temperature before an aqueous solution of $H_2O_2$ (30% w/w, 32 mL) and NaOH (2N, 40 mL) was added dropwise to the reaction mixture, which was allowed to warm at room temperature. The aqueous layer was then extracted with EtOAc (3 x 50mL). The combined organic extracts were washed twice with saturated aqueous $Na_2S_2O_3$, dried over $Na_2SO_4$, filtered and the solvent was removed *in vacuo* to provide the diastereomers of the desired secondary alcohols that were used without further purification $^1$H NMR (500 MHz, $CDCl_3$): $\delta_H$ = 4.82 (s, 1H), 4.68 (s, 1H), 4.13 (q, J = 6.4 Hz, 1H), 2.60 – 2.53 (m, 3H), 2.33 – 2.29 (m, 1H), 1.90 – 1.78

(m, 3H), 1.74 (s, 3H), 1.70 (dd, J = 8.4, 4.0 Hz, 1H), 1.50 – 1.43 (m, 2H), 1.11 (d, J = 6.4 Hz, 3H), 1.06 (s, 3H); $^{13}C$ NMR (125 MHz, $CDCl_3$): $\delta_C$ = 217.4, 146.9, 111.2, 70.5, 53.1, 44.4, 43.9, 33.3, 24.6, 21.3, 18.4, 16.6.

To a round-bottom flask DMAP (2.2 g, 18.2 mmol, 0.55 eq) and $Et_3N$ (27.7 mL, 198.6 mmol, 6.0 eq) are dissolved in DCM. The resulting crude (7.0 g, 35.3 mmol, 1.0 eq) was dissolved in dry DCM (60 mL) and was added to the previously prepared solution of bases at 0 °C. Methanesulfonyl chloride (7.7 mL, 99.3 mmol, 3.0 eq) was then added dropwise and the reaction mixture was allowed to warm slowly at room temperature and was stirred for 12h. The reaction was quenched with saturated aqueous $NH_4Cl$ (45 mL), the phases were separated, the aqueous layer was extracted three times with DCM (3x45 mL) and the combined organic extracts were washed with saturated aqueous $NH_4Cl$, dried over $Na_2SO_4$ and filtered. The solvent was removed *in vacuo* and the residue was purified by column chromatography on silica gel, elution with PS:EtOAc 12:1 (TLC: $R_f$ = 0.42 upon PS:EtOAc 3:1, UV inactive on TLC, stains brown upon *p*-anisaldehyde staining) afforded pure compound **SI2** (6.5 g, 89%), as yellow oil. $^1H$ NMR (500 MHz, $CDCl_3$): $\delta_H$ = 5.37 (q, J = 6.4 Hz, 1H), 4.81 (s, 1H), 4.72 (s, 1H), 2.95 (s, 3H), 2.74 (dd, J = 14.1, 11.2 Hz, 1H), 2.49 (ddd, J = 14.2, 4.4, 1.5 Hz, 1H), 2.46 – 2.40 (m, 1H), 1.95 (ddd, J = 14.4, 4.9, 3.9 Hz, 1H), 1.83 – 1.77 (m, 1H), 1.75 (s, 3H), 1.56 – 1.49 (m, 1H), 1.44 (d, J = 6.4 Hz, 3H), 1.11 (s, 3H); $^{13}C$ NMR (125 MHz, $CDCl_3$): $\delta_C$ = 211.9, 146.7, 110.7, 80.2, 52.0, 45.6, 43.2, 38.9, 34.3, 25.3, 20.7, 17.5, 15.9;

**(2R,5R)-2-methyl-5-(prop-1-en-2-yl)-2-vinylcyclohexan-1-one (SI1)**[1]

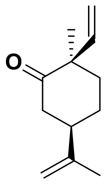


**SI1**

**MW**: 178.28 g/mol

**Molecular Formula**: $C_{12}H_{18}O$

To a flame-dried round-bottom flask, anhydrous LiBr (10.3 g, 118.4 mmol, 5.0 eq) and anhydrous $Li_2CO_3$ (10.5 g, 142.1 mmol, 6.0 eq) were added. Compound **SI2** (6.5 g, 23.7 mmol 1.0 eq) was dissolved in dry DMF (100 mL). The resulting mixture was refluxed in oil bath at 140 °C under argon atmosphere for 4.5h. After being cooled down, the reaction was quenched with water (200 mL), the phases were separated and the aqueous layer was extracted three times with EtOAc (3x200 mL). The combined organic extracts were dried over $Na_2SO_4$, filtered and the solvent was removed *in vacuo*. The residue was purified by column chromatography on silica gel, elution with

PS:EtOAc 70:1 (TLC: $R_f$ = 0.56 upon PS:EtOAc 10:1, UV inactive on TLC, stains brown upon *p*-anisaldehyde staining afforded compound **SI1** (3.2 g, 76%), as yellow oil. $[\alpha]^{25}_{D}$= +96.0 (c 0.01 $CHCl_3$); $^1$H NMR (500 MHz, $CDCl_3$): $\delta_H$ = 5.92 (dd, *J* = 17.6, 10.7 Hz, 1H), 5.16 (d, *J* = 10.8 Hz, 1H), 5.01 (d, *J* = 17.7 Hz, 1H), 4.75 (s, 1H), 4.72 (s, 1H), 2.56 (t, *J* = 13.9 Hz, 1H), 2.40 – 2.29 (m, 2H), 2.06 (dt, *J* = 13.6, 3.5 Hz, 1H), 1.81 – 1.73 (m, 2H), 1.73 (s, 3H), 1.65 – 1.56 (m, 1H), 1.15 (s, 3H) $^{13}$C NMR (125 MHz, $CDCl_3$): $\delta_C$ = 212.6, 147.6, 142.3, 115.6, 109.7, 51.7, 46.7, 44.2, 38.8, 27.1, 24.0, 20.4.

## 2. Allylic chlorination and Kornblum oxidation[2,3]

After the desired chain was introduced, we proceeded to the allylic chlorination of compound **SI1** with $Ca(OCl)_2$ and the resulting compound **6** was subjected to Kornblum oxidation, yielding aldehyde **SI3**.

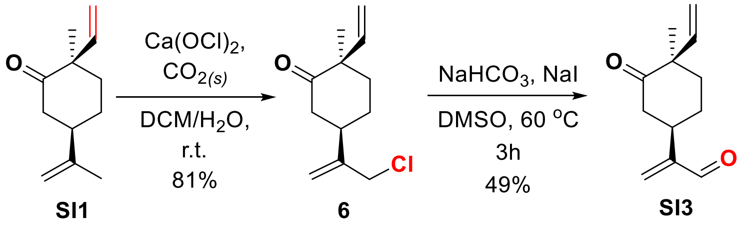


**Scheme SI2**. Chlorination of **SI1** and subsequent oxidation

**(2R,5R)-5-(3-chloroprop-1-en-2-yl)-2-methyl-2-vinylcyclohexan-1-one (6)**

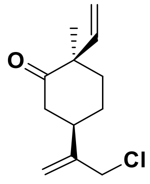


**6**

**MW**: 212.72 g/mol

**Molecular Formula**: $C_{12}H_{17}ClO$

To a round-bottom flask, $Ca(OCl)_2$ (2.5 g, 17.9 mmol, 1.0 eq) was suspended in water (50 mL) and small pieces of dry ice were added inside the flask so that the mixture was chilled. Solution of compound **SI1** (3.2 g, 17.9 mmol, 1.0 eq) in DCM (70 mL) was then added slowly while small pieces of dry ice were added regularly to maintain the reaction mixture cool. After stirring for 2.5h approximately (the consumption of the starting material was monitored by TLC), the reaction mixture was filtered *in vacuo* via Buchner funnel to remove the insoluble inorganic components and the resulting filtrate was transferred to a separation funnel. The layers were separated and the aqueous layer was extracted three times with DCM (3x45 mL). The combined organic extracts were dried over

$Na_2SO_4$, filtered and the solvent was removed *in vacuo*. The residue was purified by column chromatography on silica gel, elution with PS:EtOAc 70:1 (TLC: $R_f$ = 0.50 upon PS:EtOAc 10:1, UV inactive on TLC, stains blue upon *p*-anisaldehyde staining) afforded compound **6** (3.15 g, 83%), as yellow oil. $[\alpha]^{25}_D$= +93.0 (c 0.01 $CHCl_3$); $^1H$ NMR (500 MHz, $CDCl_3$) $\delta_H$ = 5.92 (dd, *J* = 17.7, 10.7 Hz, 1H), 5.22 (s, 1H), 5.18 (d, *J* = 10.7 Hz, 1H), 5.03 (s, 1H), 5.00 (d, 1H), 4.08 (dd, 2H), 2.66 – 2.53 (m, 2H), 2.44 (dt, *J* = 12.9, 2.9 Hz, 1H), 2.08 (dq, *J* = 12.6, 5.5, 4.5 Hz, 1H), 1.93 – 1.86 (m, 1H), 1.78 (ddd, *J* = 13.6, 8.6, 3.4 Hz, 1H), 1.73 – 1.58 (m, 1H), 1.16 (s, 3H). $^{13}C$ NMR (125 MHz, $CDCl_3$) $\delta_C$ = 211.6, 147.5, 142.1, 115.8, 114.5, 51.6, 47.0, 44.2, 42.00, 38.6, 27.4, 24.0. HRMS (ESI, m/z): calcd for $C_{12}H_{17}ClOK^+$($[M+K]^+$): 251.0600, found 251.0602

**2-((1R,4R)-4-methyl-3-oxo-4-vinylcyclohexyl)acrylaldehyde (SI3)**

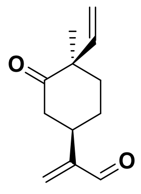


SI3

**MW**: 192.26 g/mol

**Molecular Formula**: $C_{12}H_{16}O_2$

To a round-bottom flask, compound **6** (3.15 g, 14.8 mmol, 1.0 eq) was dissolved in DMSO (50 mL). Then, $NaHCO_3$ (2.48 g, 29.6 mmol, 2.0 eq) and NaI (3.33 g, 22.2 mmol, 1.5 eq) were added and the resulting mixture was refluxed in oil bath at 60 °C under argon atmosphere for approximately 3h. After being cooled down, the reaction was quenched with water (150 mL), the phases were separated and the aqueous layer was extracted three times with EtOAc (3x100 mL). The combined organic extracts were dried over $Na_2SO_4$, filtered and the solvent was removed *in vacuo*. The residue was purified by column chromatography on silica gel, elution with PS:EtOAc 15:1 (TLC: $R_f$ = 0.49 upon PS:EtOAc 3:1, UV inactive on TLC, stains green upon *p*-anisaldehyde staining) afforded compound **S3** (1.26 g, 44%), as an unstable yellow-green oil. $[\alpha]^{25}_D$= +120.0 (c 0.002 $CHCl_3$); $^1H$ NMR (500 MHz, $CDCl_3$): $\delta_H$ = 9.53 (s, 1H), 6.25 (s, 1H), 6.05 (s, 1H), 5.93 (dd, *J* = 17.7, 10.3 Hz, 1H), 5.18 (d, *J* = 10.7 Hz, 1H), 5.02 (d, *J* = 17.6 Hz, 1H), 2.93 (t, *J* = 12.2 Hz, 1H), 2.62 (t, *J* = 13.4 Hz, 1H), 2.36 (dt, *J* = 13.6, 3.3 Hz, 1H), 2.06 (dt, *J* = 13.6, 3.1 Hz, 1H), 1.89 – 1.79 (m, 1H), 1.77 (dd, *J* = 11.8, 3.0 Hz, 1H), 1.75 – 1.63 (m, 1H), 1.17 (s, 3H); $^{13}C$ NMR_(125 MHz, $CDCl_3$): $\delta_C$ = 211.1, 193.7, 152.3, 142.1, 133.5, 115.7, 51.6, 43.1, 38.6, 37.6, 27.2, 24.0.

## 3. Pinnick oxidation[4], subsequent lactonization and protection of the Michael system

After the desired aldehyde was obtained, we proceeded to transforming aldehyde **SI3** to carboxylic acid **7**, via a facile Pinnick oxidation. The carboxylic acid **7** was then subjected to a lactonization reaction. After attempting a series of conditions reported below, we achieved this lactonization via a photochemical reaction in the presence of NBS. The Michael system on the resulting lactone **8** was then protected with the bulky thiophenyl group, giving rise to compound **9**.

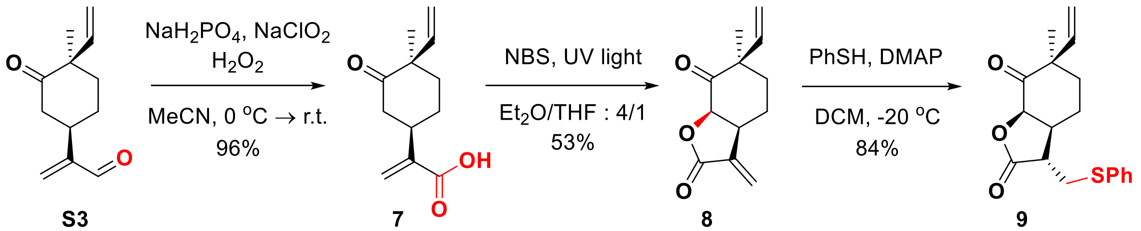


**S3** **7** **8** **9**

**Scheme SI3**. Lactone formation and protection of the Michael system

**2-((1R,4R)-4-methyl-3-oxo-4-vinylcyclohexyl)acrylic acid**[5]

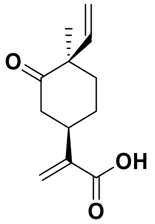


**7**

**MW**: 208.26 g/mol

**Molecular formula**: $C_{12}H_{16}O_3$

To a round-bottom flask, compound **S3** (1.26 g, 6.6 mmol, 1.0 eq) was dissolved in MeCN (30 mL) and was cooled at 0 °C before aqueous solution of $NaH_2PO_4$ (0.37 g, 3.0 mmol, 0.45 eq) was added. After slow addition of $H_2O_2$ (30%, 1.53 mL, 1.3 eq) the resulting mixture was stirred for 5 minutes and aqueous solution of $NaClO_2$ (1.19 g, 13.2 mmol, 2.0 eq) was added dropwise. The resulting mixture was allowed to warm at room temperature and was stirred for 1h. The solvent was removed *in vacuo*. After addition of saturated aqueous solution of $NaHCO_3$, until pH=9 (the pH was monitored with pH indicator paper) the layers were separated and the aqueous layer was extracted three times with DCM (3x30 mL). The aqueous extract was collected and and acidified with HCl (6N), to pH=3 The aqueous layer was extracted three times with EtOAc (3x30 mL). The combined organic extracts were

dried over $Na_2SO_4$, filtered and the solvent was removed *in vacuo*, yielding compound **7** (1.32 g, 96%), as thick, colorless liquid. (TLC: $R_f$ = 0.32 upon PS:EtOAc 3:1, UV inactive on TLC, stains blue upon Seebach staining); $[\alpha]^{25}_{D}$= +200.0 (c 0.002 $CHCl_3$); $^1H$ NMR (500 MHz, $CDCl_3$): $\delta_H$ = 6.38 (s, 1H), 5.92 (dd, *J* = 17.6, 10.8 Hz, 1H), 5.68 (s, 1H), 5.18 (d, *J* = 10.6 Hz, 1H), 5.02 (d, *J* = 17.7 Hz, 1H), 2.95 – 2.83 (m, 1H), 2.60 (t, *J* = 13.4 Hz, 1H), 2.43 (ddd, *J* = 13.6, 3.9, 2.2 Hz, 1H), 2.07 (dt, *J* = 13.7, 3.3 Hz, 1H), 1.89 (ddd, *J* = 13.1, 6.3, 3.4 Hz, 1H), 1.85 – 1.75 (m, 1H), 1.67 (td, *J* = 13.4, 3.8 Hz, 1H), 1.17 (s, 3H); $^{13}C$ NMR_(125 MHz, $CDCl_3$): $\delta_C$ = 211.7, 171.7, 142.5, 142.1, 126.3, 115.8, 51.6, 43.9, 40.2, 38.5, 27.6, 24.0.

As shown in **Table 1**, a series of reactions was carried out in order to obtain the desired lactone **8**. Use of benzene as solvent in the absence of irradiation during heating gave a complex mixture. Change of solvent to diethyl ether while reducing the temperature, the reaction time and the doses of NBS added gave similar results. Maintaining these conditions while significantly reducing the reaction time gave rise to lactone **8** in low yields. Better results were observed when the addition of NBS was conducted periodically via syringe pump, lasting in total 1 hour, yielding to the desired lactone **8**.

**Table 1. Lactonization attempts of carboxylic acid 7**

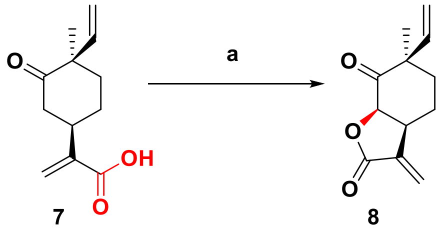


| a/a | NBS addition | Irratiation | Solvent | Temperature | Reaction Time (including NBS addition time) | Products (Yield) |
|---|---|---|---|---|---|---|
| **1** | 2 doses | - | PhH | 80 °C | 5.5 h | Complex mixture |
| **2** | 1 dose | UV | $Et_2O$ | r.t. | 4 h | Complex mixture |
| **3** | 1 dose | UV | $Et_2O$ | r.t. | 3.5 h | **8** (14%) |
| **4** | 0.2 mL/min for 15’ | UV | $Et_2O$/THF 4 eq./1 eq. | r.t. | 3 h | **8** (4%) |

| | | | | | | |
|---|---|---|---|---|---|---|
| **5** | 0.233 mL/min for 60’ | UV | $Et_2O$/THF 4 eq./1 eq. | r.t. | 3.5 h | **8** (53%) |

**(3aS,6R,7aR)-6-methyl-3-methylene-6-vinyltetrahydrobenzofuran-2,7(3H,4H)-dione (8)**

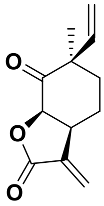

**8**

**MW**: 206.24 g/mol

**Molecular Formula**: $C_{12}H_{14}O_3$

To a 80 ml test tube, compound **7** (400 mg, 1,92 mmol) was dissolved in $Et_2O$ (56 mL), and the tube was carefully closed with rubber septum under argon atmosphere (at any given moment the rubber septum should not encounter with the solution in the tube). The solution was placed under UV light. NBS (341,8 mg, 1,92 mmol, 1 eq) was dissolved in THF (14 mL) and was taken in a 20 ml syringe covered and protected from visible light with aluminum foil. The syringe was placed in a syringe pump and programmed for addition of its content over (rate: 0.233 mL/min) into the irradiated test tube. The resulting mixture was stirred for 2,5 h under UV light after the addition of NBS. The reaction was quenched with saturated aqueous solution of $NaHCO_3$ (3x60 mL) until pH=9 (the pH was monitored with pH indicator paper), the phases were separated and the aqueous layer was extracted three times with EtOAc (3x60 mL).

- The aqueous extract was collected and acidified with HCl (6N) at pH=3 to neutralize the non-reactant starting material. After acidification, the aqueous layer was extracted three times with EtOAc (3x60 mL). The combined organic extracts were dried over $Na_2SO_4$, filtered and the solvent was removed *in vacuo*. The retrieved carboxylic acid **7** underwent a next round of lactonization following the described protocol.
- The combined organic extracts from lactonization reaction were dried over $Na_2SO_4$, filtered and the solvent was removed *in vacuo*. The residue was dissolved in PhH (8 mL) and washed once with water (8 mL) and once with saturated aqueous solution of NaCl (8 mL). The combined organic extracts were dried over $Na_2SO_4$, filtered and the solvent was removed *in vacuo*.

The lactones from every individual lactonization were combined and purified by column chromatography on silica gel, elution with PS:EtOAc 3:1 (TLC: $R_f$ = 0.41 upon PS:EtOAc 3:1, UV active on TLC, stains green upon stains blue

upon Seebach staining) afforded compound **8** (210.0 mg, 53%), as light yellow solid. $[\alpha]^{25}_D$= -137.78 (c 0.005 $CHCl_3$); $^1H$ NMR (500 MHz, $CDCl_3$): $\delta_H$ = 6.31 (d, *J* = 3.1 Hz, 1H), 6.03 (dd, *J* = 17.6, 10.9 Hz, 1H), 5.61 (d, *J* = 2.7 Hz, 1H), 5.17 (d, *J* = 10.8 Hz, 1H), 5.08 (d, *J* = 10.2 Hz, 1H), 5.05 (d, *J* = 2.0 Hz, 1H), 3.73 – 3.63 (m, 1H), 2.30 – 2.19 (m, 1H), 2.06 – 1.95 (m, 1H), 1.87 – 1.74 (m, 2H), 1.34 (s, 3H); $^{13}C$ NMR (125 MHz, $CDCl_3$): $\delta_C$ = 206.9, 169.0, 139.9, 136.1, 122.4, 114.2, 77.9, 50.3, 42.4, 33.1, 23.1, 22.6; HRMS (ESI, m/z): calcd for $C_{12}H_{14}O_3K^+$([M+K]$^+$): 245.0575, found: 245.0574

**(3S,3aS,6R,7aR)-6-methyl-3-((phenylthio)methyl)-6-vinyltetrahydrobenzofuran-2,7(3H,4H)-dione (9)**

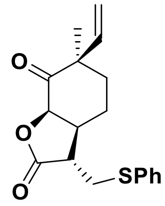

**9**

**MW**: 316.42 g/mol

**Molecular Formula**: $C_{18}H_{20}O_3S$

To a flame-dried round-bottom flask, compound **8** (325.0 mg, 1.58 mmol, 1.0 eq) was dissolved in DCM (30 mL) and cooled at -20 °C under argon atmosphere. DMAP (28.8 mg, 0.23 mmol, 0.23 eq) and thiophenol (0.18 mL, 1.73 mmol, 1.1 eq) were added to the solution. The resulting mixture was stirred for 2.5h. The solvent was removed *in vacuo* and the residue was purified by column chromatography on silica gel, elution with PS:EtOAc 3:1 (TLC: $R_f$ = 0.35 upon PS:EtOAc 1.5:1, UV active on TLC, stains blue upon Seebach staining) afforded pure compound **9** (420.0 mg, 84%), as light-yellow oil. $[\alpha]^{25}_D$= -50.5 (c 0.0063 $CHCl_3$); $^1H$ NMR (500 MHz, $CDCl_3$): $\delta_H$ = 7.36 (d, *J* = 7.7 Hz, 2H), 7.31 (t, *J* = 7.6 Hz, 2H), 7.23 (t, *J* = 7.3 Hz, 1H), 6.05 (dd, *J* = 17.6, 10.9 Hz, 1H), 5.16 (d, *J* = 10.9 Hz, 1H), 5.08 (d, *J* = 9.0 Hz, 1H), 5.04 (d, *J* = 17.6 Hz, 1H), 3.52 (dd, *J* = 13.8, 3.9 Hz, 1H), 3.14 (m, 1H), 3.01 (dd, *J* = 13.9, 8.3 Hz, 1H), 2.44 (ddd, *J* = 11.7, 8.3, 3.9 Hz, 1H), 2.24 – 2.18 (m, 1H), 1.93 – 1.84 (m, 1H), 1.78 (d, *J* = 2.2 Hz, 2H), 1.32 (s, 3H).; $^{13}C$ NMR (125 MHz, $CDCl_3$): $\delta_C$ = 207.5, 175.3, 139.7, 134.7, 129.8, 129.3, 126.9, 114.1, 78.1, 50.5, 44.5, 42.2, 33.7, 33.4, 22.6, 22.2. HRMS (ESI, m/z): calcd for $C_{18}H_{20}O_3SNa^+$([M+Na]$^+$): 339.1025, found: 339.1026

## 4. Alkylation: Introduction of the isopropenyl chain

The introduction of the desired isopropenyl group was a crucial step to our synthetic plan. After having carefully studied different molecular models, we concluded that, in order for the following oxy Cope/ene reaction to be successful, the isopropenyl chain should be introduced solely from the *α*-side of the carbonyl. Our initial thought was to use isopropenyl lithium (**SI5**) prepared by the respective bromide in the presence of $^tBuLi$. However, the

yield of this reaction was low and returned a mixture of the two diastereomers, **5** and **SI4**, in a ratio 2:1. We then tried the use of the organomagnesium reagent **SI6** (prepared by the respective bromide in the presence of magnesium metal), but in this case the product observed was only compound **SI4**, in a moderate yield. Fortunately, we had more satisfying results when we used organocerium compound **SI7** (please advise the experimental section for details). In this case, the yield was optimized and the ratio of the two diastereomers was 4:1. The results are exhibited in **Table 2**.

**Table 2. Alkylation attempts of the protected lactone 9**

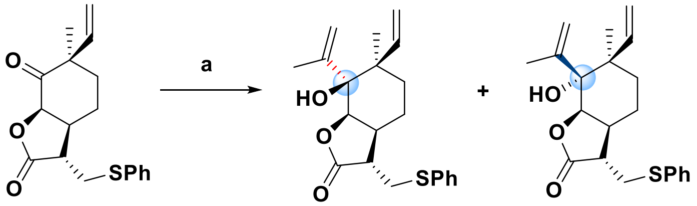


**9** **5** **SI4**

| a | Alkylating reagent | Product (Yield) |
|---|---|---|
| **1** | 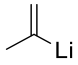  **SI5** | **5:SI4** / 2:1 (50%) |
| **2** | 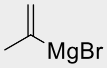  **SI6** | **SI4** (60%) |
| **3** | 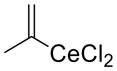  **SI7** | **5:SI4** / 4:1 (66%) |

**(3S,3aS,6R,7S,7aR)-7-hydroxy-6-methyl-3-((phenylthio)methyl)-7-(prop-1-en-2-yl)-6-vinylhexahydrobenzo furan-2(3H)-one (5)**

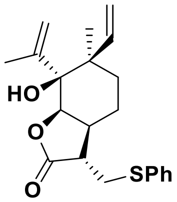


**5**

**MW**: 358.50 g/mol

**Molecular Formula**: $C_{21}H_{26}O_3S$

**Organocerium reagent synthesis***:*

*Drying and activate $CeCl_3$:* To a flame-dried Schlenk tube, cerium (III) chloride (1.17g, 4.7 mmol) was stirred *in high vacuo (<$10^{-5}$)* under argon in oil bath at 140 °C for 5h and then was allowed to return at room temperature. The Schlenk tube was cooled in an ice bath (0 °C), and dry, degassed THF (8,8 mL) was introduced at once to cerium (III) chloride to activate it under an argon atmosphere.The dispersion was stirred for 12h at rt.

*Grignard synthesis:* To a flame-dried round-bottom flask, Mg (316.0 mg, 13.0 mmol) was burned *in vacuo* for activation and dispersed in dry THF (1 mL) under argon atmosphere. To another flame-dried round-bottom flask, 2-bromopropene (1 mL, 0.11 mmol) was dissolved in dry THF under argon atmosphere. Part of the halide solution (1 mL) was added to the flask with the metal under vigorous stirring, until the solution got warm and turned brown-black (initiation points varies from few minutes to 30min depending on magnesium condition). The remaining solution of allylhalide was added from the top of the reflux condenser dropwise to maintain a gentle reflux in the mixture. As soon as the addition completed, the flask was placed in reflux (70 °C) under argon atmosphere and. The resulting mixture was stirred for 30 min and was allowed to return at room temperature before it was used.

*Transmetallation:* The Schlenk tube with the cerium (III) chloride dispersed in THF was cooled at -78 °C and the Grignard reagent (0.84M, 5.5 mL) was added dropwise. The resulting mixture was stirred for 1h at -78 °C.

To a flame-dried round-bottom flask, compound **9** (512.0 mg, 1.63 mmol, 1.0 eq was azeotropically dried over PhH (x3). Then, it was dissolved in THF (5 mL) under argon atmosphere and cooled at -78 °C. The cooled at -78 °C organocerium compound (10.2 mL, 3.26 mmol, 2.0 eq) was added at once in the flask containing **9** and the resulting mixture was stirred for 1h. The reaction was quenched with $NH_4Cl$ (15 mL) and the aqueous layer was extracted three times with EtOAc (3x30 mL). The combined organic extracts were dried over $Na_2SO_4$, filtered and the solvent was removed *in vacuo*. The residue was purified by column chromatography on silica gel, elution with PS:$Et_2O$ (3:1) (TLC: $R_f$ = 0.65 upon PS:$Et_2O$ 1:1, UV active on TLC, stains blue upon seebach staining) afforded compound **5** (385.0 mg, 66%), as white solid. $[\alpha]^{25}_D$= -24.0 (c 0.01 toluene); $^1$H NMR (500 MHz, $CDCl_3$): $\delta_H$ = 7.38 (d, *J* = 7.8 Hz, 2H), 7.30 (t, *J* = 7.6 Hz, 2H), 7.21 (d, *J* = 7.4 Hz, 1H), 6.03 (dd, *J* = 17.8, 10.9 Hz, 1H), 5.15 (s, 1H), 5.06 (d, *J* = 10.9 Hz, 1H), 5.01 – 4.91 (m, 3H), 3.46 (dd, *J* = 13.5, 4.1 Hz, 1H), 3.22 (ddd, *J* = 12.0, 7.3, 4.1 Hz, 1H), 3.07 (dd, *J* = 13.5, 7.2 Hz, 1H), 2.82 – 2.76 (m, 1H), 2.04 (dddd, *J* = 27.9, 16.0, 10.8, 4.5 Hz, 2H), 1.86 (s, 3H), 1.83 (d, *J* = 5.1 Hz, 1H), 1.12 – 1.04 (m, 2H), 1.03 (s, 3H).; $^{13}$C NMR (125 MHz, $CDCl_3$): $\delta_C$ = 178.4, 146.2, 143.7, 136.0, 129.4,

129.1, 126.4, 116.0, 113.2, 78.6, 77.9, 43.9, 42.5, 39.2, 34.5, 27.4, 22.9, 20.0, 19.4. HRMS (ESI, m/z): calcd for $C_{21}H_{26}O_3SNa^+$([M+Na]$^+$): 381.1495, found: 381.1494

When the same experimental procedure was conducted in the presence of dry LiCl (1 equiv) diluted in the flask containing **9,** compound **5** was delivered in a much better diastereoselectivity reaching 15:1 ratio and an overall yield of 63%. The described reaction is quite tricky and depends on the dryness of LiCl used. In cases where LiCl is not completely dry the reaction provides a bad profile with many unidentified products.

## 5. oxy-Cope/ ene reaction

Compound **5** can be used as a common scaffold to access the two main skeletons of *Apiaceae* (*β*-hydrogen) and *Asteraceae* families (*α*-hydrogen). These skeletons were envisioned to be approached via a sequence of oxy Cope/ ene reactions.

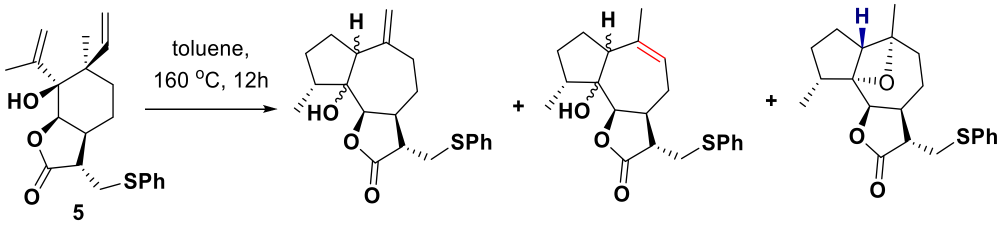


**Scheme SI4**. Key step: oxy-Cope/ ene reactions

The observed stereochemistry of the resulting guaianolides can be explained based on the mechanism of the reaction. The thermal oxy-Cope reaction of the common elemanolide scaffold **5** with the proper stereochemistry, where the vinyl- and the isopropenyl- chains form an imaginary chair conformation, leads to germacranolide **SI8**, which was not isolated and after enol-ketone tautomerization gives rise to compound **4**.

In order for the ene reaction to take place, the vinyl- methyl and the carbonyl should be aligned. This can happen in two ways: either after carbonyl rotation, which leads to conformation A and consequently to *Apiaceae*-type skeleton **11**, or after double bond rotation, which leads to conformation B and therefore *Asteraceae*-type skeleton **12**. The products received were in accordance with our predictions and the mechanism explanation is shown in **Scheme SI5**. Compound **10** can derive from compound **11**, after double bond isomerization, due to oxidative decomposition commented in the manuscript.

In more details, when the thermal oxy-Cope/ene reaction was conducted at 160 °C in the presence of dioxygen, only the isomerized guaianolide **10** was obtained in 41% yield, suggesting an oxidative decomposition and/or

transformation of **12** to **11** before the isomerization step (Scheme 2B). To substantiate our hypothesis, heating a pure sample of **11** in toluene in the presence of dioxygen at 150 $^{o}$C led to the isolation of the stable peroxide **14** and the isomerized product **10**, indicating a thermal opening of the peroxide bridge to **10**. Indeed, when oxetane **14** was heated at 150 $^{o}$C, led only to product **10** in 80% yield. A similar oxidative transformation was also evidenced when compound **12** was heated under dioxygen at 150 $^{o}$C, providing the isomerized **13** without isolating the respective peroxide **15**, along with **11** and **10**. This interesting unprecedented oxidative isomerization of alkenes will be further commented on below (Scheme 3). Heating the $\beta$-H isomer **11** under deoxygenated conditions at 150 $^{o}$C led to a 3:1 mixture of **11:12**, demonstrating the reversibility of the ene-process. Connecting every piece of information in a postulated mechanism, supports a non-reversible oxy-Cope/reversible ene-reaction sequence towards the thermodynamic production preference for **11**, bearing the $\beta$-H stereoisomer in the guaianolide junction, when dioxygen is absent. A closer look to germacranolide conformers can shed light to this preference as a part of a dipole moment decrease in the system. Oxidative isomerization of **11** leads to the non-reversible production of compound **10** when dioxygen is present. Focusing on the germacranolide conformers **4A,B**, it is expected that an appropriate metal interaction between the carbonyl and the lactone moieties might allow the delay for the rotation of the carbonyl group, enriching the production of $\alpha$-H guaianolide stereoisomer **12**. Indeed, when LiCl was introduced in the oxy-Cope/ene reaction of **5** in deoxygenated toluene at 160 $^{o}$C, a preference for **12** was obtained as a 2:1 mixture.

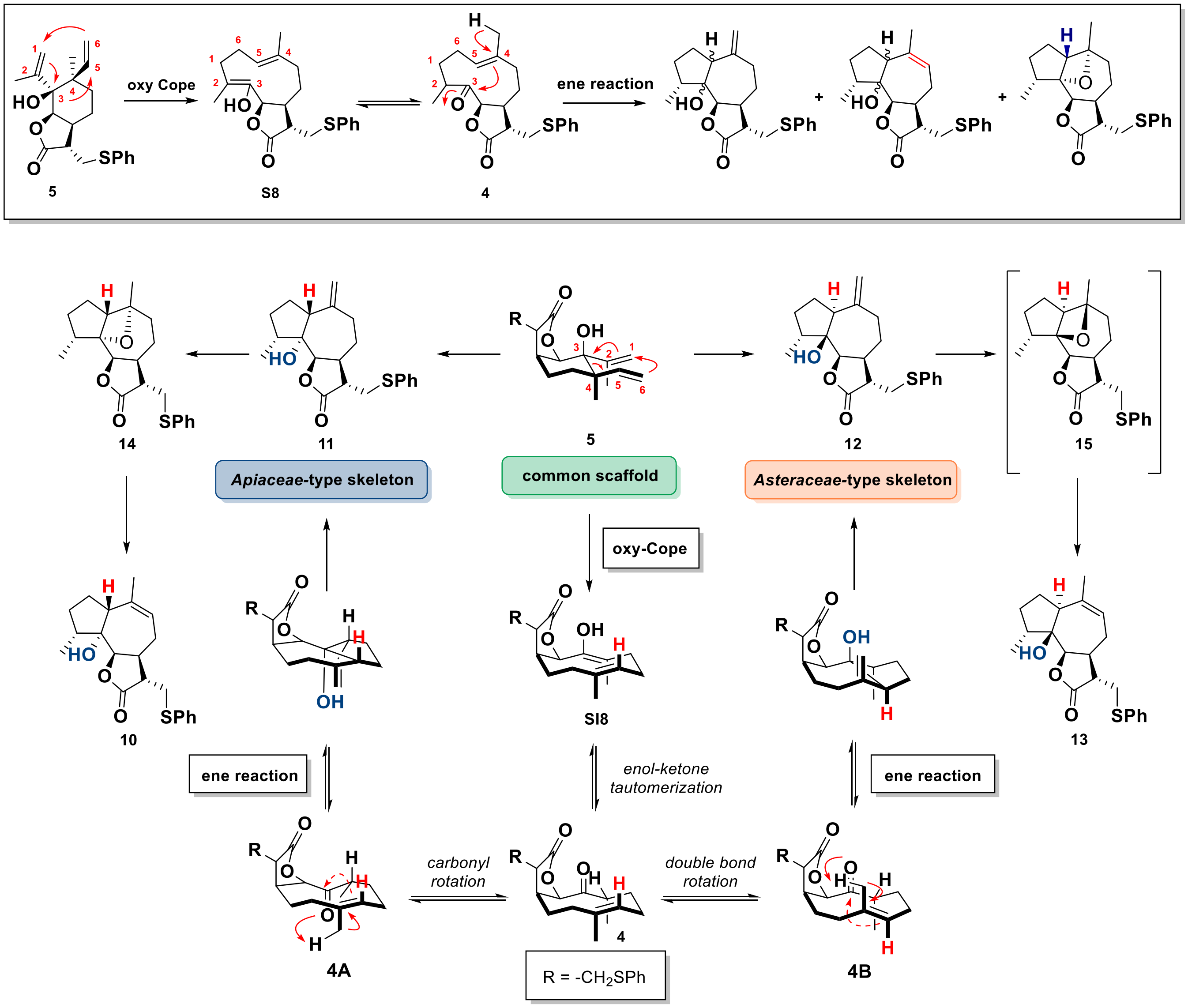


**Scheme SI5**. Proposed mechanism of oxy-Cope/ ene reactions

**(3S,3aS,6aR,9R,9aR,9bR)-9a-hydroxy-9-methyl-6-methylene-3-((phenylthio)methyl)decahydroazuleno[4,5-b]furan-2(3H)-one (11)**

**(3S,3aS,6aS,9R,9aS,9bR)-9a-hydroxy-9-methyl-6-methylene-3-((phenylthio)methyl)decahydroazuleno[4,5-b]furan-2(3H)-one (12)**

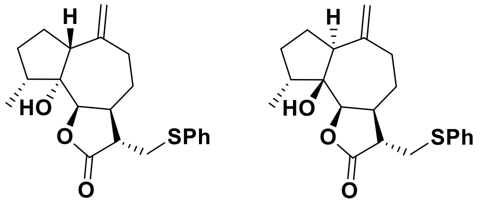


11 12

**MW**: 358.50 g/mol

**Molecular Formula**: $C_{21}H_{26}O_3S$

To a sealed tube, compound **5** (75.0 mg, 0.21 mmol, 1.0 eq) was dissolved in toluene (0,03 M, 7 mL) and the solution was deoxygenated at room temperature by bubbling argon for 10 min. The tube was tightly sealed and the mixture was stirred in oil bath at 160 °C for 12h before allowed to return at room temperature. The solvent was removed *in vacuo* and the residue was purified by column chromatography on silica gel:

**Compound 11**: elution with PS:$Et_2O$ 6:1 (TLC: $R_f$ = 0.48 upon PS:$Et_2O$ 1:1, UV active on TLC, stains blue upon seebach staining) afforded pure compound **11** (40.5 mg, 54%), as light yellow solid. $[\alpha]^{25}_D$= -5.9 (c 0.013 $CHCl_3$); $^1H$ NMR (500 MHz, $CDCl_3$): $\delta_H$ = 7.40 (d, *J* = 7.2 Hz, 2H), 7.32 (t, *J* = 7.6 Hz, 2H), 7.25 (t, J = 7.6 Hz, 1H), 5.05 (s, 1H), 4.91 (s, 1H), 4.61 (d, *J* = 7.5 Hz, 1H), 3.46 (dd, *J* = 13.5, 4.0 Hz, 1H), 3.03 (dd, *J* = 13.6, 10.2 Hz, 1H), 2.90 (dd, *J* = 7.9, 4.5 Hz, 1H), 2.82 (t, *J* = 8.5 Hz, 1H), 2.65 (dt, *J* = 10.2, 4.0 Hz, 1H), 2.43 – 2.34 (m, 1H), 2.27 (dt, *J* = 10.3, 7.0 Hz, 1H), 2.10 – 2.01 (m, 1H), 1.85 – 1.80 (m, 5H), 1.45 – 1.29 (m, 1H), 0.97 (d, *J* = 6.8 Hz, 3H).; $^{13}C$ NMR (125 MHz, $CDCl_3$): $\delta_C$ = 177.0, 148.4, 134.2, 130.3, 129.3, 127.1, 113.0, 80.8, 79.9, 48.0, 46.9, 41.7, 41.1, 35.1, 33.7, 30.5, 29.5, 25.2, 12.6.; HRMS (ESI, m/z): calcd for $C_{21}H_{26}O_3SNa^+$($[M+Na]^+$): 381.1495, found: 381.1493

**Compound 12**: elution with PS:$Et_2O$ 1:1 (TLC: $R_f$ = 0.28 upon PS:$Et_2O$ 1:1, UV active on TLC, stains blue upon seebach staining) afforded pure compound **12** (13.6 mg, 18%), as yellow oil. $[\alpha]^{25}_D$= -10.5 (c 0.013 $CHCl_3$); $^1H$ NMR (500 MHz, $CDCl_3$): $\delta_H$ = 7.38 (m, 2H), 7.29 (m, 2H), 7.20 (t, *J* = 7.4 Hz, 1H), 4.98 (s, 1H), 4.86 (s, 1H), 4.64 (d, *J* = 8.3 Hz, 1H), 3.46 (dd, *J* = 13.5, 3.5 Hz, 1H), 3.07 (dd, *J* = 13.5, 7.0 Hz, 1H), 2.93 – 2.80 (m, 2H), 2.62 – 2.46 (m, 2H), 2.32 (dq, *J* = 13.8, 7.3 Hz, 1H), 2.22 (qt, *J* = 7.1, 3.7 Hz, 1H), 2.13 – 2.02 (m, 1H), 1.96 (ddd, *J* = 10.3, 5.2, 1.9 Hz, 2H), 1.88 (t, *J* = 12.8 Hz, 1H), 1.65 – 1.54 (m, 1H), 1.35 – 1.23 (m, 1H), 1.09 (d, *J* = 7.1 Hz, 3H).; $^{13}C$ NMR (125 MHz, $CDCl_3$): $\delta_C$

= 177.3, 148.2, 135.5, 129.6, 129.1, 126.6, 112.7, 85.8, 83.0, 49.2, 46.8, 46.2, 43.4, 36.0, 34.7, 31.0, 29.2, 25.5, 19.2.; HRMS (ESI, m/z): calcd for $C_{21}H_{26}O_3SK^+$([M+K]$^+$): 397.1234 found: 397.1233

*Alternatively*: To a sealed tube, compound **5** (75.0 mg, 0.21 mmol, 1.0 eq) was dissolved in toluene (0,03 M, 7 mL), LiCl (0.89 mg, 0.021 mmol, 10%) was added and the solution was deoxygenated at room temperature by bubbling argon for 10 min. The tube was tightly sealed and the mixture was stirred in oil bath at 160 °C for 12h before allowed to return at room temperature. The solvent was removed *in vacuo* and the residue was purified by column chromatography on silica gel:

**Compound 11**: elution with PS:$Et_2O$ 6:1 (TLC: $R_f$ = 0.48 upon PS:$Et_2O$ 1:1, UV active on TLC, stains blue upon seebach staining) afforded pure compound **11** (17.3 mg, 23%), as light yellow solid.

**Compound 12**: elution with PS:$Et_2O$ 1:1 (TLC: $R_f$ = 0.28 upon PS:$Et_2O$ 1:1, UV active on TLC, stains blue upon seebach staining) afforded pure compound **12** (35 mg, 46%), as yellow oil.

**(3S,3aS,6aR,9R,9aR,9bR)-9a-hydroxy-6,9-dimethyl-3-((phenylthio)methyl)-3a,4,6a,7,8,9,9a,9boctahydro azuleno[4,5-b]furan-2(3H)-one (10)**

**(3S,3aS,6aS,9R,9aS,9bR)-9a-hydroxy-6,9-dimethyl-3-((phenylthio)methyl)-3a,4,6a,7,8,9,9a,9b-octahydro azuleno[4,5-b]furan-2(3H)-one (13)**

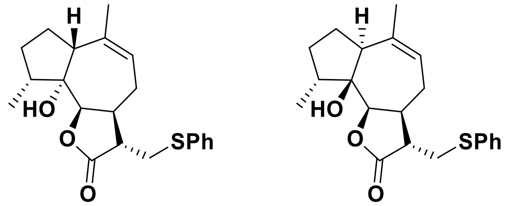


**10** **13**

**MW**: 358.50 g/mol

**Molecular Formula**: $C_{21}H_{26}O_3S$

To a sealed tube, compound **5** (10.0 mg, 0.028 mmol, 1.0 eq) was dissolved in toluene (0,03 M, 0.92 mL) and the solution was bubbled with oxygen. The resulting solution was stirred in oil bath at 160 °C for 12h and then was allowed to return at room temperature. The solvent was removed *in vacuo* and the residue was purified by column chromatography on silica gel:

**Compound 10**: elution with PS:$Et_2O$ 5:1 (TLC: $R_f$ = 0.40 upon PS:$Et_2O$ 1:1, UV active on TLC, stains blue upon seebach staining) afforded pure compound **10** (4.1 mg, 41%), as yellow solid. $[\alpha]^{25}_D$= +50.0 (c 0.001 $CHCl_3$); $^1$H NMR

(500 MHz, $CDCl_3$): $\delta_H$ = 7.37 (d, *J* = 7.7 Hz, 2H), 7.30 (t, *J* = 7.6 Hz, 2H), 7.21 (t, *J* = 7.3 Hz, 1H), 5.39 – 5.34 (m, 1H), 4.49 (d, *J* = 6.9 Hz, 1H), 3.43 (dd, *J* = 13.7, 3.1 Hz, 1H), 2.98 (dd, *J* = 13.7, 7.7 Hz, 1H), 2.85 – 2.76 (m, 2H), 2.64 – 2.54 (m, 2H), 2.42 (dt, *J* = 17.0, 6.8 Hz, 1H), 2.31 – 2.15 (m, 1H), 1.90 (qd, *J* = 9.0, 8.0, 4.3 Hz, 1H), 1.74 (m, 2H), 1.62 (s, 3H), 1.41 – 1.30 (m, 1H), 0.96 (d, *J* = 6.8 Hz, 3H).; $^{13}$C NMR (125 MHz, $CDCl_3$): $\delta_C$ =177.3, 138.0, 134.9, 130.0, 129.1, 126.8, 121.7, 80.7, 79.5, 47.2, 42.0, 40.2, 40.1, 34.0, 27.9, 27.1, 26.3, 23.9, 12.3.; HRMS (ESI, m/z): calcd for $C_{21}H_{26}O_3SK^+$($[M+K]^+$): 397.1234, found: 397.1237

**Compound 13**: elution with PS:$Et_2O$ 2:1 (TLC: $R_f$ = 0.35 upon PS:$Et_2O$ 1:1, UV active on TLC, stains blue upon seebach staining) afforded pure compound **13** (2.0 mg, 20%), as yellow solid. $[\alpha]^{25}_D$= +66.7 (c 0.001 $CHCl_3$); $^1$H NMR (500 MHz, $CDCl_3$): $\delta_H$ = 7.39 (d, *J* = 8.2 Hz, 2H), 7.31 (t, *J* = 7.8 Hz, 2H), 7.25 – 7.19 (m, 1H), 5.48 (d, *J* = 7.5 Hz, 1H), 4.57 (d, *J* = 7.1 Hz, 1H), 3.43 (dd, *J* = 13.5, 3.2 Hz, 1H), 3.04 (dd, *J* = 13.4, 7.9 Hz, 1H), 2.72 (h, *J* = 5.2, 4.5 Hz, 2H), 2.60 (t, *J* = 9.2 Hz, 2H), 2.10 (ddt, *J* = 9.9, 6.8, 3.5 Hz, 3H), 1.96 (dp, *J* = 12.1, 4.2 Hz, 1H), 1.92 – 1.85 (m, 1H), 1.74 (s, 3H), 1.06 (d, *J* = 6.5 Hz, 3H).

$^{13}$C NMR (125 MHz, $CDCl_3$): $\delta_C$ = 177.9, 137.9, 135.1, 130.0, 129.2, 126.8, 121.1, 82.5, 81.2, 47.3, 46.0, 45.3, 41.6, 34.8, 31.3, 27.7, 27.2, 23.9, 18.7.; HRMS (ESI, m/z): calcd for $C_{21}H_{26}O_3SNa^+$($[M+Na]^+$): 381.1495, found: 381.1494

*Alternatively*: To a sealed tube, compound **11** (5.0 mg, 0.014 mmol, 1.0 eq) was dissolved in toluene (0,03 M, 0.46 mL) and the solution was bubbled with oxygen. The resulting solution was stirred in oil bath at 150 °C for 3h and then was allowed to return at room temperature. The solvent was removed *in vacuo* and the residue was purified by column chromatography on silica gel: elution with PS:$Et_2O$ 5:1 (TLC: $R_f$ = 0.40 upon PS:$Et_2O$ 1:1, UV active on TLC, stains blue upon seebach staining) afforded pure compound **10** (4.0 mg, 80%), as yellow solid.

*Alternatively*: To a sealed tube, compound **14** (5.0 mg, 0.014 mmol, 1.0 eq) was dissolved in toluene (0,1 mL), the solution was stirred in oil bath at 150 °C for 4h and then was allowed to cool at room temperature. The solvent was removed *in vacuo* and the residue was purified by column chromatography on silica gel: elution with PS:$Et_2O$ 5:1 (TLC: $R_f$ = 0.40 upon PS:$Et_2O$ 1:1, UV active on TLC, stains blue upon seebach staining) afforded pure compound **10** (4 mg, 80%), as yellow solid.

**(3S,3aS,6R,6aR,9R,9aR,9bR)-6,9-dimethyl-3-((phenylthio)methyl)octahydro-4H-6,9a-epidioxyazuleno[4,5-b]furan-2(3H)-one (14)**

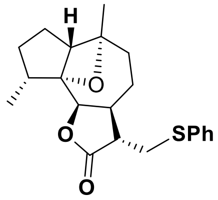

**14**

**MW**: 358.50 g/mol

**Molecular Formula**: $C_{21}H_{26}O_3S$

To a sealed tube, compound **5** (165.0 mg, 0.46 mmol, 1.0 eq) was dissolved in toluene (0,03 M, 15 mL) and the solution was bubbled with oxygen for 10 min. The tube was sealed, the mixture was stirred in oil bath at 160 °C for 12h and then was allowed to return at room temperature. The solvent was removed *in vacuo* and the residue was purified by column chromatography on silica gel: elution with PS:$Et_2O$ 7:1 (TLC: $R_f$ = 0.35 upon PS:$Et_2O$ 1:1, UV active on TLC, stains blue upon Seebach staining) afforded pure compound **14** (30 mg, 18%), as yellow solid. $[\alpha]^{25}_D$= -21.1 (c 0.0067 $CHCl_3$); $^1$H NMR (500 MHz, $CDCl_3$): $\delta_H$ = 7.44 – 7.38 (d, 2H), 7.33 (m, 2H), 7.27 (d, *J* = 5.0 Hz, 1H), 4.67 (d, *J* = 5.7 Hz, 1H), 3.38 (dd, *J* = 13.6, 4.3 Hz, 1H), 3.00 (dd, *J* = 13.5, 11.3 Hz, 1H), 2.94 (dt, *J* = 13.2, 4.7 Hz, 1H), 2.80 (d, *J* = 8.0 Hz, 1H), 2.45 (dd, *J* = 11.3, 4.3 Hz, 1H), 2.04 – 1.96 (m, 1H), 1.93 – 1.78 (m, 4H), 1.69 (qd, *J* = 8.6, 7.8, 4.1 Hz, 3H), 1.59 (m, 1H), 1.18 (s, 3H), 0.91 (d, *J* = 6.5 Hz, 3H); $^{13}$C NMR (125 MHz, $CDCl_3$): $\delta_C$ = 177.4, 134.1, 130.6, 129.3, 127.2, 89.3, 83.8, 77.8, 49.2, 41.6, 40.8, 39.5, 38.8, 34.1, 32.8, 27.2, 24.9, 22.2, 9.8. HRMS (ESI, m/z): calcd for $C_{21}H_{26}O_3SNa^+$($[M+Na]^+$): 381.1495, found: 381.1492

## 4. Final steps towards epoxy-osmitopsin (20)

The final steps on this route include, firstly, the selective reduction of the *exo*- double bond (Δ10,14) and then, the challenging dehydration towards the formation of the desired 4,5-double bond, which proved to undergo isomerization in the presence of silica and chloroform. Use of Burgess reagent as dehydrating agent led to a mixture of compounds **18** and **19** and following treatment of the mixture with *m*-CPBA resulted in the formation of the corresponding epoxides. The final deprotection was achieved after reaction of the epoxides with TBAF, eventually giving rise to 4,5$\alpha$-epoxy-osmitopsin **(20)**.

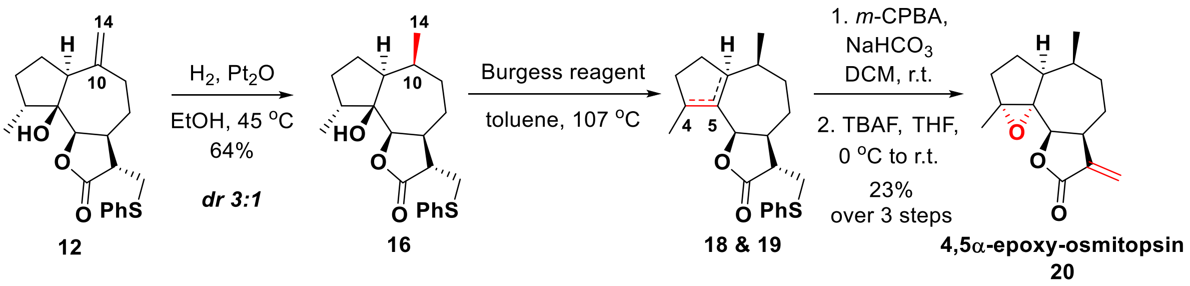


**Scheme SI6**. Final transformations towards 4,5*α*-epoxy-osmitopsin **20**

**(3S,3aS,6S,6aS,9R,9aS,9bR)-9a-hydroxy-6,9-dimethyl-3-((phenylthio)methyl)decahydroazuleno[4,5-b]furan-2(3H)-one (16)**

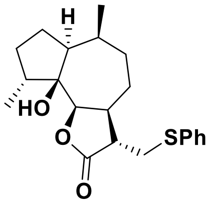


**16**

**MW**: 360.51

**Molecular formula**: $C_{21}H_{28}O_3S$

To a sealed tube, compound **12** (45 mg, 0.13 mmol, 1.0 eq) is dissolved in EtOH (3 mL), $PtO_2$ (2.8 mg, 0.013, 10%) was added and $H_2$ was bubbled through the solution. The tube was sealed and the resulting mixture was stirred in oil bath at 60 °C for 12h. The solution was filtered through a short pad of celite. The solvent was removed *in vacuo* and the residue was purified by column chromatography on silica gel, elution with Hex:$Et_2O$ 4:1 (TLC: $R_f$ = 0.46 upon Hex:$Et_2O$ 1:1.5, UV active on TLC, stains green upon *p*-anisaldehyde staining) afforded pure compound **16** (30 mg, 64%), as white solid. $[\alpha]^{25}_D$= -24.4 (c 0.01, $CHCl_3$); $^1H$ NMR (500 MHz, $CDCl_3$) $\delta_H$ = 7.39 (dt, *J* = 4.9, 2.2 Hz, 2H), 7.29 (t, *J* = 7.6 Hz, 2H), 7.20 (t, *J* = 7.4 Hz, 1H), 4.51 (d, *J* = 9.3 Hz, 1H), 3.43 (dd, *J* = 13.3, 4.3 Hz, 1H), 3.13 (dd, *J* = 13.2, 7.1 Hz, 1H), 2.95 (ddd, *J* = 11.0, 7.0, 4.2 Hz, 1H), 2.85 – 2.74 (m, 1H), 2.09 – 2.00 (m, 3H), 1.98 – 1.92 (m, 2H), 1.91 – 1.72 (m, 3H), 1.78 (m, 2H), 1.43 (ddd, *J* = 14.6, 11.6, 3.8 Hz, 1H), 1.29 (m, 1H), 1.08 (d, *J* = 7.3 Hz, 3H), 1.01 (d, *J* = 7.1 Hz, 3H); $^{13}C$ NMR (125 MHz, $CDCl_3$) $\delta_C$ = 178.0, 135.9, 129.6, 129.1, 126.5, 88.7, 83.5, 48.2, 46.7, 45.1, 43.5, 34.6, 32.4, 31.9, 29.7, 26.2, 23.7, 18.9, 14.8.; HRMS (ESI, m/z): calcd for $C_{21}H_{29}O_3S^+$([M+H]$^+$): 361.1832, found: 361.1832

At this point, our attempts towards the dehydration yielding the desired Δ4,5 double bond proved to be challenging. As shown in **Table 3**, a series of reagents were used, with TFA, *p*-TSA and $BF_3$ $OEt_2$ giving only starting material. Use of Martin's sulfurane in the presence of $Et_3N$ gave rise to deprotected compound **17**, which after treatment with phosgene and DMAP lead to retrieval of starting material. Better results were observed with Burgess reagent, when we were able to obtain a mixture of compounds **18** and **19** (1:2 ratio). However, due to sensitivity of compound **18** to chromatographic purification and $CDCl_3$ the mixture was directly treated with *m*-CPBA, in the next step.

**Table 3. Dehydration attempts of compound 16**

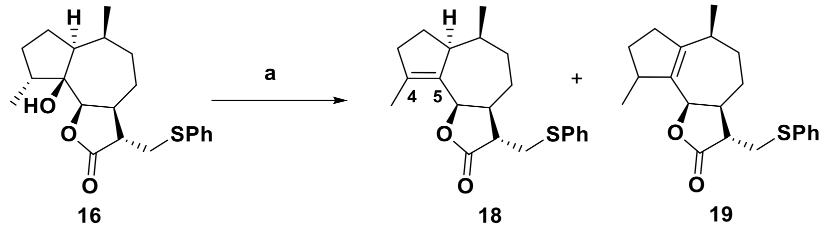


| a/a | Starting material | Reagent | Solvent/ Temperature | Product (Yield) |
|---|---|---|---|---|
| **1** | **16** | TFA | DCM, 50 °C | Starting material |
| **2** | **16** | *p*-TSA | Toluene, 45 °C | Starting material |
| **3** | **16** | $BF_3 \cdot OEt_2$ | DCM, 40 °C | Starting material |
| **4** | **16** | Martin's sulfurane & $Et_3N$ | toluene, 80 °C | **17** (45%) |
| **5** | **17** | Phosgene & DMAP | DCM, 60 °C | Starting material |
| **6** | **16** | Burgess reagent | toluene, 107 °C | **18, 19**/1:2 (90%) |

**(3S,3aS,6S,9R,9bR)-6,9-dimethyl-3-((phenylthio)methyl)-3a,4,5,6,7,8,9,9b-octahydroazuleno[4,5-b]furan-2(3H)-one (19)**

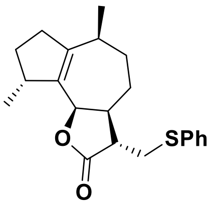

**19**

**MW**: 342.50

**Molecular formula**: $C_{21}H_{26}O_2S$

To a sealed tube compound **16** (15 mg, 0.042 mmol, 1.0 eq) is dissolved in toluene (5 mL) and Burgess reagent (29.75 mg, 0.125 mmol, 3.0 eq) was added. The resulting mixture was stirred in oil bath overnight at 107°C and then was allowed to cool at room temperature. The solvent was removed *in vacuo*. Examination of the NMR spectrum showed a mixture of compounds **18** and **19** which, after purification by column chromatography on silica gel, elution with PS:$Et_2O$ 5:1 (TLC: $R_f$ = 0.48 upon PS:$Et_2O$ 1:1, UV active on TLC, stains blue upon *p*-anisaldehyde staining) afforded only compound **19** (8 mg, 62%), as white solid. $[\alpha]^{25}_D$= -13.9 (c 0.001, $CHCl_3$); $^1$H NMR (500 MHz, $CDCl_3$) $\delta_H$ = 7.40 (d, *J* = 7.8 Hz, 2H), 7.34 – 7.30 (m, 2H), 7.25 – 7.23 (m, 1H), 5.23 (d, *J* = 7.7 Hz, 1H), 3.42 (dd, *J* = 13.4, 4.2 Hz, 1H), 3.02 (dd, *J* = 13.6, 10.2 Hz, 1H), 2.91 – 2.83 (m, 1H), 2.69 (ddt, *J* = 10.3, 6.8, 3.9 Hz, 1H), 2.52 (dt, *J* = 10.5, 4.2 Hz, 1H), 2.46 – 2.36 (m, 2H), 2.36 – 2.13 (m, 1H), 2.06 – 1.95 (m, 1H), 1.72 – 1.68 (m, 2H), 1.63 – 1.32 (m, 3H), 1.03 (t, *J* = 7.1 Hz, 6H).

$^{13}$C NMR (125 MHz, Benzene-d6) $\delta_C$ = 177.3, 143.7, 134.5, 133.4, 130.3, 129.2, 127.0, 79.3, 48.3, 42.9, 42.8, 34.7, 34.5, 33.5, 31.1, 30.8, 27.3, 19.7, 19.4..; HRMS (ESI, m/z): calcd for $C_{21}H_{27}O_2S^+$([M+H]$^+$): 343.1726, found: 343.1729

**(2aS,3S,5aS,8aR,8bR,9aR)-3,9a-dimethyl-6-methyleneoctahydro-2H-oxireno[2',3':3,3a]azuleno[4,5-b]furan-7(6H)-one (20)**

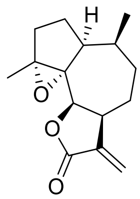

**20**

**MW**: 248.32

**Molecular formula**: $C_{15}H_{20}O_3$

To a sealed tube compound **16** (60 mg, 0.17 mmol, 1.0 eq) is dissolved in toluene (5 mL) and Burgess reagent (118.98 mg, 0.50 mmol, 3.0 eq) was added. The resulting mixture was stirred in oil bath overnight at 107 °C and then was allowed to cool at room temperature. The solvent was removed *in vacuo*.

To a round-bottom flask, the mixture of compounds **18** and **19** (35 mg, 0.10 mmol, 1.0 eq) was dissolved in DCM (3 ml) and $NaHCO_3$ and *m*-CPBA (70%) (49.3 mg, 0.20 mmol, 2.0 eq) were added subsequently. The resulting mixture was stirred for 30 minutes at room temperature. The reaction was quenched with $Na_2S_2O_3$ (3 mL) and the aqueous layer was extracted three times with DCM (3x5 mL). The combined organic extracts were dried over $Na_2SO_4$, filtered and the solvent was removed *in vacuo*.

To a round-bottom flask compound the resulting mixture of epoxides (16.2 mg, 0.045 mmol, 1.0 eq) was dissolved in dry THF (5 mL) and TBAF (0.18 mL, 0.18 mmol, 4.0 eq) was added at 0 °C. The resulting mixture was stirred at room temperature for 2h under Argon atmosphere and was allowed to return at room temperature. The reaction was quenched with water and the aqueous layer was extracted three times with EtOAc (3x5 mL). The combined organic extracts were dried over $Na_2SO_4$, filtered and the solvent was removed *in vacuo*. The residue was purified by column chromatography on silica gel, elution with Benzene:$Et_2O$ (13:1) (TLC: $R_f$ = 0.45 upon Benzene:$Et_2O$ 2:1, UV active on TLC, stains blue upon anisaldehyde staining) afforded compound **20** (10.1 mg), as colorless oil. $[\alpha]^{25}_D$= -15.3 (c 0.002 $CHCl_3$); $^1$H NMR (500 MHz, $CDCl_3$): $\delta_H$ = 6.33 (d, *J* = 3.4 Hz, 1H), 5.62 (d, J = 2.8 Hz, 1H), 5.20 (d, *J* = 8.8 Hz, 1H), 3.34 – 3.23 (m, 1H), 2.37 (dt, *J* = 10.0, 5.0 Hz, 1H), 1.99 (ddd, *J* = 13.4, 9.2, 4.4 Hz, 1H), 1.89 (dd, *J* = 11.1, 6.0 Hz, 2H), 1.77 – 1.70 (m, 1H), 1.66 – 1.54 (m, 4H), 1.45 (p, *J* = 7.6, 6.7 Hz, 1H), 1.39 (s, 3H), 0.91 (d, *J* = 6.9 Hz, 3H). $^{13}$C NMR (125 MHz, $CDCl_3$): $\delta_C$ = 169.5, 138.6, 123.2, 76.40, 72.1, 71.3, 47.6, 38.7, 33.1, 30.9, 29.7, 29.1, 20.9, 20.1, 16.1.; HRMS (ESI, m/z): calcd for $C_{15}H_{20}O_3Na^+$($[M+Na]^+$): 271.1305, found: 271.1308

**(3aS,6S,6aS,9aS,9bR)-9a-hydroxy-6,9-dimethyl-3-methylenedecahydroazuleno[4,5-b]furan-2(3H)-one (17)**

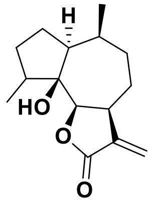

**17**

**MW**: 250.34

**Molecular formula**: $C_{15}H_{22}O_3$

To a round-bottom flask compound **16** (40.0 mg, 0.11 mmol, 1.0 eq) was dissolved in dry THF (5 mL) and TBAF (0.44 mL, 0.44 mmol, 1.0 eq) was added at 0 °C. The resulting mixture was stirred at room temperature for 2h under Argon atmosphere and was allowed to return at room temperature. The reaction was quenched with water and the aqueous layer was extracted three times with EtOAc (3x3 mL). The combined organic extracts were dried over $Na_2SO_4$, filtered and the solvent was removed *in vacuo*. The residue was purified by column chromatography on silica gel, elution with Hex:$Et_2O$ (3:1) (TLC: $R_f$ = 0.42 upon Hex:$Et_2O$ 1:1, UV active on TLC, stains blue upon anisaldehyde staining) afforded compound **17** (25 mg, 91%), as white solid. $[\alpha]^{25}_{D}$= -27.4 (c 0.01 $CHCl_3$); $^1$H NMR (500 MHz, $CDCl_3$) $\delta_H$ = 6.19 (d, *J* = 3.5 Hz, 1H), 5.49 (d, *J* = 3.1 Hz, 1H), 4.64 (d, *J* = 9.2 Hz, 1H), 3.26 (ddp, *J* = 12.2, 9.0, 3.3 Hz, 1H), 2.29 – 2.17 (m, 1H), 2.14 – 2.00 (m, 3H), 1.92 – 1.77 (m, 4H), 1.54 (td, *J* = 12.5, 4.1 Hz, 1H), 1.29 (ddd, *J* = 7.2, 5.2, 1.8 Hz, 1H), 1.10 (d, *J* = 7.2 Hz, 3H), 1.02 (d, *J* = 6.9 Hz, 3H).

$^{13}$C NMR (125 MHz, Benzene-d6) $\delta_C$ = 169.9, 141.4, 118.3, 87.3, 82.2, 47.7, 44.7, 42.0, 33.0, 32.2, 29.9, 26.7, 23.7, 18.1, 14.7.; HRMS (ESI, m/z): calcd for $C_{15}H_{23}O_3^+$([M+H]$^+$): 251.1642, found: 251.1641

**(3aS,6S,6aS,9aS,9bR)-6,9-dimethyl-3-methylene-3a,4,5,6,6a,7,9a,9b-octahydroazuleno[4,5-b]furan-2(3H)-one (22)**

**(3aS,9R,9aS,9bR)-6,9-dimethyl-3-methylene-3a,4,5,7,8,9,9a,9b-octahydroazuleno[4,5-b]furan-2(3H)-one (21)**

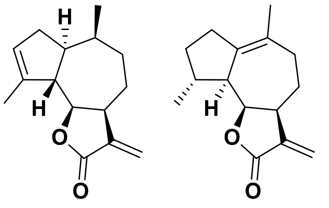

**22** **21**

**MW**: 232.32

**Molecular formula**: $C_{15}H_{20}O_2$

To a sealed tube compound **17** (13 mg, 0.055 mmol, 1.0 eq) was dissolved in dry DCE (1 mL) and $BF_3Et_2O$ (20 µL, 0.17 mmol, 3.0 eq) was added at 0 °C. The resulting mixture was stirred at room temperature for 4h. Although the starting material was not fully consumed, as evidenced by TLC on Benzene:$Et_2O$ 10:1 the reaction was quenched with $NaHCO_3$ and the aqueous layer was extracted three times with DCM (3x3 mL). The combined organic extracts were dried over $Na_2SO_4$, filtered and the solvent was removed *in vacuo*. The residue was purified by column chromatography on silica gel,

**Compound 22**: elution with Hex:EtOAc (36:1) (TLC: $R_f$ = 0.50 upon Hex:EtOAc 3:1, UV active on TLC, stains blue upon anisaldehyde staining) afforded compound **22** (4 mg, 31%), as white solid. $[\alpha]^{25}_{D}$= -11.8 (c 0.001 $CHCl_3$); $^1H$ NMR (500 MHz, $CDCl_3$) $\delta_H$ = 6.24 (d, *J* = 2.0 Hz, 1H), 5.62 (d, *J* = 1.8 Hz, 1H), 5.34 (s, 1H), 4.32 (dd, *J* = 10.2, 6.9 Hz, 1H), 3.06 (ddd, *J* = 11.9, 6.9, 2.2 Hz, 1H), 2.78 (t, *J* = 10.2 Hz, 1H), 2.32 – 2.27 (m, 1H), 2.16 – 2.09 (m, 2H), 2.08 – 2.02 (m, 1H), 1.98 – 1.90 (m, 1H), 1.82 (s, 3H), 1.75 (tt, *J* = 6.6, 2.1 Hz, 1H), 1.71 – 1.65 (m, 1H), 1.39 (dd, *J* = 14.9, 6.4 Hz, 1H), 0.90 (d, *J* = 7.0 Hz, 3H); $^{13}C$ NMR (125 MHz, $CDCl_3$) $\delta_C$ = 170.7, 141.6, 128.3, 125.7, 122.1, 84.3, 48.4, 45.7, 44.5, 34.3, 32.5, 30.1, 29.7, 15.3, 14.1.; HRMS (ESI, m/z): calcd for $C_{15}H_{21}O_2^+$([M+H]$^+$): 233.1536, found: 233.1537

**Compound 21**: elution with Hex:EtOAc (34:1) (TLC: $R_f$ = 0.45 upon Hex:EtOAc 3:1, UV active on TLC, stains blue upon anisaldehyde staining) afforded compound **21** (6 mg, 47%), as white solid. $[\alpha]^{25}_{D}$= -16.6 (c 0.001 $CHCl_3$); $^1H$ NMR (500 MHz, $CDCl_3$) $\delta_H$ 6.34 (d, *J* = 2.6 Hz, 1H), 5.55 (d, *J* = 2.2 Hz, 1H), 4.81 (d, *J* = 8.2 Hz, 1H), 3.37 – 3.30 (m, 1H), 2.53 (d, *J* = 10.1 Hz, 1H), 2.38 – 2.25 (m, 2H), 2.22 – 2.04 (m, 4H), 1.86 (dt, *J* = 12.6, 6.6 Hz, 1H), 1.75 (dq, *J* = 14.3, 4.4 Hz, 1H), 1.49 (s, 3H), 1.13 – 1.09 (m, 1H), 1.05 (d, *J* = 6.5 Hz, 3H).; $^{13}C$ NMR (125 MHz, $CDCl_3$) $\delta_C$ = 170.7, 138.7, 133.2, 126.0, 122.0, 81.7, 50.1, 40.2, 38.3, 33.5, 32.3, 32.0, 28.6, 21.3, 18.5.; HRMS (ESI, m/z): calcd for $C_{15}H_{20}O_2K^+$([M+K]$^+$): 271.1095, found: 271.1093

## 5. Final steps towards 6-*epi*-cichopumilide (3) & neoartabsin (27)

Towards the functionalization of *Apiaceae* carbocyclic cores **10** and **11** we used Martin's sulfurane, as dehydrating agent, in order to introduce the desired double bond at the requisite positions 4,5 and 1,5-. Then, Mukaiyama hydration followed to achieve the stereoselective introduction of a hydroxyl group on C10 which after deprotection of the thiophenyl group and reduction provided 6-*epi*-cichopumilide (**3**) and neoartabsin (**27**) respectively.

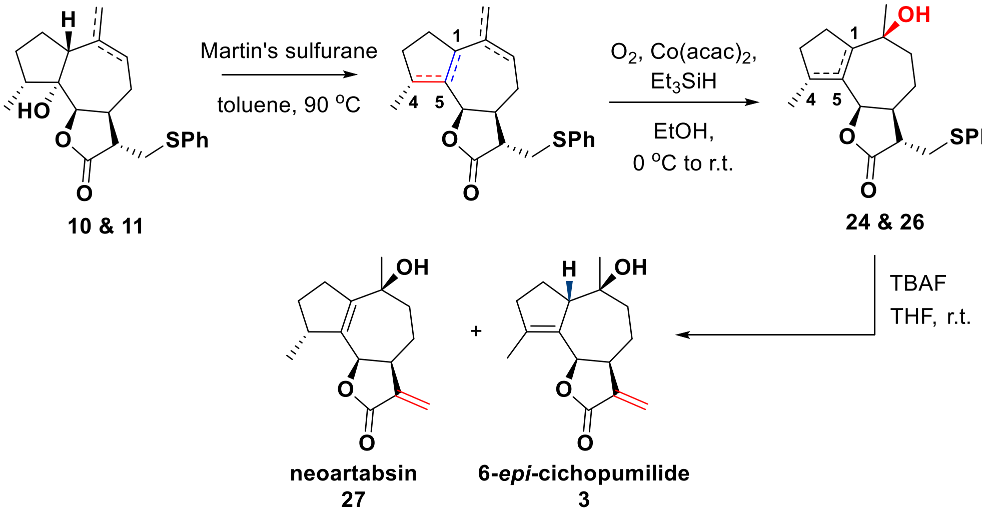


**Scheme SI7**. Final transformations towards 6-*epi*-cichopumilide **3** & neoartabsin **27**

**(3S,3aS,6aR,9bR)-6,9-dimethyl-3-((phenylthio)methyl)-3a,4,6a,7,8,9b-hexahydroazuleno[4,5-b]furan-2(3H)-one (23)**

**(3S,3aS,9R,9bR)-6,9-dimethyl-3-((phenylthio)methyl)-3a,4,7,8,9,9b-hexahydroazuleno[4,5-b]furan-2(3H)-one (25)**

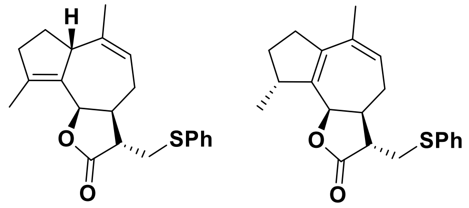


**23** **25**

**Molecular weight**: 340.48

**Molecular formula**: $C_{21}H_{24}O_2S$

To a sealed tube compound **10** (40.0 mg, 0.12 mmol, 1.0 eq) was dissolved in toluene and Martin's sulfurane (118.5 mg, 0.17 mmol, 1.5 eq) was added. The resulting mixture was stirred in oil bath for 2.5h at 90 °C and then was allowed to cool at room temperature. The solvent was removed *in vacuo* and the residue was purified by column chromatography on silica gel:

**Compound 23**: elution with PS:$Et_2O$ 10:1 (TLC: $R_f$ = 0.32 upon PS:$Et_2O$ 2:1, UV active on TLC, stains blue upon seebach staining) afforded pure compound **23** (16 mg, 39%), as white solid. $[\alpha]^{25}_D$= +60.6 (c 0.003, $CHCl_3$); $^1$H NMR (500 MHz, $CDCl_3$) δ = 7.39 (d, *J* = 7.4 Hz, 2H), 7.31 (t, *J* = 7.7 Hz, 2H), 7.23 (t, *J* = 7.3 Hz, 1H), 5.55 (d, *J* = 8.9 Hz, 1H), 5.03 (dd, *J* = 8.6, 4.6 Hz, 1H), 3.52 (dd, *J* = 13.3, 3.8 Hz, 1H), 3.30 (m, 1H), 3.01 (dd, *J* = 13.3, 10.2 Hz, 1H), 2.87 (m, 1H), 2.77 (m, 1H), 2.50 (dd, *J* = 15.0, 1.7 Hz, 1H), 2.33 (m, 1H), 2.24 (m, 2H), 1.82 (m, 1H), 1.71 (s, 3H), 1.58 (s, 3H), 1.55 (m, 1H); $^{13}$C NMR (125 MHz, $CDCl_3$) $\delta_C$ = 178.2, 143.2, 139.7, 133.1, 131.9, 129.9, 129.2, 126.8, 117.7, 78.8, 51.8, 43.5, 42.9, 36.8, 35.9, 29.9, 27.0, 23.6, 14.1.; HRMS (ESI, m/z): calcd for $C_{21}H_{24}O_2SNa^+$([M+Na]$^+$): 363.1389, found: 363.1391

**Compound 25**: elution with PS:$Et_2O$ 14:1 (TLC: $R_f$ = 0.57 upon PS:$Et_2O$ 2:1, UV active on TLC, stains blue upon seebach staining) afforded pure compound **25** (17 mg, 42%), as white solid. $[\alpha]^{25}_D$= +46.1 (c 0.005, $CHCl_3$); $^1$H NMR (500 MHz, $CDCl_3$) $\delta_H$ = 7.39 (d, *J* = 7.8 Hz, 2H), 7.31 (t, *J* = 7.6 Hz, 2H), 7.23 (t, *J* = 7.6 Hz, 1H), 5.64 (t, J = 6.85 Hz, 1H), 5.12 (d, J = 8.84 Hz 1H), 3.47 (m, 1H), 2.98 (m, 2H), 2.82 (m, 1H), 2.73 (m, 1H), 2.54 (m, 1H), 2.37 (m, 1H), 2.16 (m, 2H), 2.02 (m, 1H), 1.73 (s, 3H), 1.44 (m, 1H), 1.11 (s, 3H); $^{13}$C NMR (125 MHz, $CDCl_3$) $\delta_C$ = 177.9, 140.7, 138.5, 135.3, 134.7, 129.9, 129.2, 126.8, 125.5, 78.7, 48.0, 45.1, 42.3, 34.5, 34.3, 30.4, 29.3, 21.7, 19.2.; HRMS (ESI, m/z): calcd for $C_{21}H_{24}O_2SNa^+$([M+Na]$^+$): 363.1389, found: 363.1389

**(3S,3aS,6S,6aS,9bR)-6-hydroxy-6,9-dimethyl-3-((phenylthio)methyl)-3a,4,5,6,6a,7,8,9b-octahydroazuleno[4,5-b]furan-2(3H)-one (24)**[6]

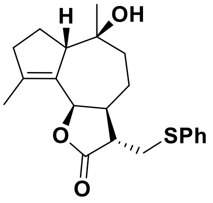


**24**

**MW**: 358.50

**Molecular formula**: $C_{21}H_{26}O_3S$

To a microwave 4 ml vial, compound **23** (20 mg, 0.058 mmol, 1.0 eq) was dissolved in EtOH (0.6 mL), $Co(acac)_2$ (2.27 mg, 0.009 mmol, 0.3 eq) was added and $O_2$ was bubbled through the solution while $PhSiH_3$ (18 µL, 0.14 mmol, 2.5 eq in 1.2 mL EtOH) was added via syringe pump over 1h at 0 °C. The resulting mixture was stirred for 2h at room temperature. The reaction was quenched with $Na_2S_2O_3$ (aq.sat., 0.3 mL), $NaHCO_3$ (aq.sat., 0.2 mL) and EtOAc (aq.sat., 0.3 mL) and was stirred for 30 minutes at room temperature. The aqueous layer was extracted

three times with EtOAc (3x2 mL). The combined organic extracts were dried over $Na_2SO_4$, filtered and the solvent was removed *in vacuo*. The residue was purified by column chromatography on silica gel, elution with Tol:$Et_2O$ 3:1 (TLC: $R_f$ = 0.46 upon Tol:$Et_2O$ 1:1, UV active on TLC, stains blue upon *p*-anisaldehyde staining) afforded compound **24** (8 mg, 38%), as green oil. $[\alpha]^{25}_{D}$= -36.9 (c 0.003, $CHCl_3$) $^1H$ NMR (500 MHz, $CDCl_3$) $\delta_H$ = 7.39 (d, *J* = 7.7 Hz, 2H), 7.31 (t, *J* = 7.5 Hz, 2H), 7.23 (d, *J* = 7.3 Hz, 1H), 5.44 (d, *J* = 8.8 Hz, 1H), 3.54 (dd, *J* = 13.4, 3.8 Hz, 1H), 3.06 (dd, *J* = 13.4, 9.0 Hz, 1H), 2.94 (td, *J* = 8.4, 3.8 Hz, 1H), 2.85 (dt, *J* = 8.5, 4.4 Hz, 1H), 2.73 (d, *J* = 9.1 Hz, 1H), 2.41 (dd, *J* = 17.4, 8.8 Hz, 1H), 2.18 (dd, *J* = 16.8, 10.1 Hz, 1H), 2.03 (dd, *J* = 13.5, 7.8 Hz, 1H), 1.90 (dt, *J* = 13.7, 9.8 Hz, 1H), 1.77 (s, 3H), 1.75 (m, 1H), 1.61 – 1.52 (m, 3H), 1.07 (s, 3H); $^{13}C$ NMR (125 MHz, $CDCl_3$) $\delta_C$ = 177.4, 146.5, 134.9, 130.5, 129.9, 129.2, 126.9, 77.8, 76.0, 55.8, 43.3, 42.0, 39.6, 37.8, 35.3, 25.3, 24.1, 21.1, 14.4.; HRMS (ESI, m/z): calcd for $C_{21}H_{26}O_3SK^+$($[M+K]^+$): 397.1234, found: 397.1237

**(3S,3aS,6S,9R,9bR)-6-hydroxy-6,9-dimethyl-3-((phenylthio)methyl)-3a,4,5,6,7,8,9,9b-octahydroazuleno[4,5-b]furan-2(3H)-one (26)**[6]

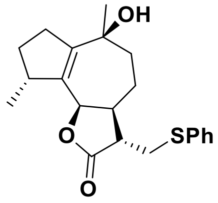


**26**

**MW**: 358.50

**Molecular formula**: $C_{21}H_{26}O_3S$

To a microwave vial, compound 25 (60 mg, 0.18 mmol, 1.0 eq) was dissolved in EtOH (1.8 mL), $Co(acac)_2$ (13.6 mg, 0.053 mmol, 0.3 eq) was added and $O_2$ was bubbled through the solution while $PhSiH_3$ 54 µL, 0.44 mmol, 2.5 eq) was added via syringe pump over 1h at 0 °C. The resulting mixture was stirred for 1h at room temperature °C. The reaction was quenched with $Na_2S_2O_3$ (aq.sat., 0.9 mL), $NaHCO_3$ (aq.sat., 0.6 mL) and EtOAc (aq.sat., 0.9 mL) and was stirred for 30 minutes at room temperature. The aqueous layer was extracted three times with EtOAc (3x3 mL). The combined organic extracts were dried over $Na_2SO_4$, filtered and the solvent was removed *in vacuo*. The residue was purified by column chromatography on silica gel, elution with Tol:$Et_2O$ 5:1 (TLC: $R_f$ = 0.52 upon Tol:$Et_2O$ 1:1, UV active on TLC, stains blue upon *p*-anisaldehyde staining) afforded compound **26** (25 mg, 39%), as green oil. $[\alpha]^{25}_{D}$= -53.7 (c 0.001, $CHCl_3$) $^1H$ NMR (500 MHz, $CDCl_3$) $\delta_H$ = 7.38 (d, *J* = 8.1 Hz, 2H), 7.30 (t, *J* = 7.5 Hz, 2H), 7.21 (t, *J* = 7.3 Hz, 1H), 5.27 (d, *J* = 8.2 Hz, 1H), 3.46 (dd, *J* = 13.6, 4.1 Hz, 1H), 3.08 (dd, *J* = 13.6, 7.8 Hz, 1H), 2.85 (p, *J* =

10.4, 9.7 Hz, 2H), 2.60 (ddd, *J* = 11.6, 7.8, 4.2 Hz, 1H), 2.56 – 2.47 (m, 2H), 1.98 (ddd, *J* = 14.9, 9.2, 6.4 Hz, 1H), 1.93 – 1.81 (m, 2H), 1.77 – 1.63 (m, 2H), 1.49 – 1.40 (m, 1H), 1.29 (s, 3H), 1.04 (d, *J* = 6.8 Hz, 3H).; $^{13}C$ NMR (125 MHz, $CDCl_3$) $\delta_C$ = 177.0, 140.3, 135.9, , 129.6, 129.2, 128.3, 126.6, 77.8, 73.8, 45.4, 45.3, 42.9, 38.0, 33.6, 31.6, 29.7, 29.0, 23.6, 19.0.; HRMS (ESI, m/z): calcd for $C_{21}H_{27}O_3S^+$([M+H]$^+$): 359.1675, found: 359.1678

**(3aS,6S,6aS,9bR)-6-hydroxy-6,9-dimethyl-3-methylene-3a,4,5,6,6a,7,8,9b-octahydroazuleno[4,5-b]furan-2(3H)-one (3)**

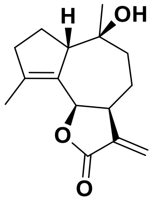

**3**

**MW**: 248.32

**Molecular formula**: $C_{15}H_{20}O_3$

To a round-bottom flask compound **24** (7mg, 0.02 mmol, 1.0 eq) was dissolved in dry THF (1 mL) and TBAF (0.078 mL, 0.078 mmol, 1.0 eq) was added at 0 °C. The resulting mixture was stirred at 0 °C for 30 minutes under Argon atmosphere and was allowed to return at room temperature. The reaction was quenched with water and the aqueous layer was extracted three times with EtOAc (3x3 mL). The combined organic extracts were dried over $Na_2SO_4$, filtered and the solvent was removed *in vacuo*. The residue was purified by column chromatography on silica gel, elution with Hex:$Et_2O$ (1:1) (TLC: $R_f$ = 0.30 upon Hex:$Et_2O$ 1:1, UV active on TLC, stains blue upon anisaldehyde staining) afforded compound **3** (4 mg, 80%), as white solid. $[\alpha]^{25}_D$= -46.2 (c 0.002, $CHCl_3$); $^1H$ NMR (500 MHz, $CDCl_3$) $\delta_H$ = 6.43 (d, *J* = 3.1 Hz, 1H), 5.56 (d, *J* = 2.7 Hz, 1H), 5.51 (d, *J* = 9.3 Hz, 1H), 3.42 (dd, *J* = 6.2, 3.6 Hz, 1H), 2.70 (d, *J* = 9.2 Hz, 1H), 2.45 (dq, *J* = 17.4, 9.1, 8.3 Hz, 1H), 2.24 – 2.16 (m, 1H), 2.05 (q, *J* = 7.7 Hz, 1H), 1.96 – 1.83 (m, 2H), 1.79 (s, 3H), 1.74 – 1.67 (m, 2H), 1.54 – 1.51 (m, 1H), 1.12 (s, 3H); $^{13}C$ NMR (125 MHz, $CDCl_3$) $\delta_C$ = 170.5, 146.0, 138.6, 130.6, 122.8, 77.2, 75.8, 55.5, 40.7, 39.0, 38.0, 25.6, 25.6, 20.9, 14.4. HRMS (ESI, m/z): calcd for $C_{15}H_{21}O_3^+$([M+H]$^+$): 249.1485, found: 249.1483

**(3aS,6S,9R,9bR)-6-hydroxy-6,9-dimethyl-3-methylene-3a,4,5,6,7,8,9,9b-octahydroazuleno[4,5-b]furan-2(3H)-one (27)**

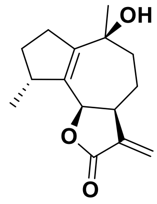

**27**

**MW**: 248.32

**Molecular formula**: $C_{15}H_{20}O_3$

To a round-bottom flask compound **26** (10 mg, 0.028 mmol, 1.0 eq) was dissolved in dry THF (1 mL) and TBAF (0.11 mL, 0.11 mmol, 4.0 eq) was added at 0 °C. The resulting mixture was stirred at 0 °C for 30 minutes under Argon atmosphere and was allowed to return at room temperature. The reaction was quenched with water and the aqueous layer was extracted three times with EtOAc (3x3 mL). The combined organic extracts were dried over $Na_2SO_4$, filtered and the solvent was removed *in vacuo*. The residue was purified by column chromatography on silica gel, elution with Hex:$Et_2O$ (1:1) (TLC: $R_f$ = 0.32 upon Hex:$Et_2O$ 1:1, UV active on TLC, stains blue upon anisaldehyde staining) afforded compound **27** (5 mg, 72%), as white solid. $[\alpha]^{25}_D$= -23.5 (c 0.002 $CHCl_3$); $^1$H NMR (500 MHz, $CDCl_3$) $\delta_H$ = 6.26 (d, *J* = 2.2 Hz, 1H), 5.62 (d, *J* = 1.9 Hz, 1H), 5.20 (d, *J* = 7.3 Hz, 1H), 3.40 (td, *J* = 8.6, 7.5, 2.9 Hz, 1H), 3.00 (d, *J* = 7.1 Hz, 1H), 2.71 – 2.61 (m, 1H), 2.44 – 2.31 (m, 1H), 2.08 – 1.99 (m, 2H), 1.83 (tdd, *J* = 13.8, 7.6, 4.5 Hz, 2H), 1.73 – 1.64 (m, 1H), 1.41 (ddt, *J* = 13.7, 8.9, 5.3 Hz, 1H), 1.33 (s, 3H), 1.09 (d, *J* = 6.8 Hz, 3H).; $^{13}$C NMR (125 MHz, $CDCl_3$) $\delta_C$ = 170.3, 143.7, 141.1, 136.1, 122.3, 77.7, 72.9, 43.2, 42.4, 38.5, 32.5, 30.3, 28.9, 28.3, 19.4. HRS (ESI, m/z): calcd for $C_{15}H_{20}O_3Na^+$($[M+Na]^+$): 271.1305, found: 271.1307

## 5. Spectra

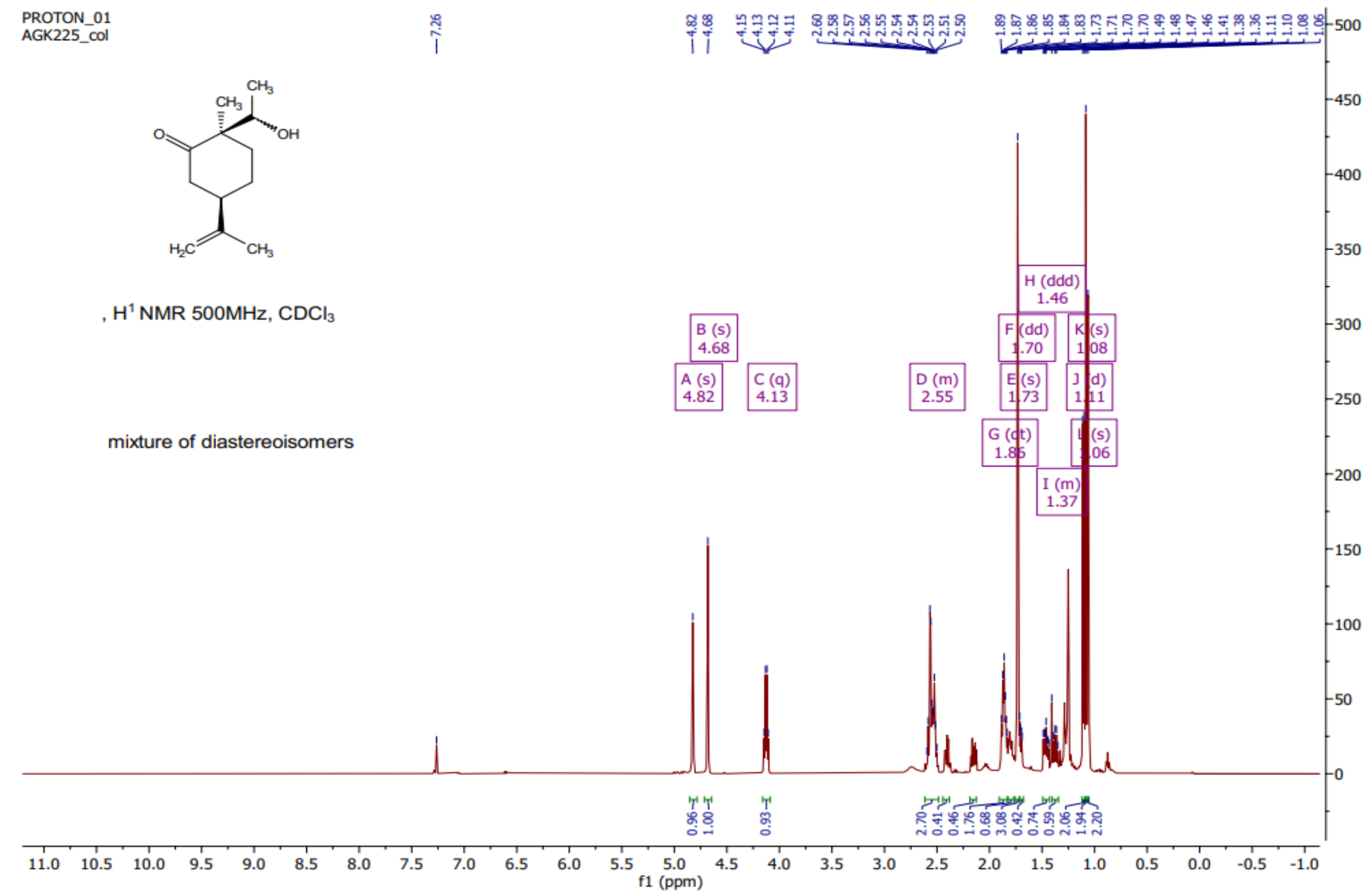
PROTON_01
AGK225_col
, H1 NMR 500MHz, CDCl3
mixture of diastereoisomers
f1 (ppm)

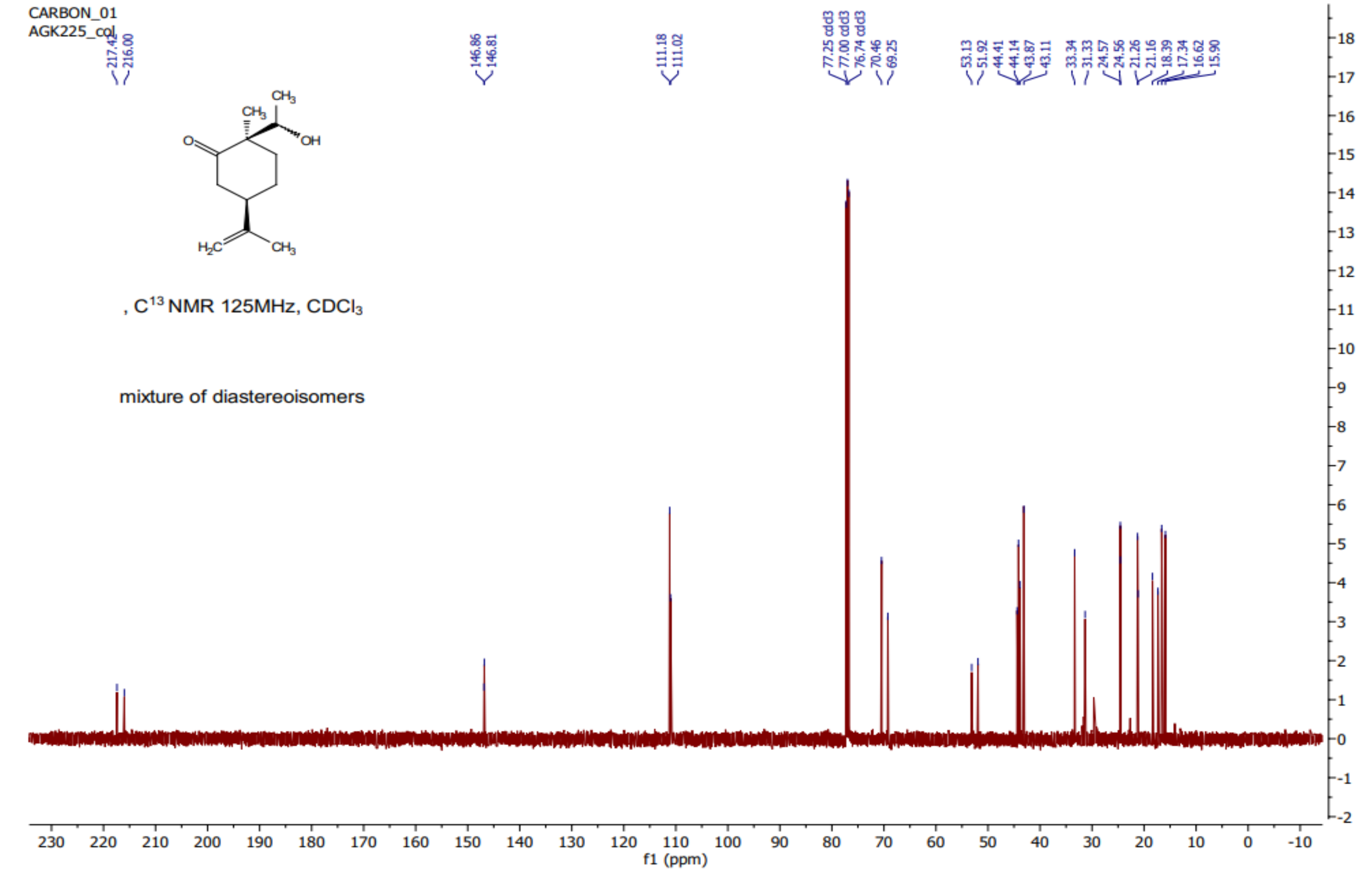
CARBON_01
AGK225_col
, C13 NMR 125MHz, CDCl3
mixture of diastereoisomers
f1 (ppm)

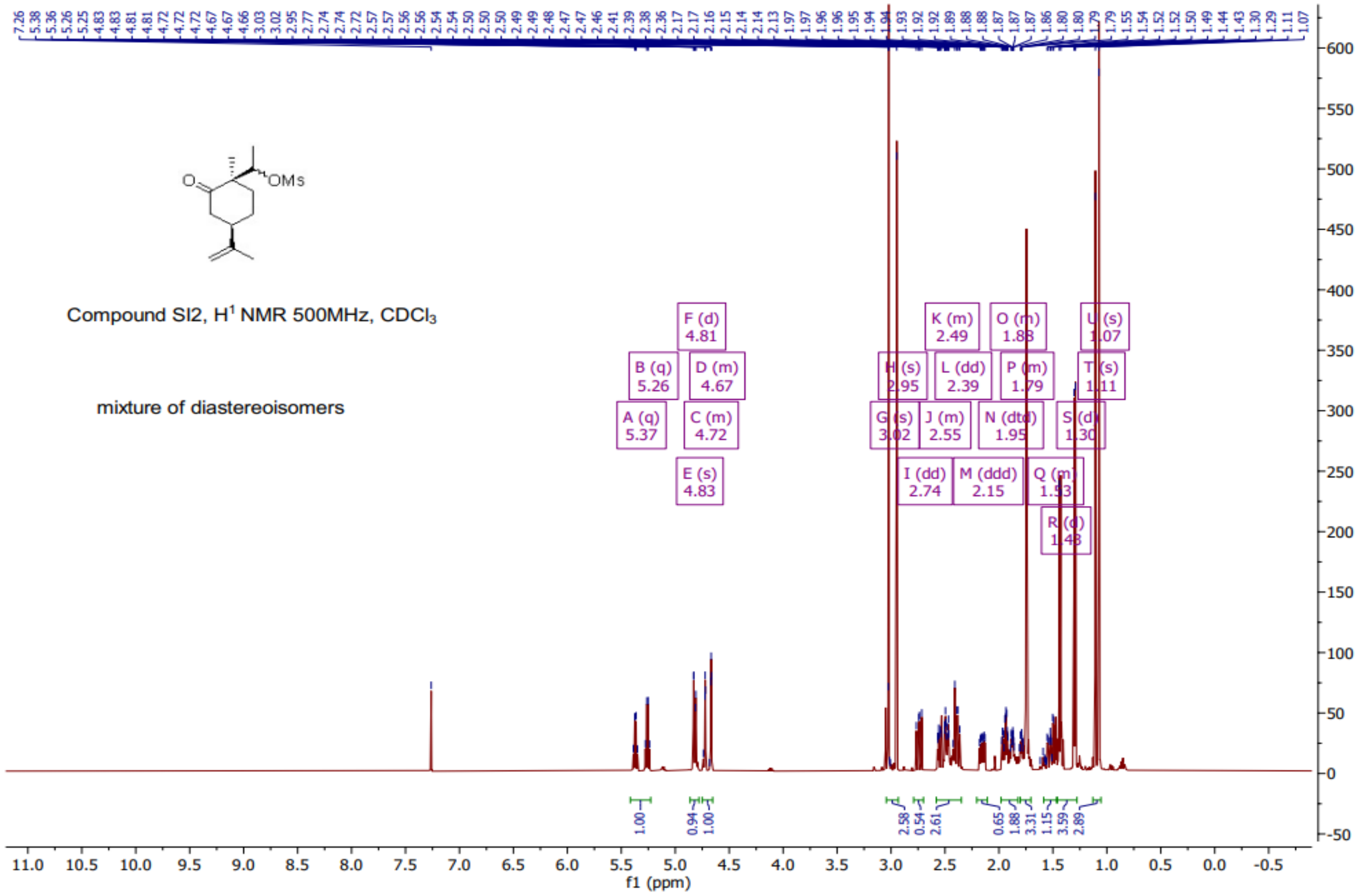
Compound SI2, H[1] NMR 500MHz, CDCl$_3$
mixture of diastereoisomers
OMs
f1 (ppm)

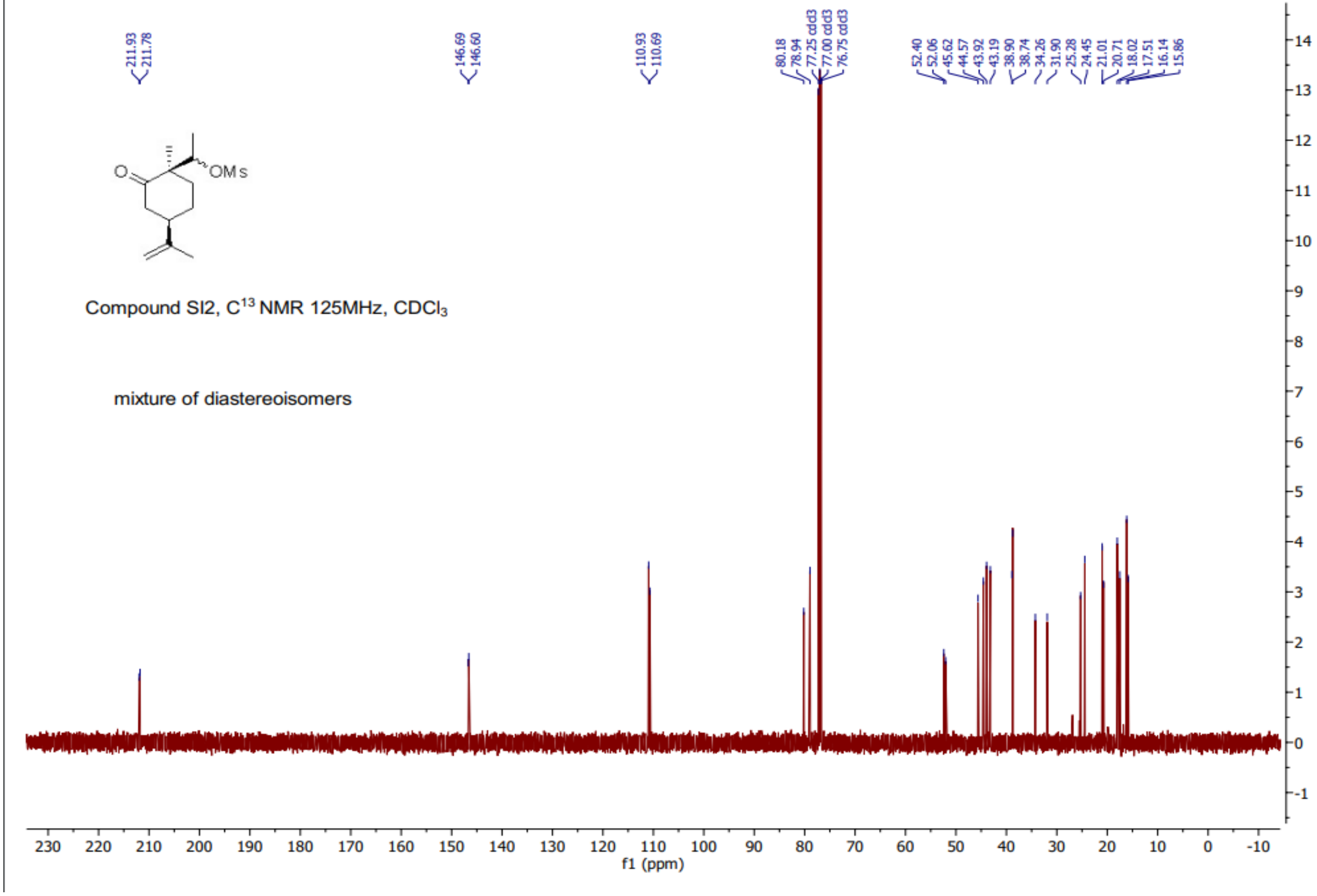
Compound SI2, C[13] NMR 125MHz, CDCl$_3$
mixture of diastereoisomers
OMs
f1 (ppm)

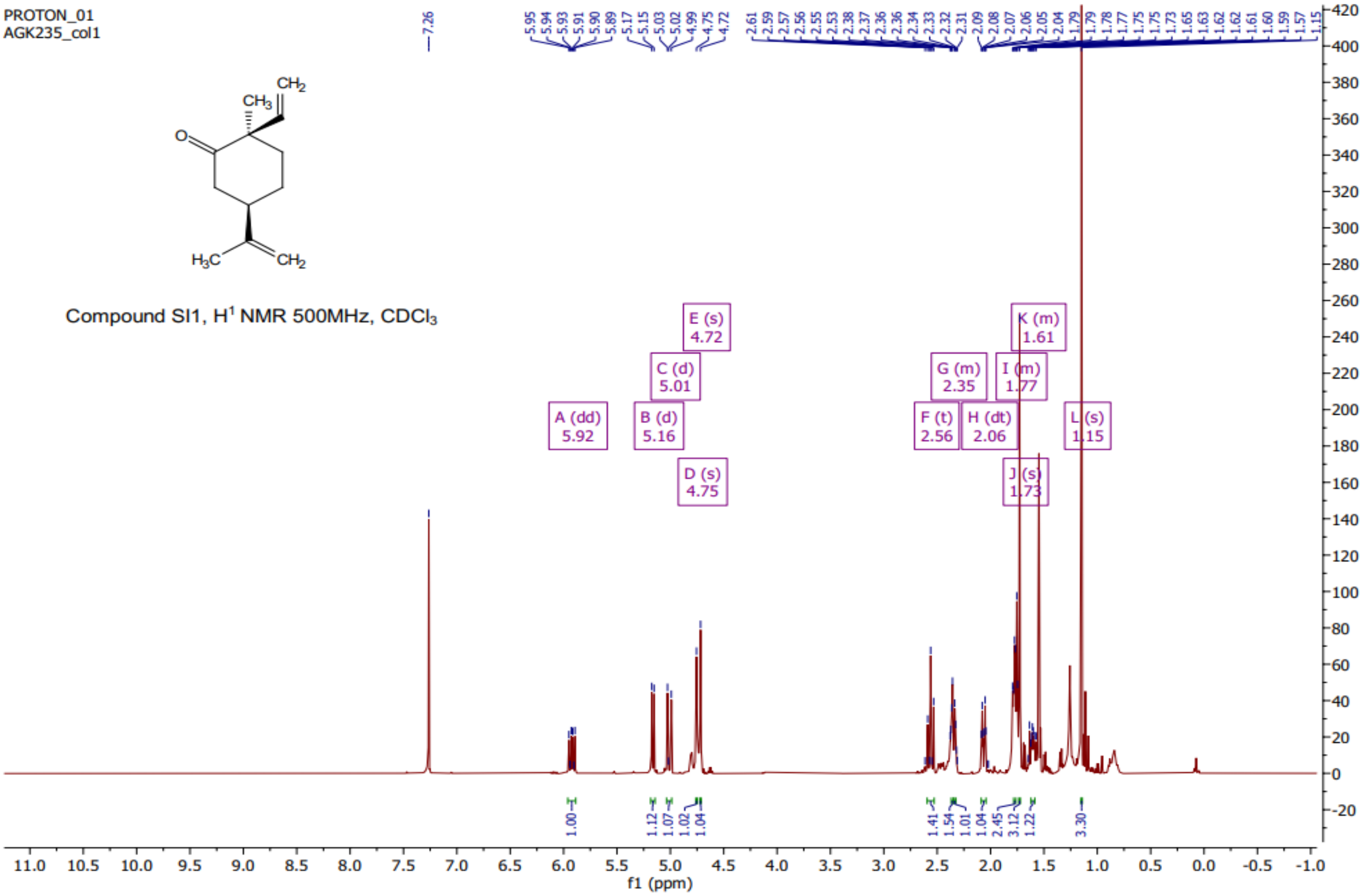
PROTON_01
AGK235_col1
Compound SI1, H1 NMR 500MHz, CDCl3
A (dd) 5.92
B (d) 5.16
C (d) 5.01
D (s) 4.75
E (s) 4.72
F (t) 2.56
G (m) 2.35
H (dt) 2.06
I (m) 1.77
J (s) 1.73
K (m) 1.61
L (s) 1.15
f1 (ppm)

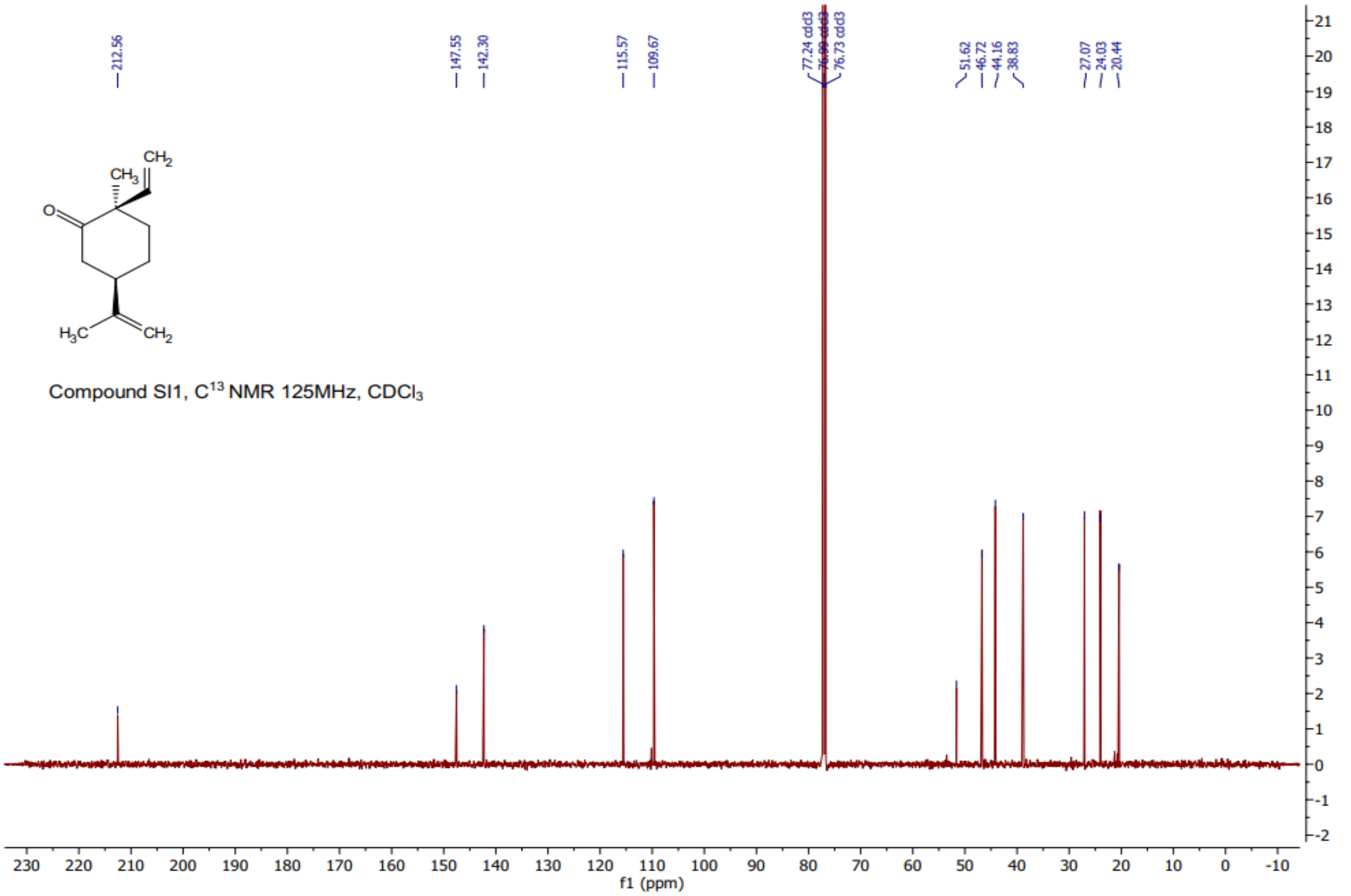
Compound SI1, C13 NMR 125MHz, CDCl3
212.56
147.55
142.30
115.57
109.67
77.24 cdcl3
76.73 cdcl3
51.62
46.72
44.16
38.83
27.07
24.03
20.44
f1 (ppm)

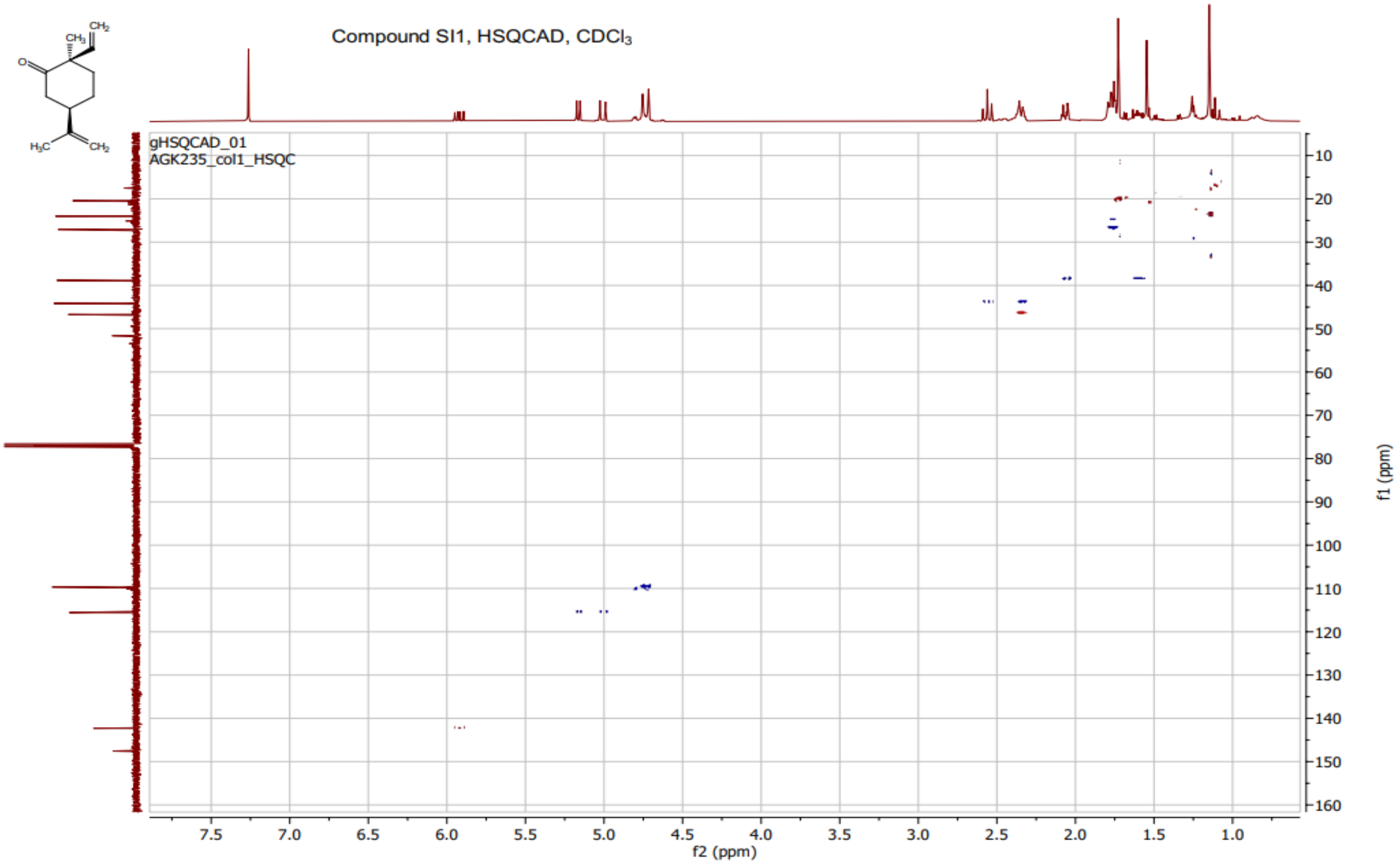
Compound SI1, HSQCAD, $CDCl_3$
gHSQCAD_01
AGK235_col1_HSQC
f2 (ppm)
f1 (ppm)

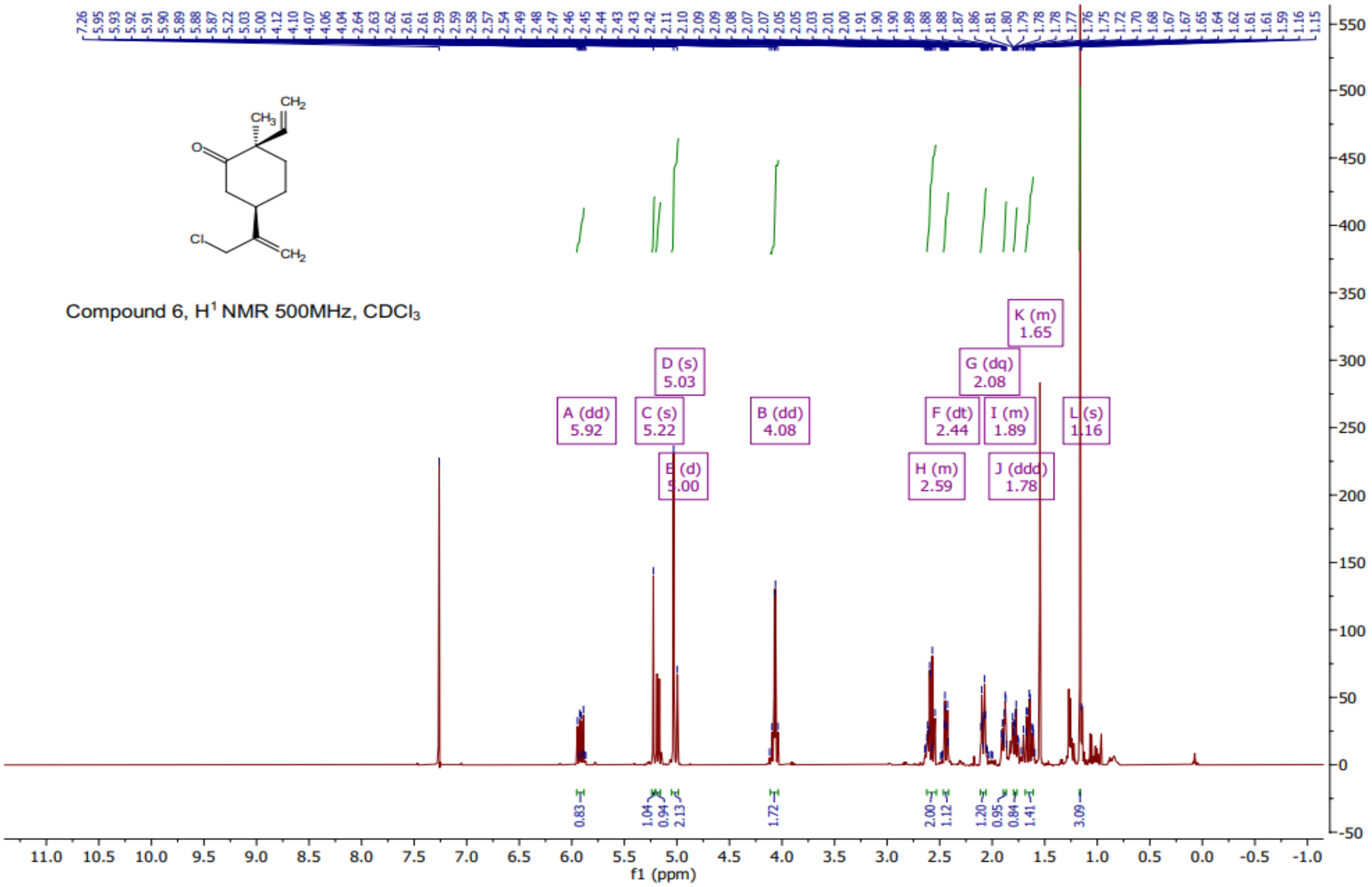
Compound 6, H[1] NMR 500MHz, $CDCl_3$
A (dd) 5.92
B (dd) 4.08
C (s) 5.22
D (s) 5.03
E (d) 5.00
F (dt) 2.44
G (dq) 2.08
H (m) 2.59
I (m) 1.89
J (ddd) 1.78
K (m) 1.65
L (s) 1.16
f1 (ppm)

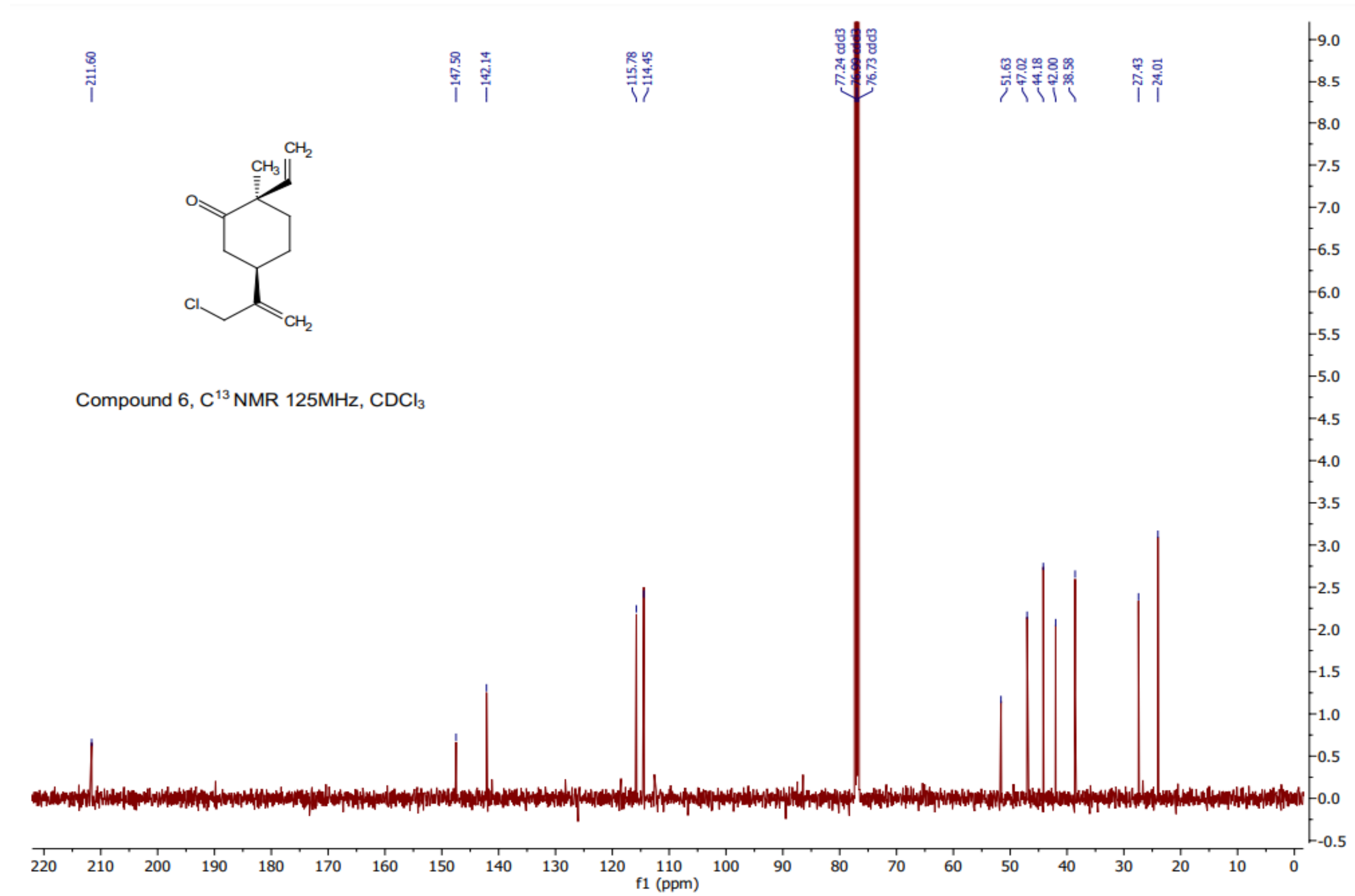
Compound 6, C13 NMR 125MHz, CDCl3
211.60
147.50
142.14
115.78
114.45
77.24 cdcl3
76.99 cdcl3
76.73 cdcl3
51.63
47.02
44.18
42.00
38.58
27.43
24.01
f1 (ppm)

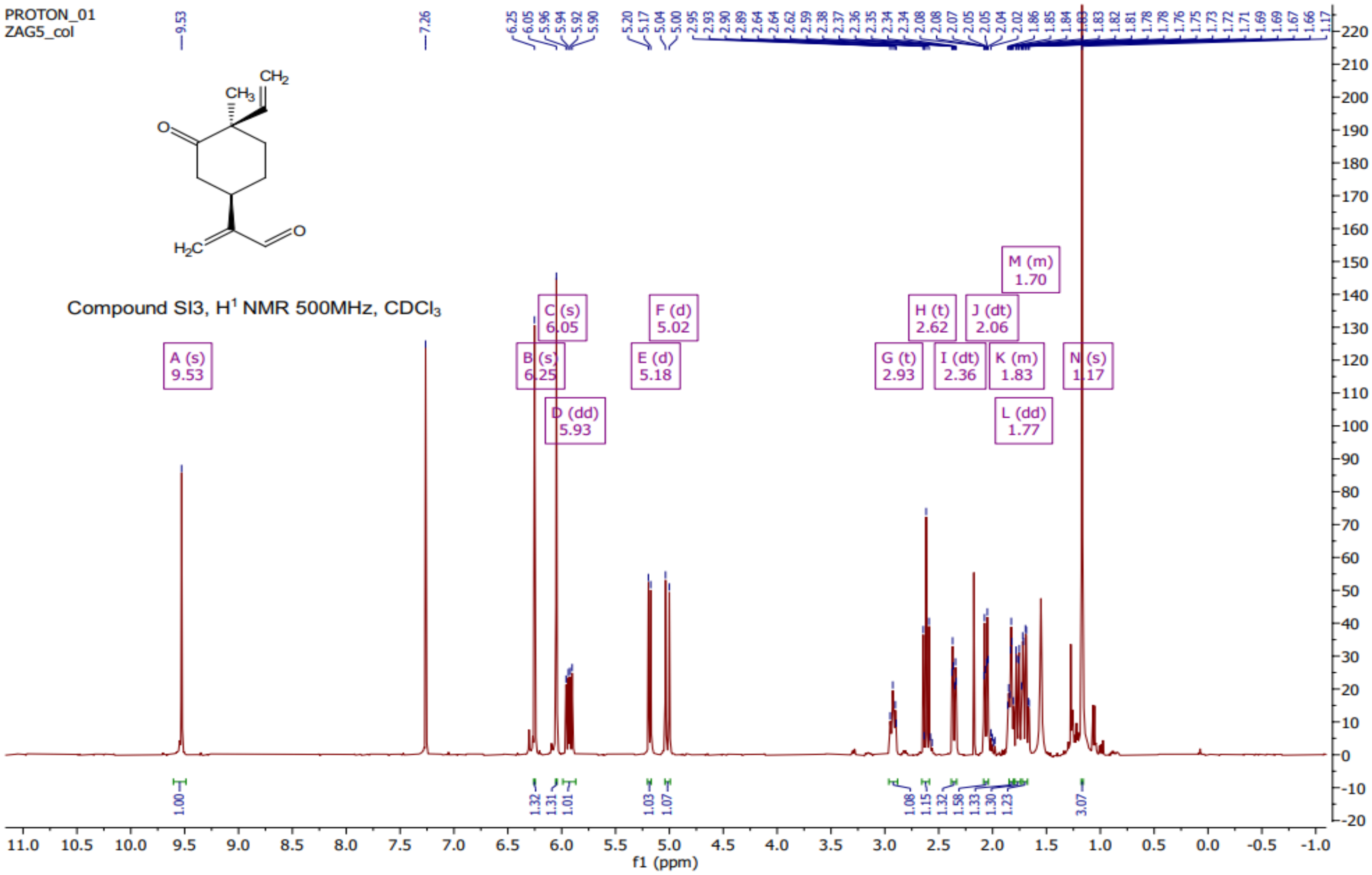
PROTON_01
ZAG5_col
Compound SI3, H1 NMR 500MHz, CDCl3
A (s) 9.53
B (s) 6.25
C (s) 6.05
D (dd) 5.93
E (d) 5.18
F (d) 5.02
G (t) 2.93
H (t) 2.62
I (dt) 2.36
J (dt) 2.06
K (m) 1.83
L (dd) 1.77
M (m) 1.70
N (s) 1.17
f1 (ppm)

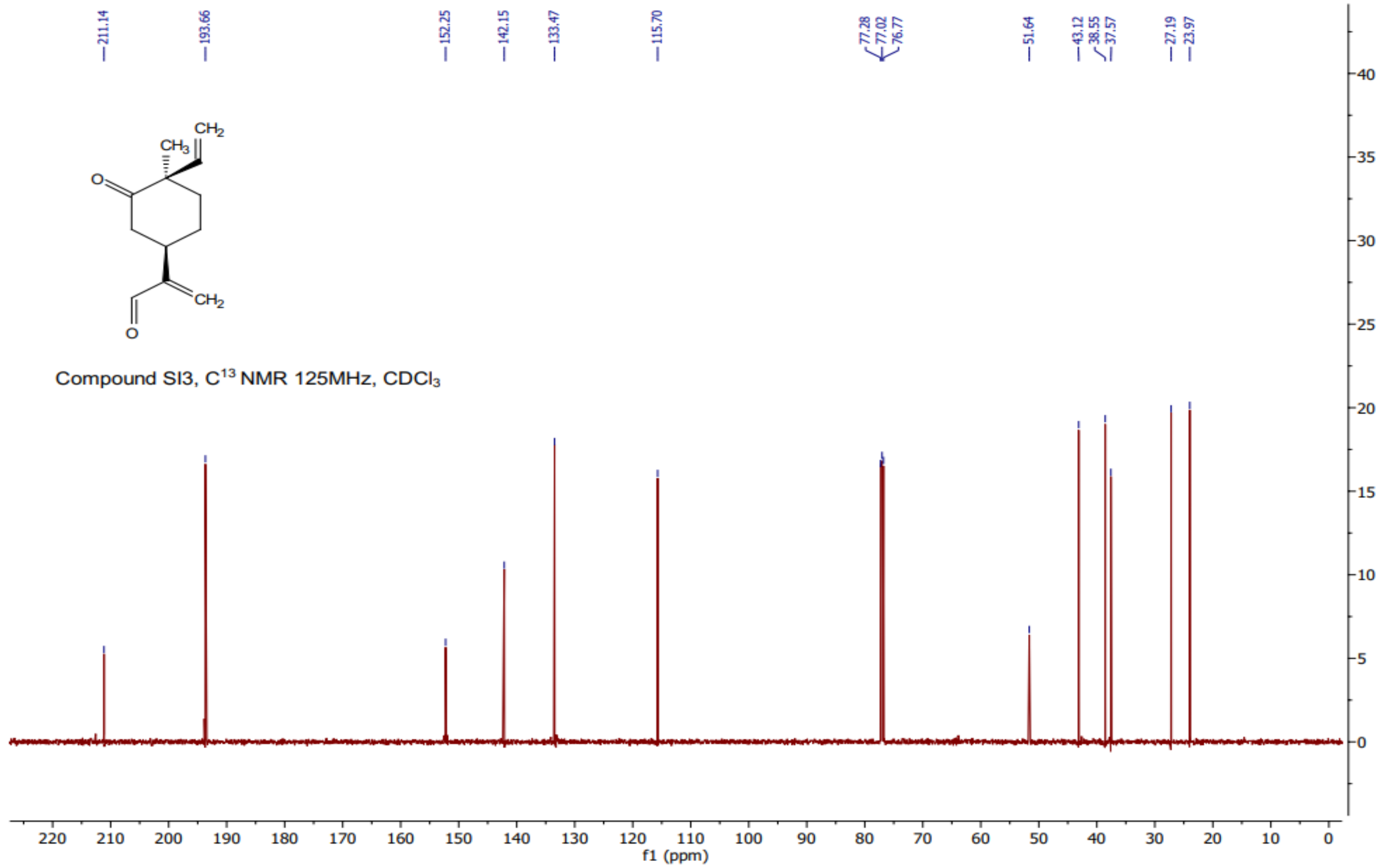
211.14
193.66
152.25
142.15
133.47
115.70
77.28
77.02
76.77
51.64
43.12
38.55
37.57
27.19
23.97
Compound SI3, C13 NMR 125MHz, CDCl3
f1 (ppm)

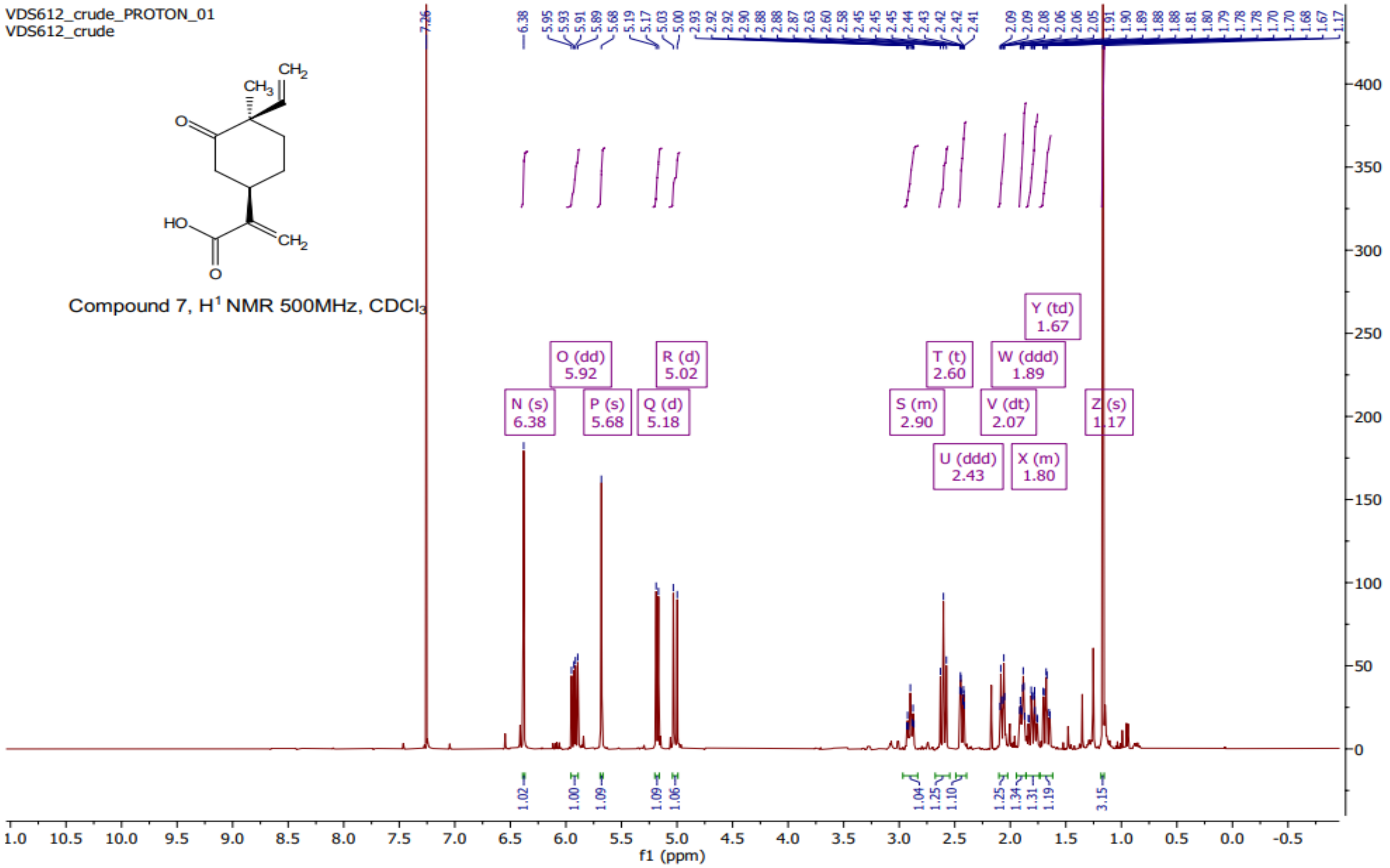
VDS612_crude_PROTON_01
VDS612_crude
Compound 7, H1 NMR 500MHz, CDCl3
N (s) 6.38
O (dd) 5.92
P (s) 5.68
Q (d) 5.18
R (d) 5.02
S (m) 2.90
T (t) 2.60
U (ddd) 2.43
V (dt) 2.07
W (ddd) 1.89
X (m) 1.80
Y (td) 1.67
Z (s) 1.17
f1 (ppm)

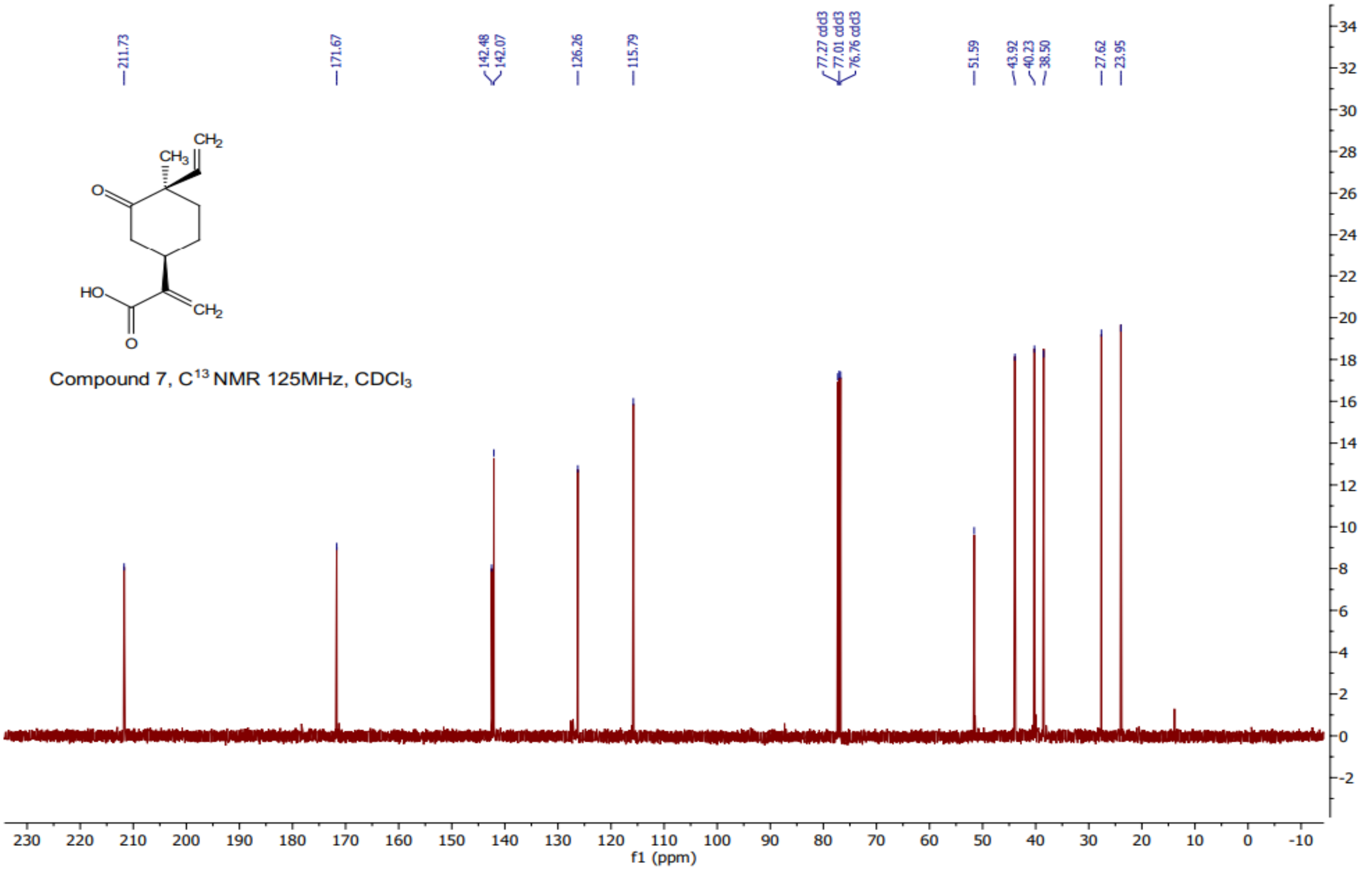

Compound 7, C[13] NMR 125MHz, CDCl3
211.73
171.67
142.48
142.07
126.26
115.79
77.27 cdcl3
77.01 cdcl3
76.76 cdcl3
51.59
43.92
40.23
38.50
27.62
23.95
f1 (ppm)


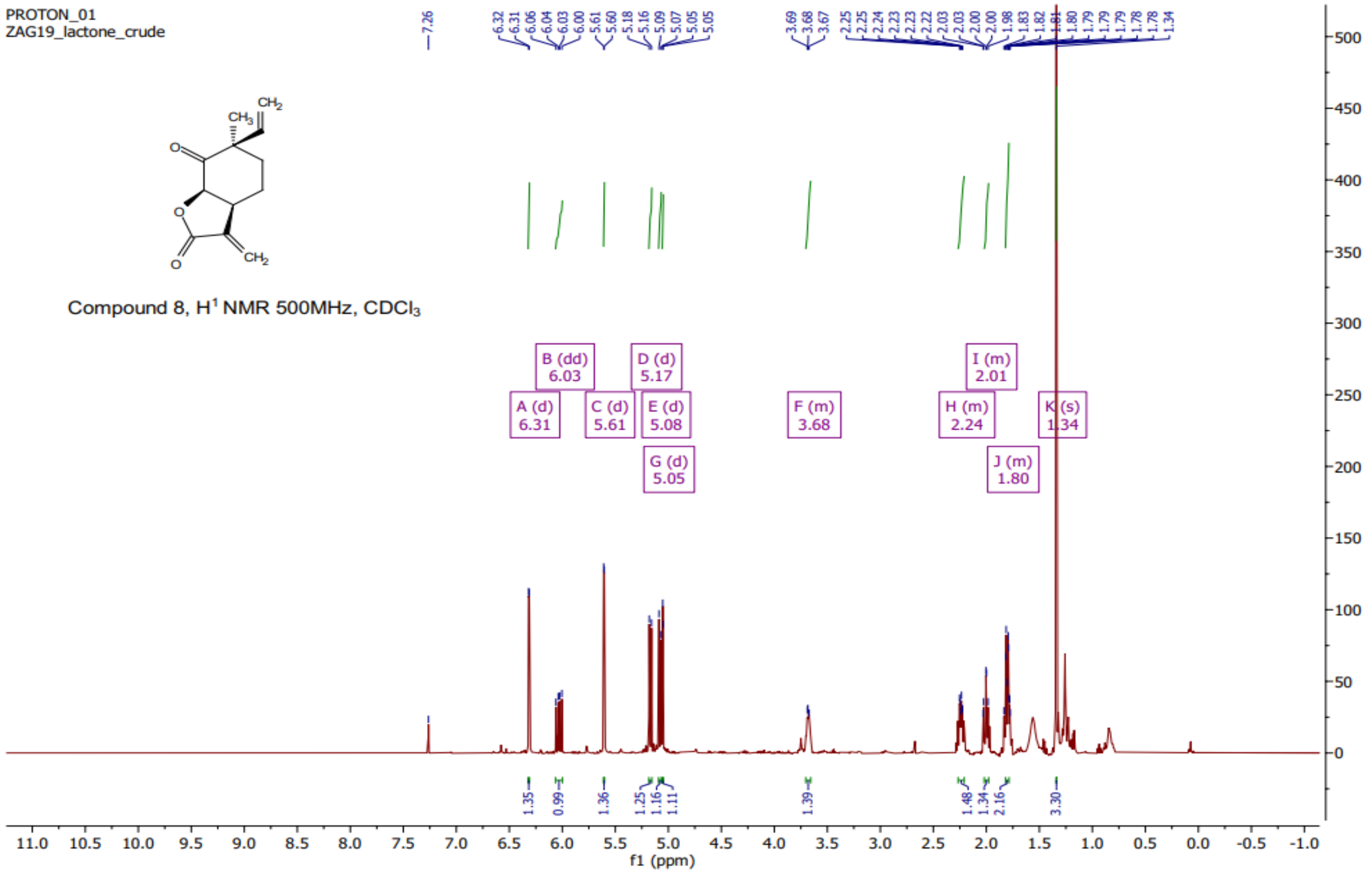

PROTON_01
ZAG19_lactone_crude
Compound 8, H[1] NMR 500MHz, CDCl3
A (d) 6.31
B (dd) 6.03
C (d) 5.61
D (d) 5.17
E (d) 5.08
G (d) 5.05
F (m) 3.68
H (m) 2.24
I (m) 2.01
J (m) 1.80
K (s) 1.34
f1 (ppm)

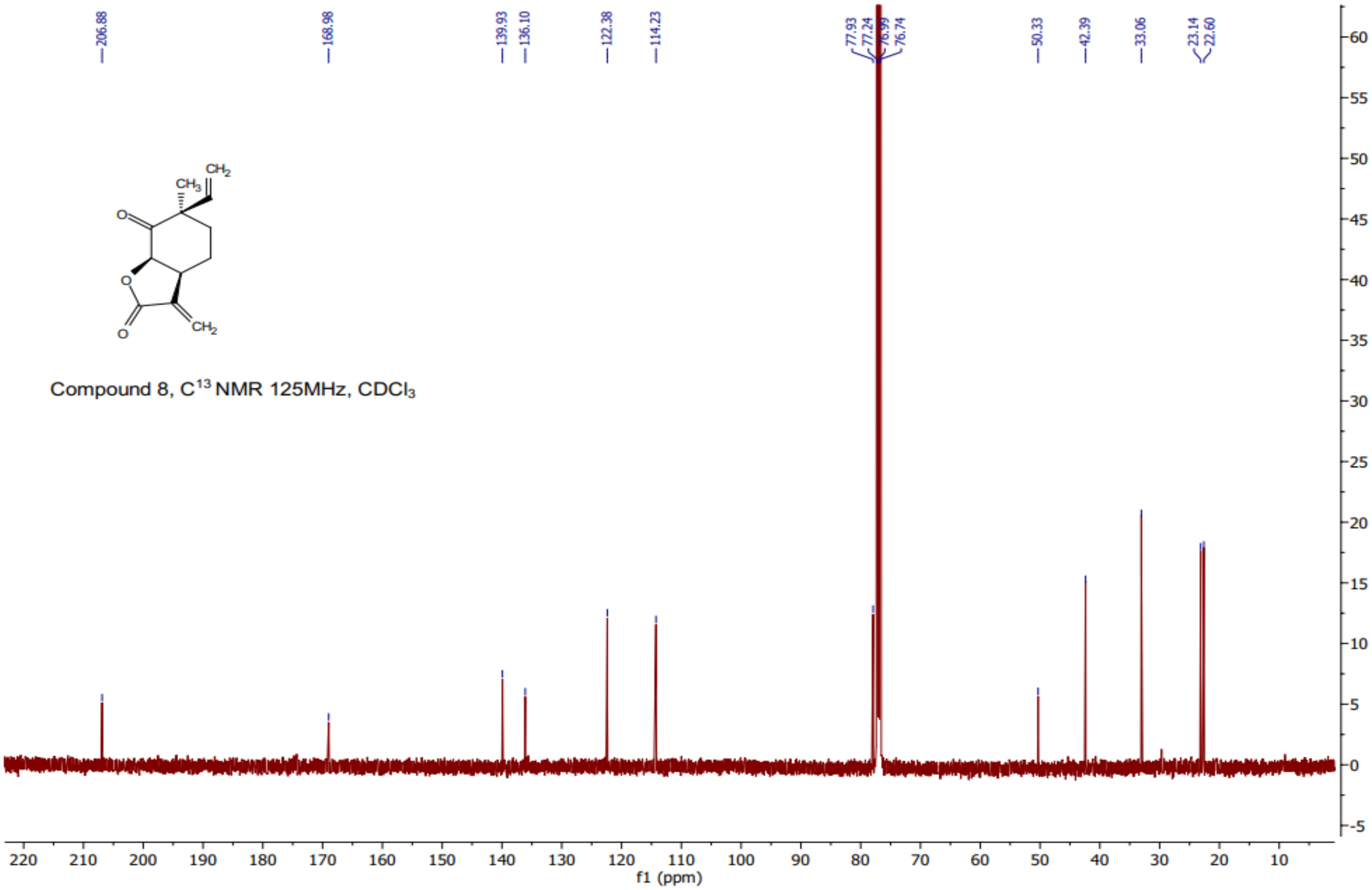
206.88
168.98
139.93
136.10
122.38
114.23
77.93
77.24
76.99
76.74
50.33
42.39
33.06
23.14
22.60
Compound 8, C13 NMR 125MHz, CDCl3
f1 (ppm)

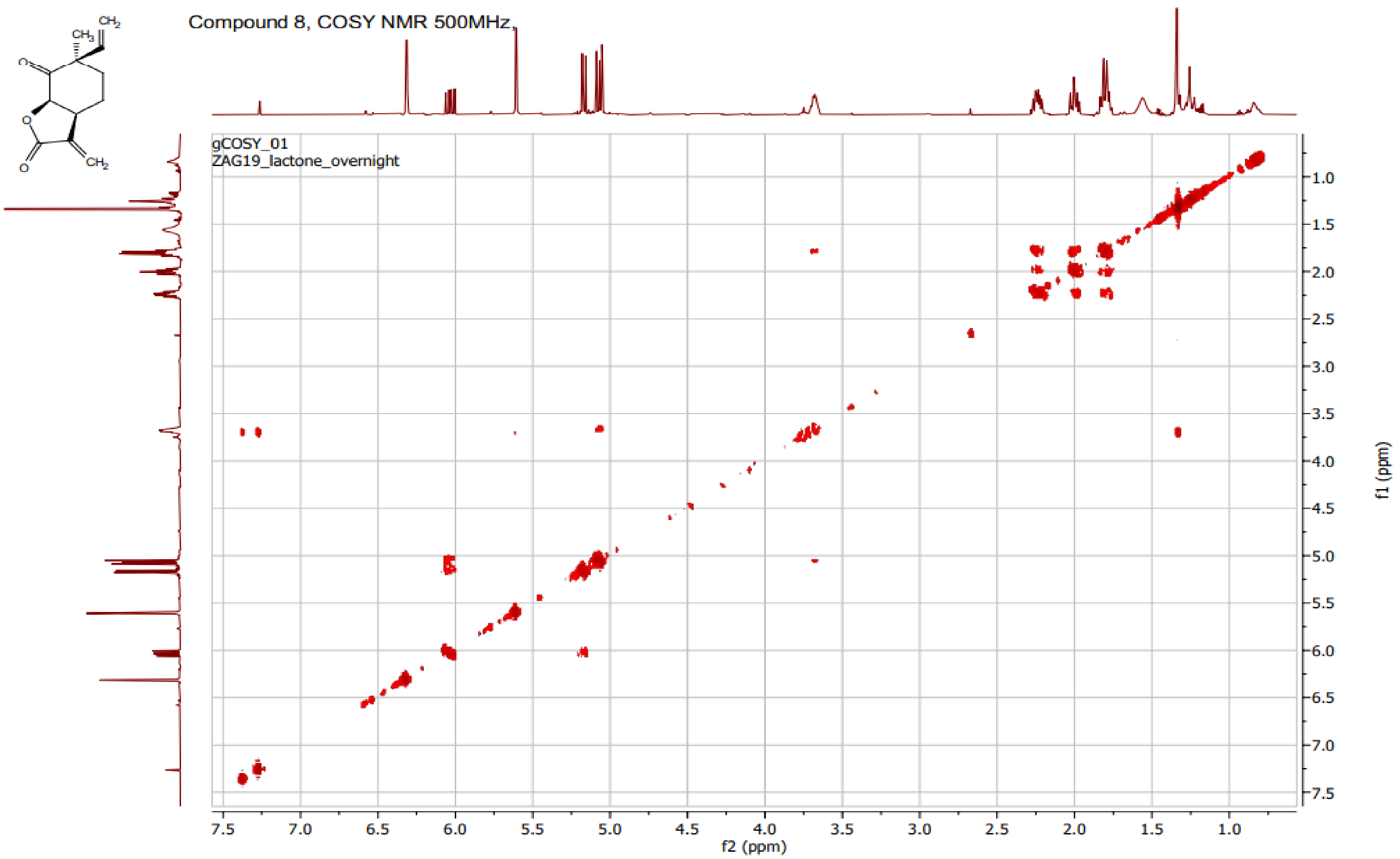
Compound 8, COSY NMR 500MHz,
gCOSY_01
ZAG19_lactone_overnight
f2 (ppm)
f1 (ppm)

Compound 8, NOESY NMR 500MHz, $CDCl_3$

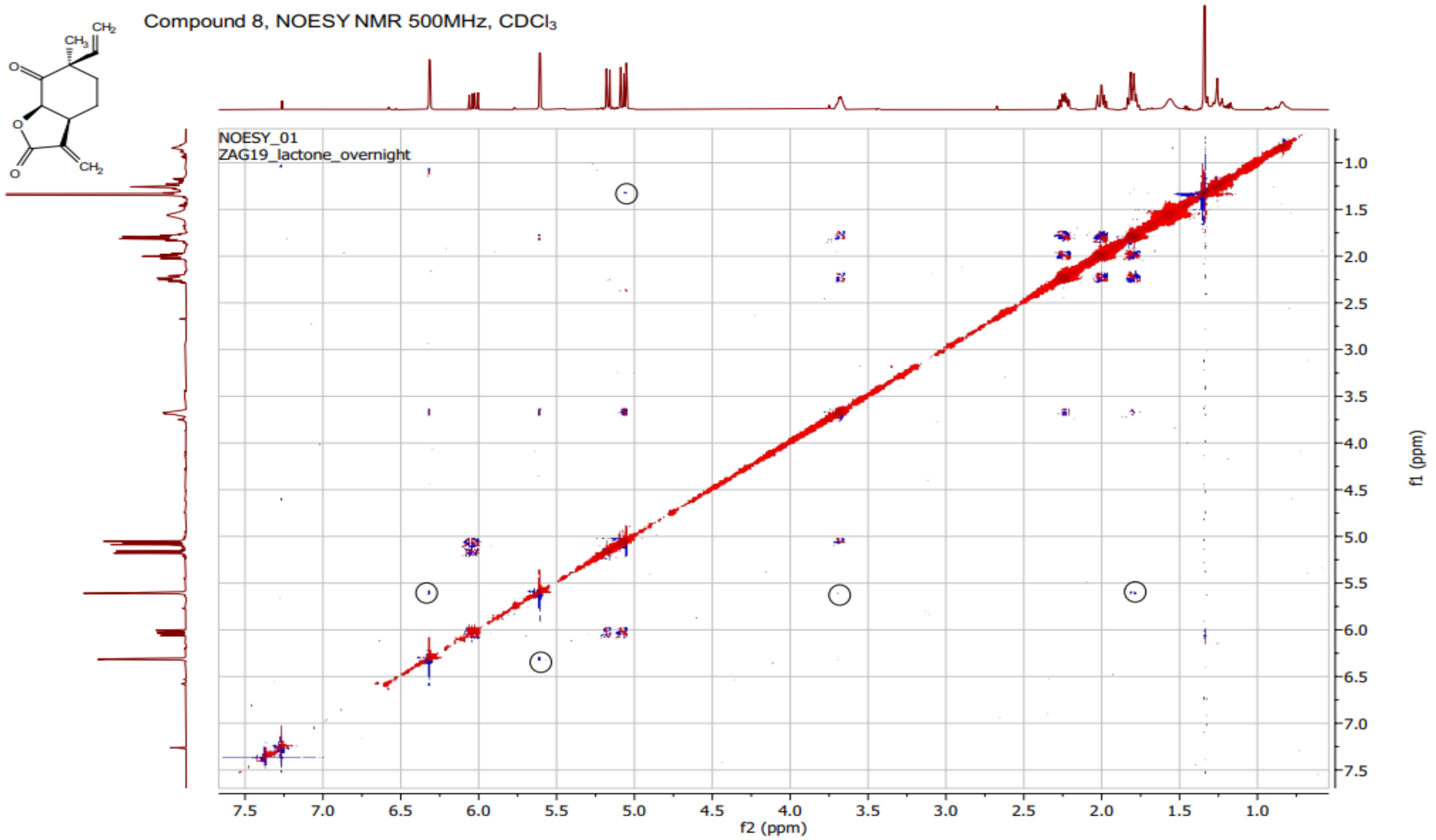


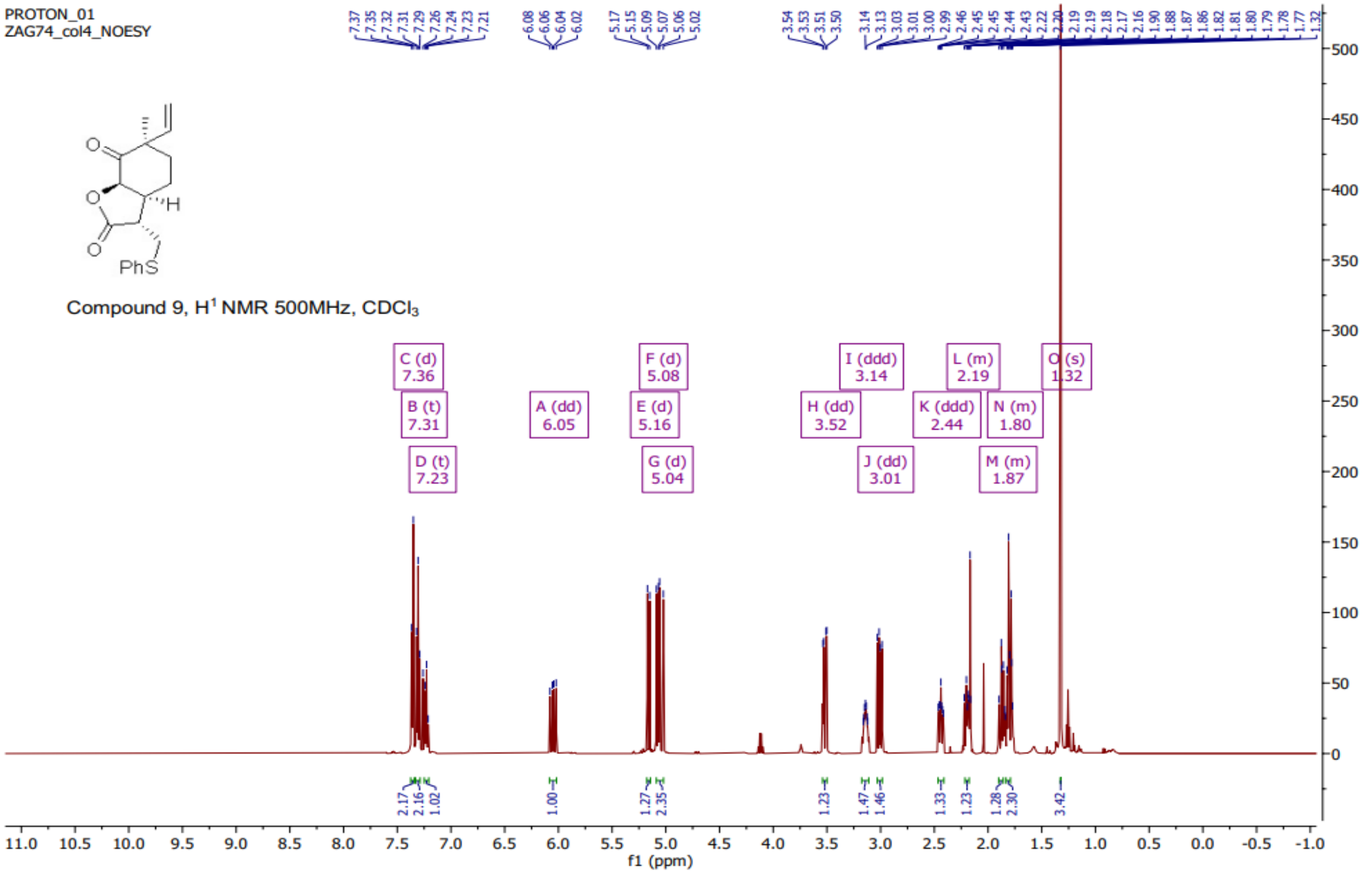


Compound 9, H[1] NMR 500MHz, $CDCl_3$

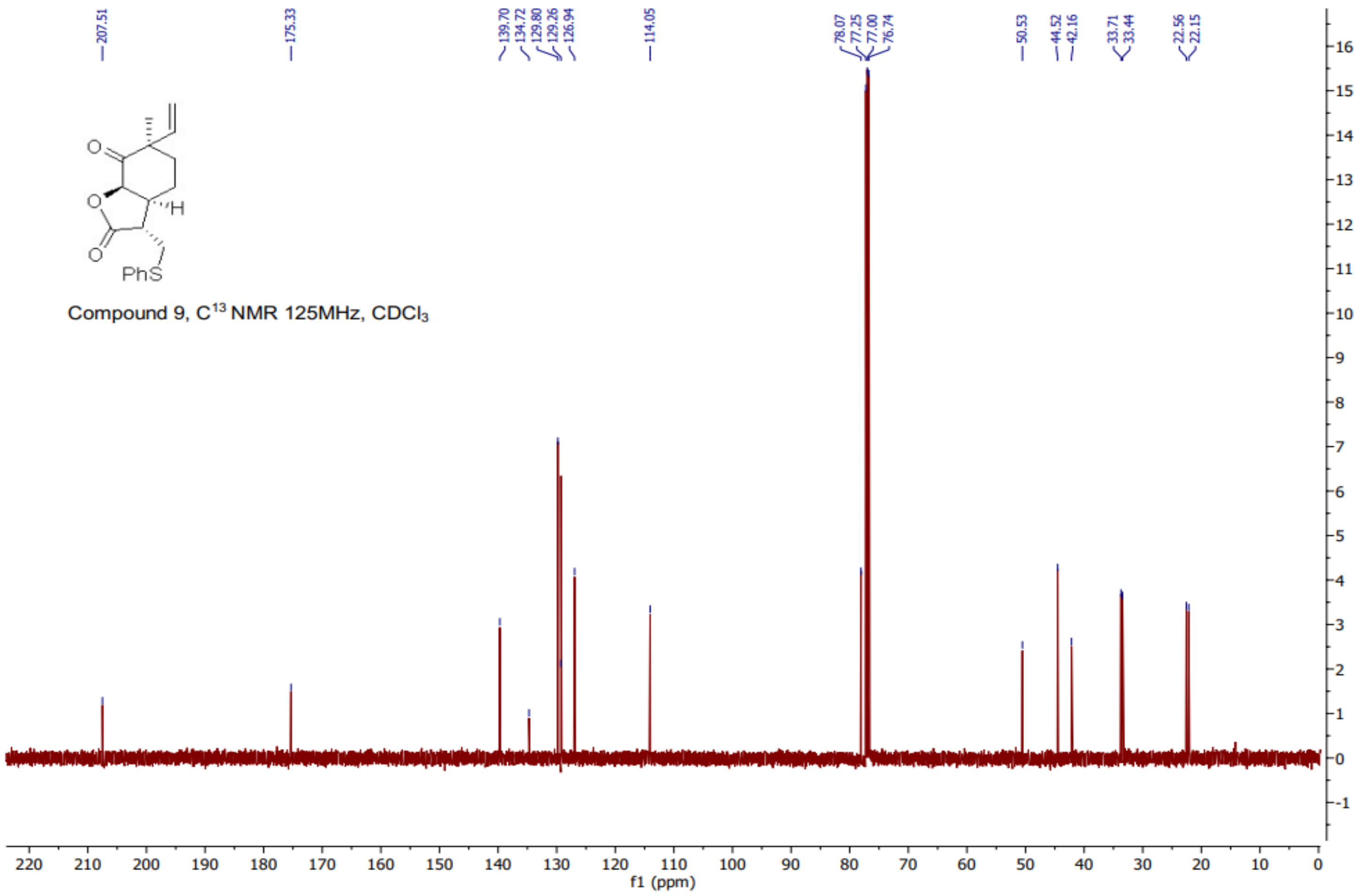
Compound 9, C13 NMR 125MHz, CDCl3
PhS
207.51
175.33
139.70
134.72
129.80
129.26
126.94
114.05
78.07
77.25
77.00
76.74
50.53
44.52
42.16
33.71
33.44
22.56
22.15
f1 (ppm)

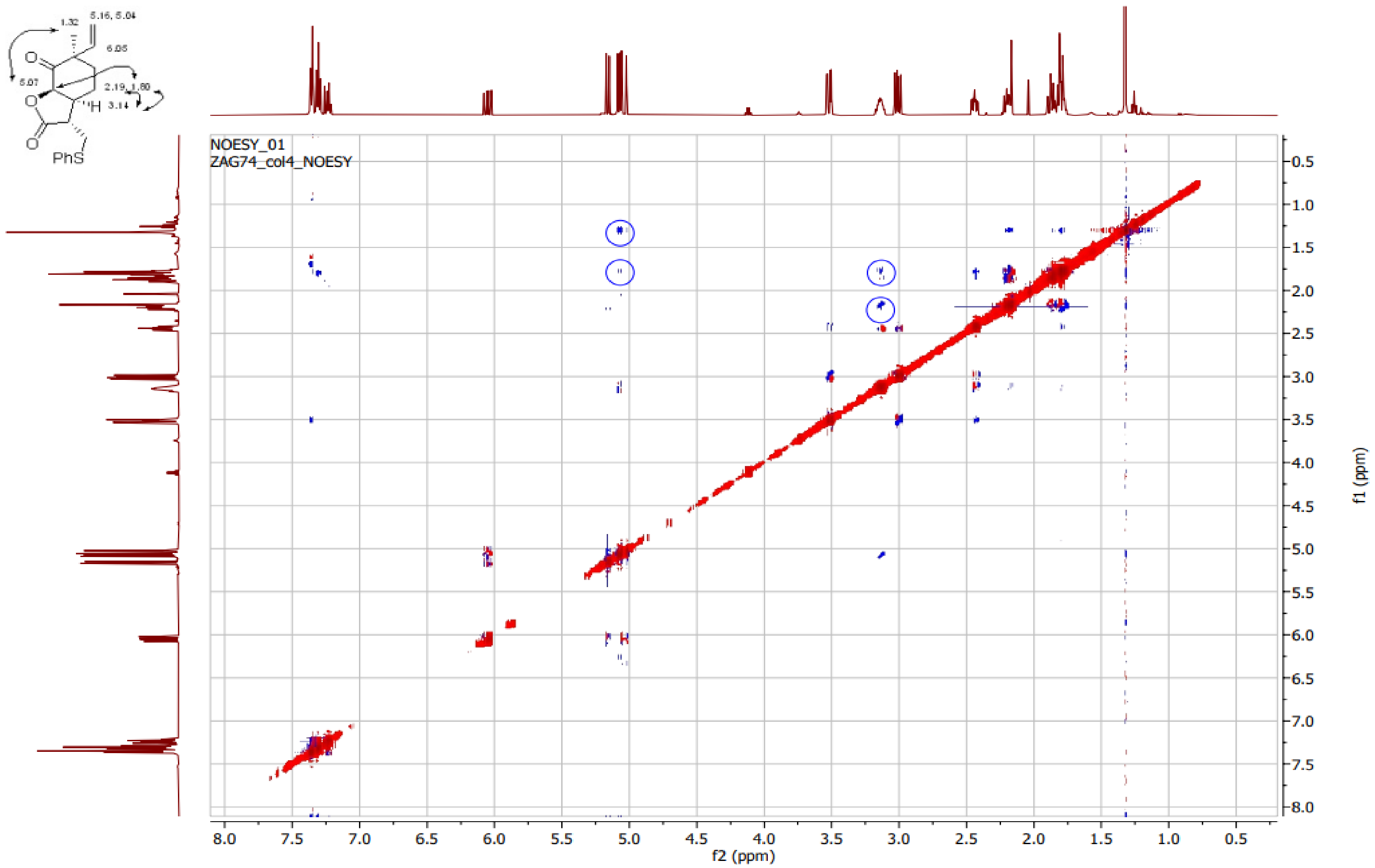
1.32
5.16, 5.04
6.05
5.07
2.19, 1.80
3.14
PhS
NOESY_01
ZAG74_col4_NOESY
f2 (ppm)
f1 (ppm)

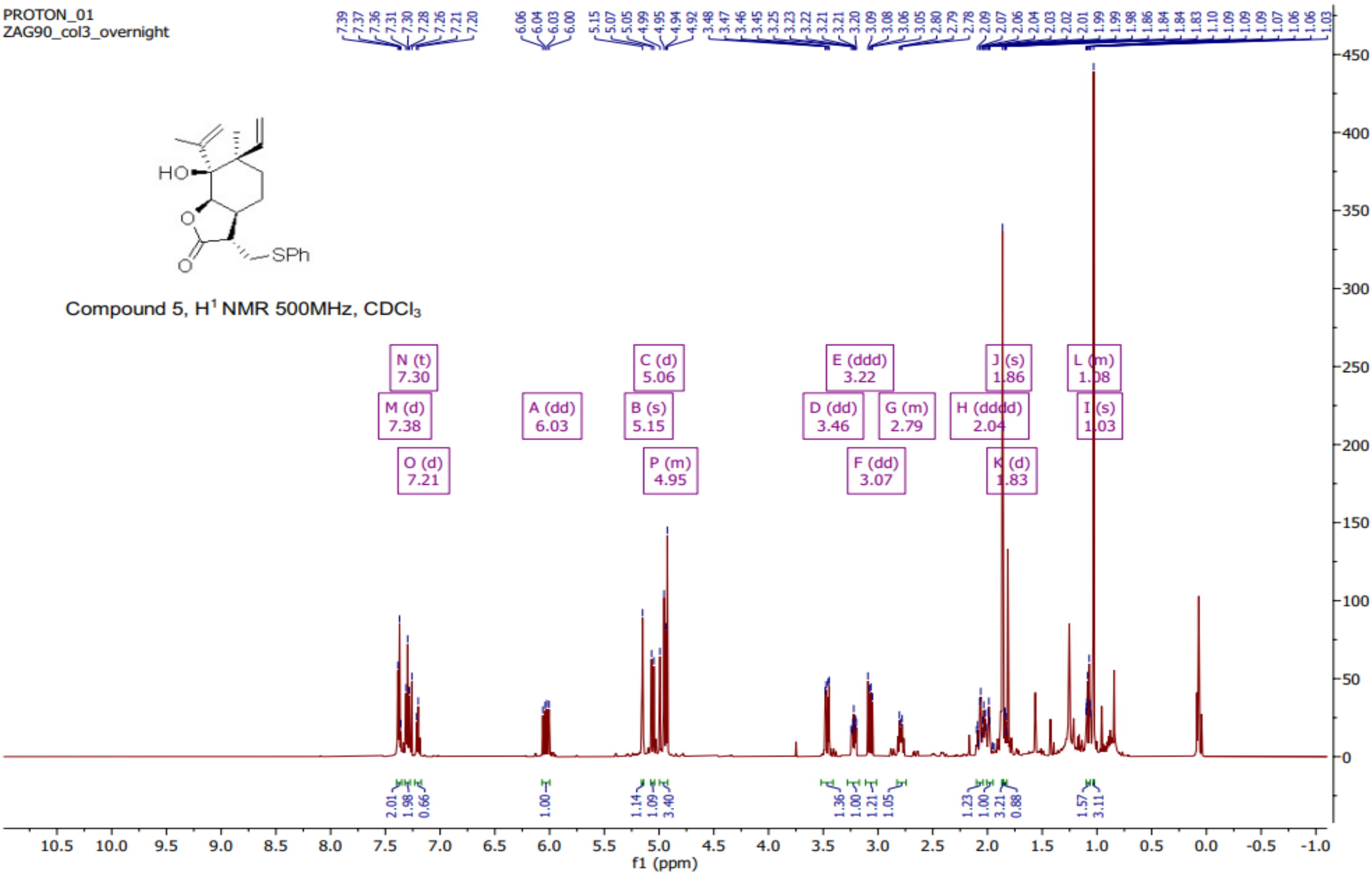
PROTON_01
ZAG90_col3_overnight
SPh
HO
O
Compound 5, H1 NMR 500MHz, CDCl3
N (t) 7.30
M (d) 7.38
O (d) 7.21
A (dd) 6.03
C (d) 5.06
B (s) 5.15
P (m) 4.95
E (ddd) 3.22
D (dd) 3.46
G (m) 2.79
F (dd) 3.07
J (s) 1.86
H (dddd) 2.04
K (d) 1.83
L (m) 1.08
I (s) 1.03
f1 (ppm)

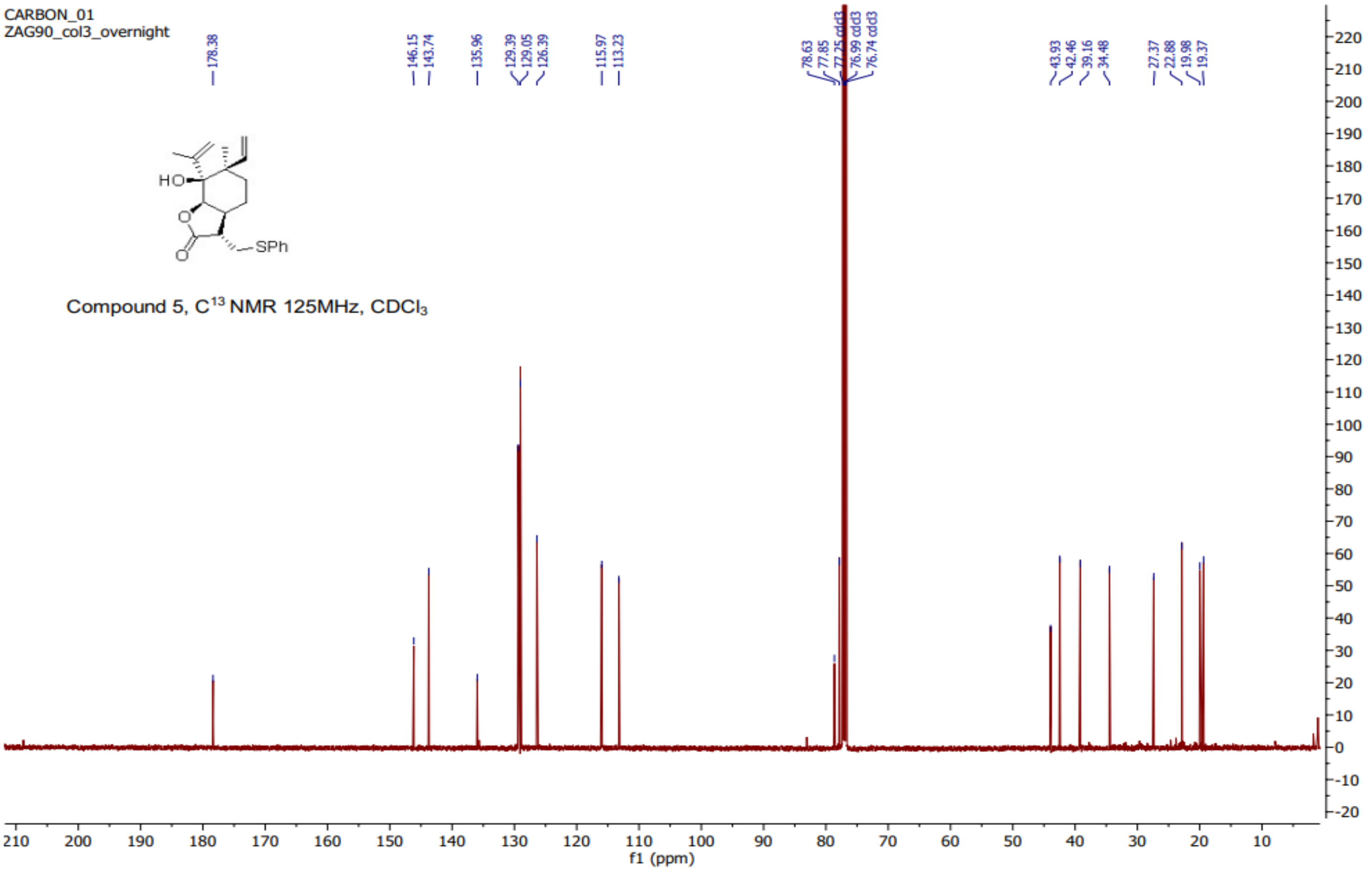
CARBON_01
ZAG90_col3_overnight
178.38
146.15
143.74
135.96
129.39
129.05
126.39
115.97
113.23
78.63
77.85
77.25 cdcl3
76.99 cdcl3
76.74 cdcl3
43.93
42.46
39.16
34.48
27.37
22.88
19.98
19.37
SPh
HO
O
Compound 5, C13 NMR 125MHz, CDCl3
f1 (ppm)

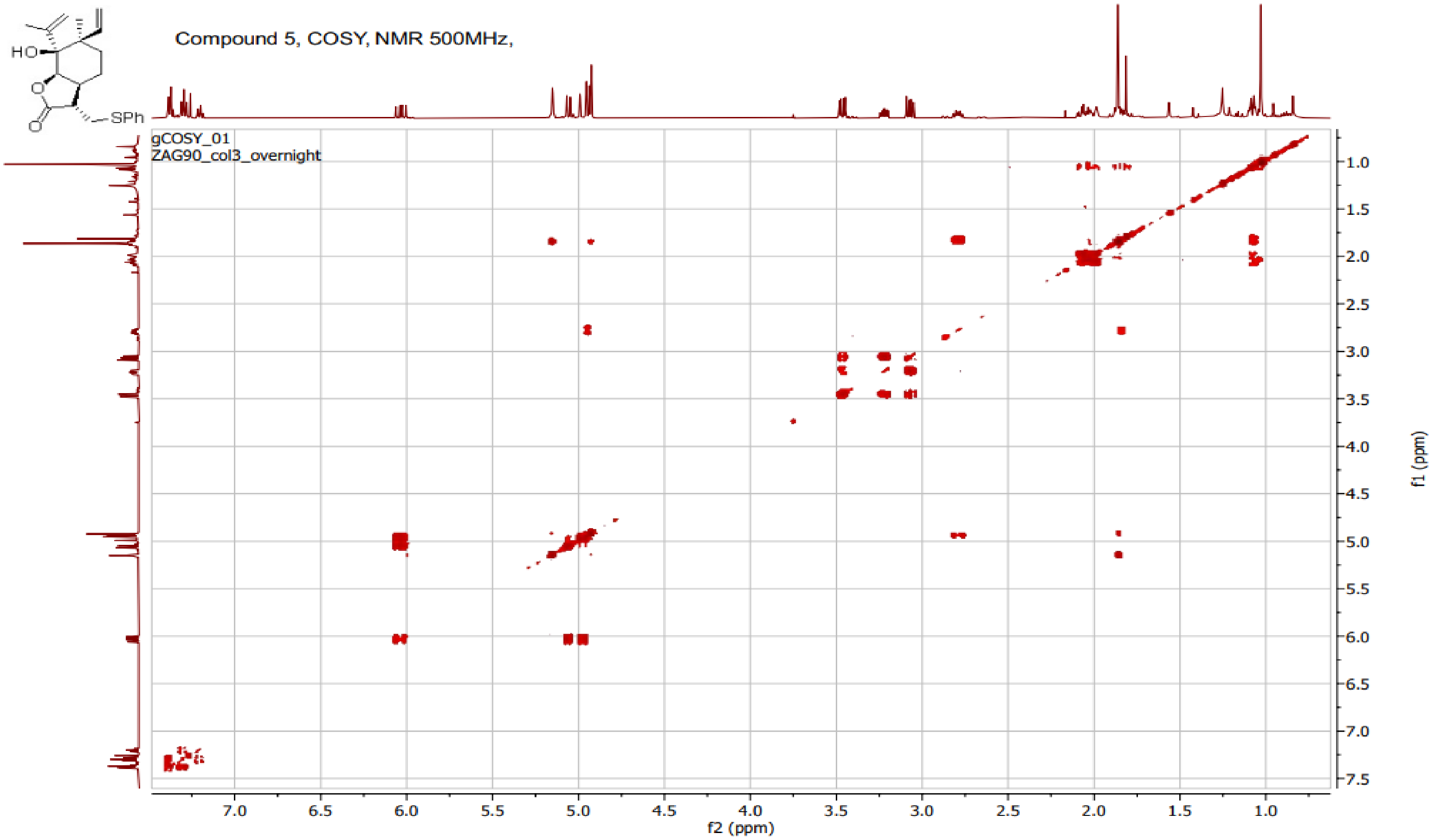
HO
O
O
SPh
Compound 5, COSY, NMR 500MHz,
gCOSY_01
ZAG90_col3_overnight
f2 (ppm)
f1 (ppm)

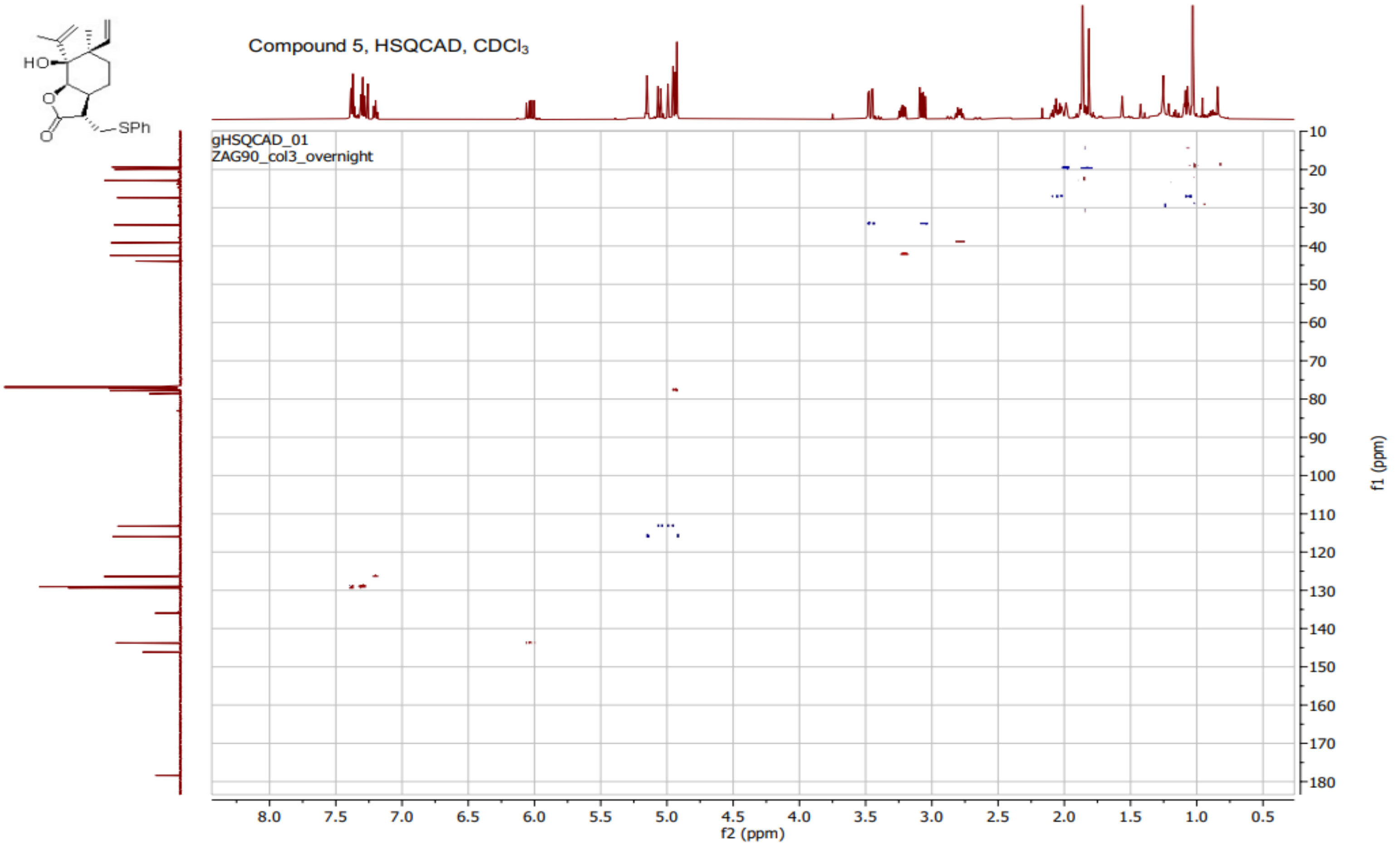
HO
O
O
SPh
Compound 5, HSQCAD, CDCl3
gHSQCAD_01
ZAG90_col3_overnight
f2 (ppm)
f1 (ppm)

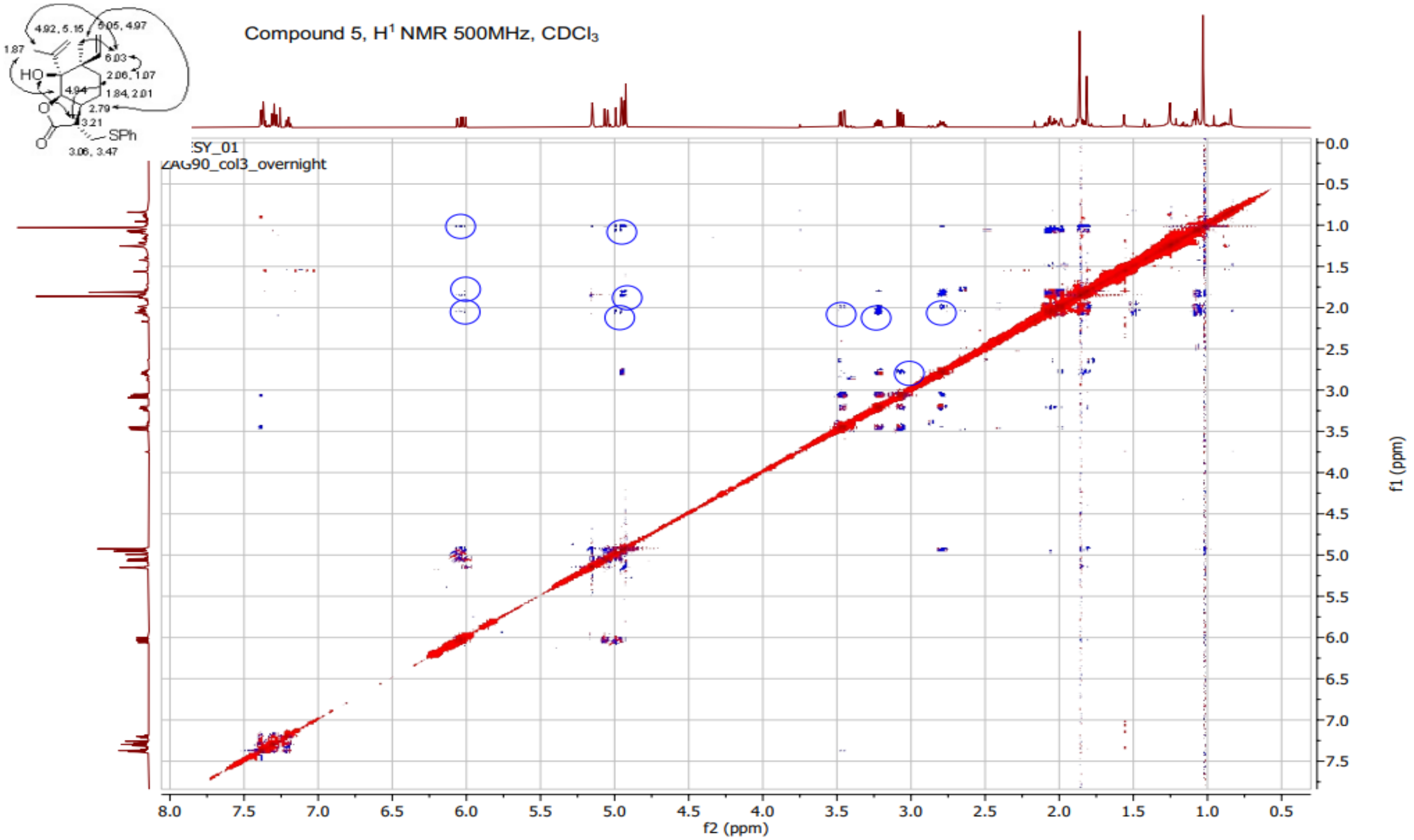
Compound 5, H[1] NMR 500MHz, CDCl$_3$
ZAG90_col3_overnight
f2 (ppm)
f1 (ppm)

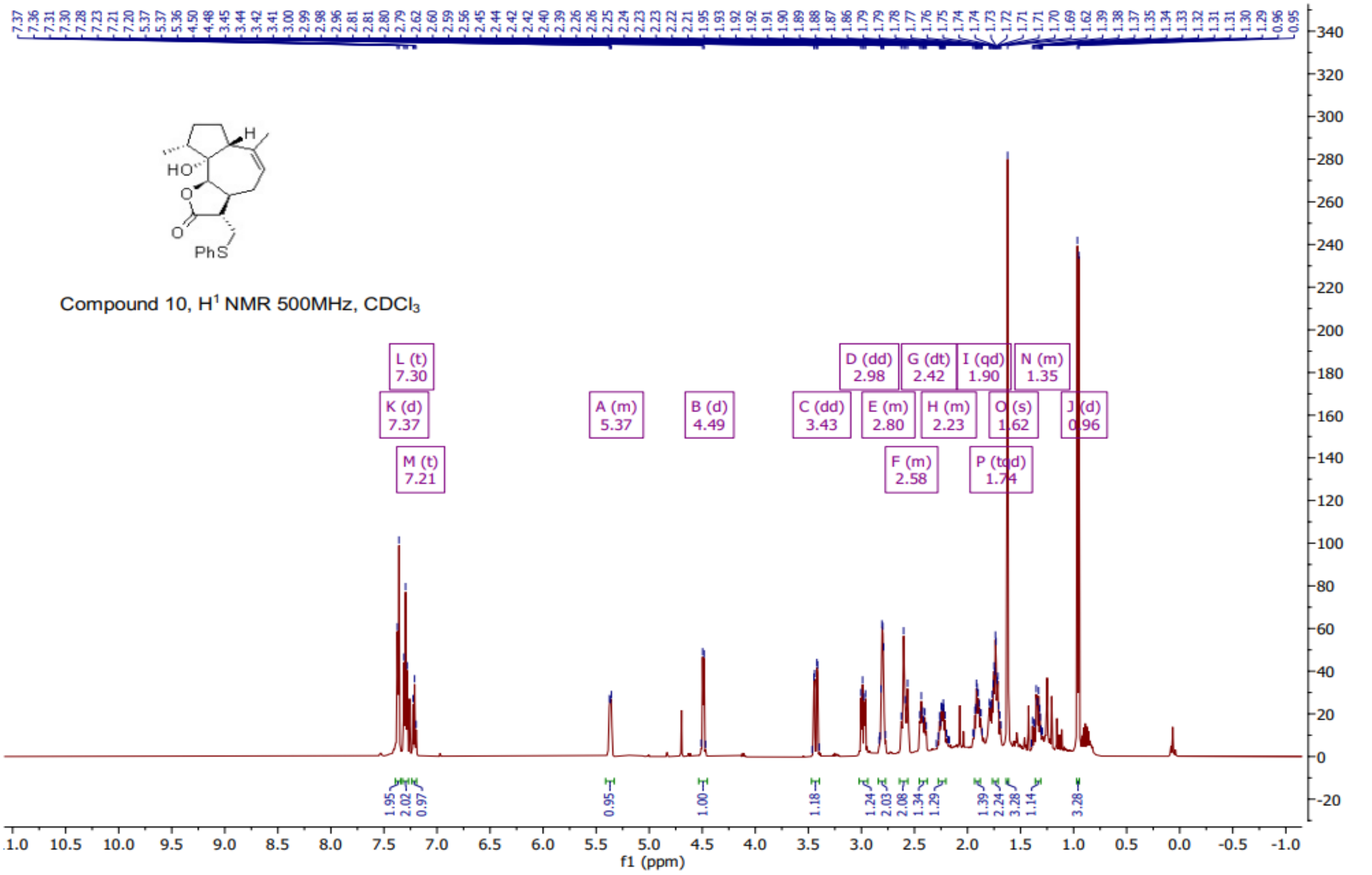
Compound 10, H[1] NMR 500MHz, CDCl$_3$
L (t) 7.30
K (d) 7.37
M (t) 7.21
A (m) 5.37
B (d) 4.49
C (dd) 3.43
D (dd) 2.98
E (m) 2.80
F (m) 2.58
G (dt) 2.42
H (m) 2.23
I (qd) 1.90
P (tdd) 1.74
O (s) 1.62
N (m) 1.35
J (d) 0.96
f1 (ppm)

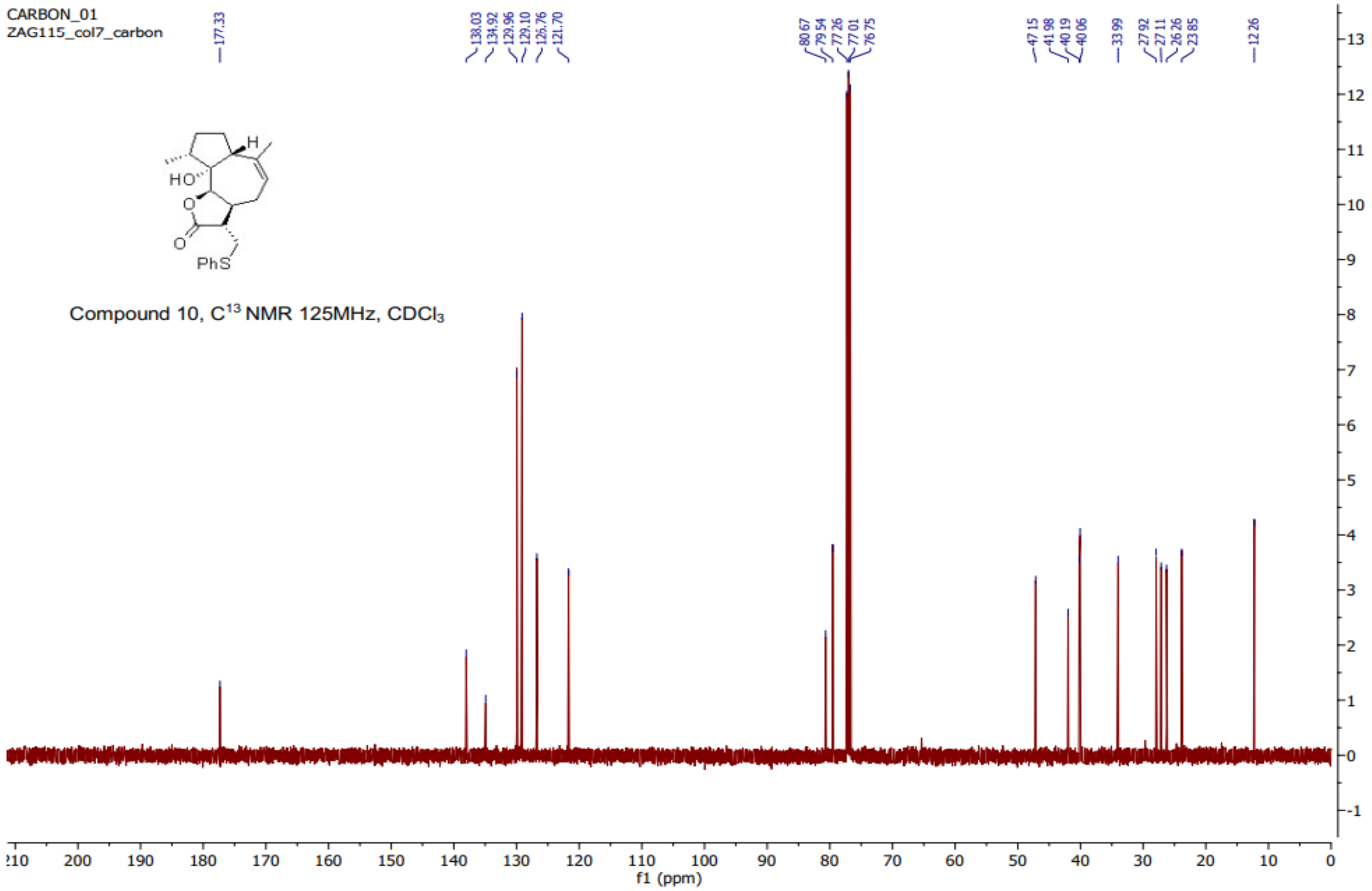
CARBON_01
ZAG115_col7_carbon
177.33
138.03
134.92
129.96
129.10
126.76
121.70
80.67
79.54
77.26
77.01
76.75
47.15
41.98
40.19
40.06
33.99
27.92
27.11
26.26
23.85
12.26
HO
H
O
PhS
Compound 10, C13 NMR 125MHz, CDCl3
f1 (ppm)

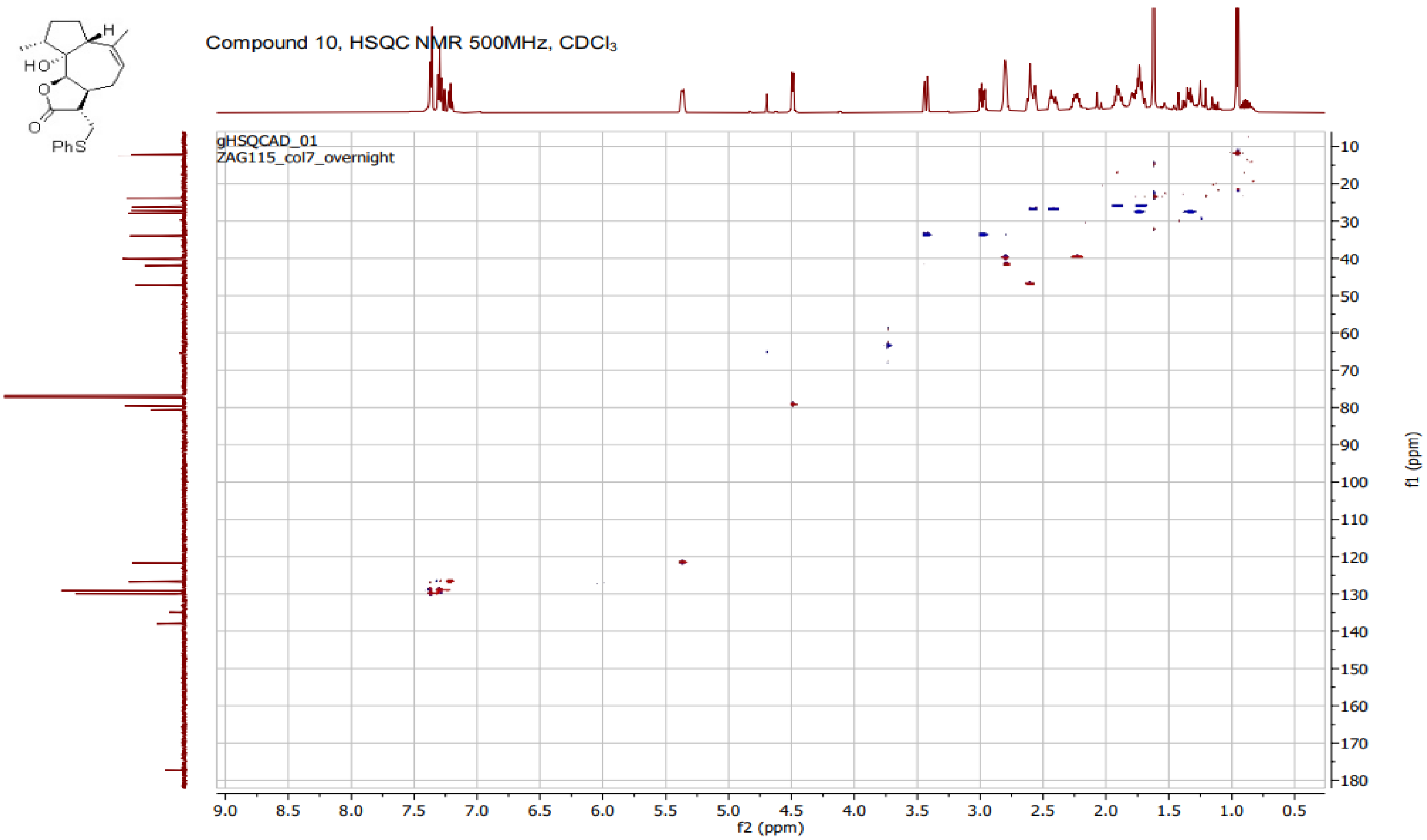
Compound 10, HSQC NMR 500MHz, CDCl3
HO
H
O
PhS
gHSQCAD_01
ZAG115_col7_overnight
f2 (ppm)
f1 (ppm)

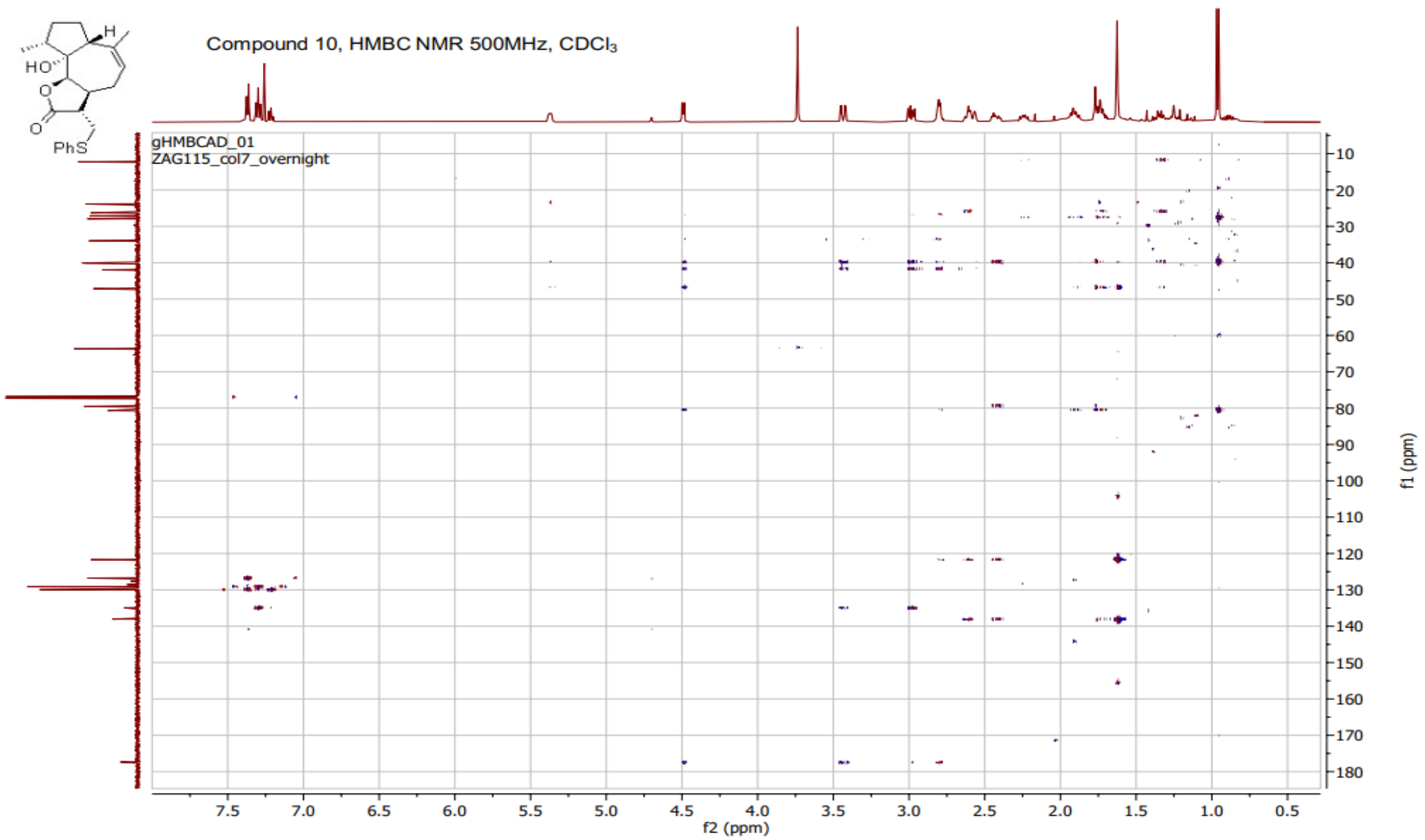
Compound 10, HMBC NMR 500MHz, CDCl3
gHMBCAD_01
ZAG115_col7_overnight
f2 (ppm)
f1 (ppm)

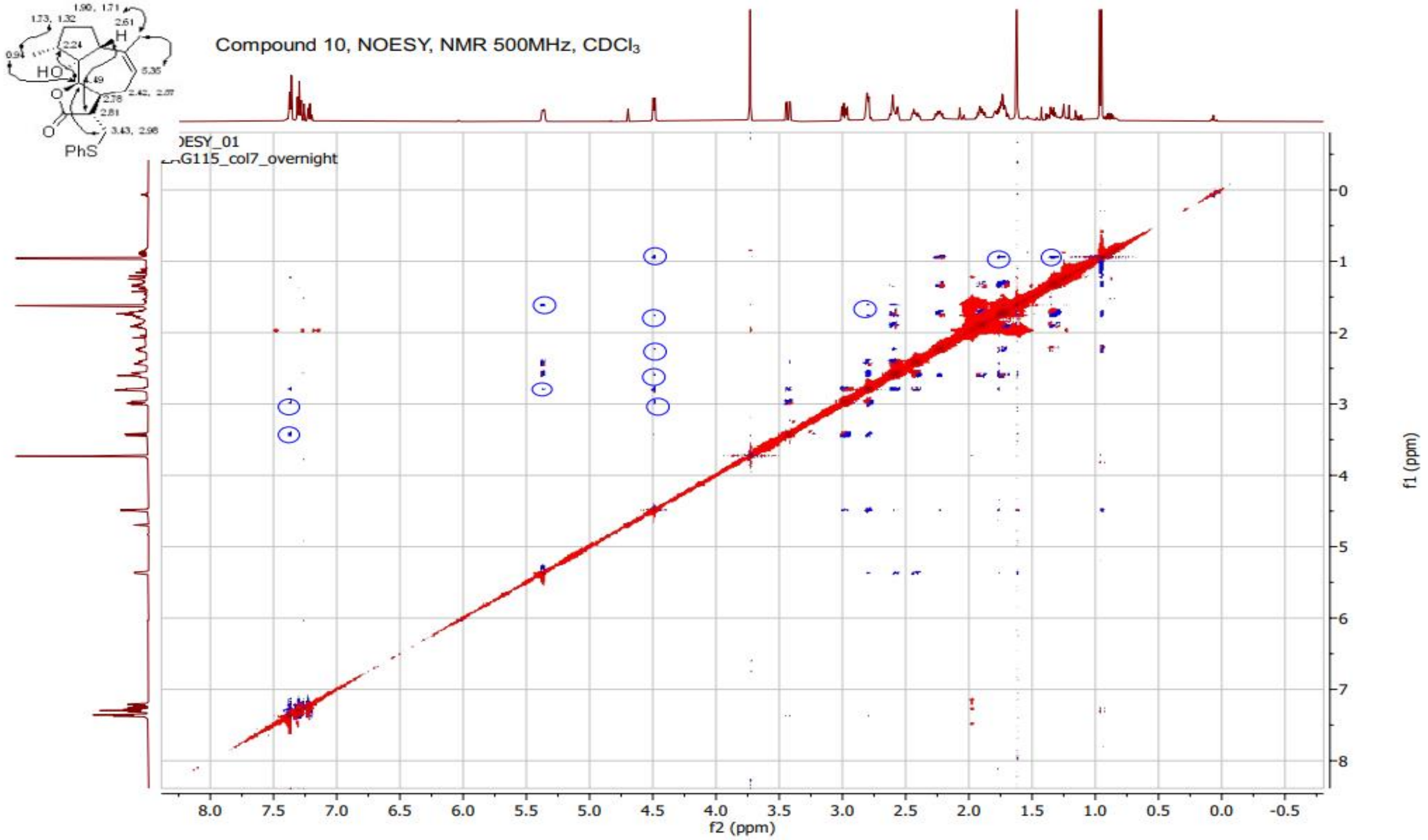
Compound 10, NOESY, NMR 500MHz, CDCl3
ZAG115_col7_overnight
f2 (ppm)
f1 (ppm)

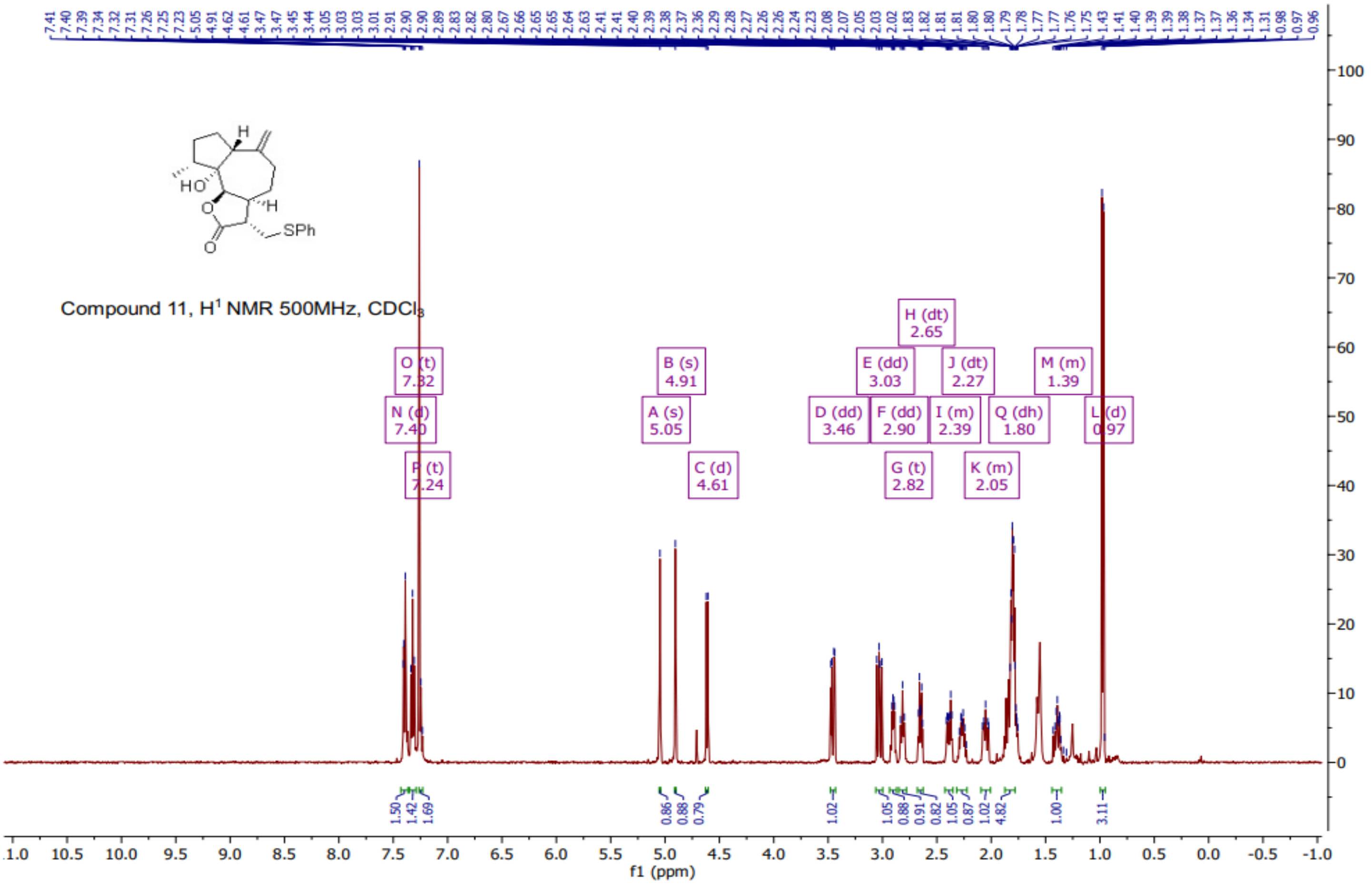

Compound 11, H[1] NMR 500MHz, CDCl3
f1 (ppm)


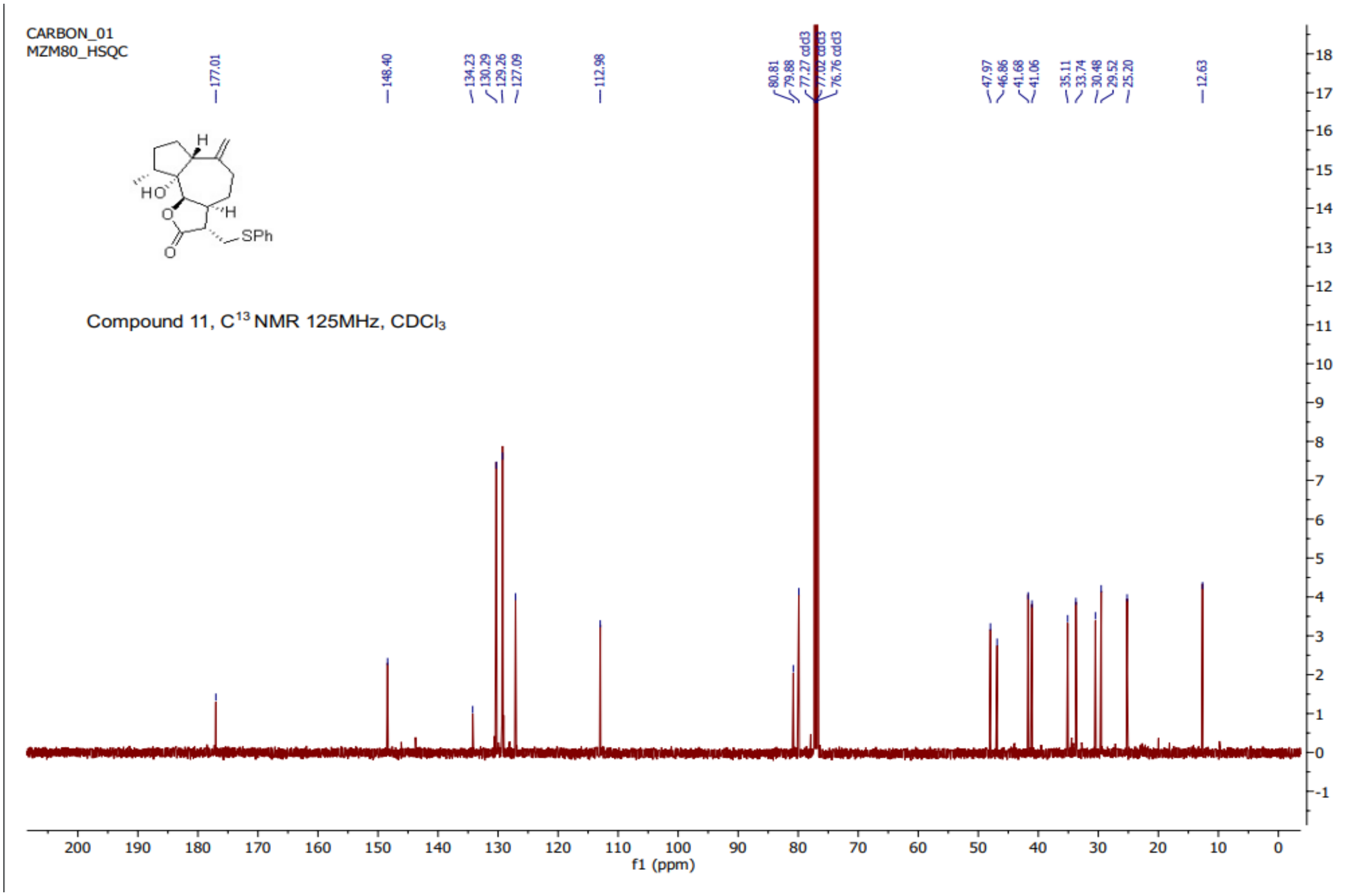

CARBON_01
MZM80_HSQC
Compound 11, C[13] NMR 125MHz, CDCl3
f1 (ppm)

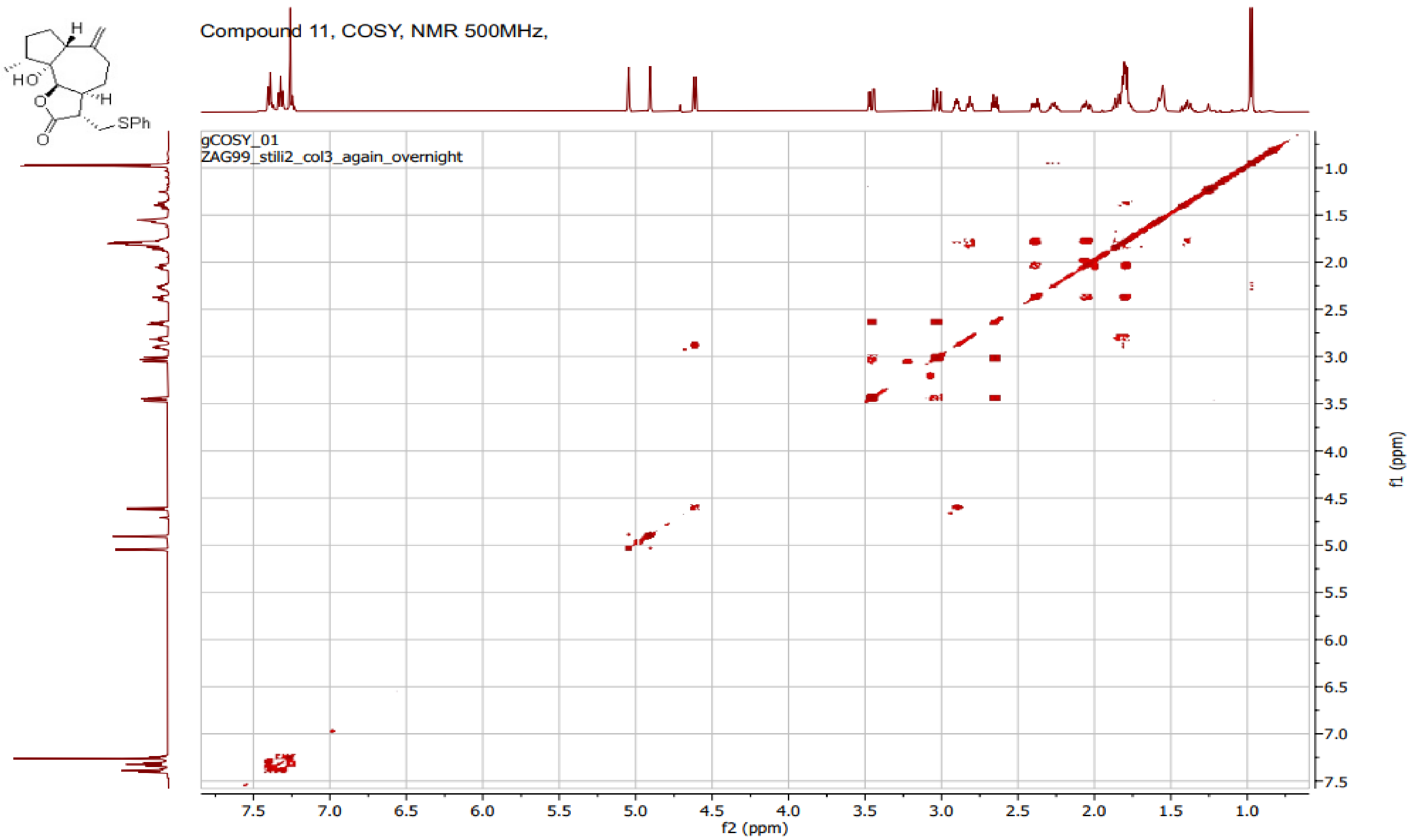
Compound 11, COSY, NMR 500MHz,
gCOSY_01
ZAG99_stili2_col3_again_overnight
f2 (ppm)
f1 (ppm)
HO
O
O
H
H
SPh

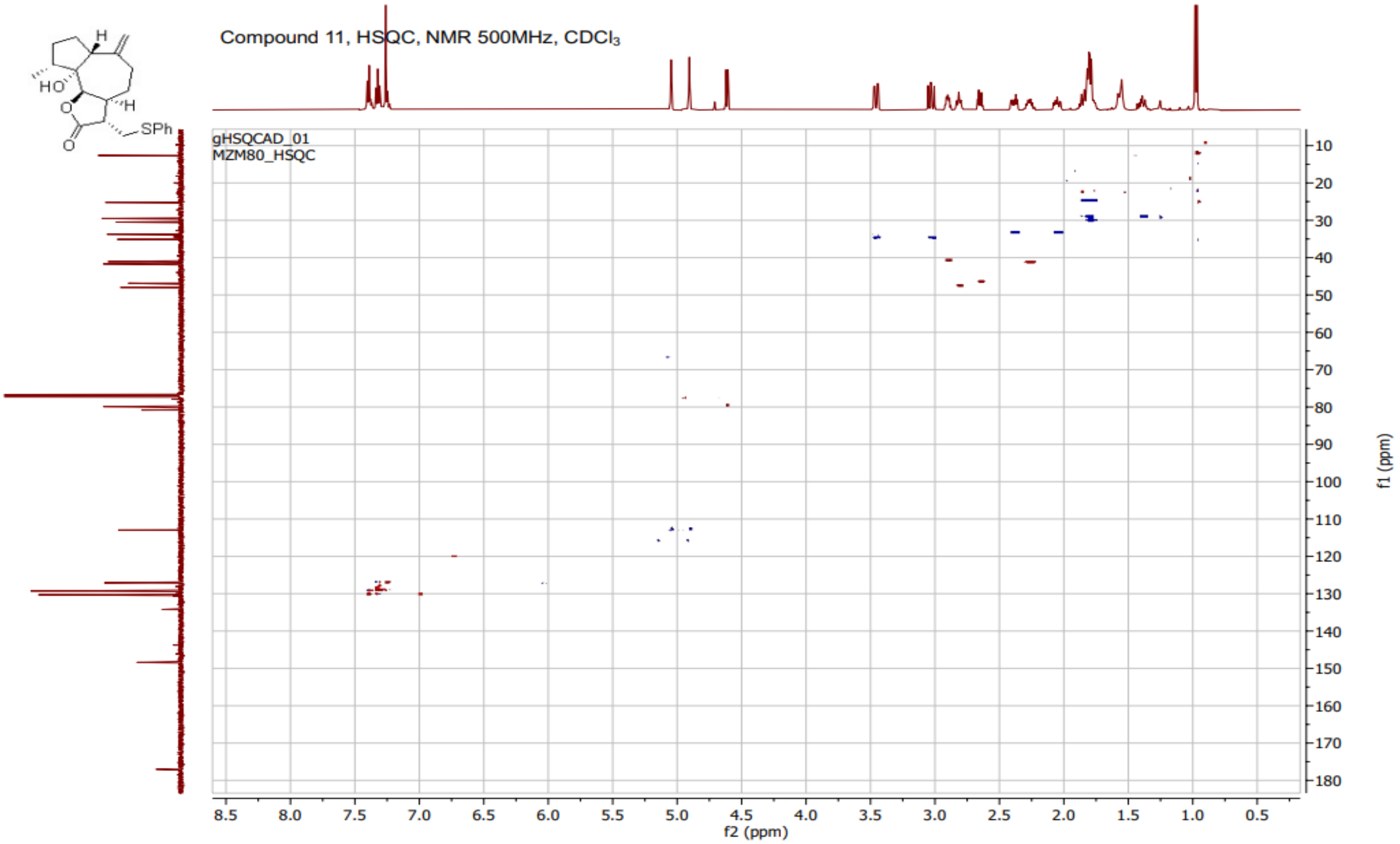
Compound 11, HSQC, NMR 500MHz, CDCl3
gHSQCAD_01
MZM80_HSQC
f2 (ppm)
f1 (ppm)
HO
O
O
H
H
SPh

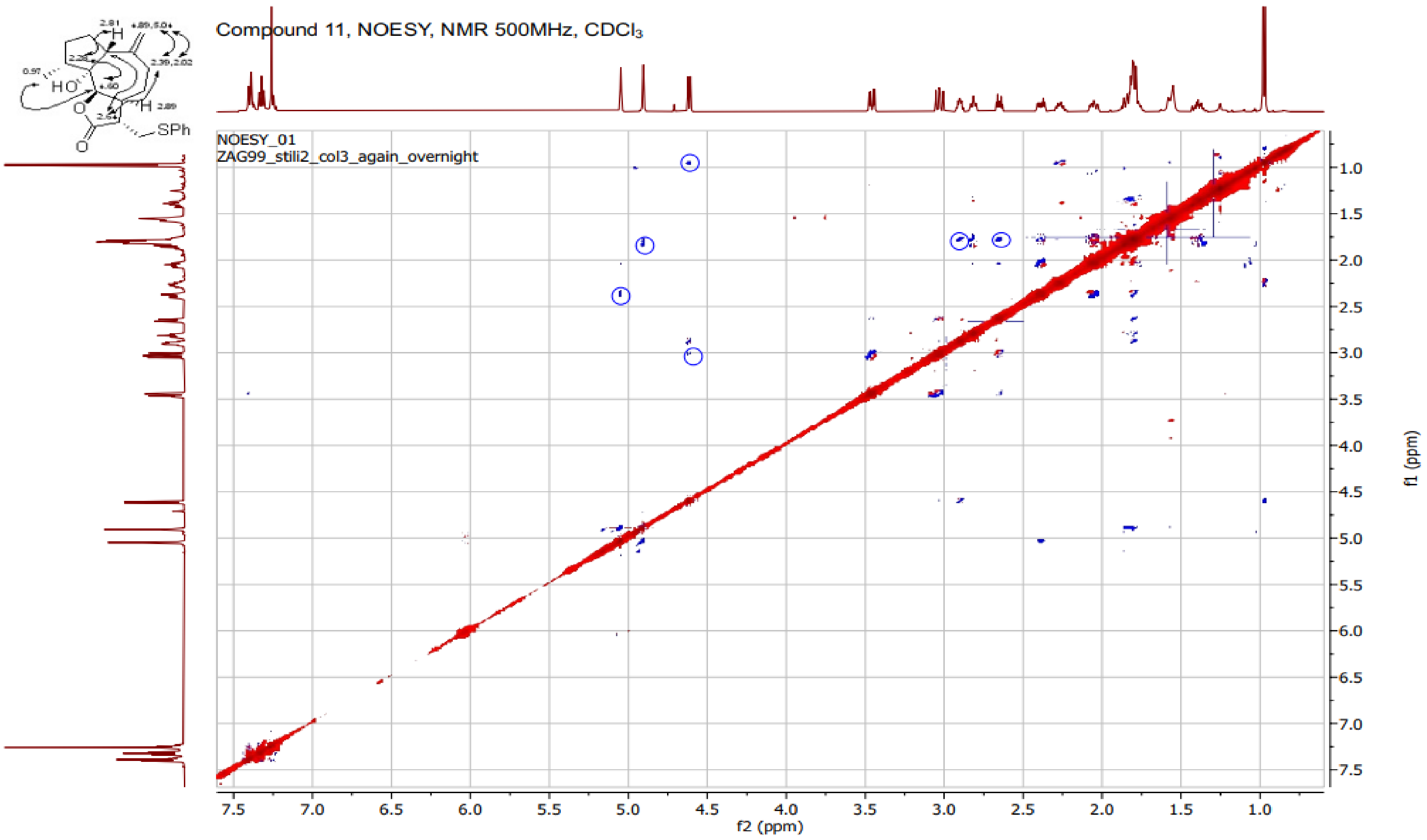
Compound 11, NOESY, NMR 500MHz, CDCl3
NOESY_01
ZAG99_stili2_col3_again_overnight
f2 (ppm)
f1 (ppm)

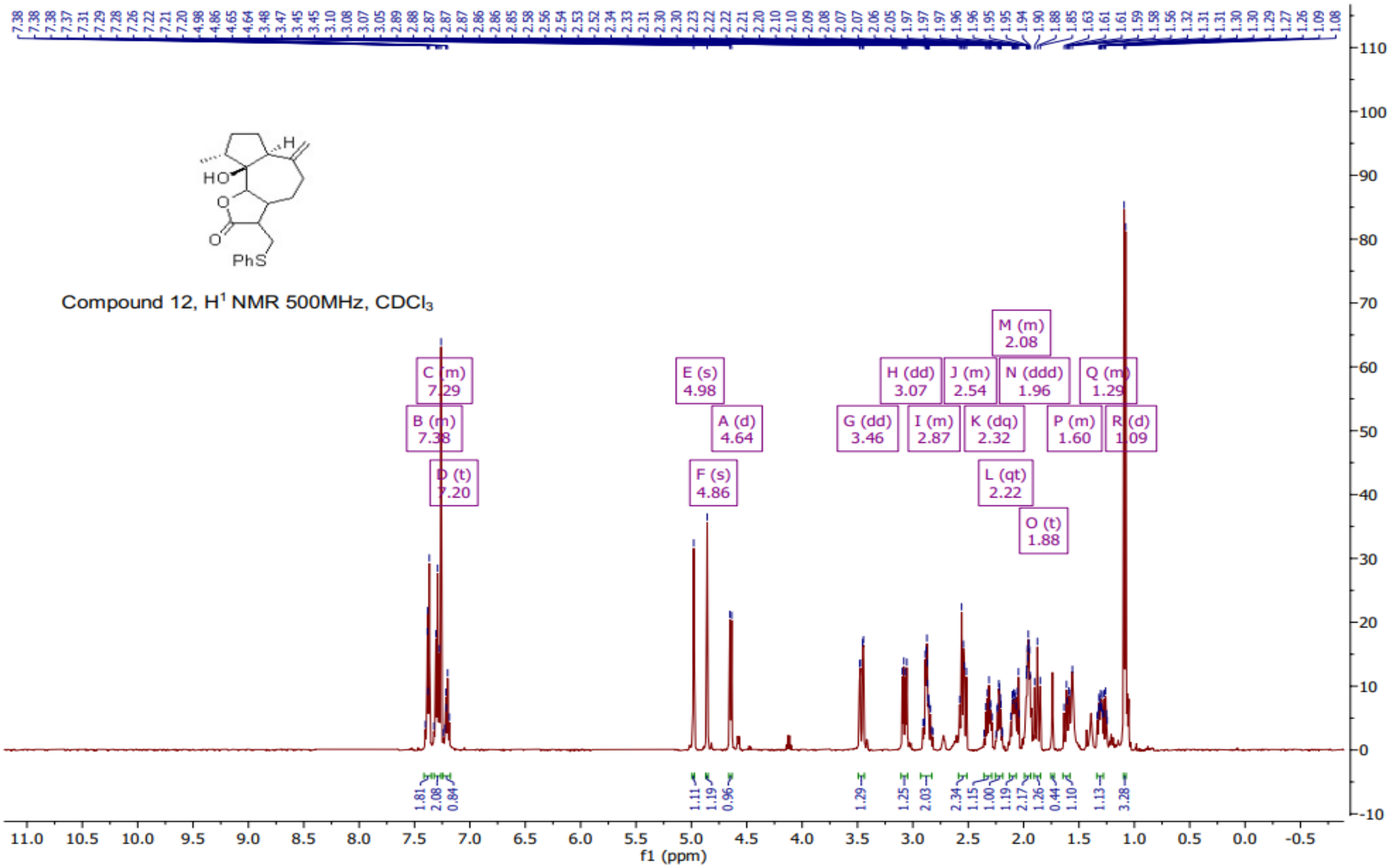
Compound 12, H1 NMR 500MHz, CDCl3
C (m) 7.29
B (m) 7.38
D (t) 7.20
E (s) 4.98
A (d) 4.64
F (s) 4.86
H (dd) 3.07
G (dd) 3.46
J (m) 2.54
I (m) 2.87
K (dq) 2.32
L (qt) 2.22
M (m) 2.08
N (ddd) 1.96
O (t) 1.88
P (m) 1.60
Q (m) 1.29
R (d) 1.09
f1 (ppm)

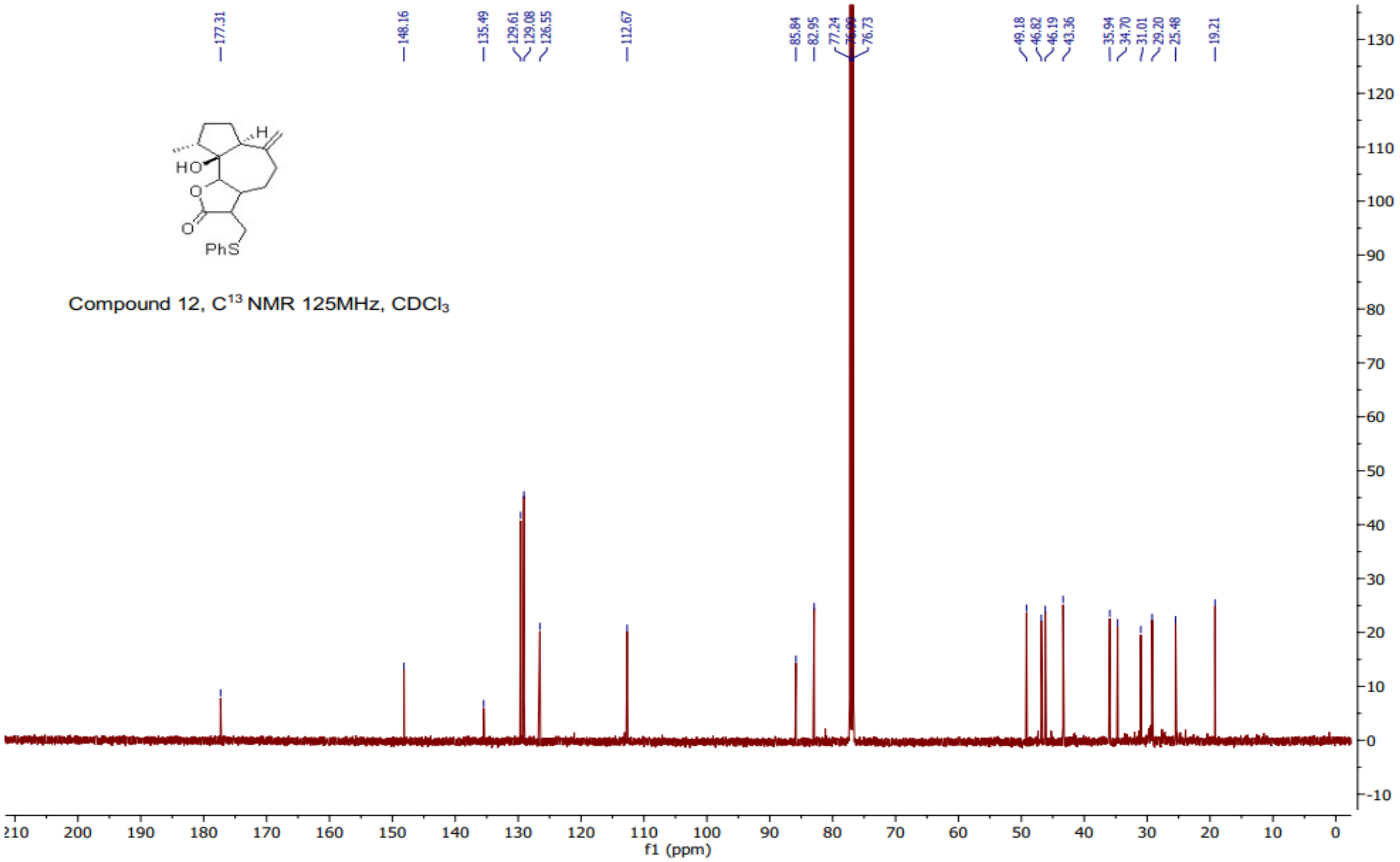
177.31
148.16
135.49
129.61
129.08
126.55
112.67
85.84
82.95
77.24
76.99
76.73
49.18
46.82
46.19
43.36
35.94
34.70
31.01
29.20
25.48
19.21
HO
O
O
PhS
H
Compound 12, C13 NMR 125MHz, CDCl3
f1 (ppm)

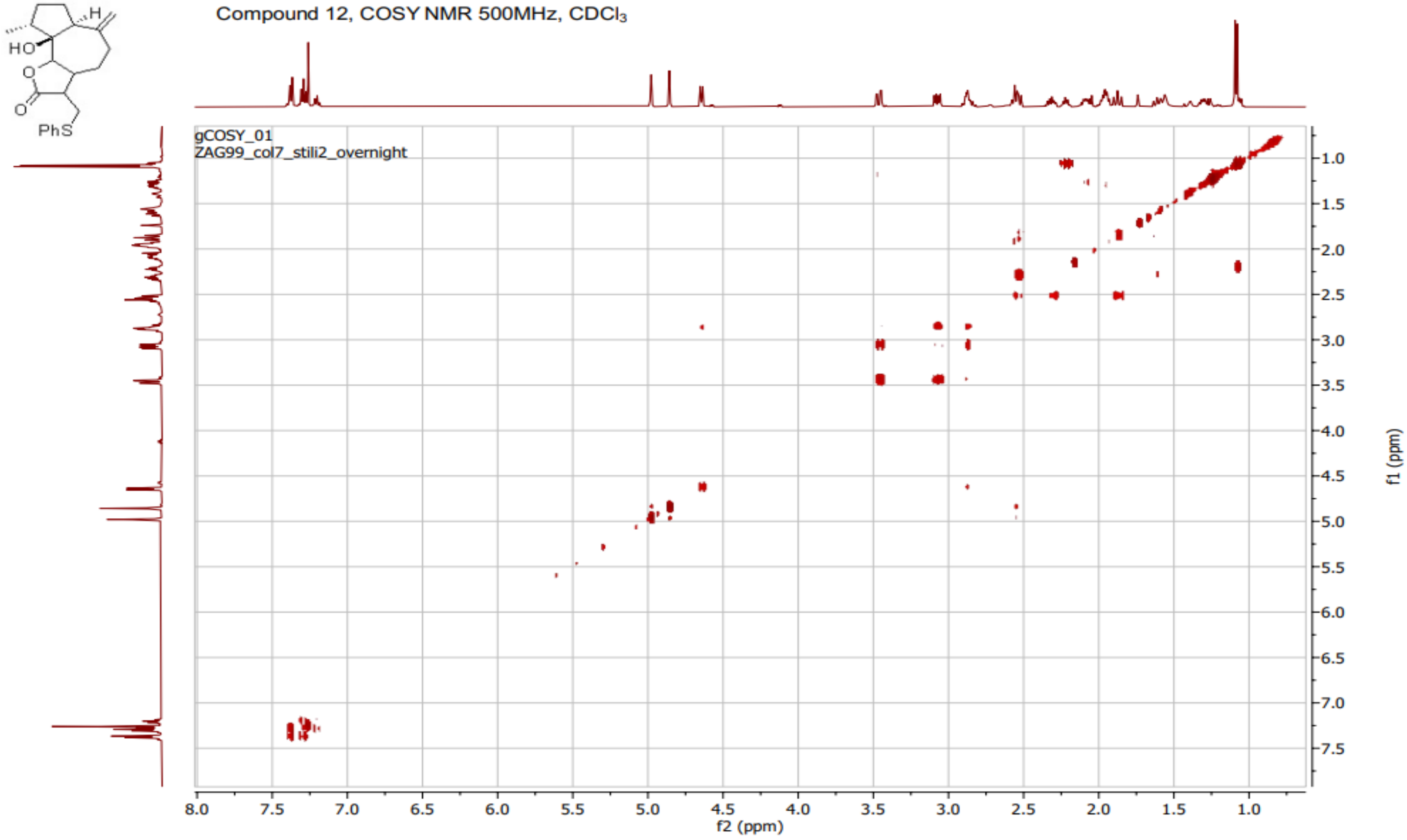
HO
O
O
PhS
H
Compound 12, COSY NMR 500MHz, CDCl3
gCOSY_01
ZAG99_col7_stili2_overnight
f2 (ppm)
f1 (ppm)

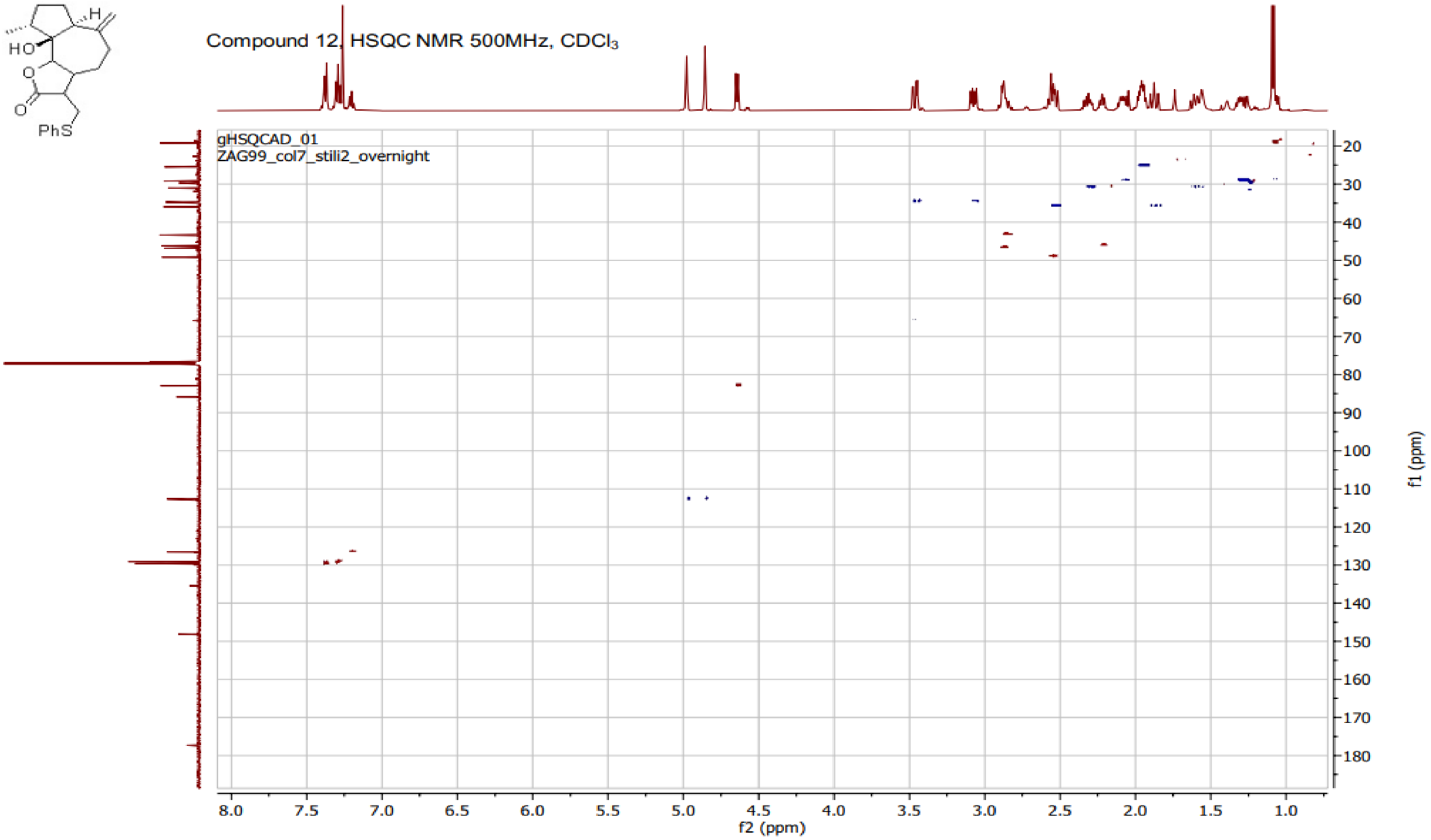
Compound 12, HSQC NMR 500MHz, $CDCl_3$
gHSQCAD_01
ZAG99_col7_stili2_overnight
f2 (ppm)
f1 (ppm)

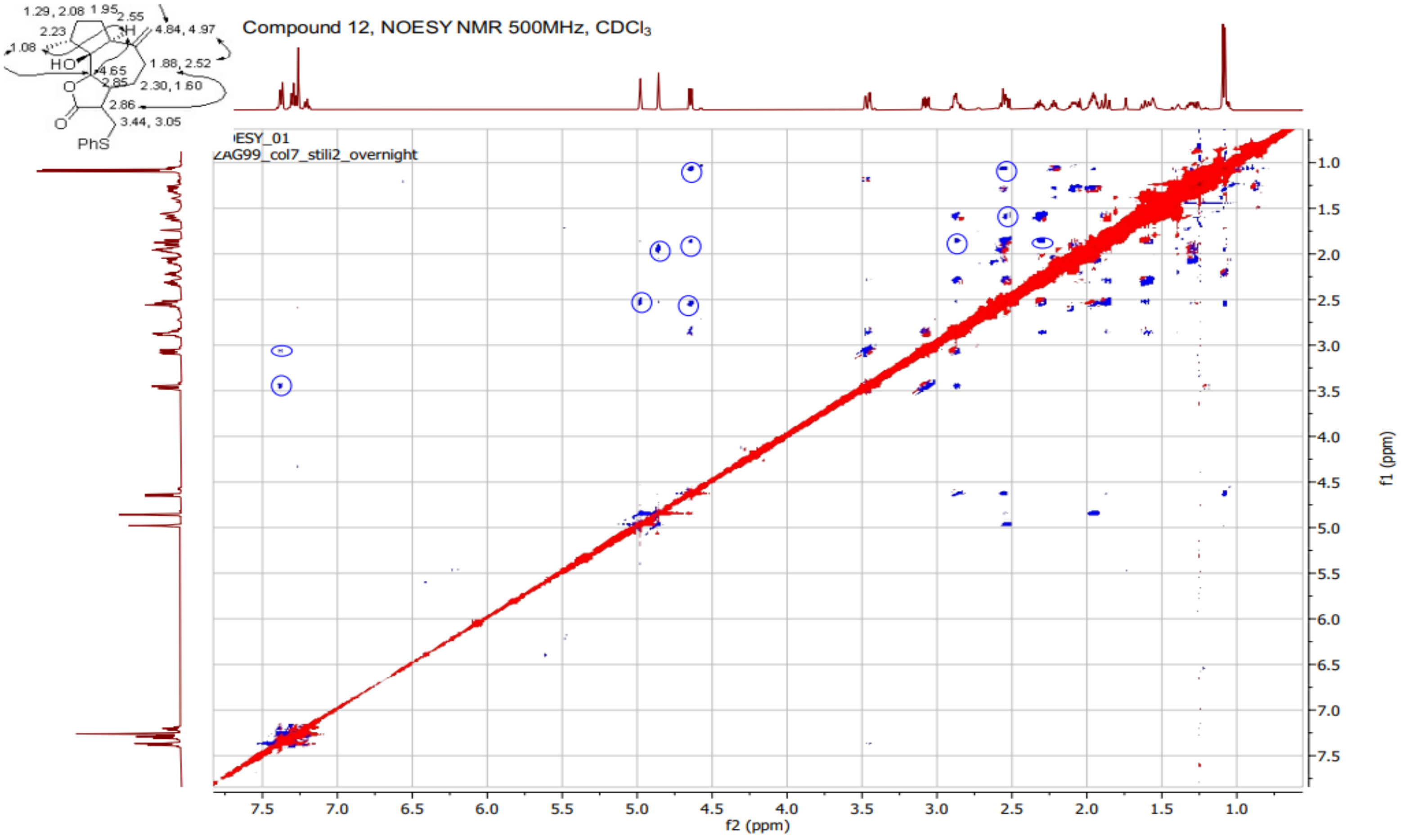
Compound 12, NOESY NMR 500MHz, $CDCl_3$
ZAG99_col7_stili2_overnight
f2 (ppm)
f1 (ppm)

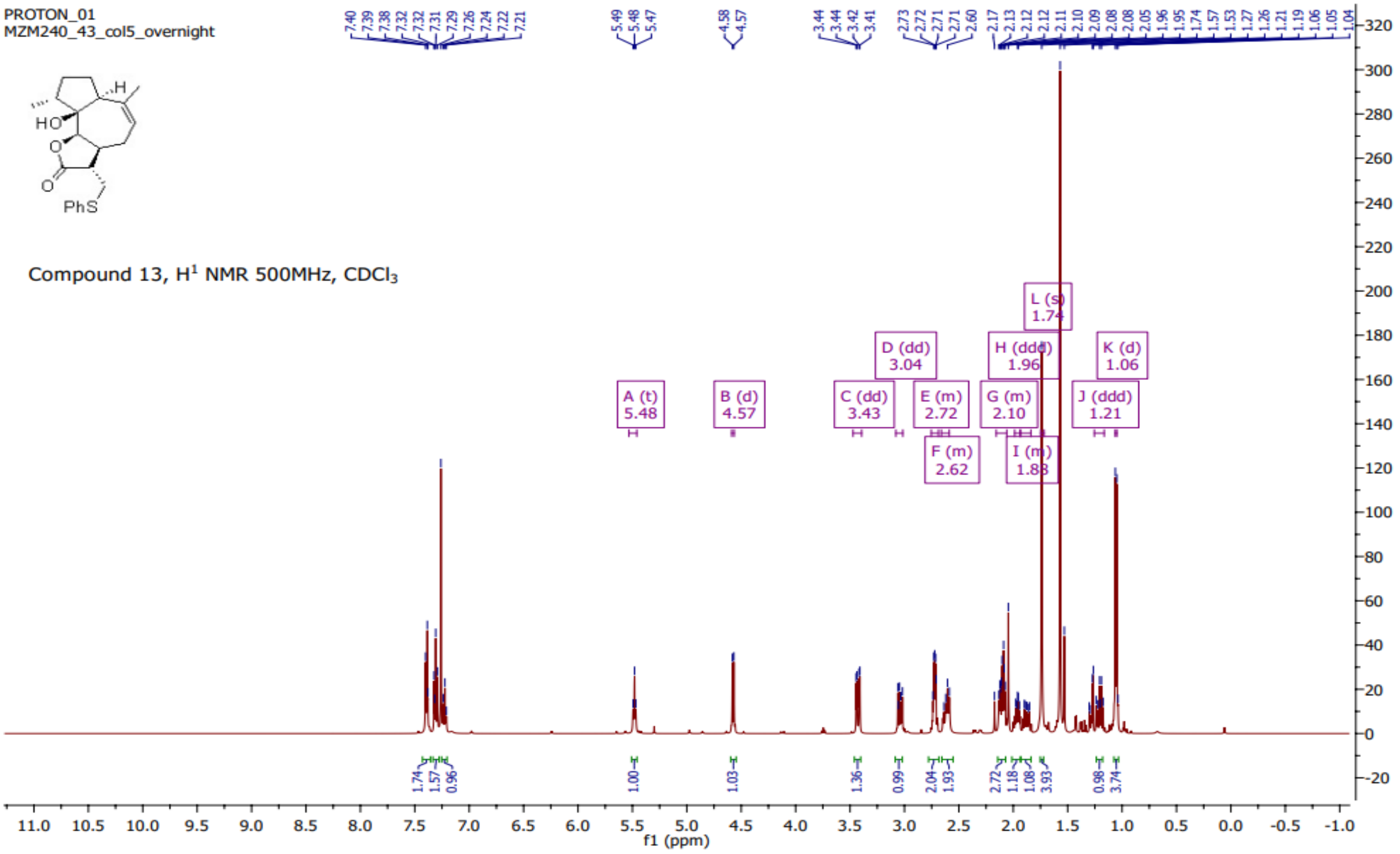
PROTON_01
MZM240_43_col5_overnight
Compound 13, H1 NMR 500MHz, CDCl3
f1 (ppm)

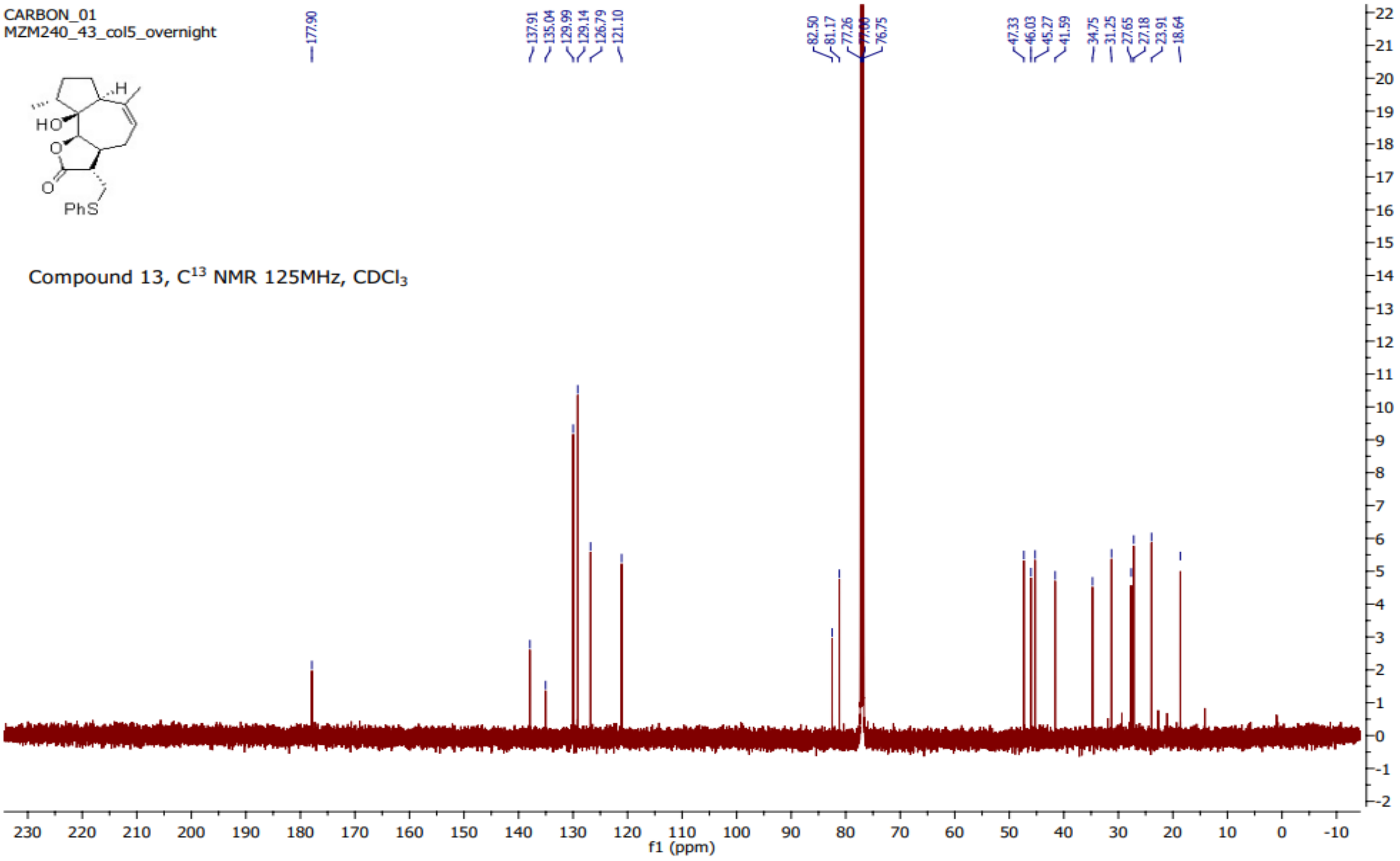
CARBON_01
MZM240_43_col5_overnight
Compound 13, C13 NMR 125MHz, CDCl3
177.90
137.91
135.04
129.99
129.14
126.79
121.10
82.50
81.17
77.26
77.00
76.75
47.33
46.03
45.27
41.59
34.75
31.25
27.65
27.18
23.91
18.64
f1 (ppm)

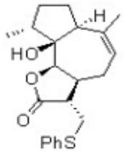

Compound 13, COSY, NMR 500MHz, $CDCl_3$

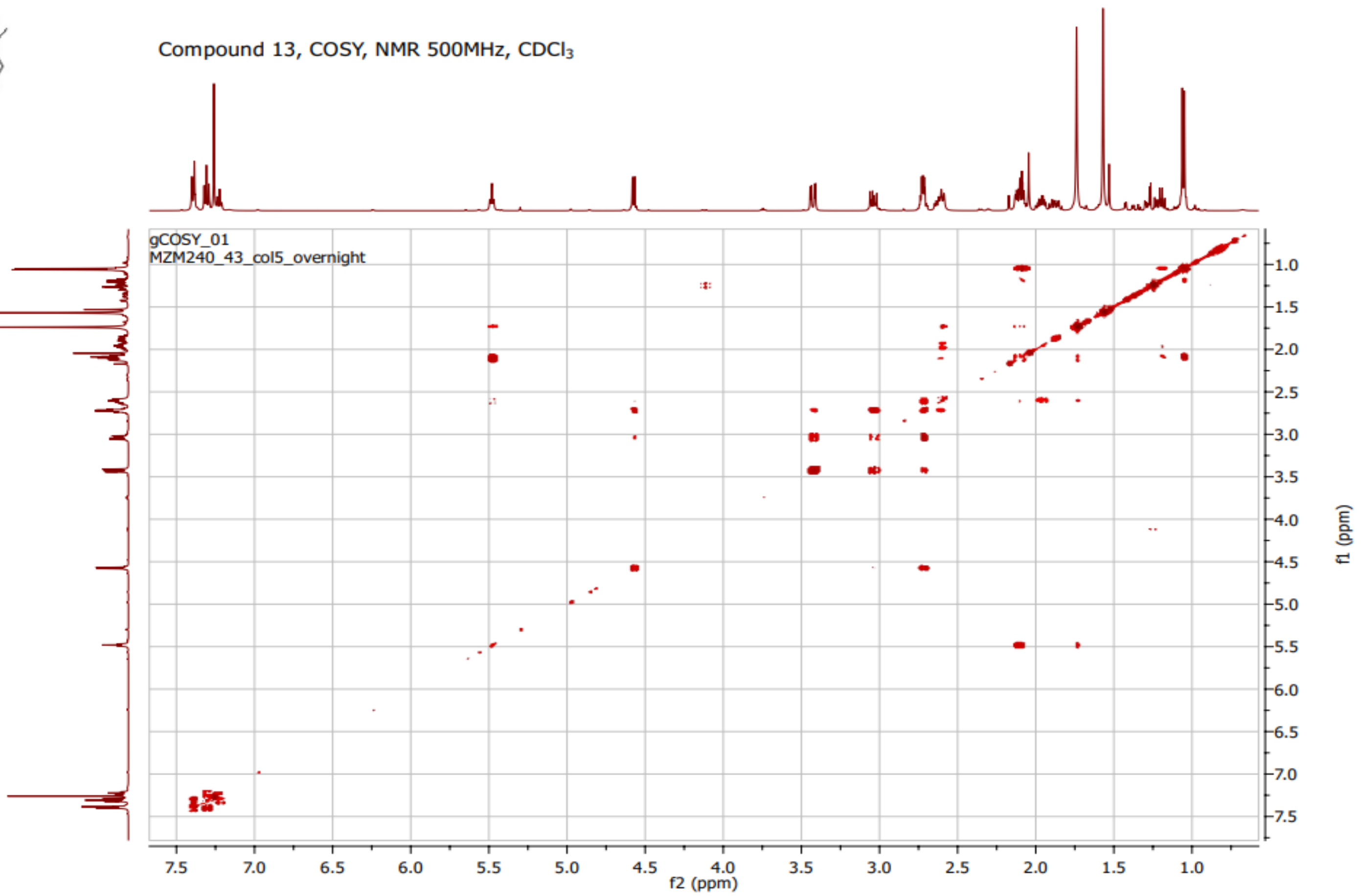


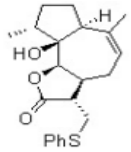

Compound 13, HSQCAD, NMR 500MHz, $CDCl_3$

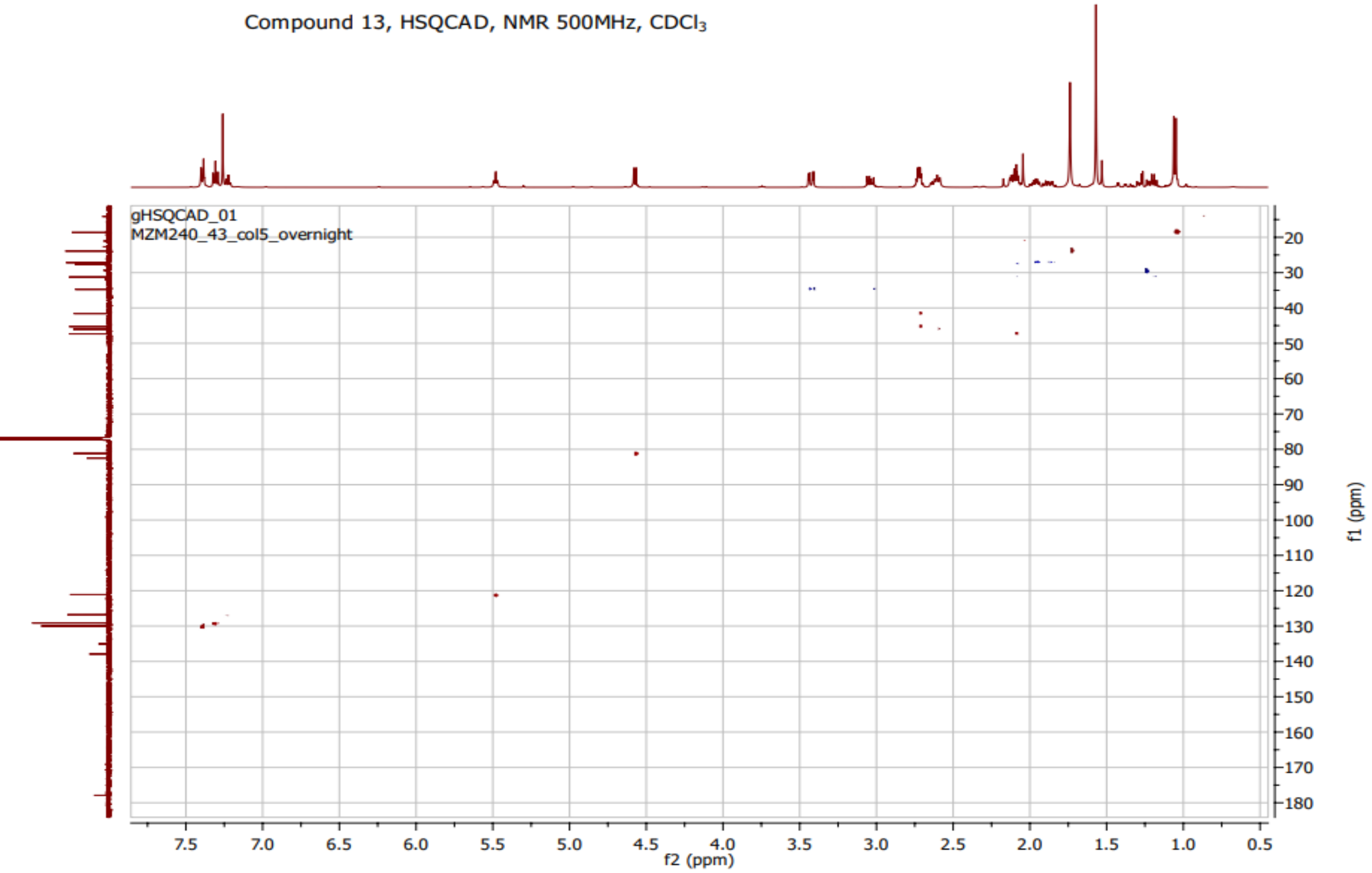

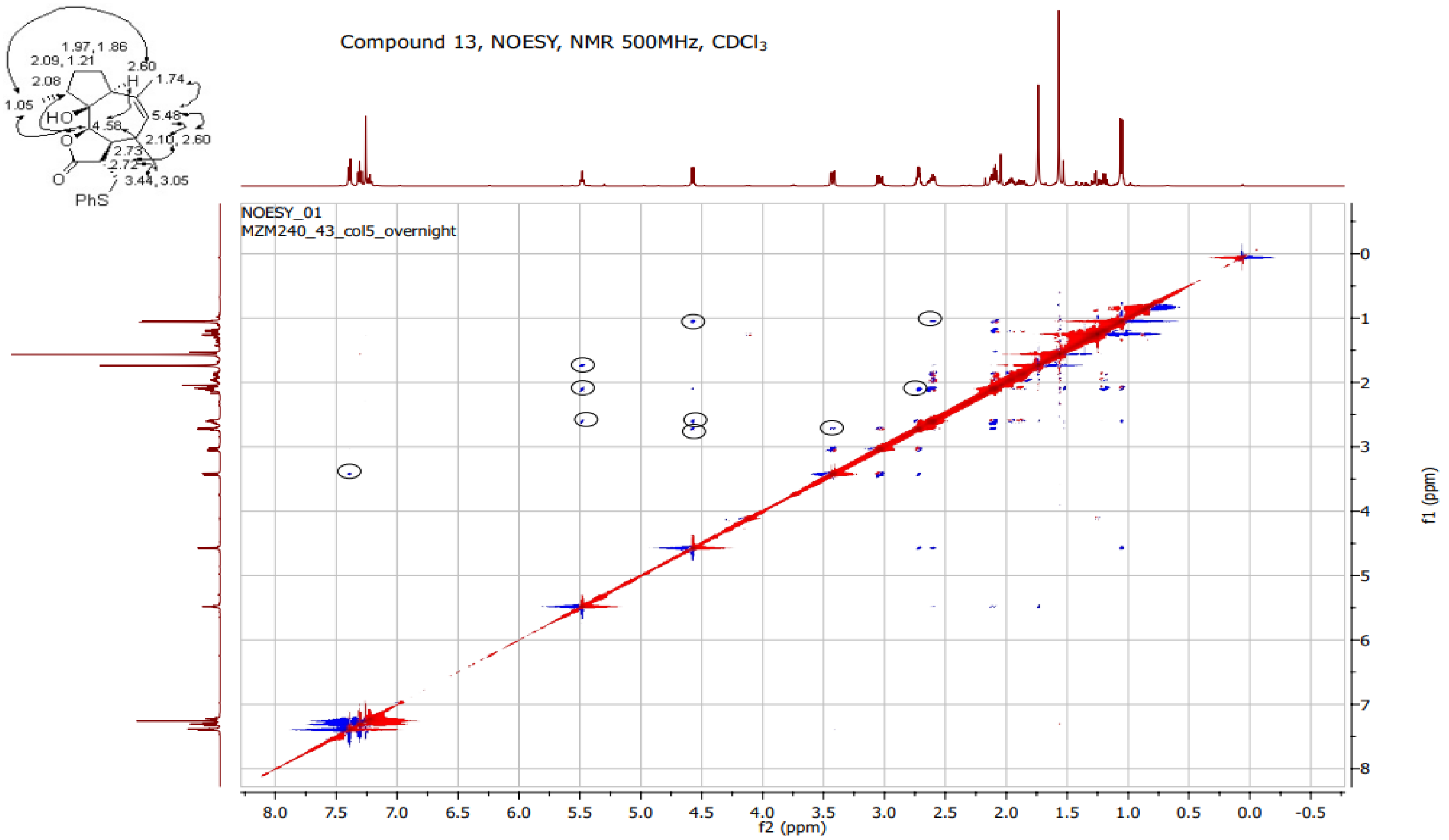

Compound 13, NOESY, NMR 500MHz, $CDCl_3$
NOESY_01
MZM240_43_col5_overnight
f2 (ppm)
f1 (ppm)
PhS
HO


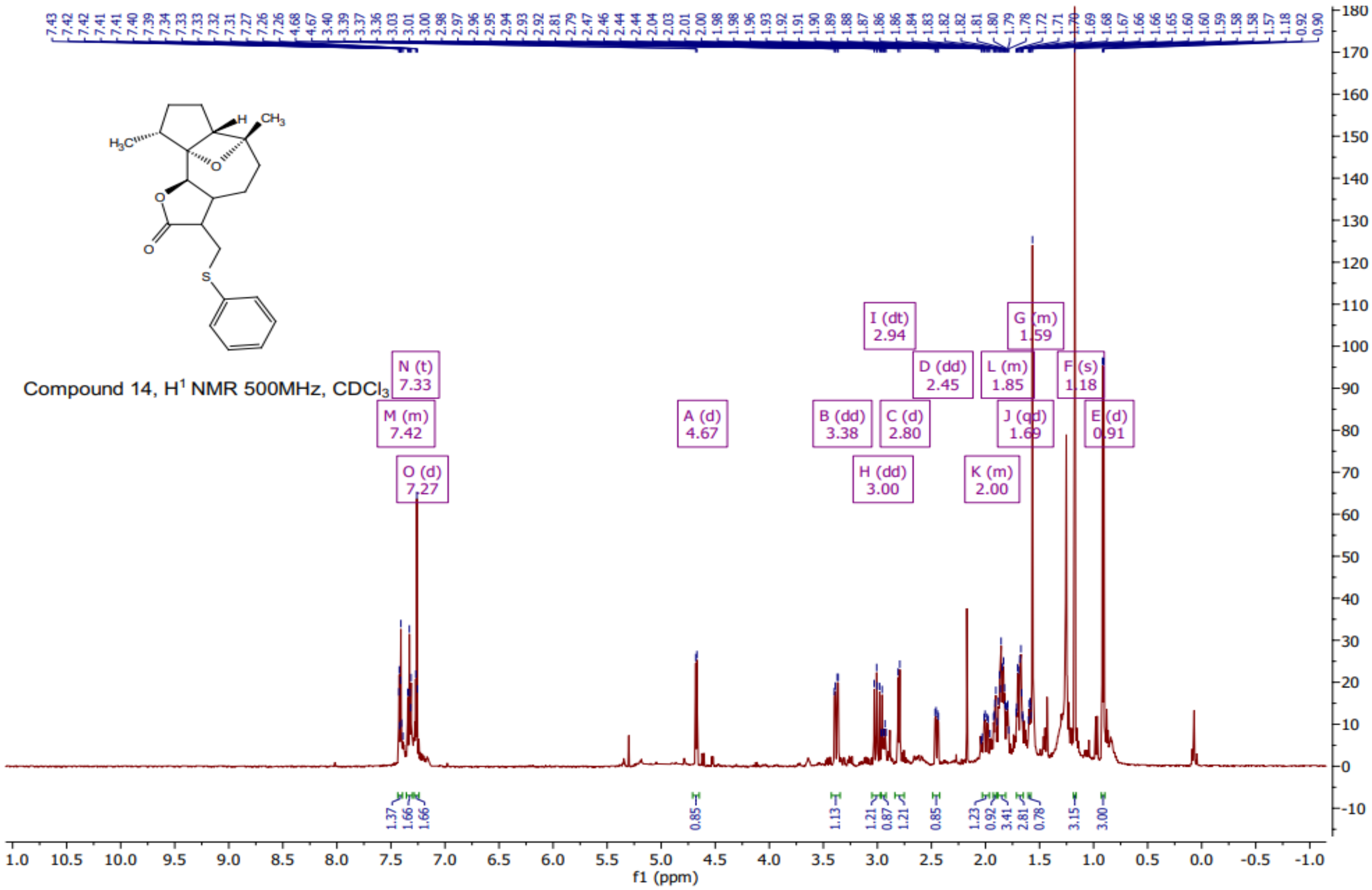

Compound 14, H[1] NMR 500MHz, $CDCl_3$
N (t) 7.33
M (m) 7.42
O (d) 7.27
A (d) 4.67
B (dd) 3.38
C (d) 2.80
H (dd) 3.00
I (dt) 2.94
D (dd) 2.45
K (m) 2.00
L (m) 1.85
J (qd) 1.69
G (m) 1.59
F (s) 1.18
E (d) 0.91
f1 (ppm)

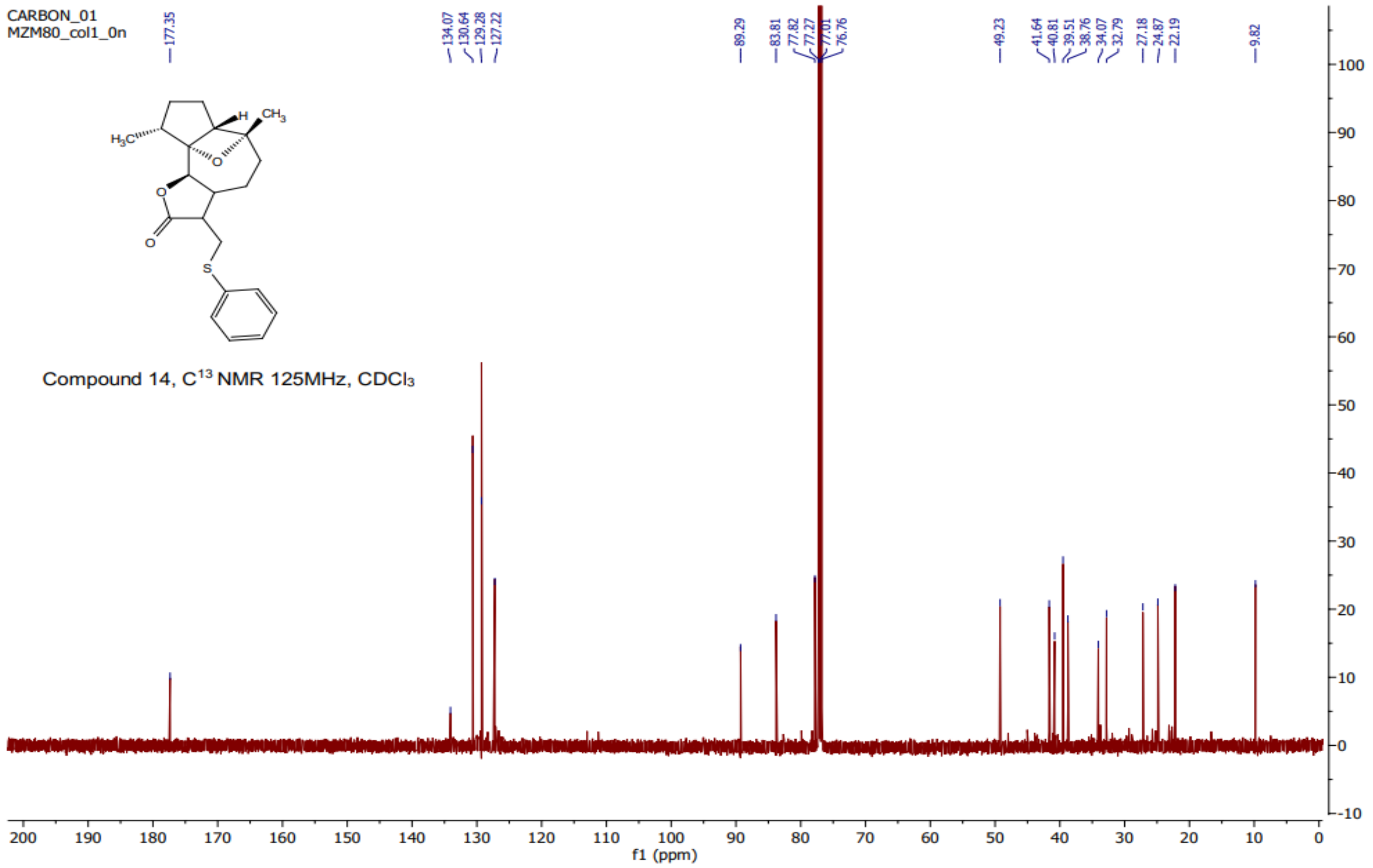
CARBON_01
MZM80_col1_0n
177.35
134.07
130.64
129.28
127.22
89.29
83.81
77.82
77.27
77.01
76.76
49.23
41.64
40.81
39.51
38.76
34.07
32.79
27.18
24.87
22.19
9.82
Compound 14, C13 NMR 125MHz, CDCl3
f1 (ppm)

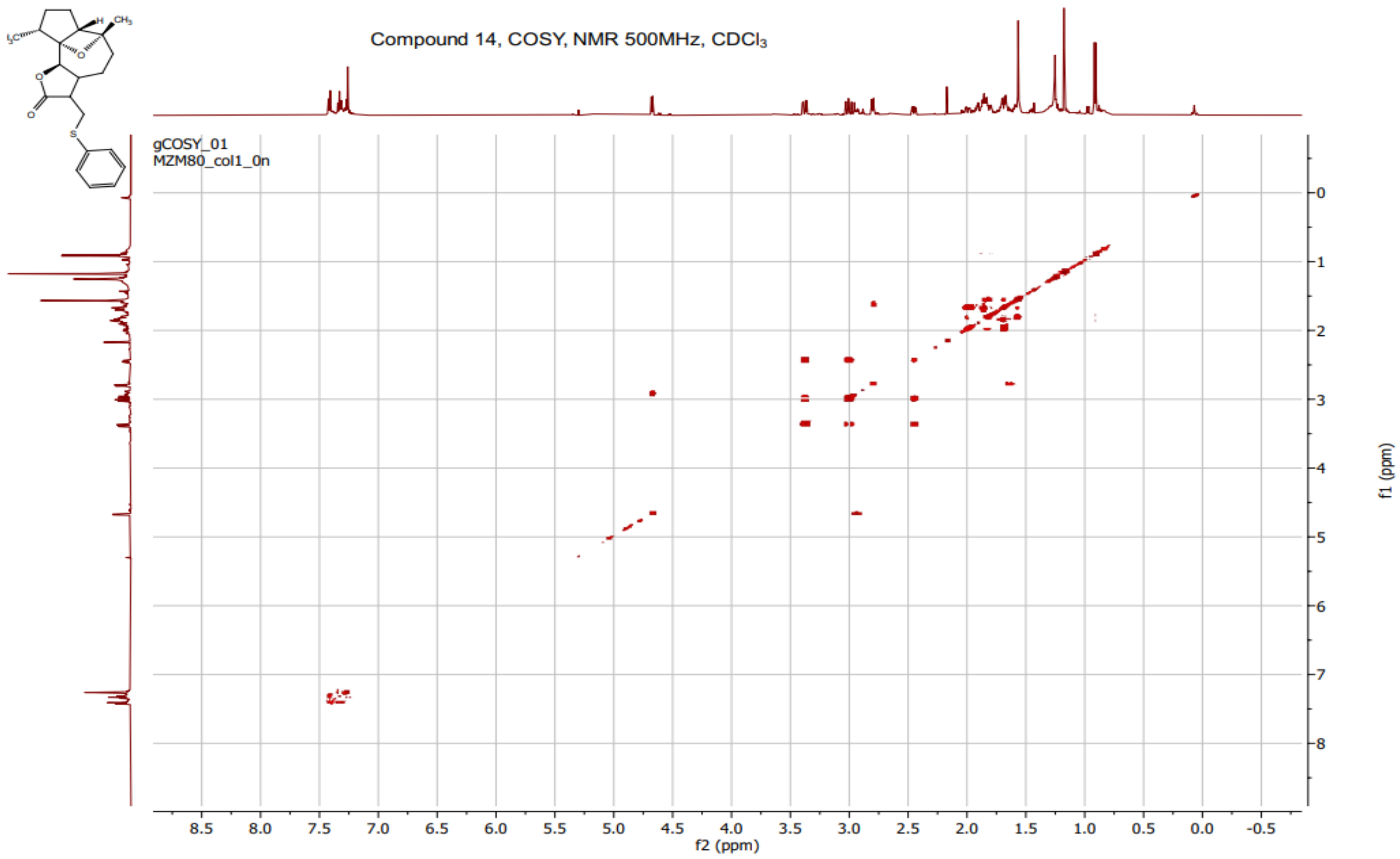
Compound 14, COSY, NMR 500MHz, CDCl3
gCOSY_01
MZM80_col1_0n
f2 (ppm)
f1 (ppm)

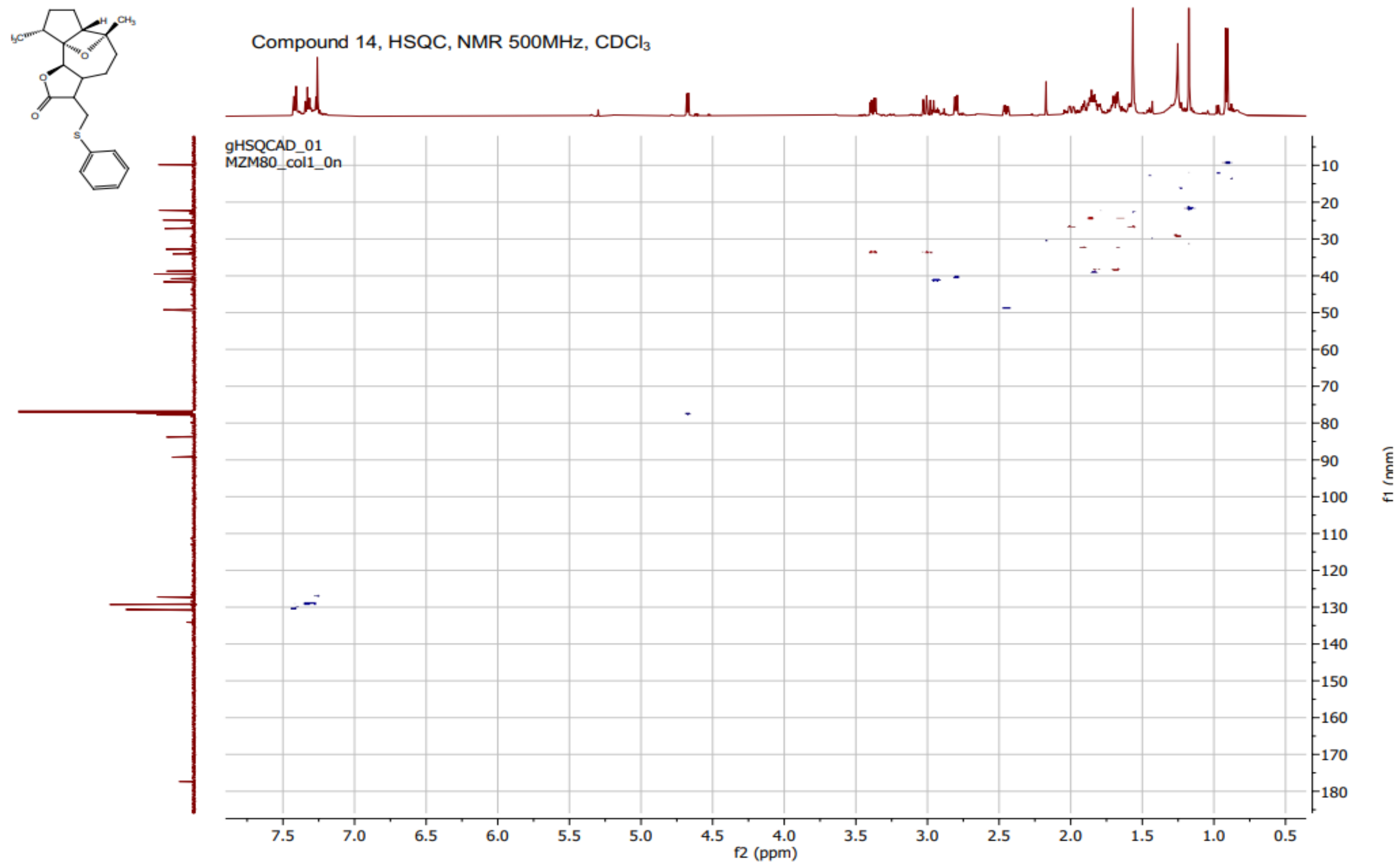
Compound 14, HSQC, NMR 500MHz, CDCl3
gHSQCAD_01
MZM80_col1_0n
f2 (ppm)
f1 (ppm)

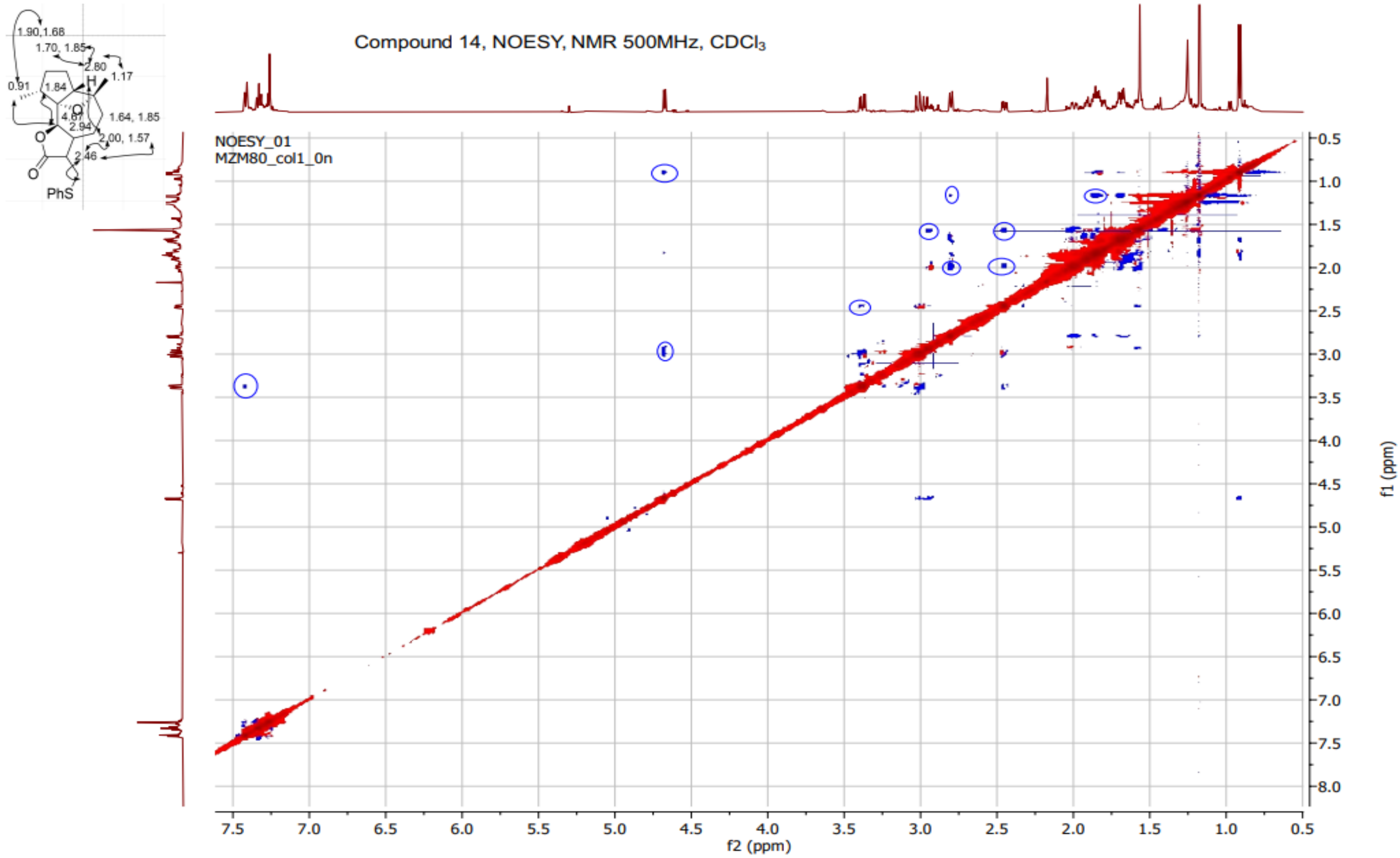
Compound 14, NOESY, NMR 500MHz, CDCl3
NOESY_01
MZM80_col1_0n
PhS
f2 (ppm)
f1 (ppm)

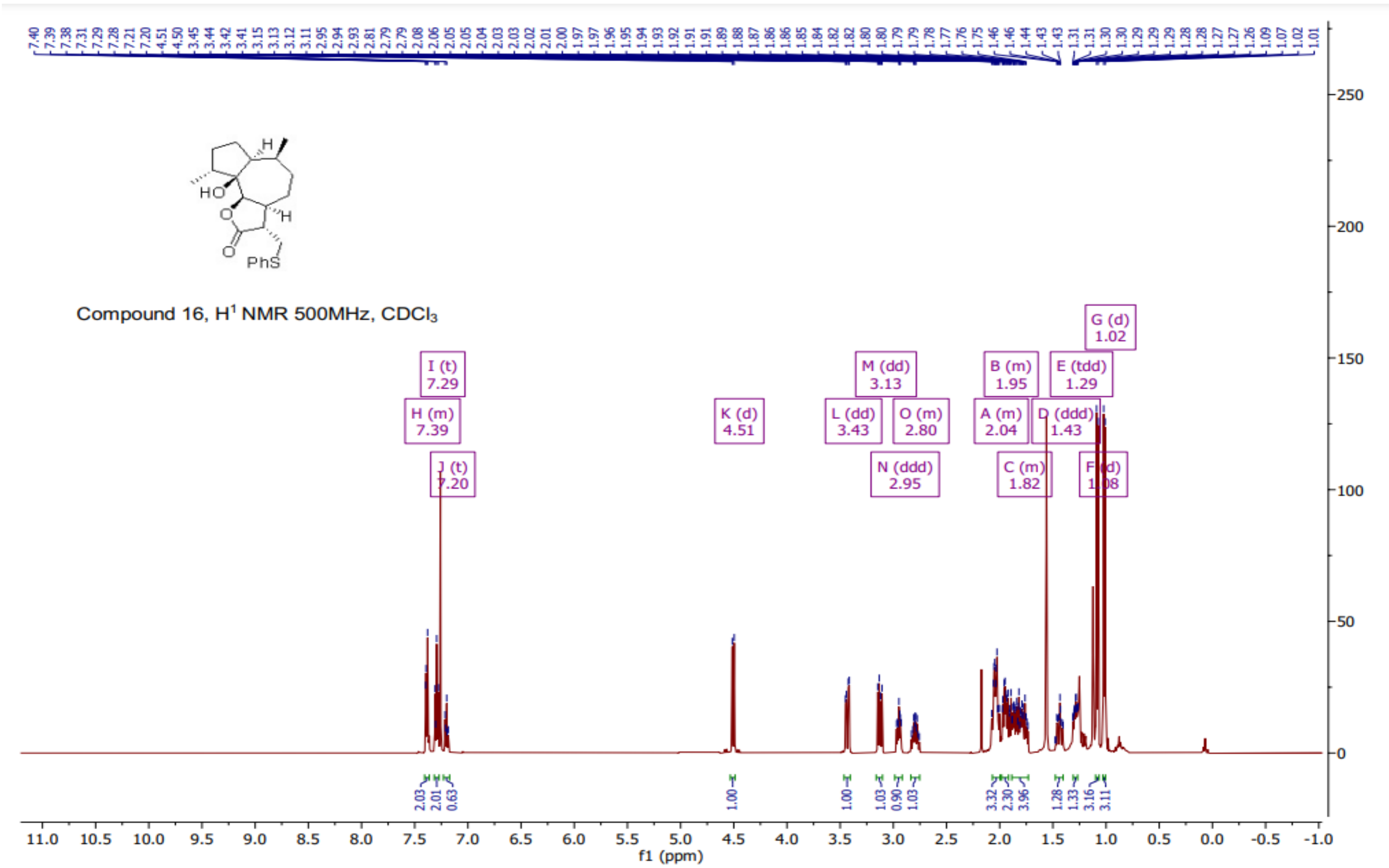

HO
H
H
O
O
PhS
Compound 16, H[1] NMR 500MHz, CDCl3
I (t) 7.29
H (m) 7.39
J (t) 7.20
K (d) 4.51
M (dd) 3.13
L (dd) 3.43
O (m) 2.80
N (ddd) 2.95
B (m) 1.95
A (m) 2.04
C (m) 1.82
E (tdd) 1.29
D (ddd) 1.43
G (d) 1.02
F (d) 1.08
f1 (ppm)


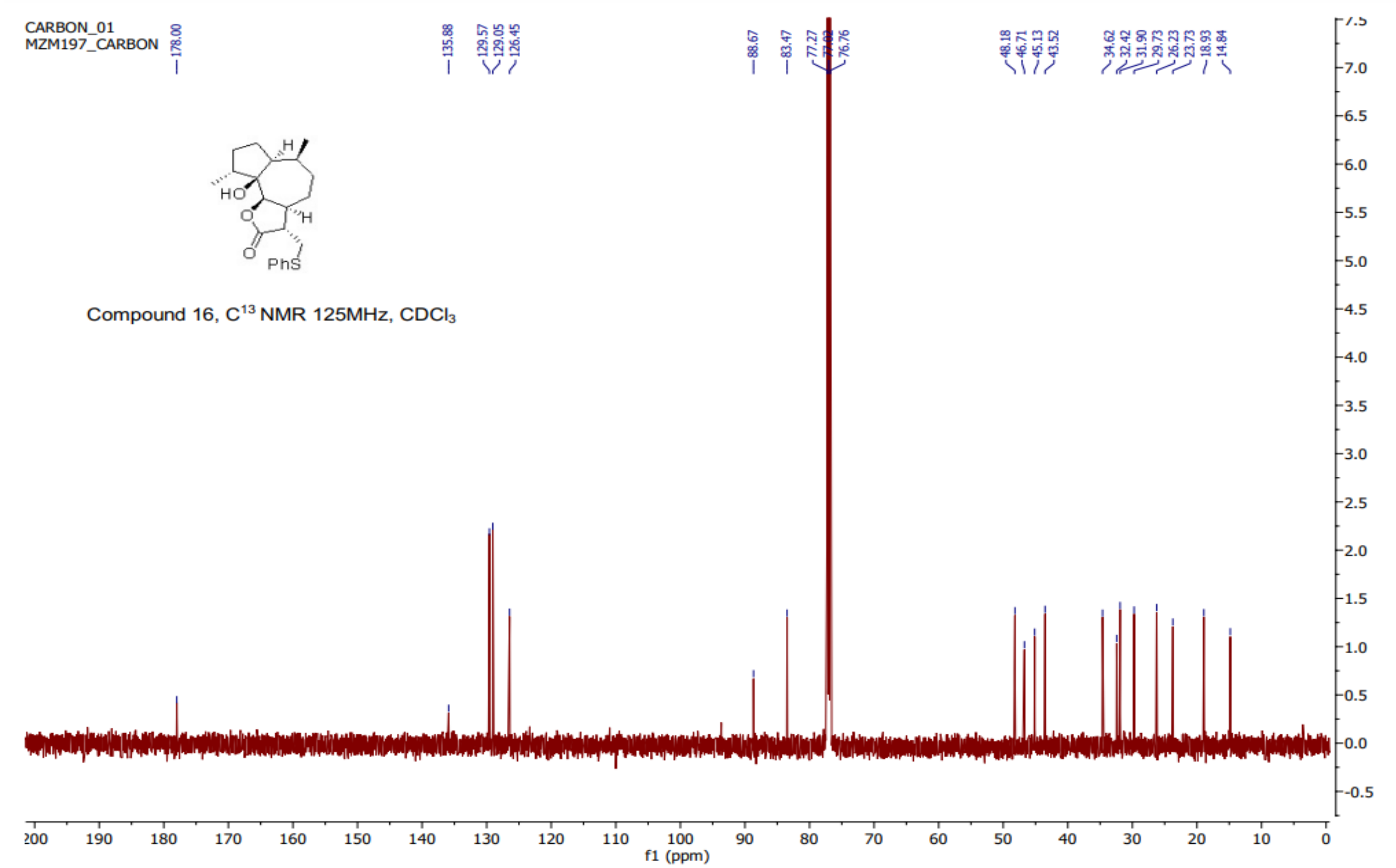

CARBON_01
MZM197_CARBON
178.00
135.88
129.57
129.05
126.45
88.67
83.47
77.27
76.76
48.18
46.71
45.13
43.52
34.62
32.42
31.90
29.73
26.23
23.73
18.93
14.84
HO
H
H
O
O
PhS
Compound 16, C[13] NMR 125MHz, CDCl3
f1 (ppm)

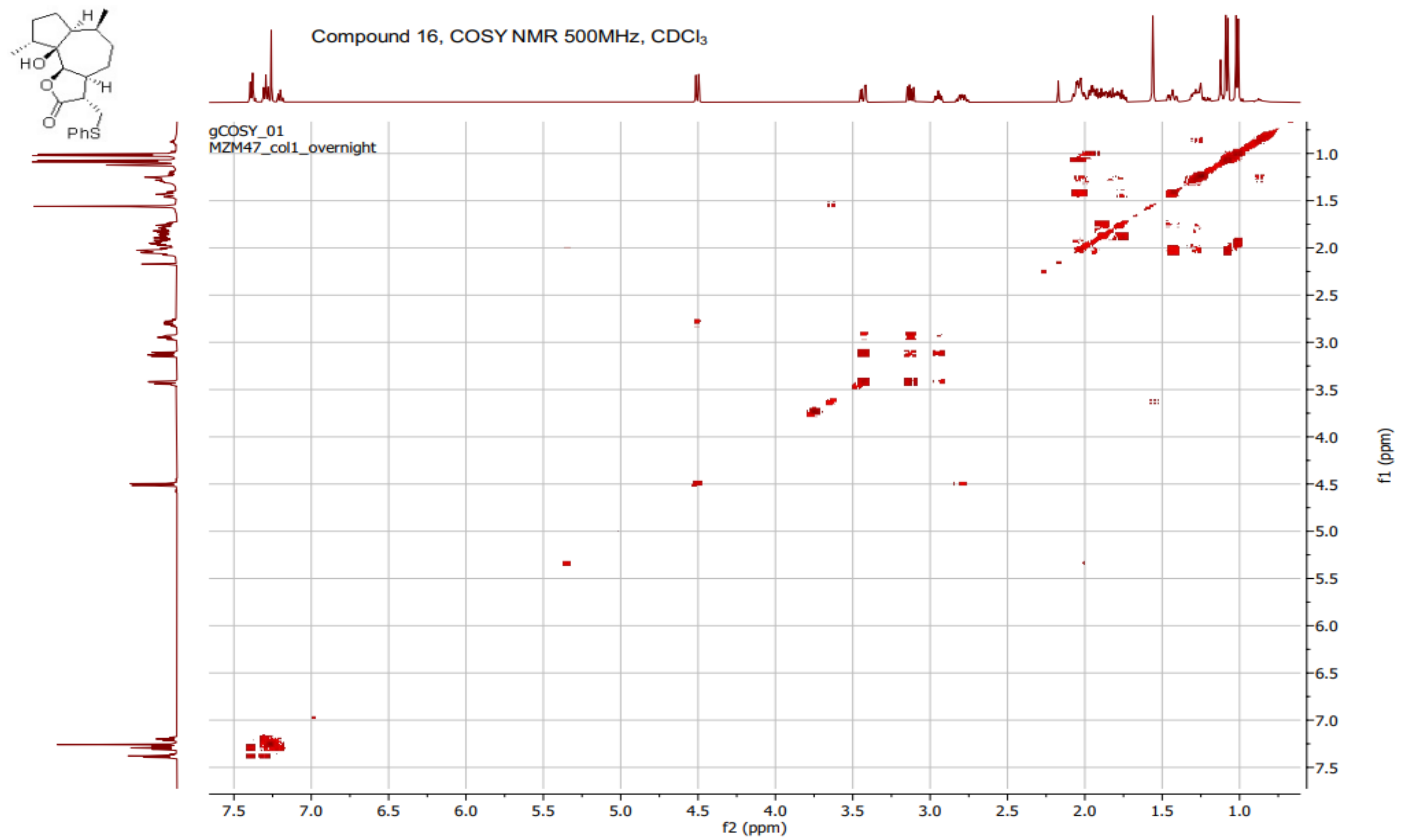
Compound 16, COSY NMR 500MHz, CDCl3
gCOSY_01
MZM47_col1_overnight
f2 (ppm)
f1 (ppm)
HO
PhS

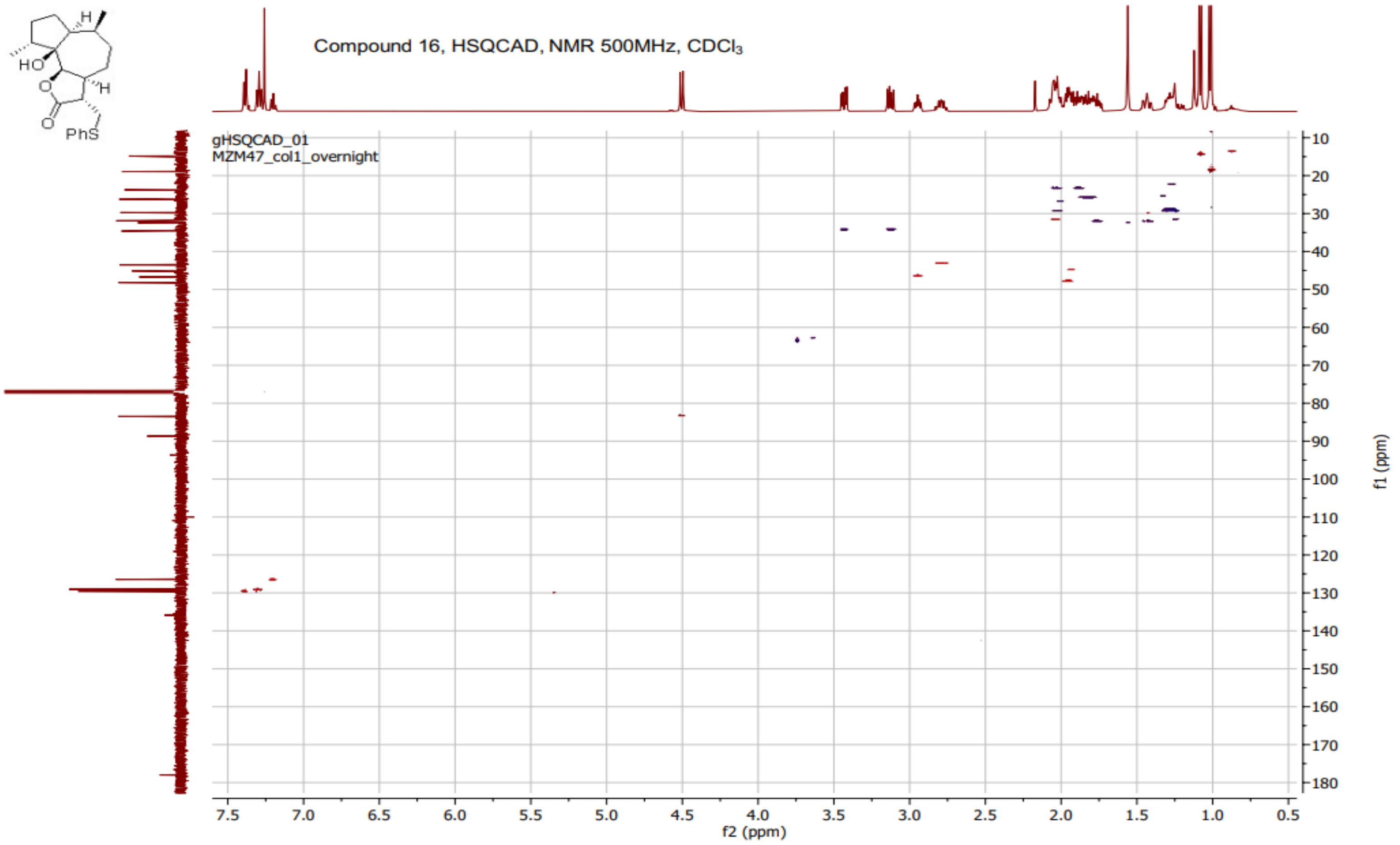
Compound 16, HSQCAD, NMR 500MHz, CDCl3
gHSQCAD_01
MZM47_col1_overnight
f2 (ppm)
f1 (ppm)
HO
PhS

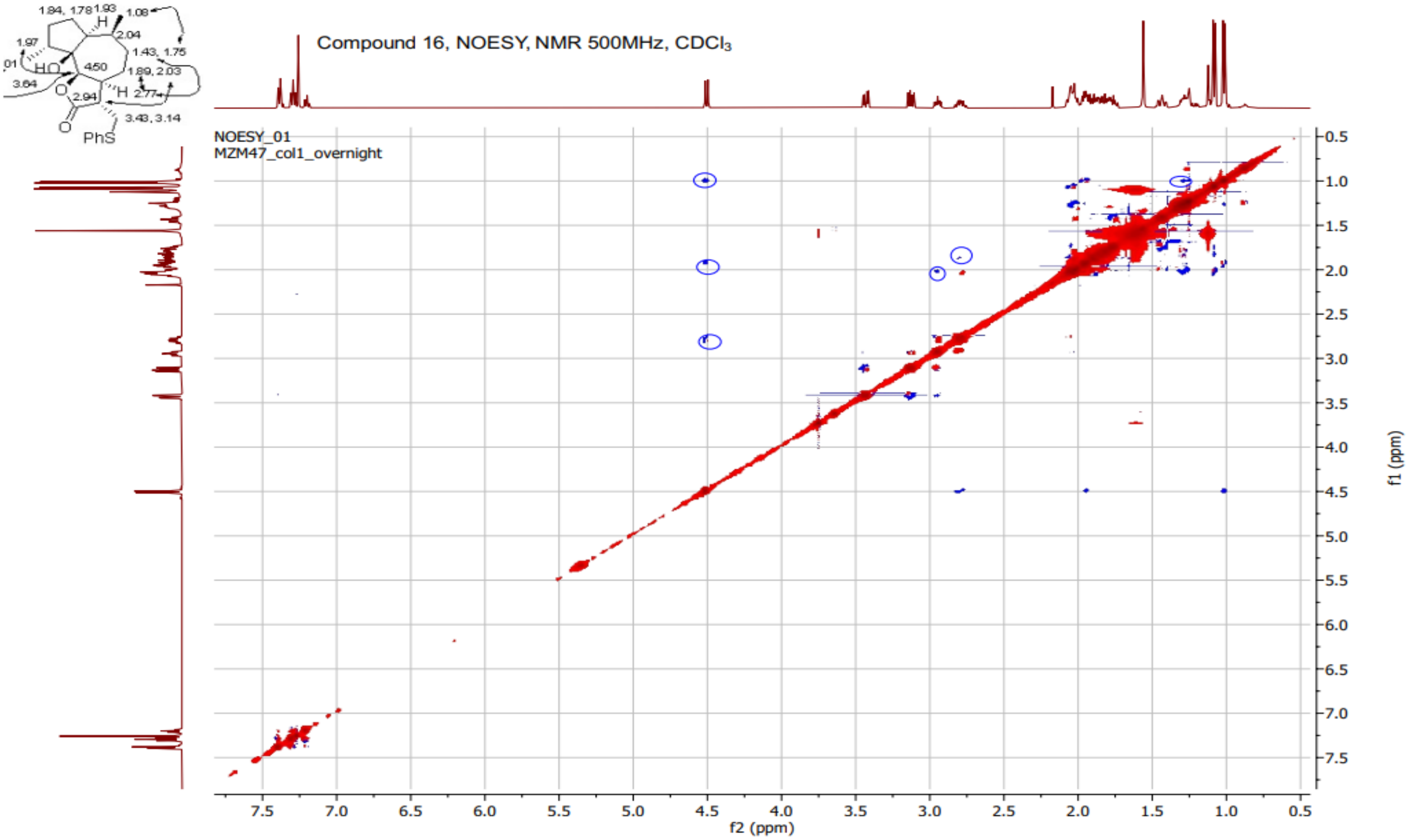
Compound 16, NOESY, NMR 500MHz, CDCl3
NOESY_01
MZM47_col1_overnight
PhS
f2 (ppm)
f1 (ppm)

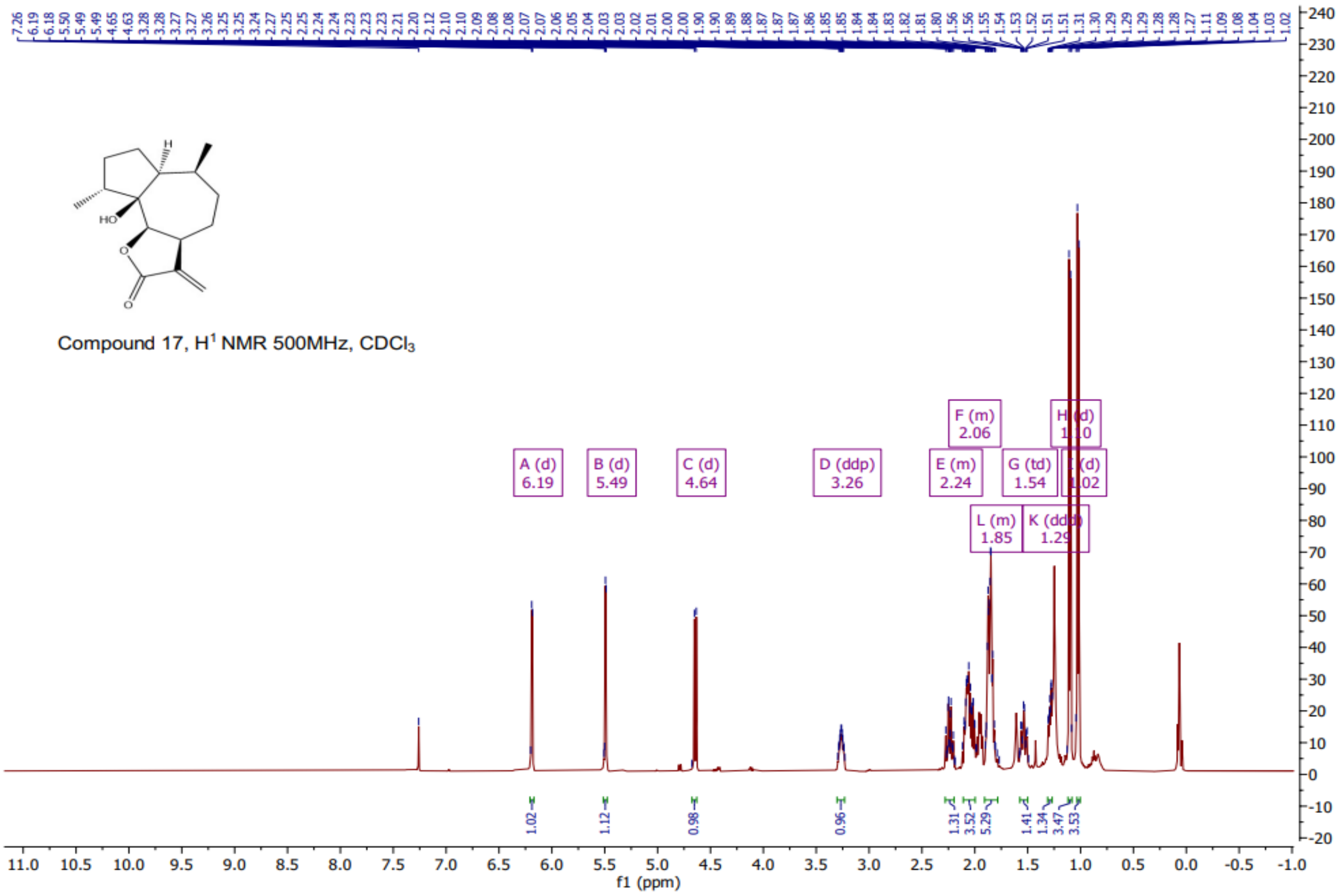
Compound 17, H1 NMR 500MHz, CDCl3
A (d) 6.19
B (d) 5.49
C (d) 4.64
D (ddp) 3.26
E (m) 2.24
F (m) 2.06
G (td) 1.54
L (m) 1.85
f1 (ppm)

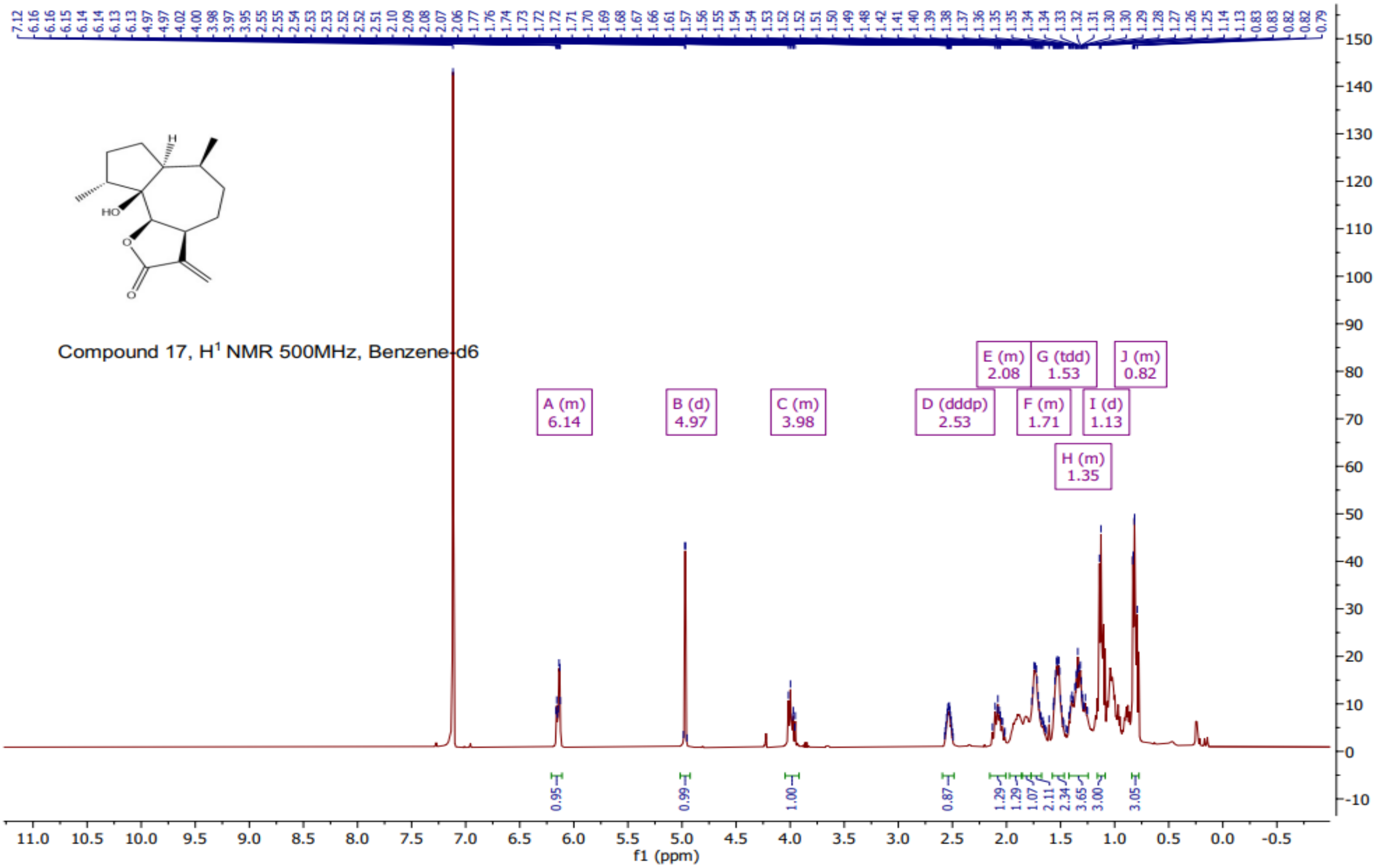
7.12
6.16
6.16
6.15
6.14
6.14
6.13
6.13
6.13
4.97
4.97
4.02
4.00
3.98
3.97
3.95
2.55
2.55
2.54
2.53
2.53
2.52
2.52
2.51
2.10
2.09
2.08
2.07
2.06
1.77
1.76
1.74
1.73
1.72
1.71
1.70
1.69
1.68
1.67
1.66
1.61
1.57
1.56
1.55
1.54
1.54
1.53
1.52
1.52
1.51
1.50
1.49
1.48
1.42
1.41
1.40
1.39
1.38
1.37
1.36
1.35
1.35
1.34
1.34
1.33
1.32
1.31
1.30
1.30
1.29
1.28
1.27
1.26
1.25
1.14
1.13
0.83
0.83
0.82
0.79
HO
O
O
H
Compound 17, H1 NMR 500MHz, Benzene-d6
A (m) 6.14
B (d) 4.97
C (m) 3.98
D (dddp) 2.53
E (m) 2.08
F (m) 1.71
G (tdd) 1.53
H (m) 1.35
I (d) 1.13
J (m) 0.82
0.95
0.99
1.00
0.87
1.29
1.29
1.07
2.11
2.34
3.65
3.00
3.05
f1 (ppm)

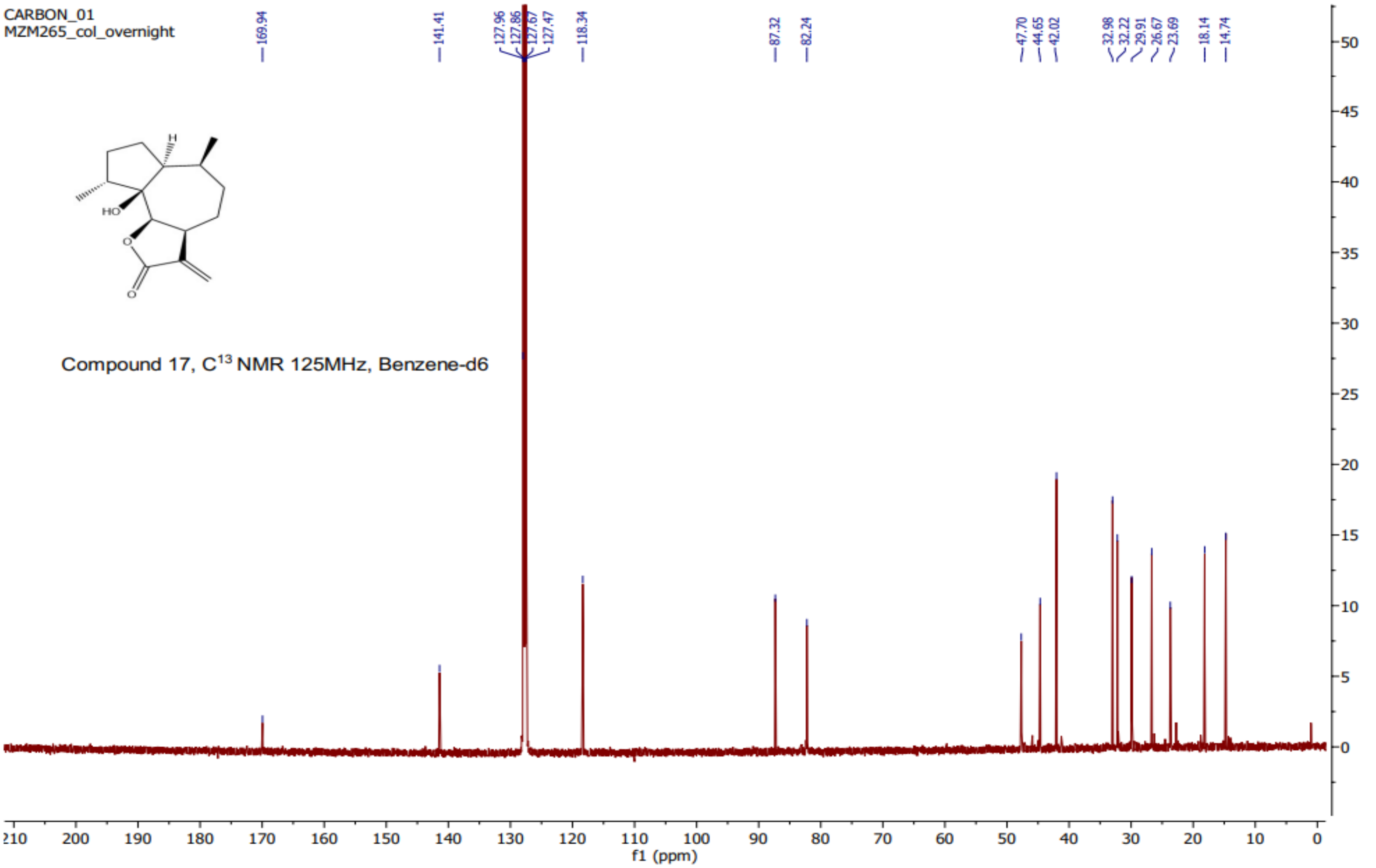
CARBON_01
MZM265_col_overnight
169.94
141.41
127.96
127.86
127.67
127.47
118.34
87.32
82.24
47.70
44.65
42.02
32.98
32.22
29.91
26.67
23.69
18.14
14.74
HO
O
O
H
Compound 17, C13 NMR 125MHz, Benzene-d6
f1 (ppm)

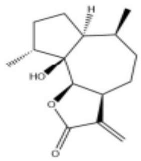

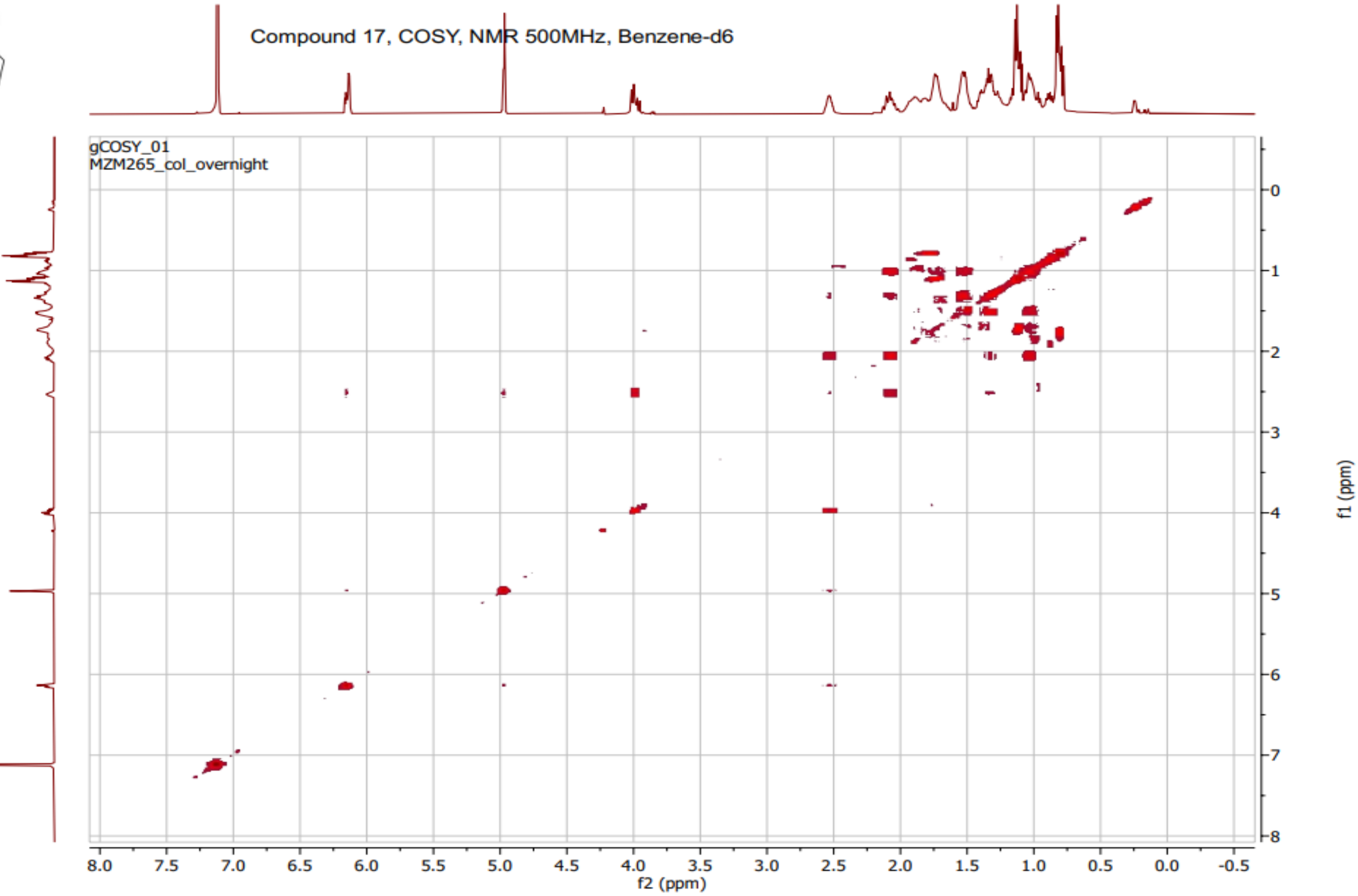

Compound 17, COSY, NMR 500MHz, Benzene-d6
gCOSY_01
MZM265_col_overnight
f2 (ppm)
f1 (ppm)


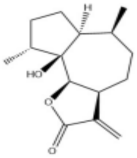

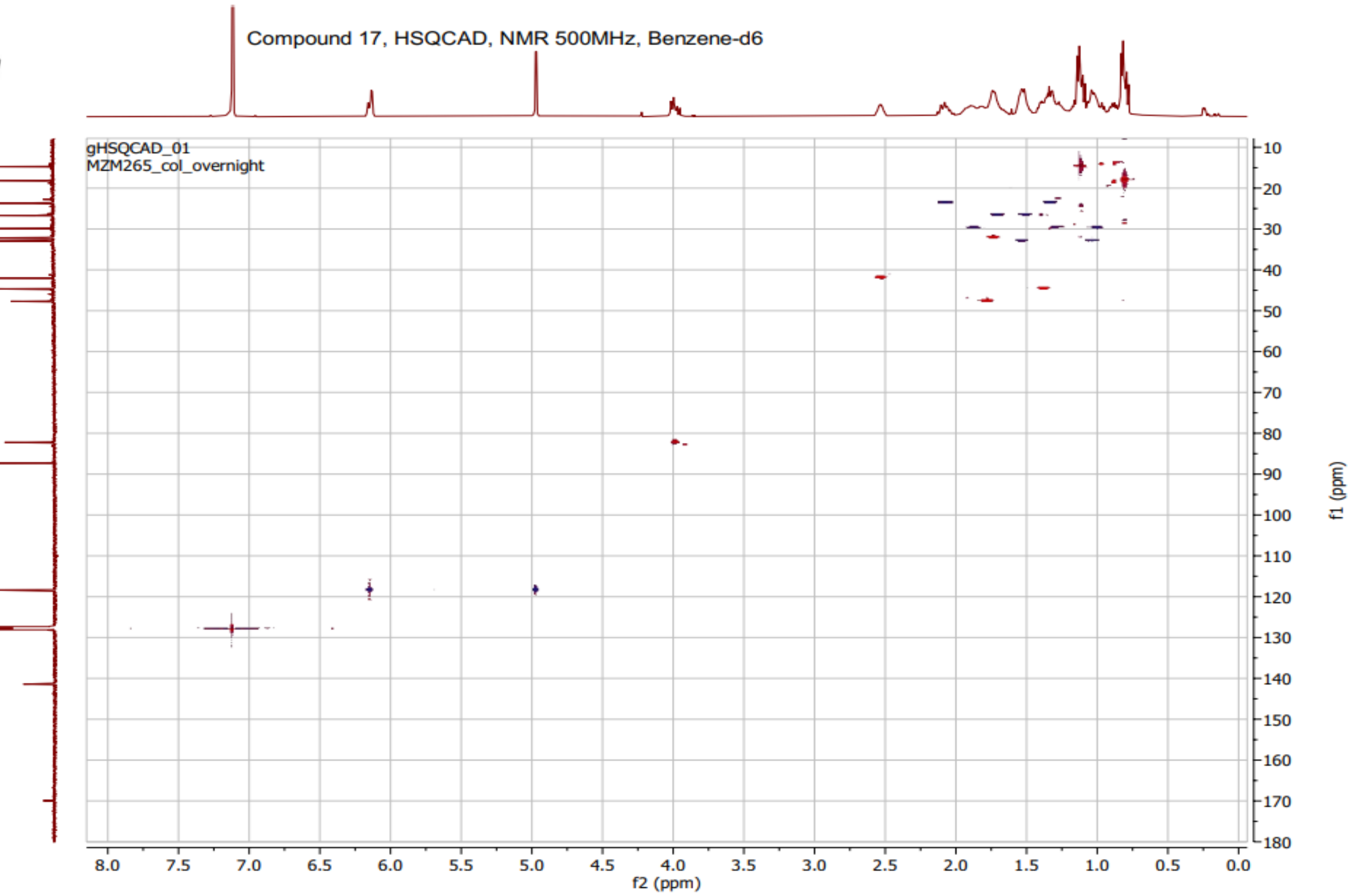

Compound 17, HSQCAD, NMR 500MHz, Benzene-d6
gHSQCAD_01
MZM265_col_overnight
f2 (ppm)
f1 (ppm)

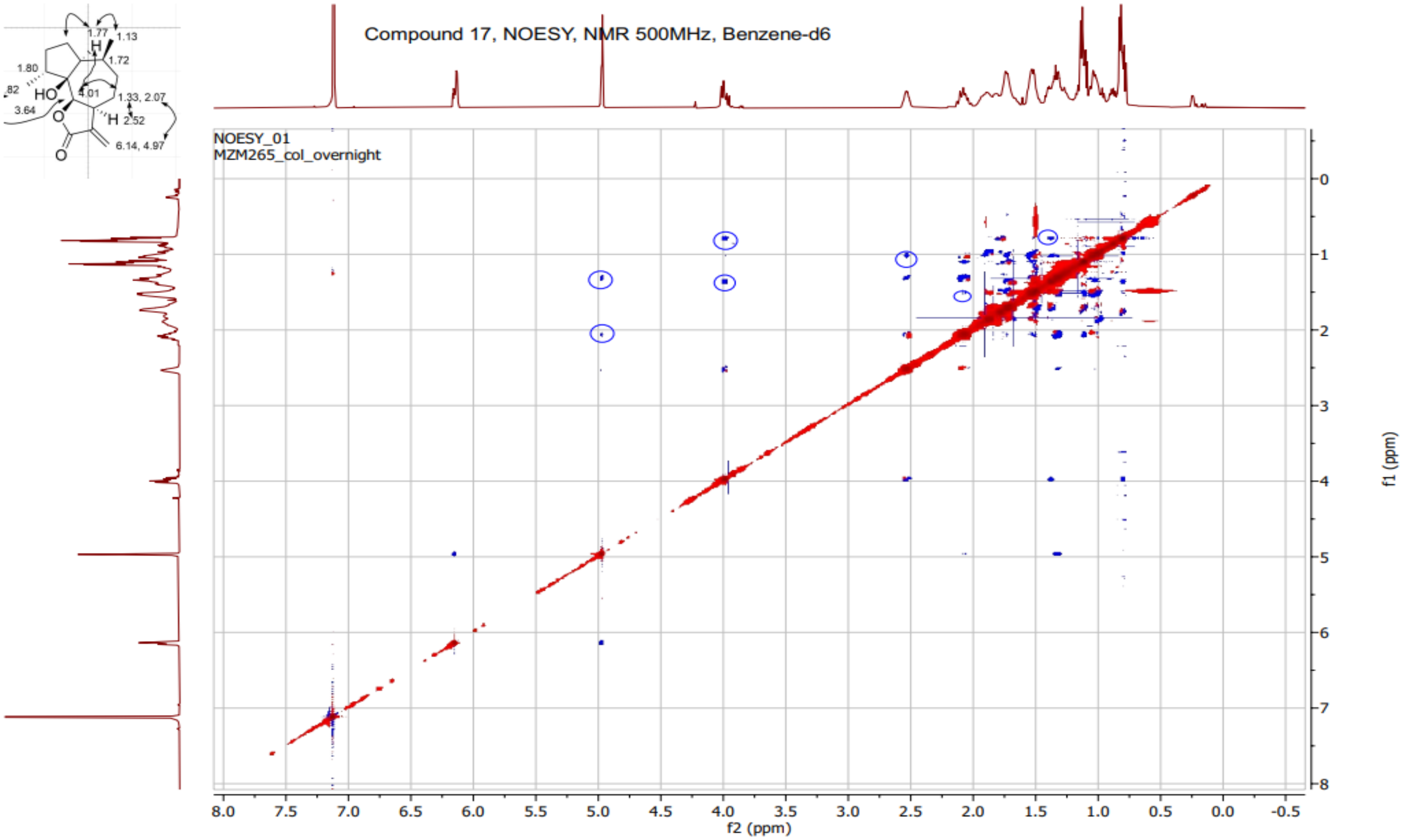
Compound 17, NOESY, NMR 500MHz, Benzene-d6
NOESY_01
MZM265_col_overnight
f2 (ppm)
f1 (ppm)

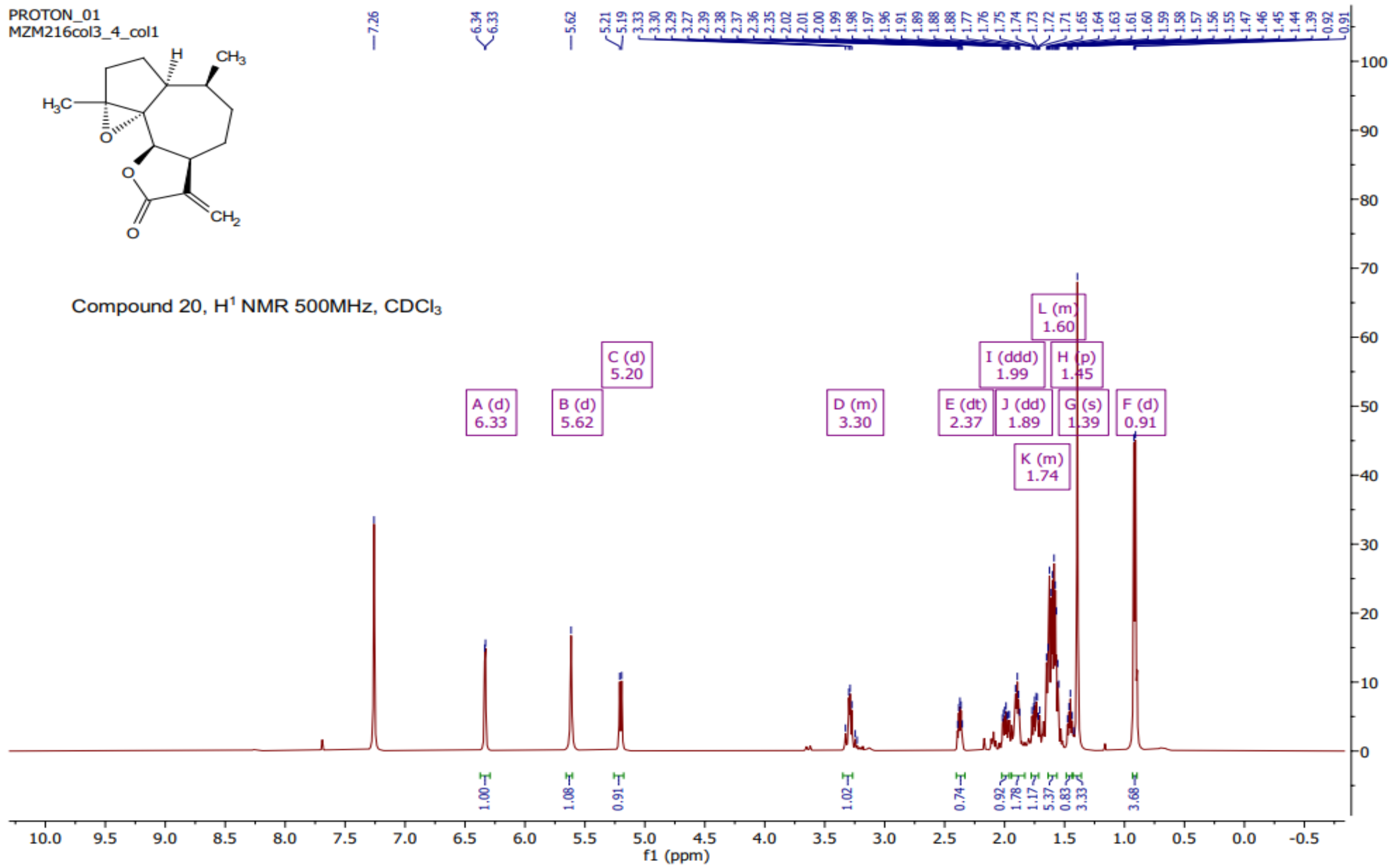
PROTON_01
MZM216col3_4_col1
Compound 20, H1 NMR 500MHz, CDCl3
A (d) 6.33
B (d) 5.62
C (d) 5.20
D (m) 3.30
E (dt) 2.37
I (ddd) 1.99
J (dd) 1.89
K (m) 1.74
L (m) 1.60
H (p) 1.45
G (s) 1.39
F (d) 0.91
f1 (ppm)

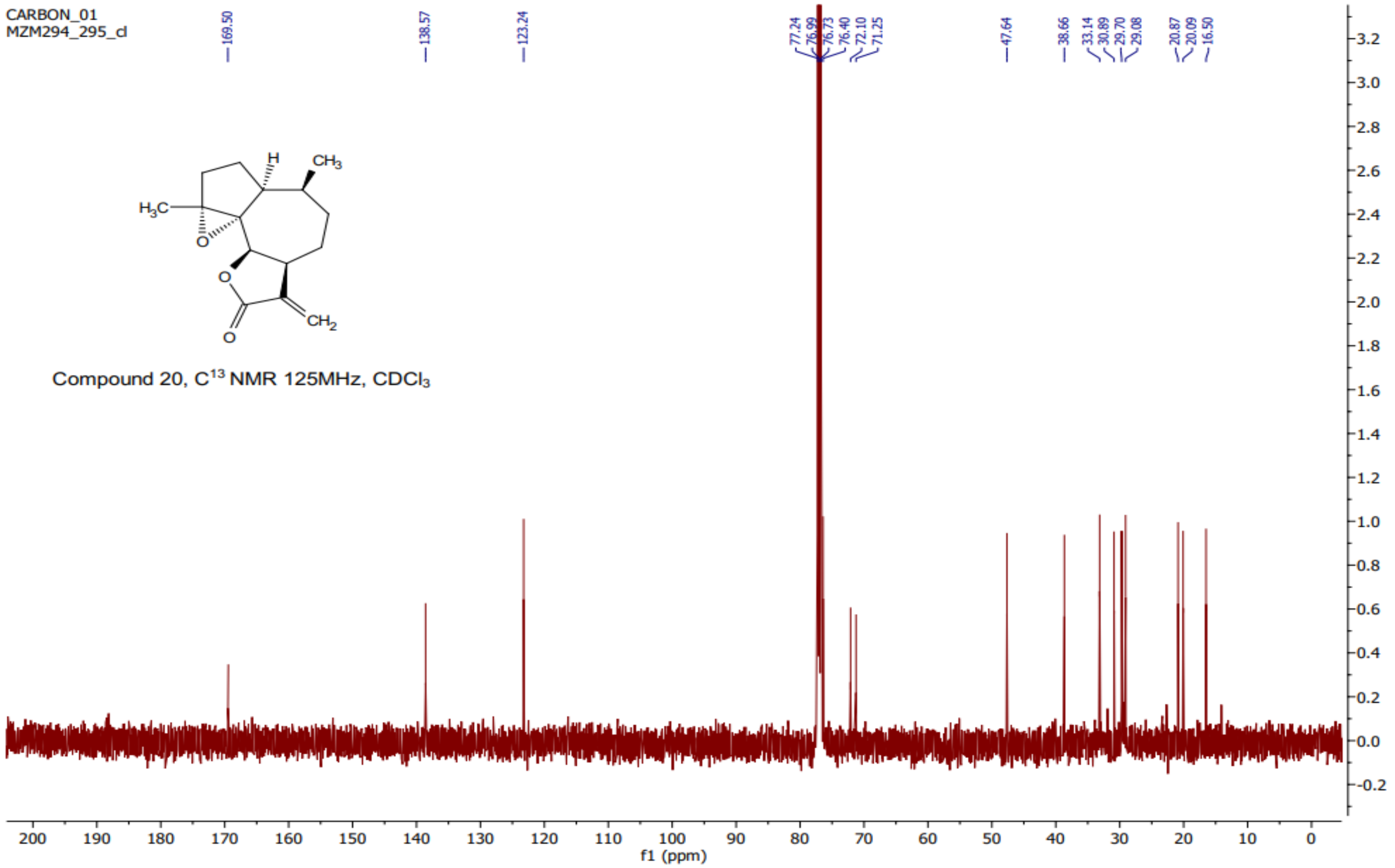


Compound 20, $C^{13}$ NMR 125MHz, $CDCl_3$

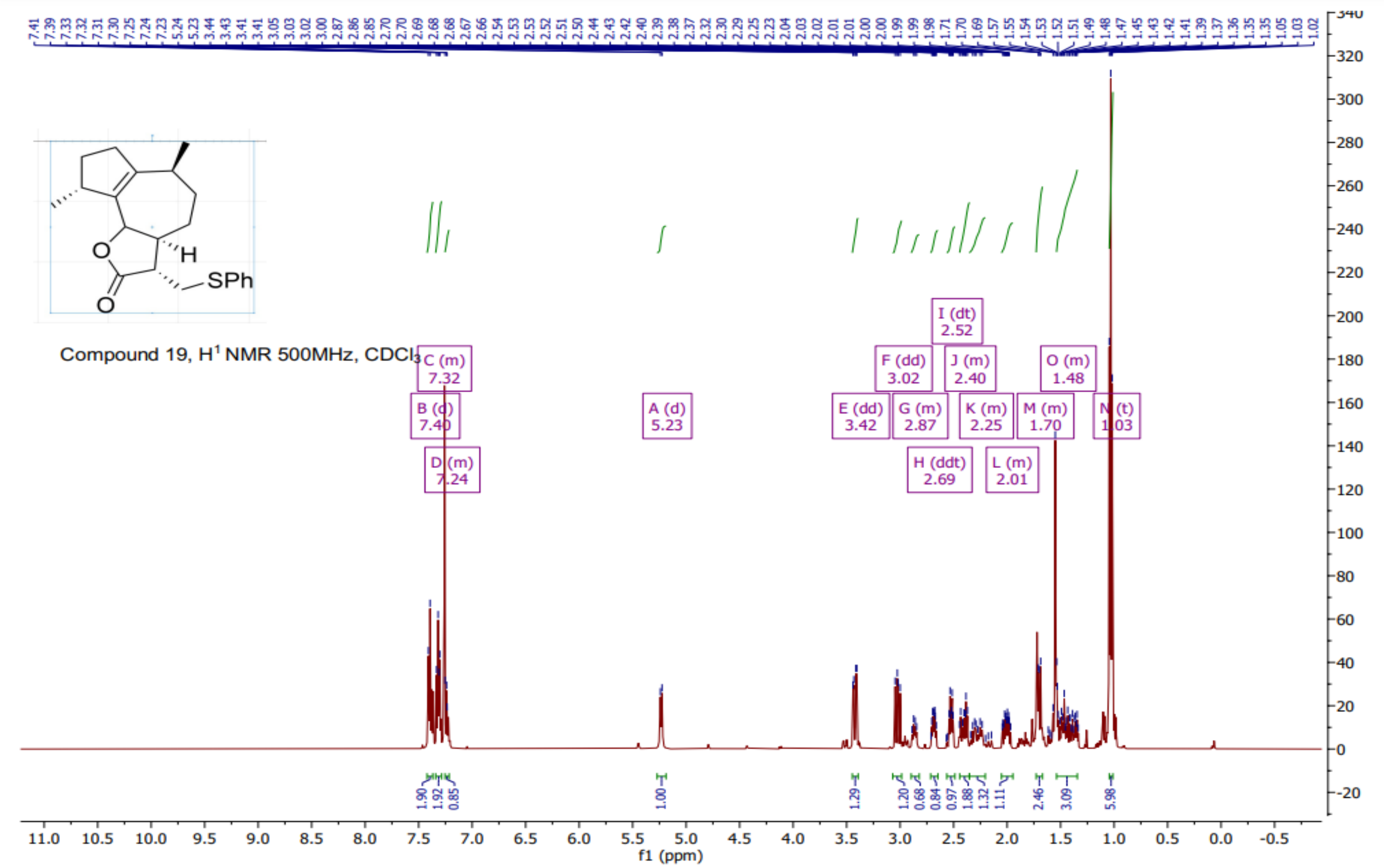


Compound 19, $H^1$ NMR 500MHz, $CDCl_3$

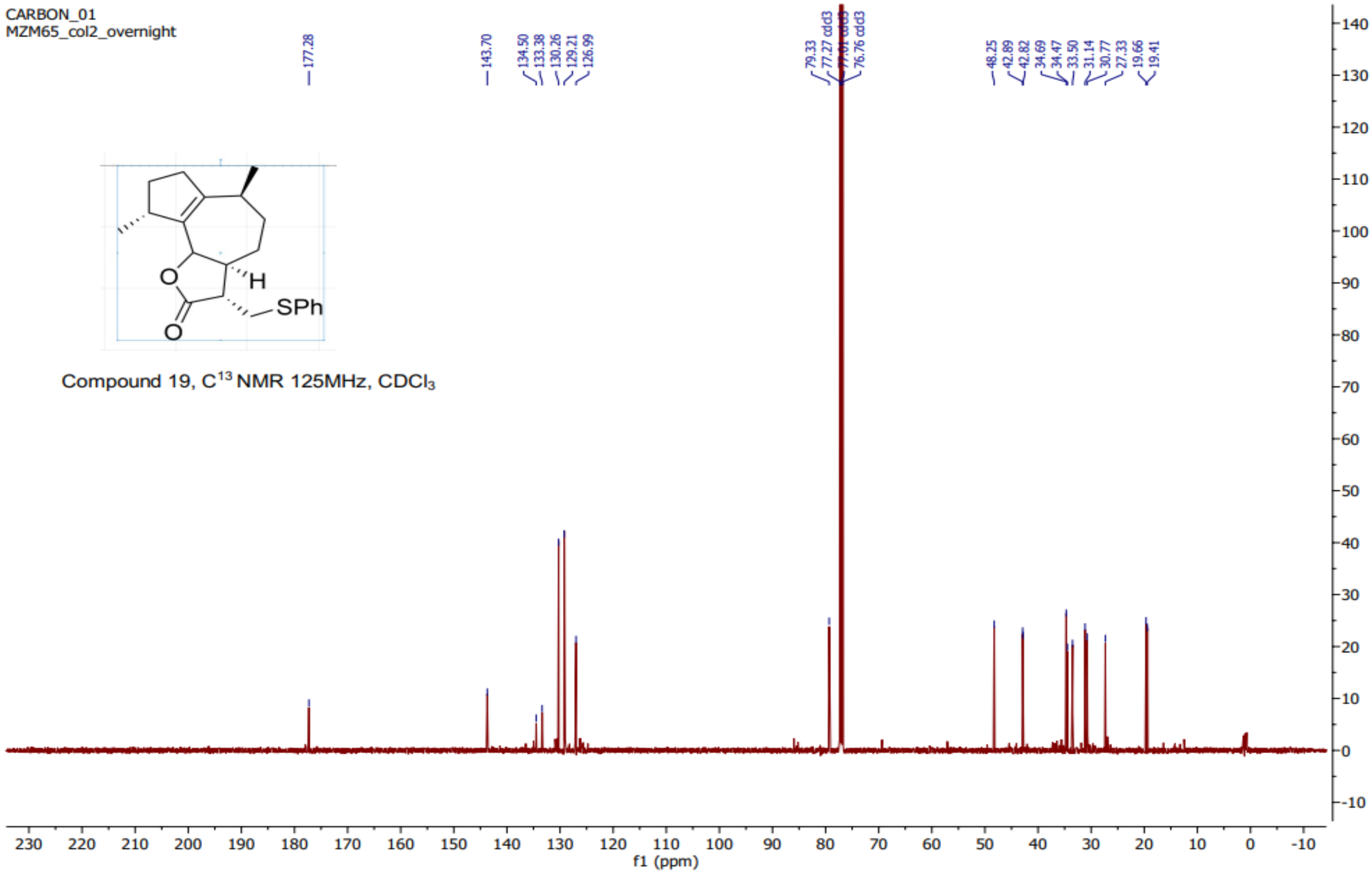
CARBON_01
MZM65_col2_overnight
177.28
143.70
134.50
133.38
130.26
129.21
126.99
79.33
77.27 cdcl3
76.76 cdcl3
48.25
42.89
42.82
34.69
34.47
33.50
31.14
30.77
27.33
19.66
19.41
SPh
Compound 19, C[13] NMR 125MHz, CDCl$_3$
f1 (ppm)

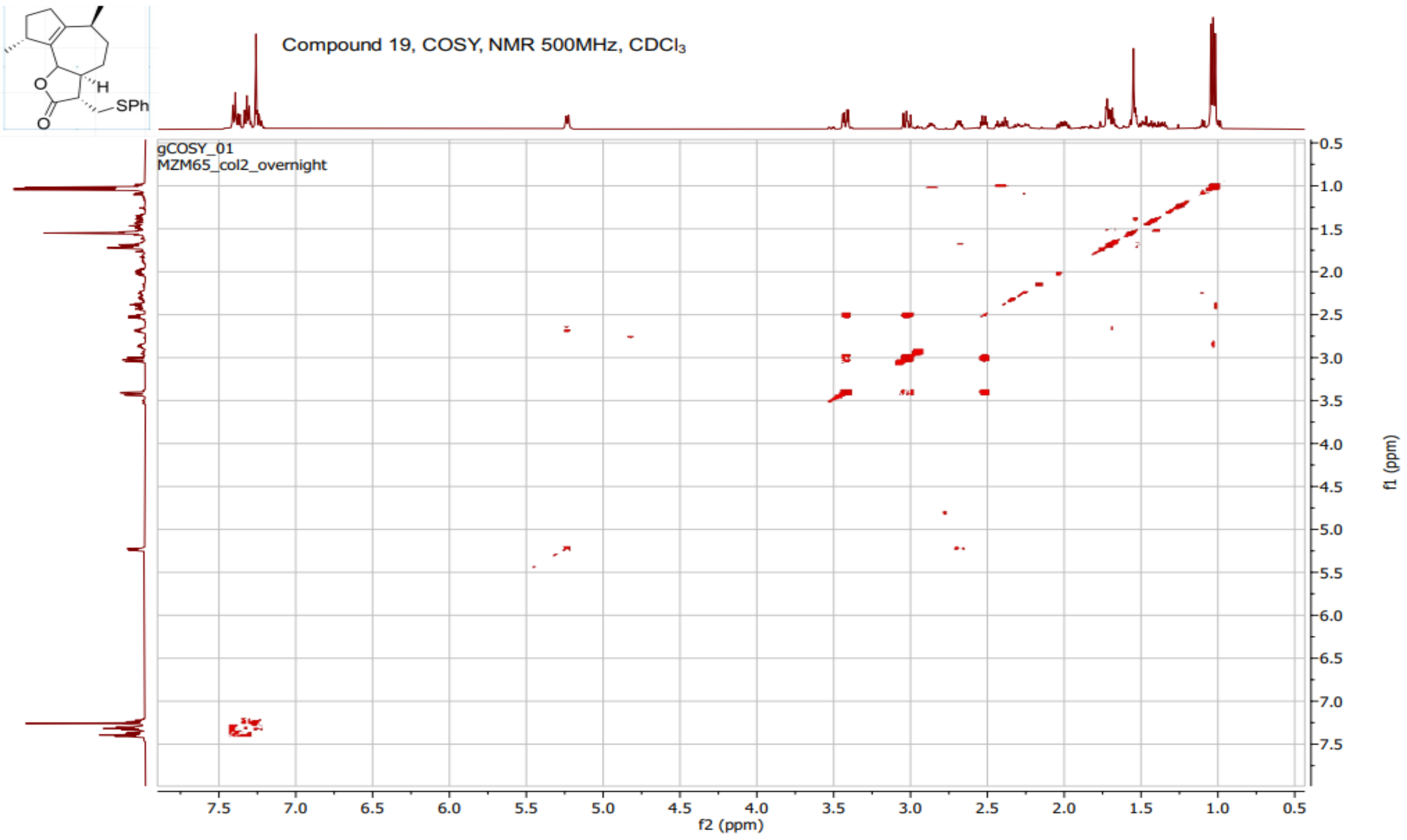
SPh
Compound 19, COSY, NMR 500MHz, CDCl$_3$
gCOSY_01
MZM65_col2_overnight
f2 (ppm)
f1 (ppm)

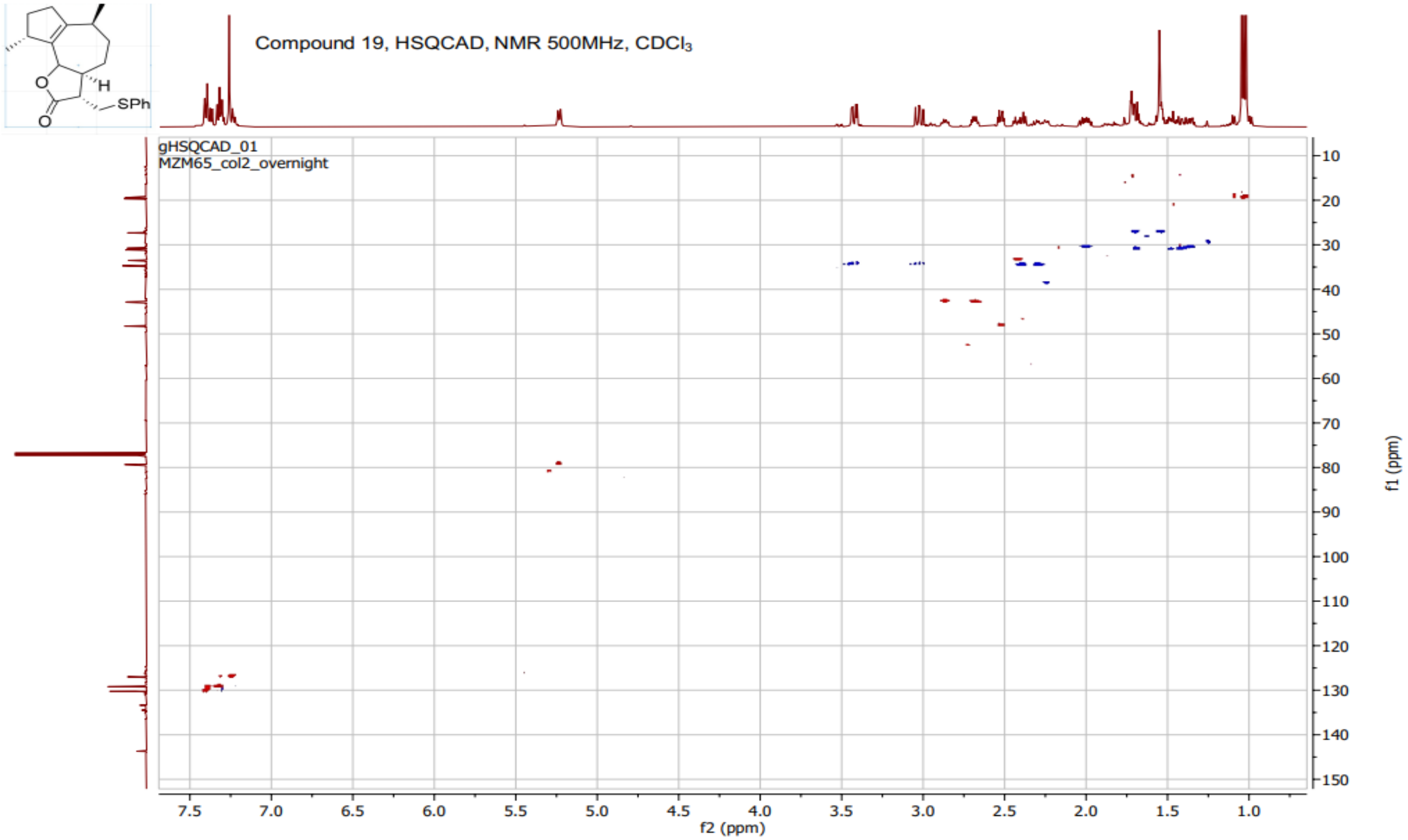
H
SPh
O
O
Compound 19, HSQCAD, NMR 500MHz, CDCl3
gHSQCAD_01
MZM65_col2_overnight
f2 (ppm)
f1 (ppm)

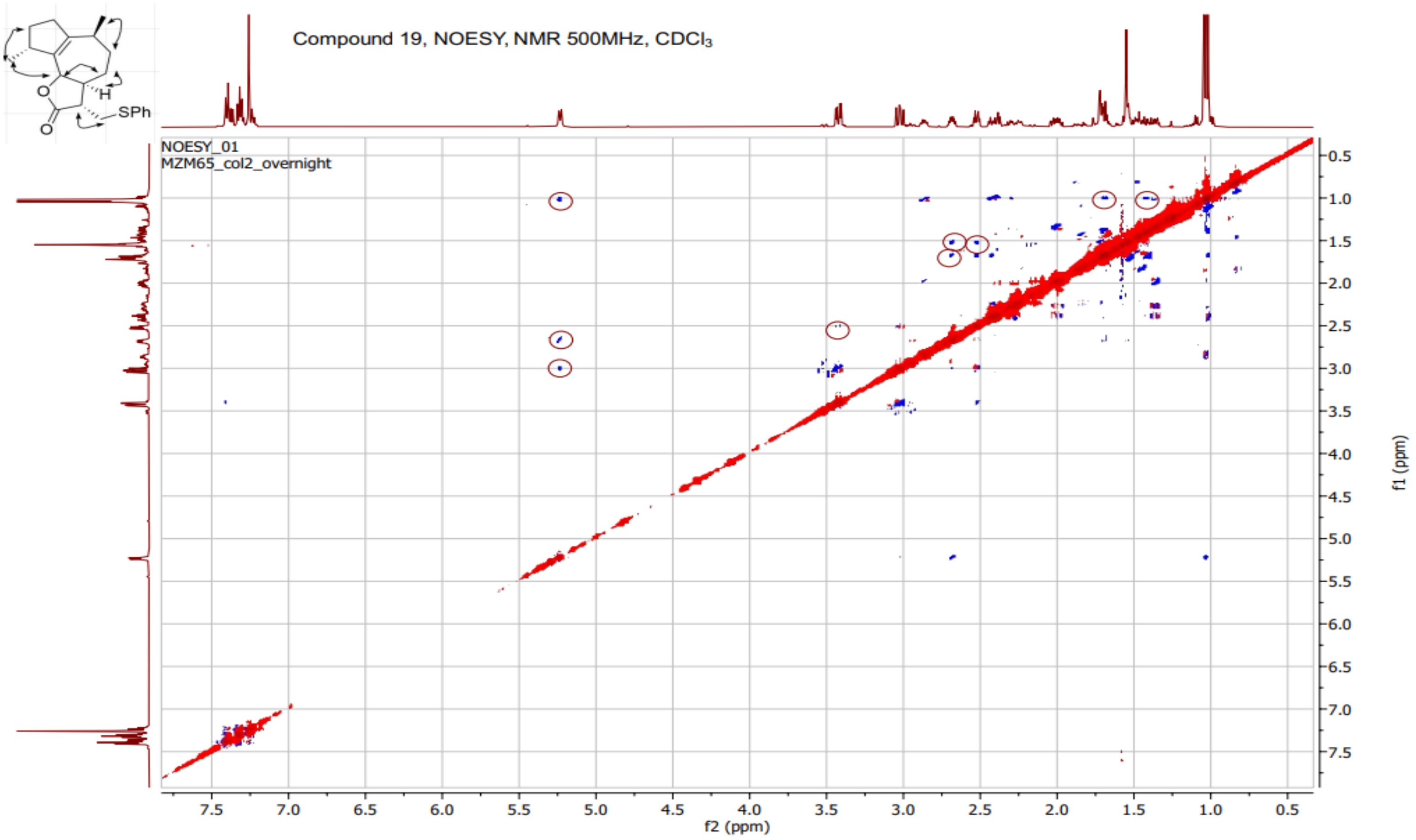
H
SPh
O
O
Compound 19, NOESY, NMR 500MHz, CDCl3
NOESY_01
MZM65_col2_overnight
f2 (ppm)
f1 (ppm)

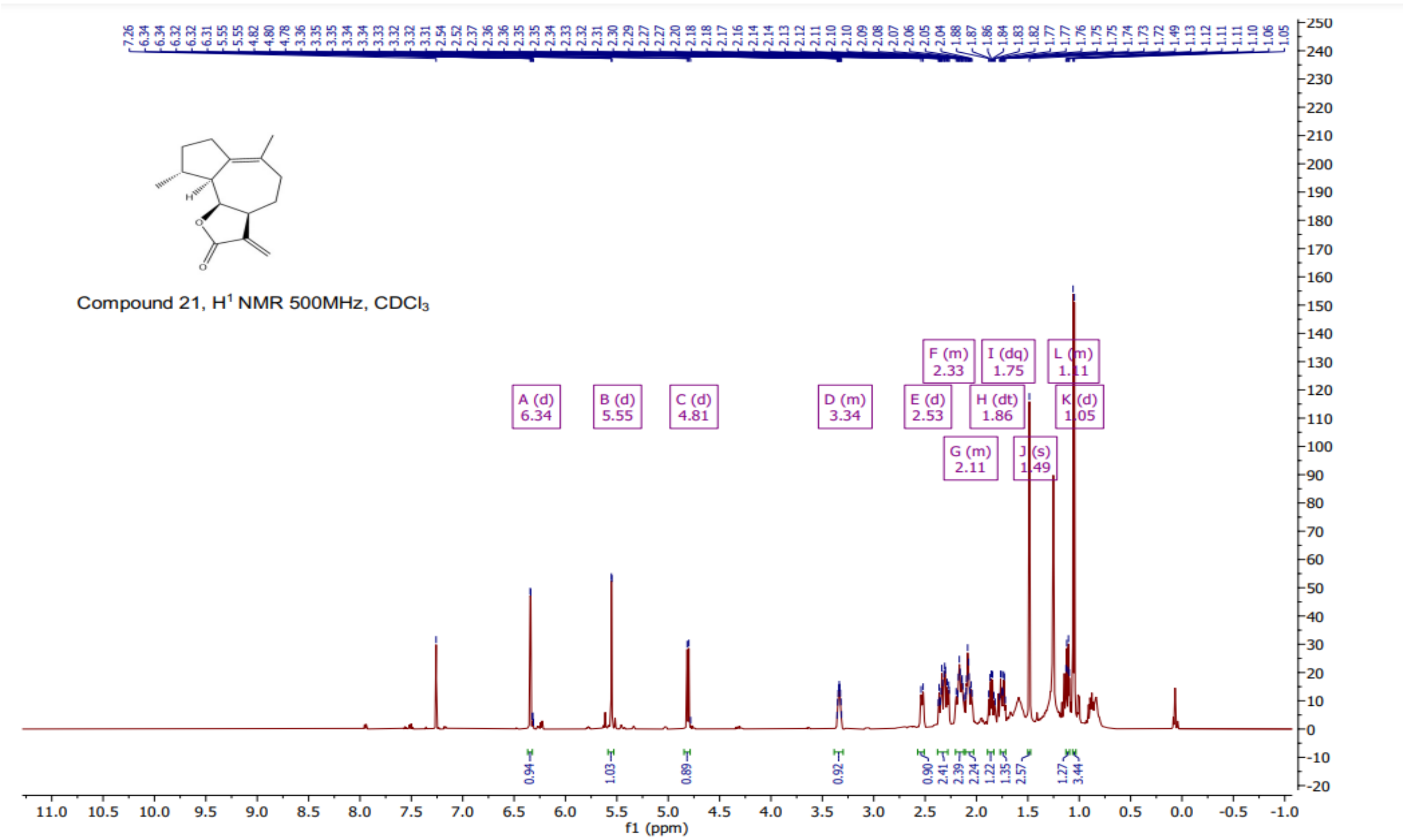
Compound 21, H1 NMR 500MHz, CDCl3
A (d) 6.34
B (d) 5.55
C (d) 4.81
D (m) 3.34
E (d) 2.53
F (m) 2.33
G (m) 2.11
H (dt) 1.86
I (dq) 1.75
J (s) 1.49
K (d) 1.05
L (m) 1.11
f1 (ppm)

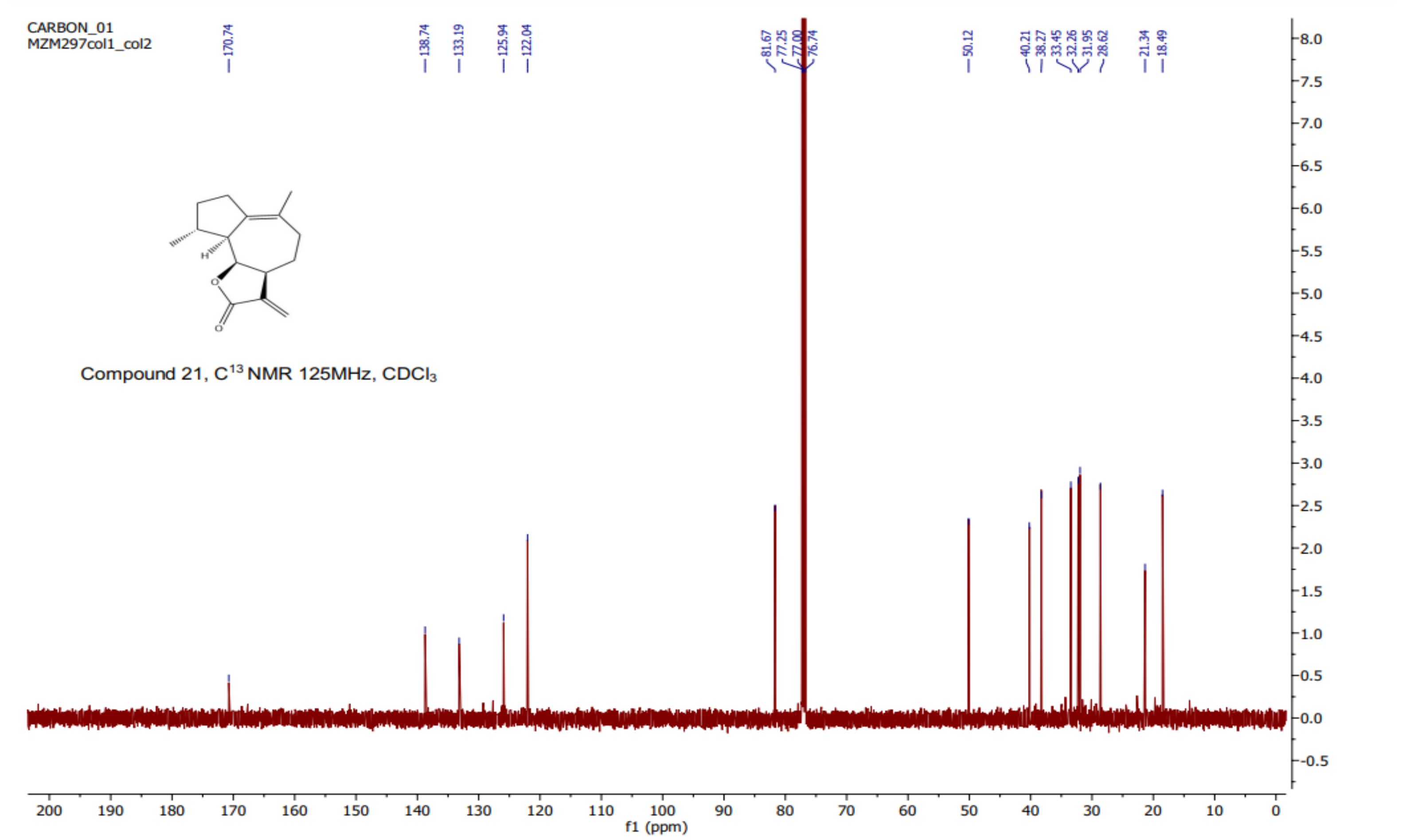
CARBON_01
MZM297col1_col2
170.74
138.74
133.19
125.94
122.04
81.67
77.25
77.00
76.74
50.12
40.21
38.27
33.45
32.26
31.95
28.62
21.34
18.49
Compound 21, C13 NMR 125MHz, CDCl3
f1 (ppm)

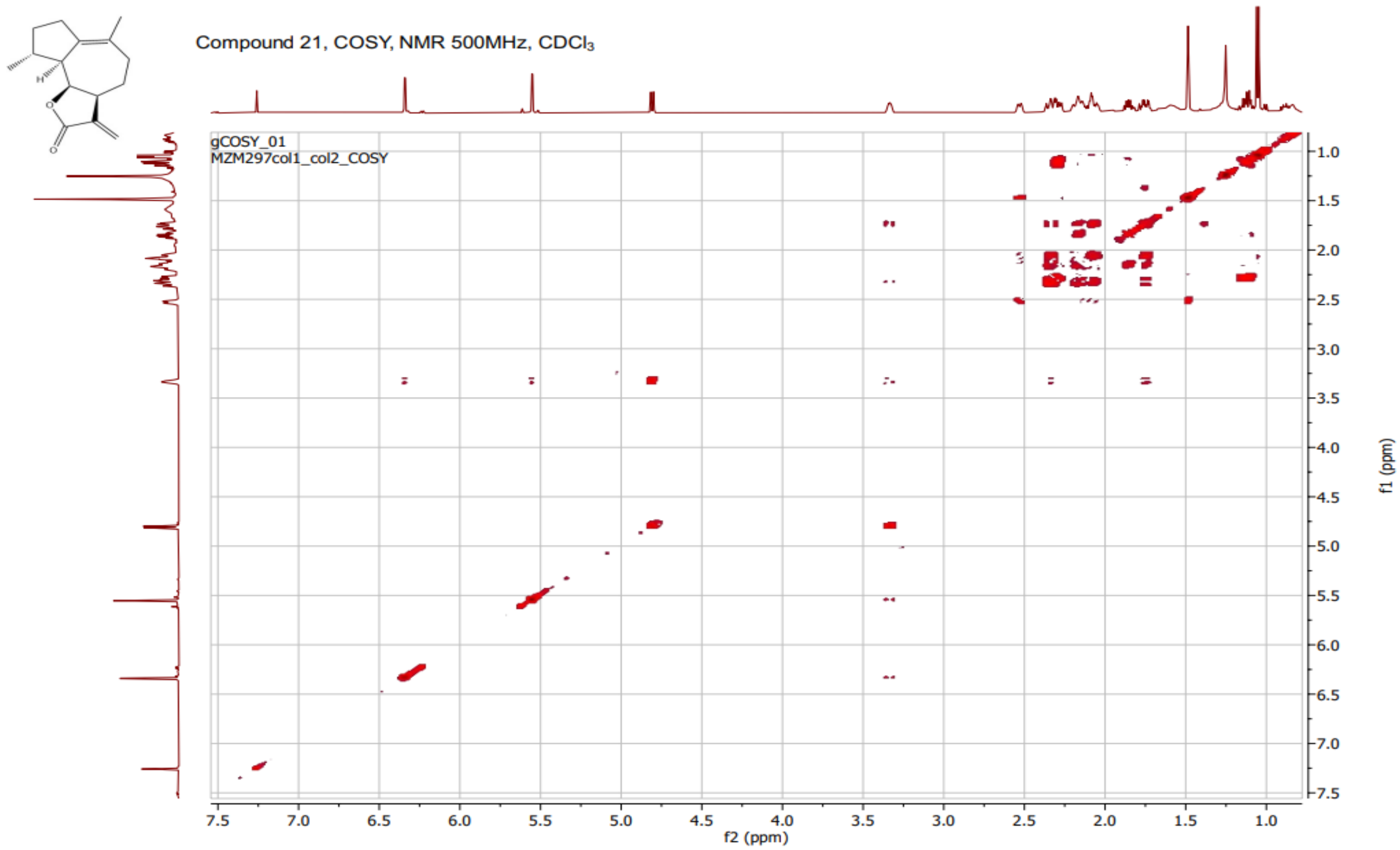
Compound 21, COSY, NMR 500MHz, CDCl3
gCOSY_01
MZM297col1_col2_COSY
f2 (ppm)
f1 (ppm)

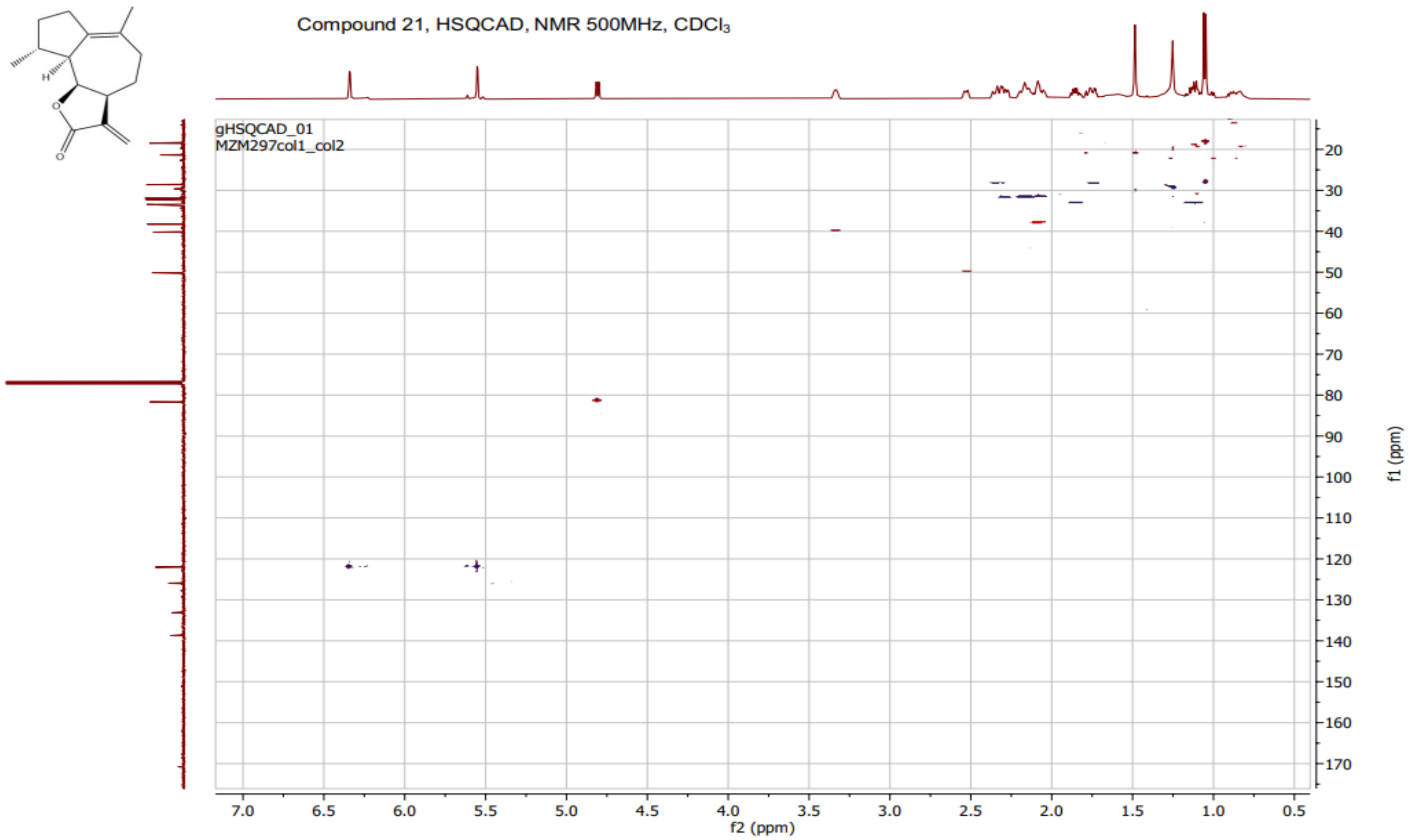
Compound 21, HSQCAD, NMR 500MHz, CDCl3
gHSQCAD_01
MZM297col1_col2
f2 (ppm)
f1 (ppm)

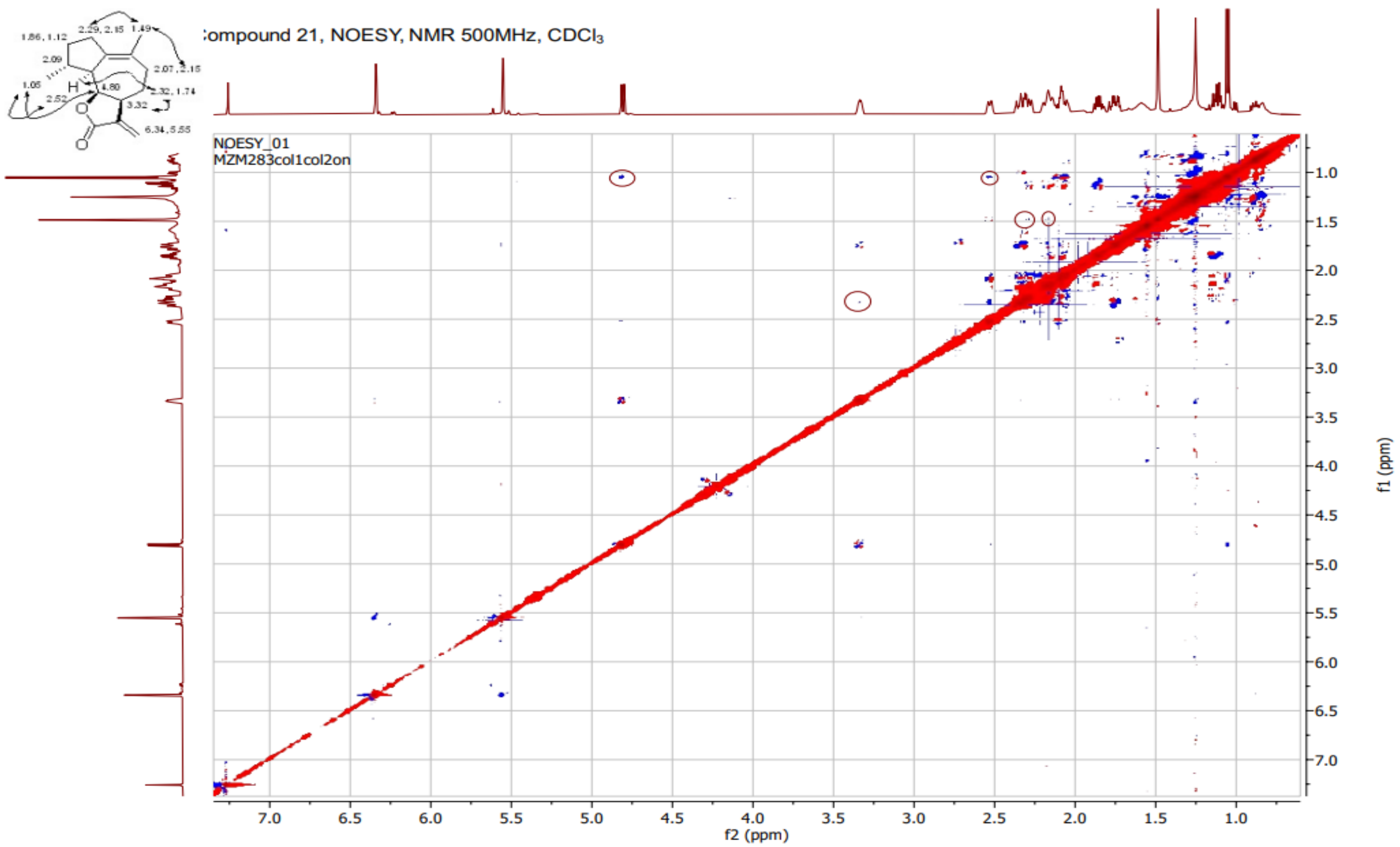
Compound 21, NOESY, NMR 500MHz, CDCl3
NOESY_01
MZM283col1col2on
f2 (ppm)
f1 (ppm)

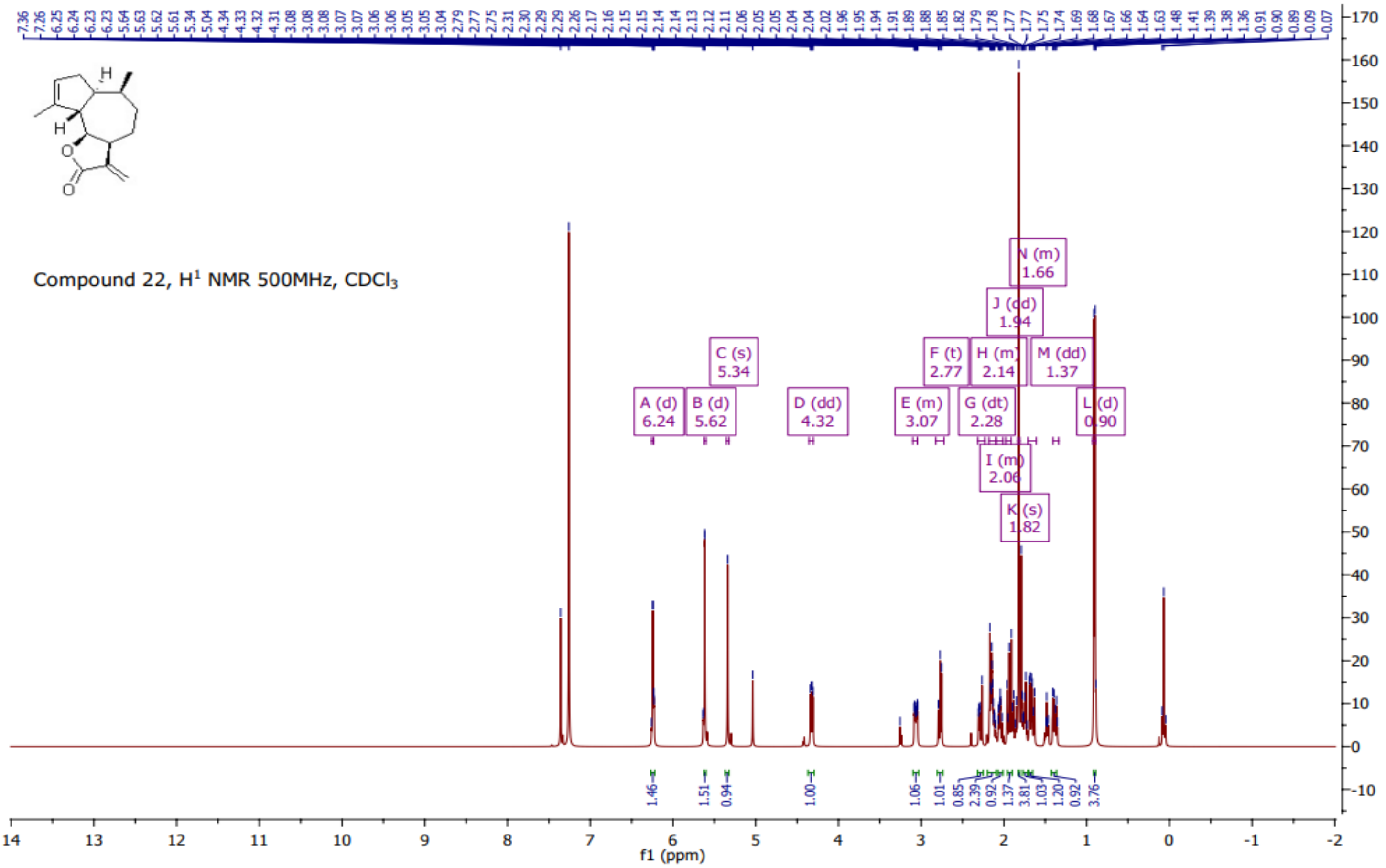
Compound 22, H1 NMR 500MHz, CDCl3
A (d) 6.24
B (d) 5.62
C (s) 5.34
D (dd) 4.32
E (m) 3.07
F (t) 2.77
G (dt) 2.28
H (m) 2.14
I (m) 2.06
J (dd) 1.94
K (s) 1.82
L (d) 0.90
M (dd) 1.37
N (m) 1.66
f1 (ppm)

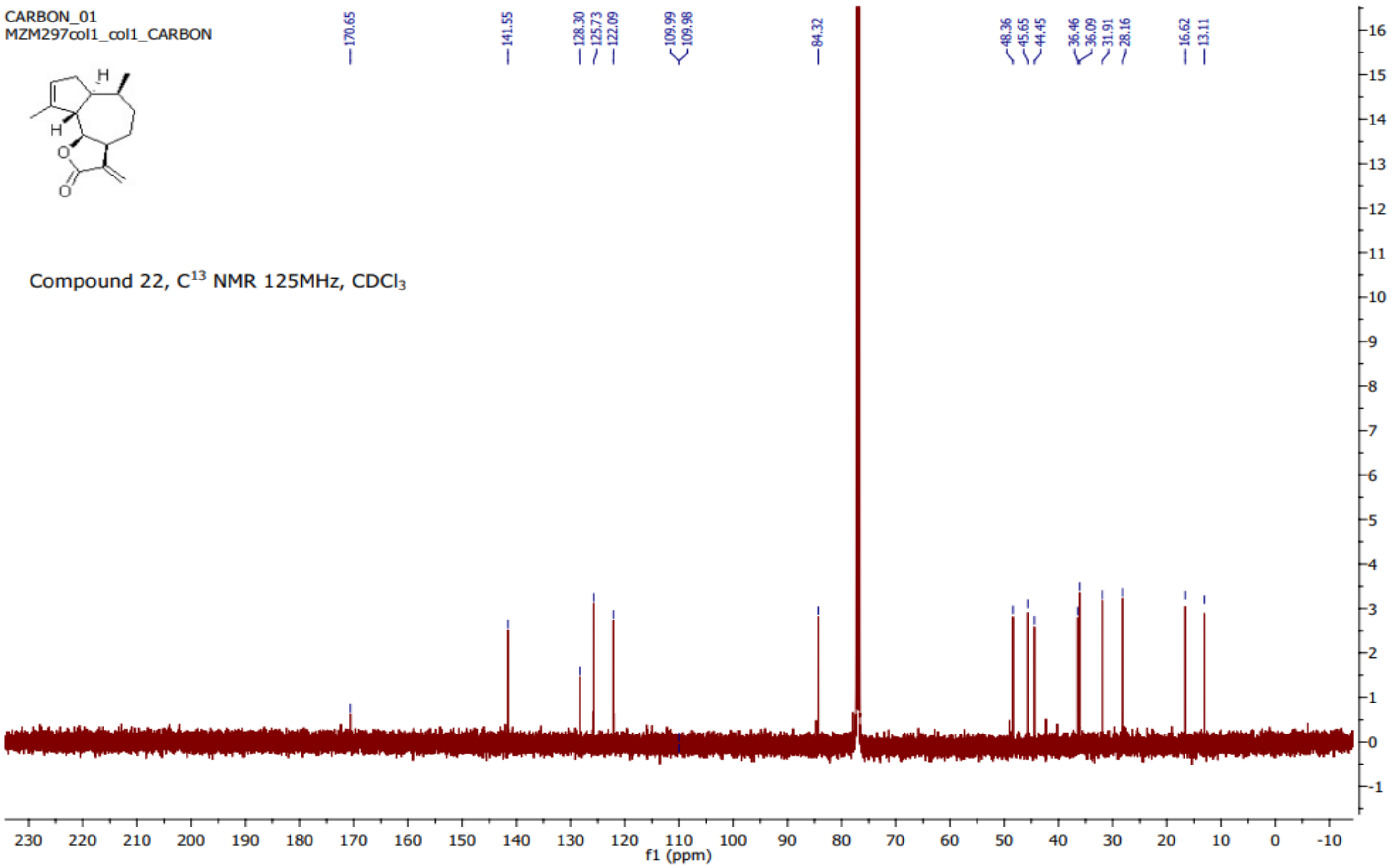
CARBON_01
MZM297col1_col1_CARBON
170.65
141.55
128.30
125.73
122.09
109.99
109.98
84.32
48.36
45.65
44.45
36.46
36.09
31.91
28.16
16.62
13.11
H
H
O
O
Compound 22, C13 NMR 125MHz, CDCl3
f1 (ppm)

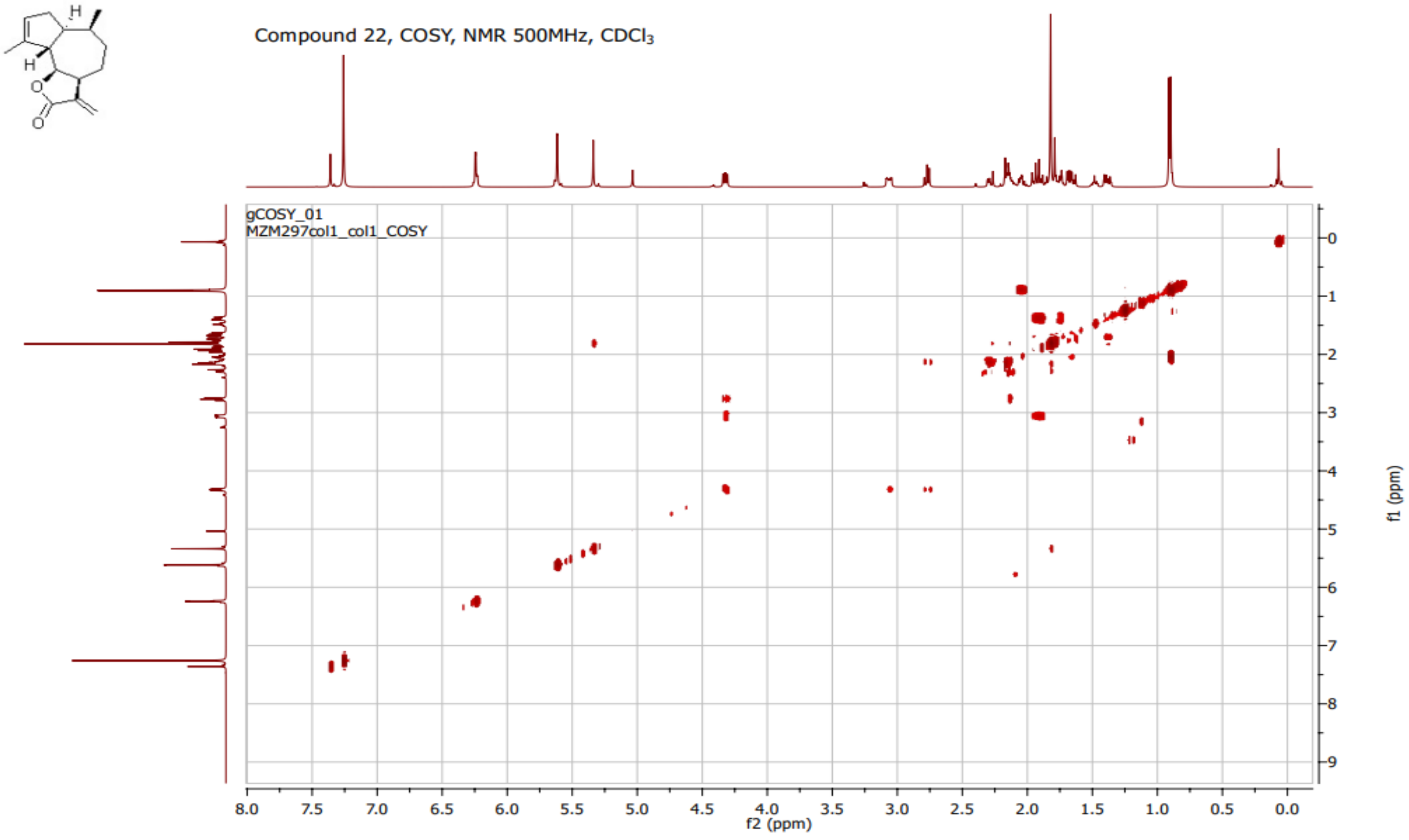
H
H
O
O
Compound 22, COSY, NMR 500MHz, CDCl3
gCOSY_01
MZM297col1_col1_COSY
f2 (ppm)
f1 (ppm)

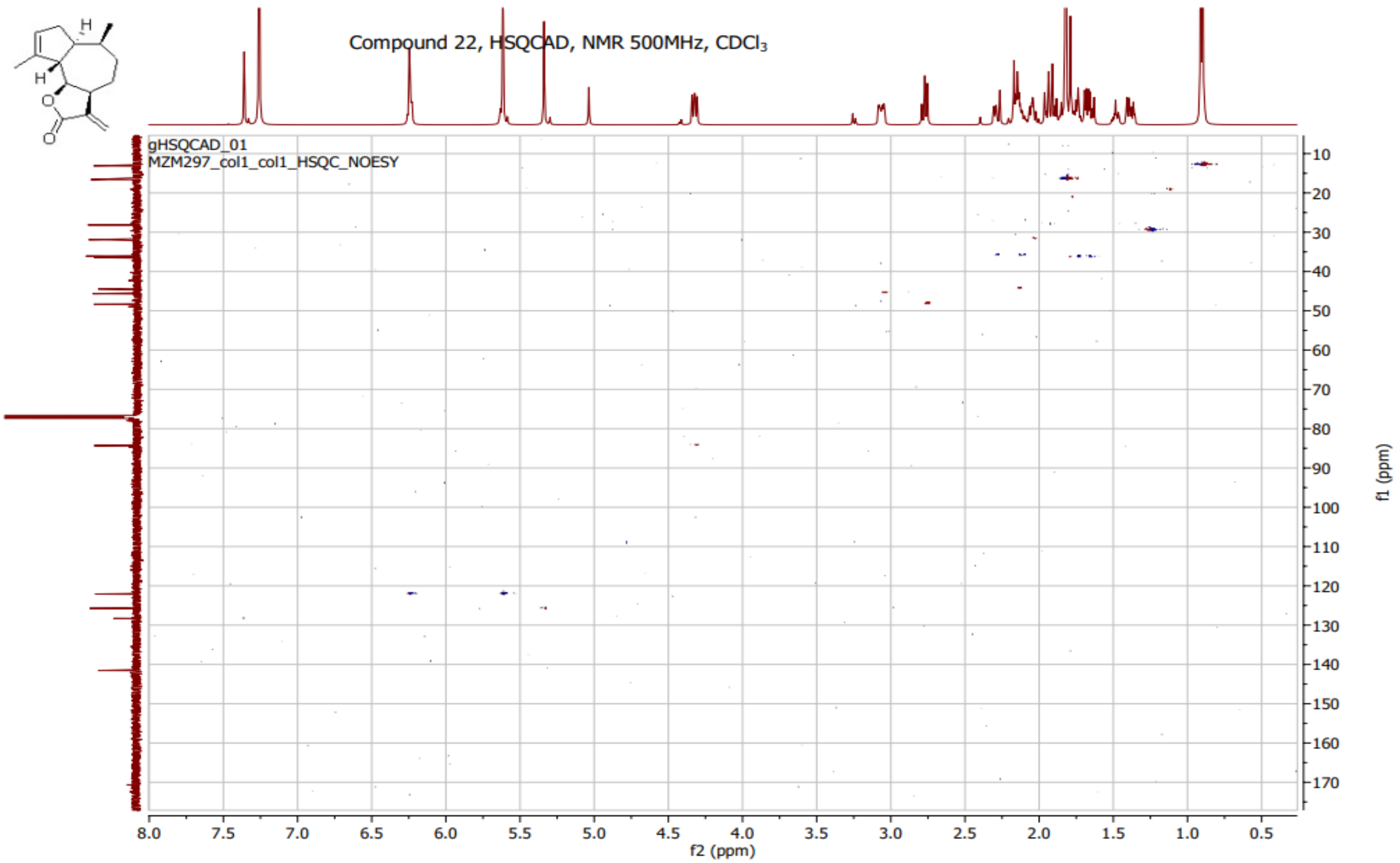
Compound 22, HSQCAD, NMR 500MHz, CDCl3
gHSQCAD_01
MZM297_col1_col1_HSQC_NOESY
f2 (ppm)
f1 (ppm)

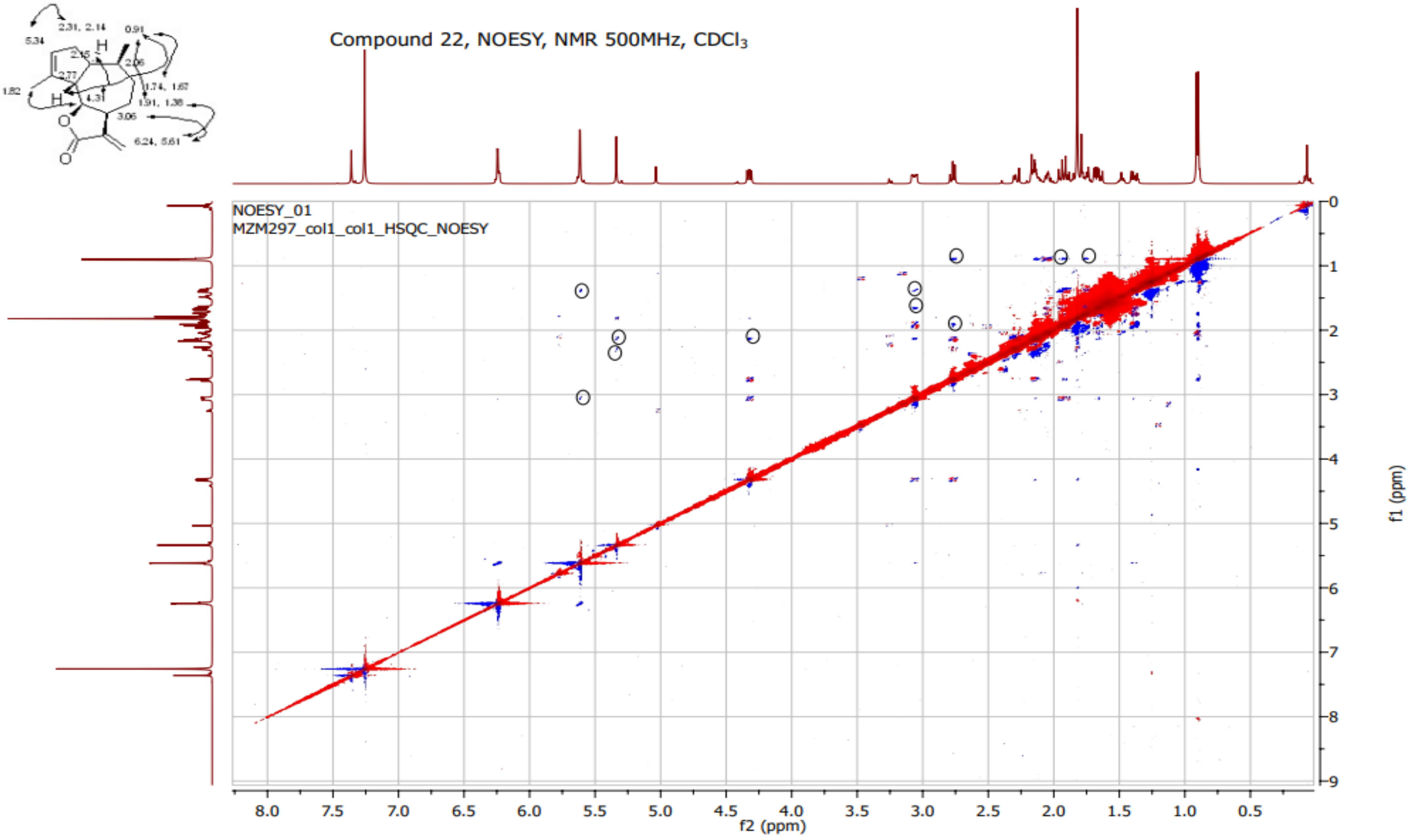
Compound 22, NOESY, NMR 500MHz, CDCl3
NOESY_01
MZM297_col1_col1_HSQC_NOESY
f2 (ppm)
f1 (ppm)

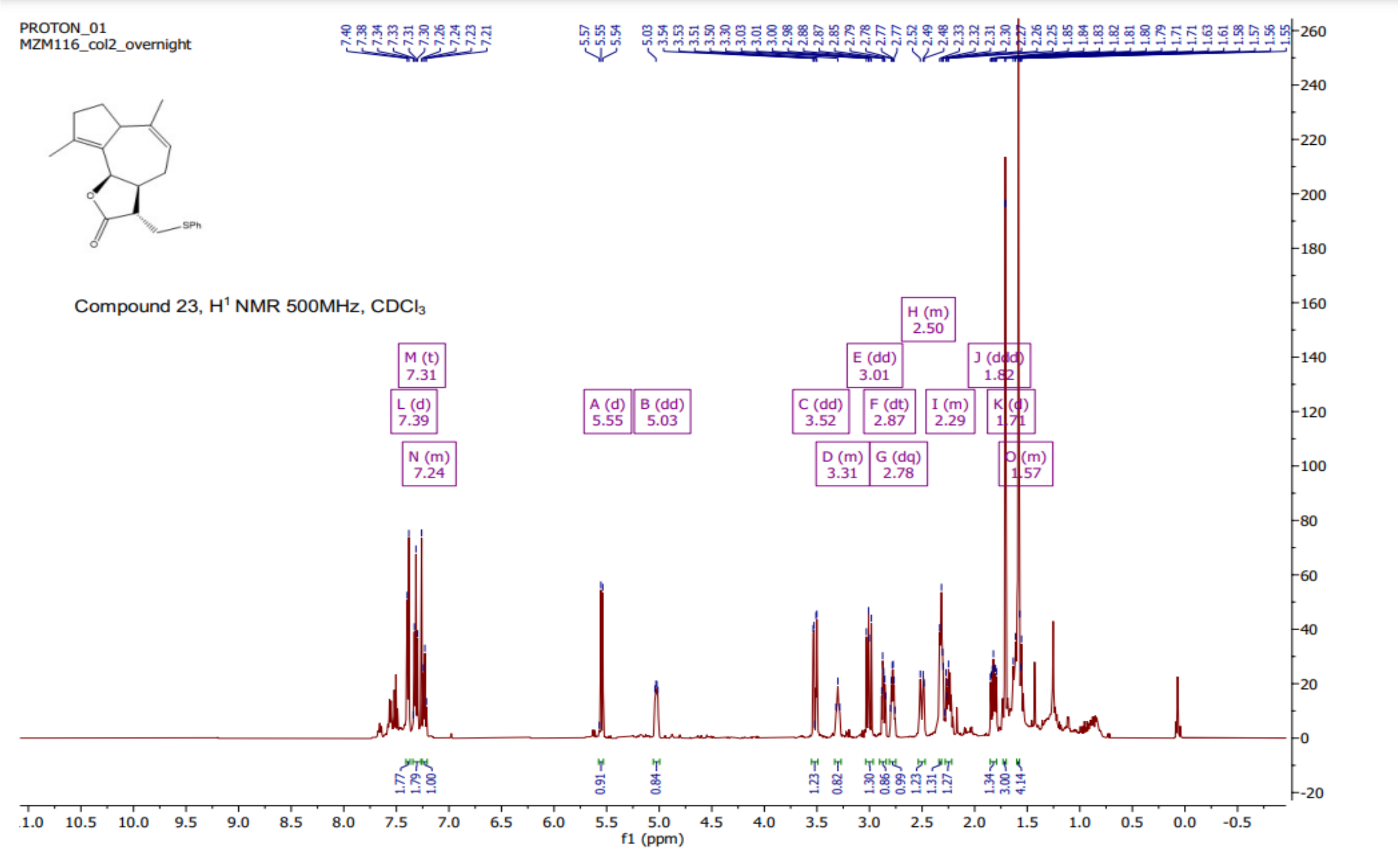
PROTON_01
MZM116_col2_overnight
Compound 23, H[1] NMR 500MHz, CDCl3
M (t) 7.31
L (d) 7.39
N (m) 7.24
A (d) 5.55
B (dd) 5.03
C (dd) 3.52
D (m) 3.31
E (dd) 3.01
F (dt) 2.87
G (dq) 2.78
H (m) 2.50
I (m) 2.29
J (ddd) 1.82
K (d) 1.71
O (m) 1.57
f1 (ppm)

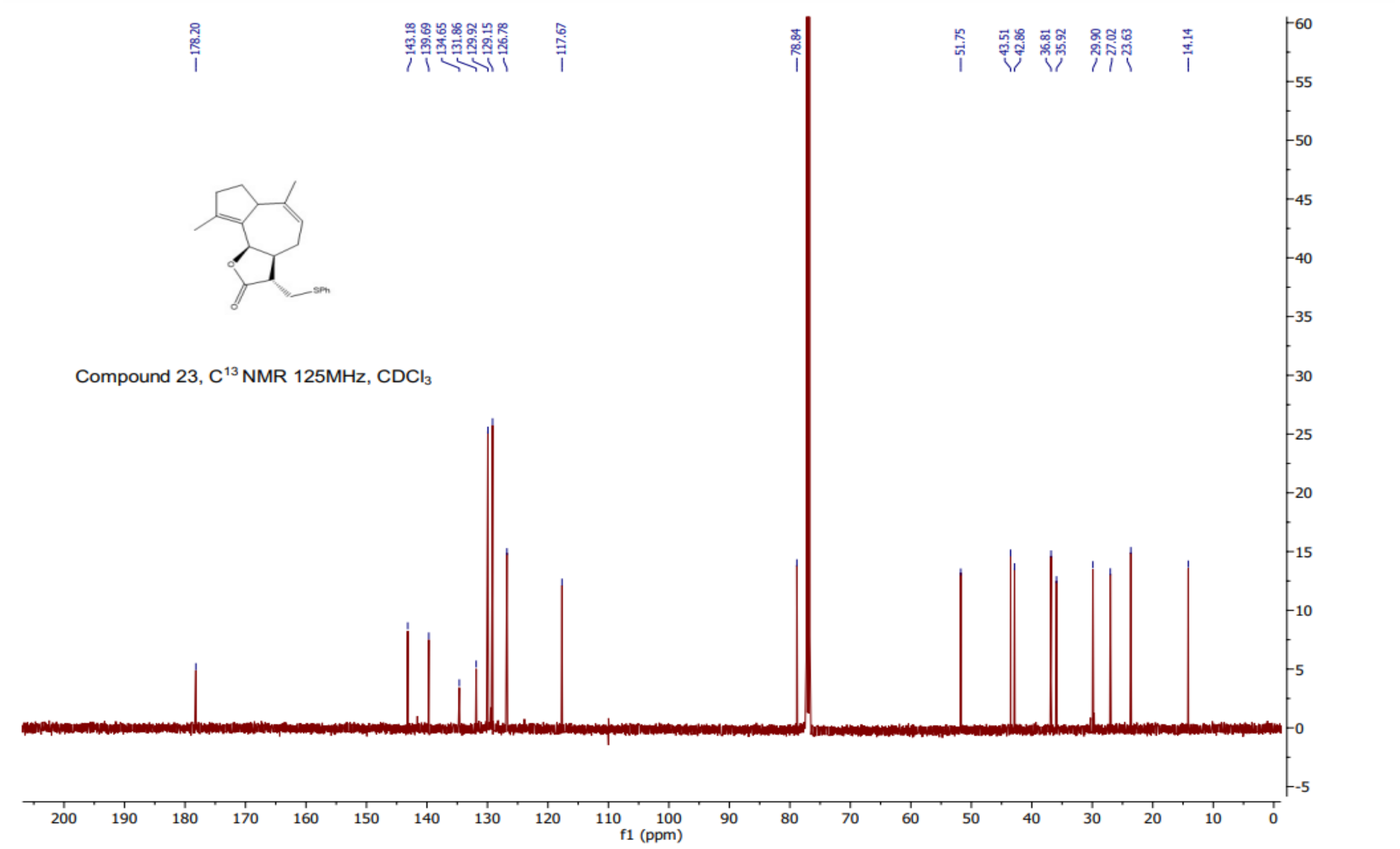
178.20
143.18
139.69
134.65
131.86
129.92
129.15
126.78
117.67
78.84
51.75
43.51
42.86
36.81
35.92
29.90
27.02
23.63
14.14
Compound 23, C[13] NMR 125MHz, CDCl3
f1 (ppm)

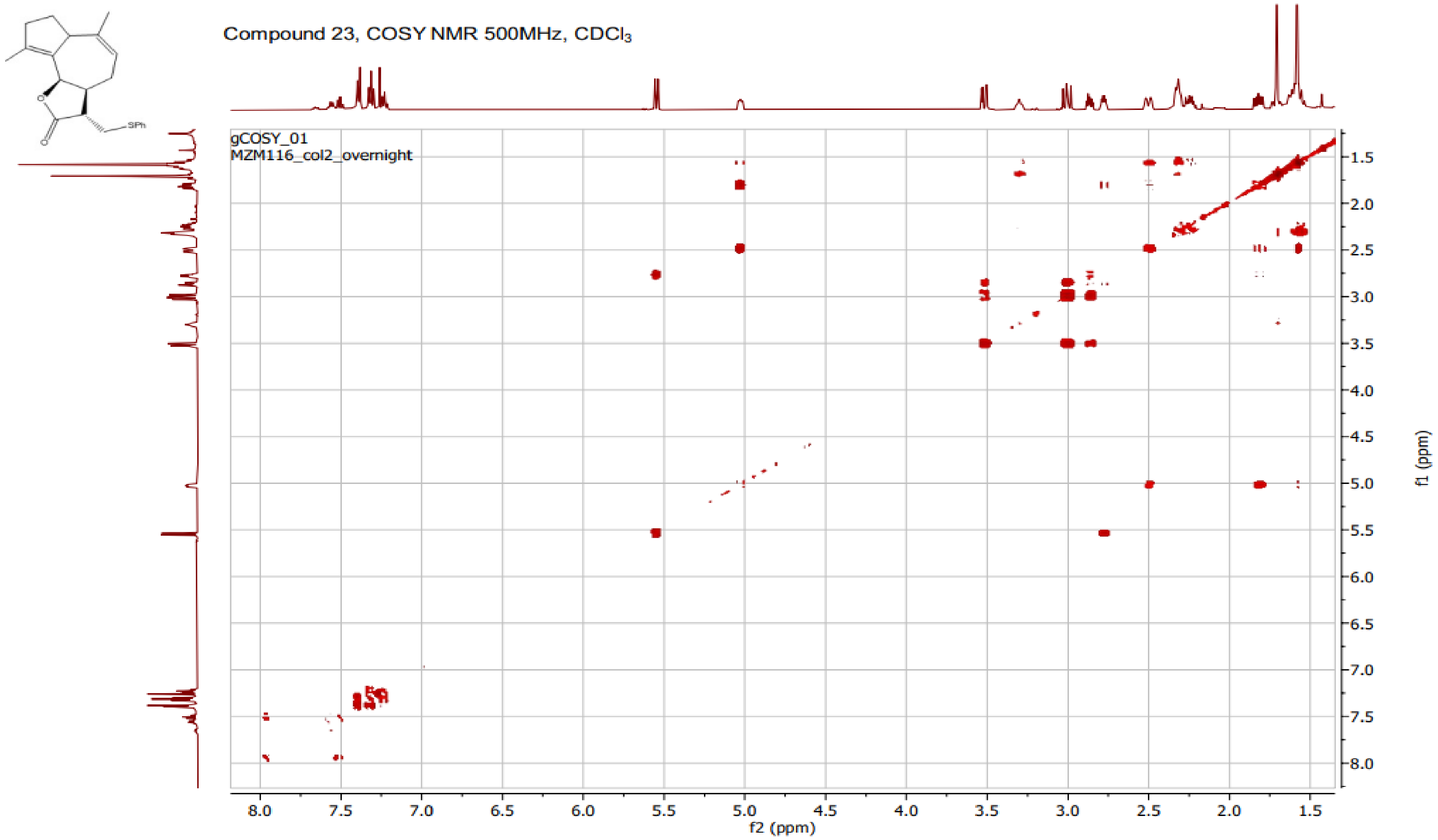

SPh
Compound 23, COSY NMR 500MHz, $CDCl_3$
gCOSY_01
MZM116_col2_overnight
f2 (ppm)
f1 (ppm)


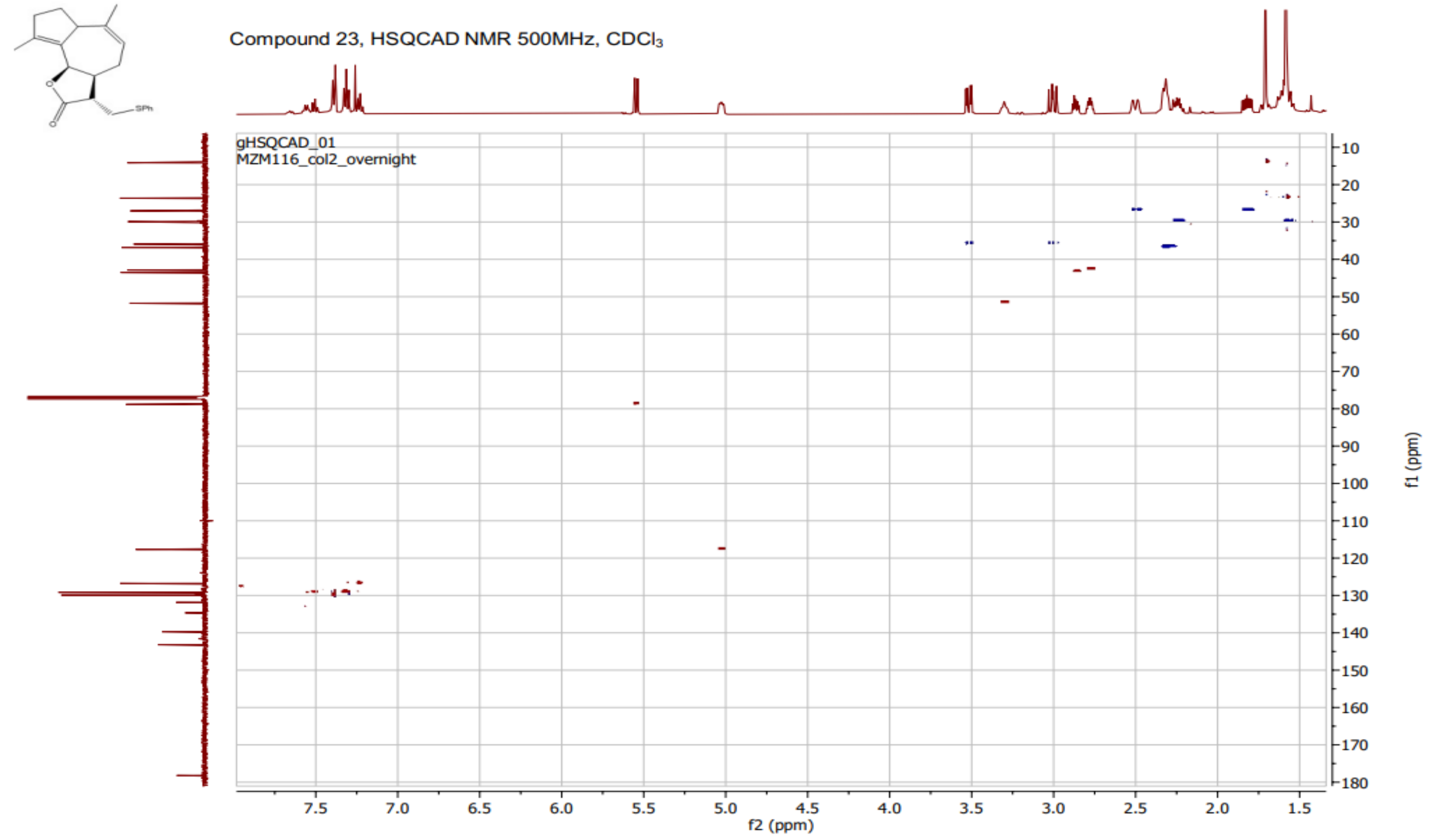

SPh
Compound 23, HSQCAD NMR 500MHz, $CDCl_3$
gHSQCAD_01
MZM116_col2_overnight
f2 (ppm)
f1 (ppm)

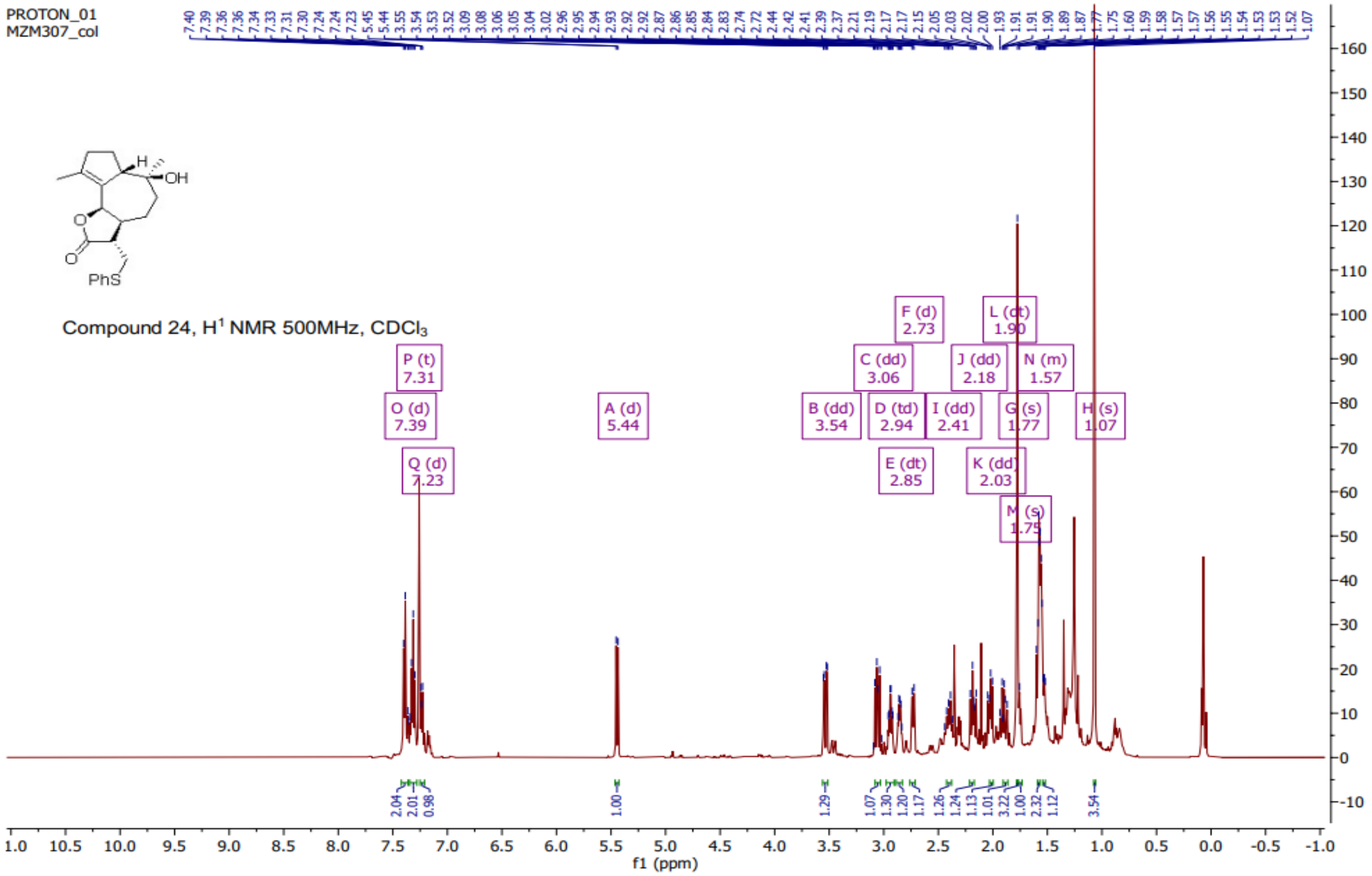
PROTON_01
MZM307_col
Compound 24, H[1] NMR 500MHz, CDCl3
f1 (ppm)

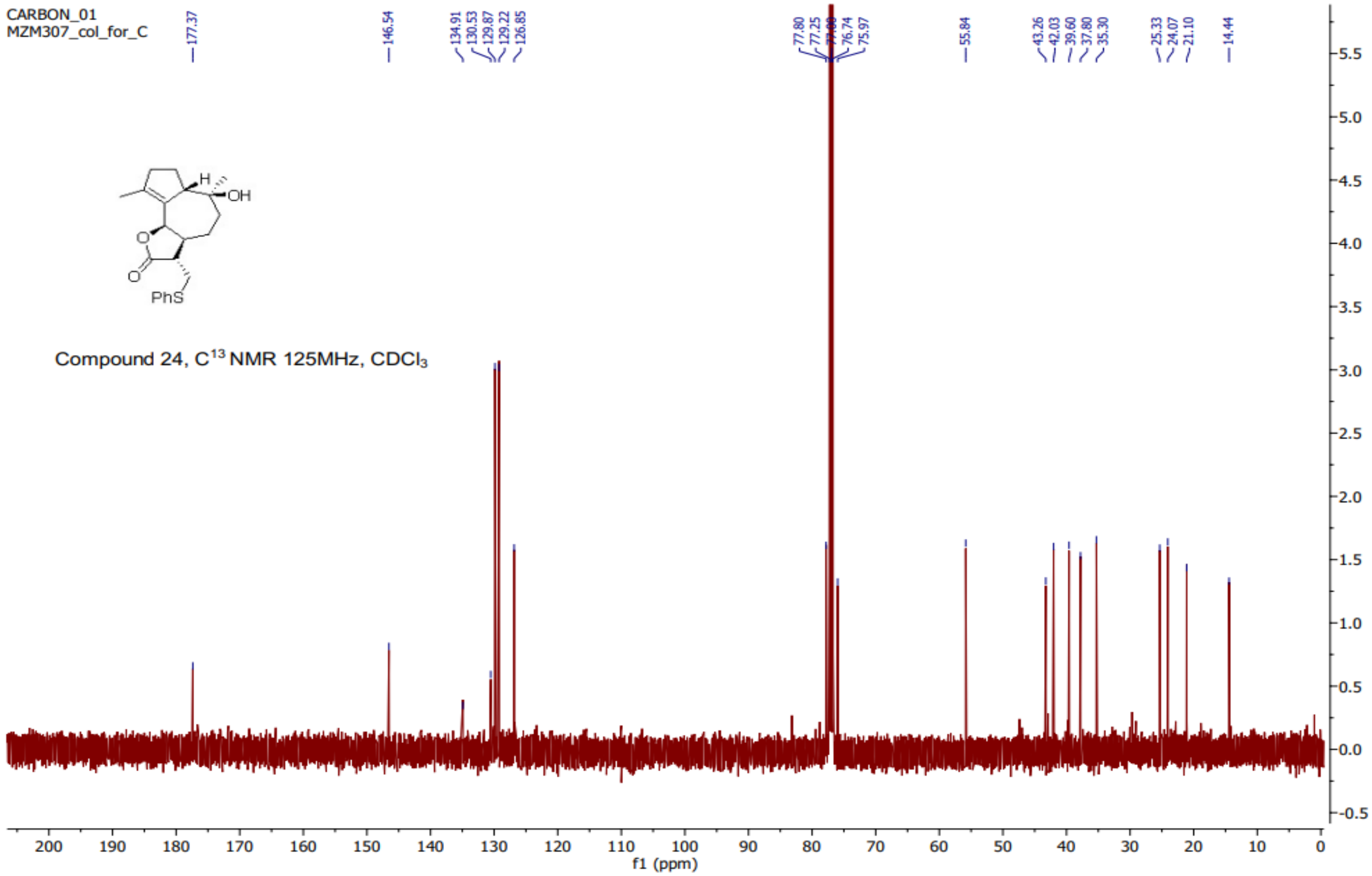
CARBON_01
MZM307_col_for_C
177.37
146.54
134.91
130.53
129.87
129.22
126.85
77.80
77.25
76.74
75.97
55.84
43.26
42.03
39.60
37.80
35.30
25.33
24.07
21.10
14.44
Compound 24, C[13] NMR 125MHz, CDCl3
f1 (ppm)

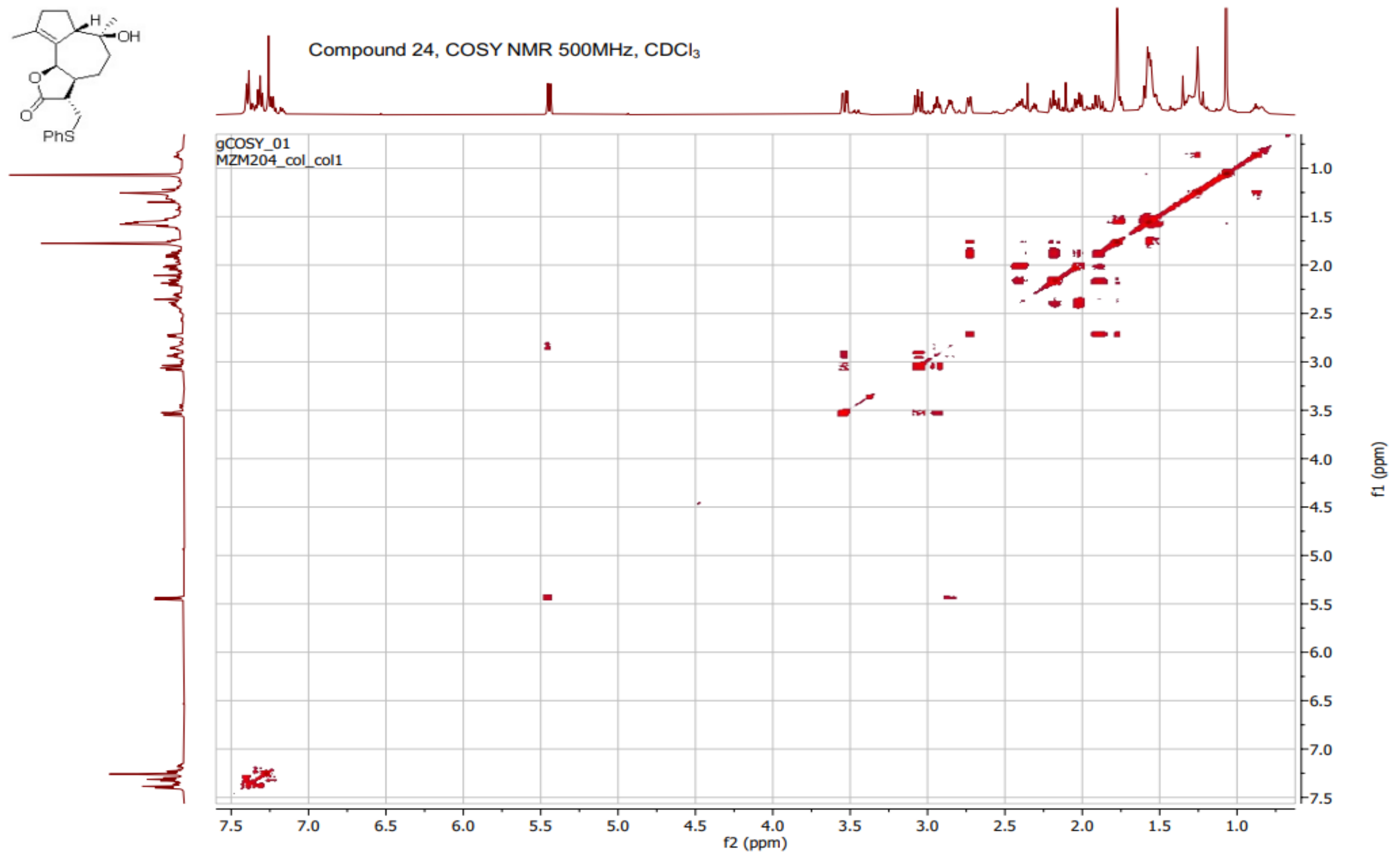
Compound 24, COSY NMR 500MHz, CDCl₃
gCOSY_01
MZM204_col_col1
f2 (ppm)
f1 (ppm)
PhS
OH
H

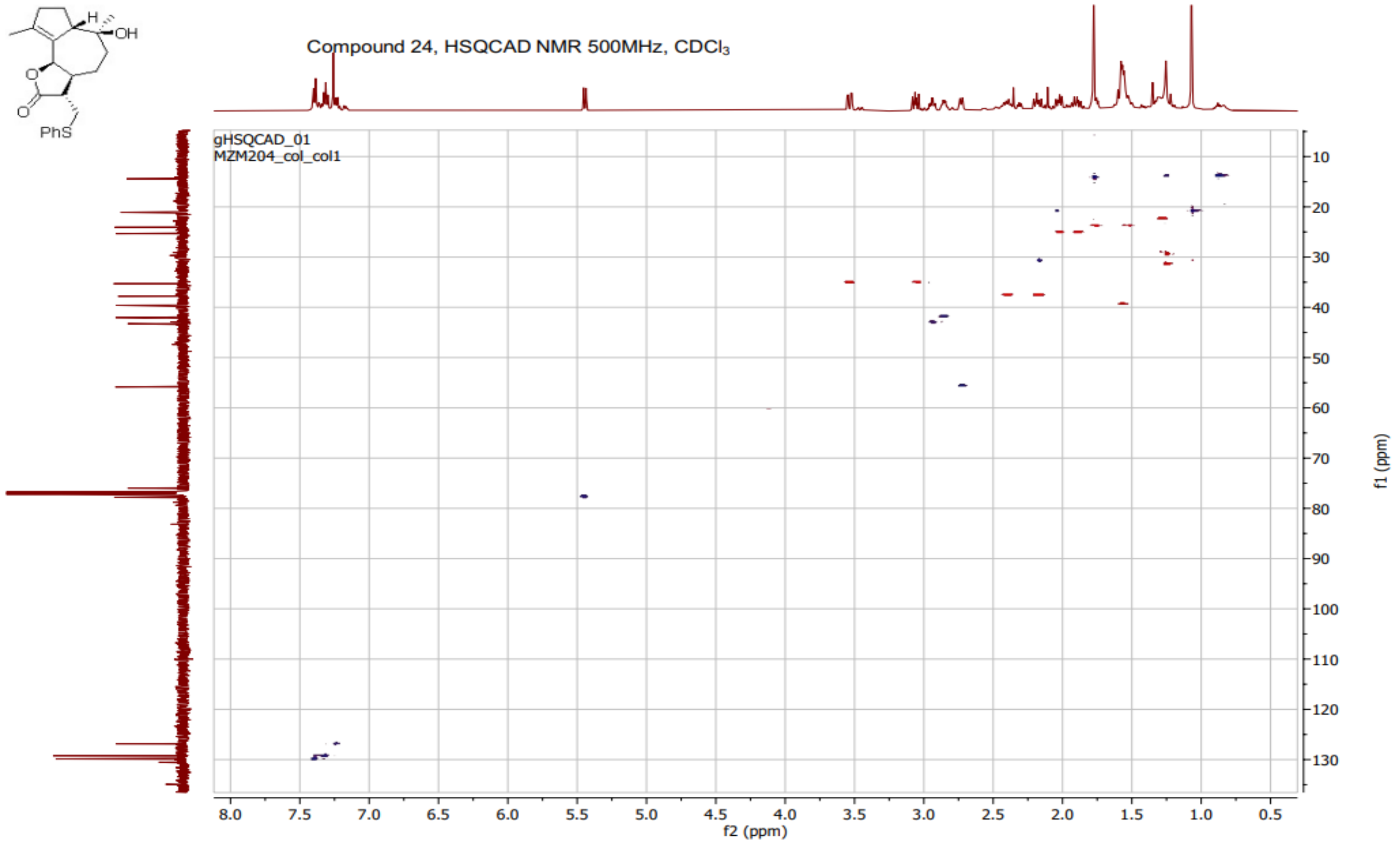
Compound 24, HSQCAD NMR 500MHz, CDCl₃
gHSQCAD_01
MZM204_col_col1
f2 (ppm)
f1 (ppm)
PhS
OH
H

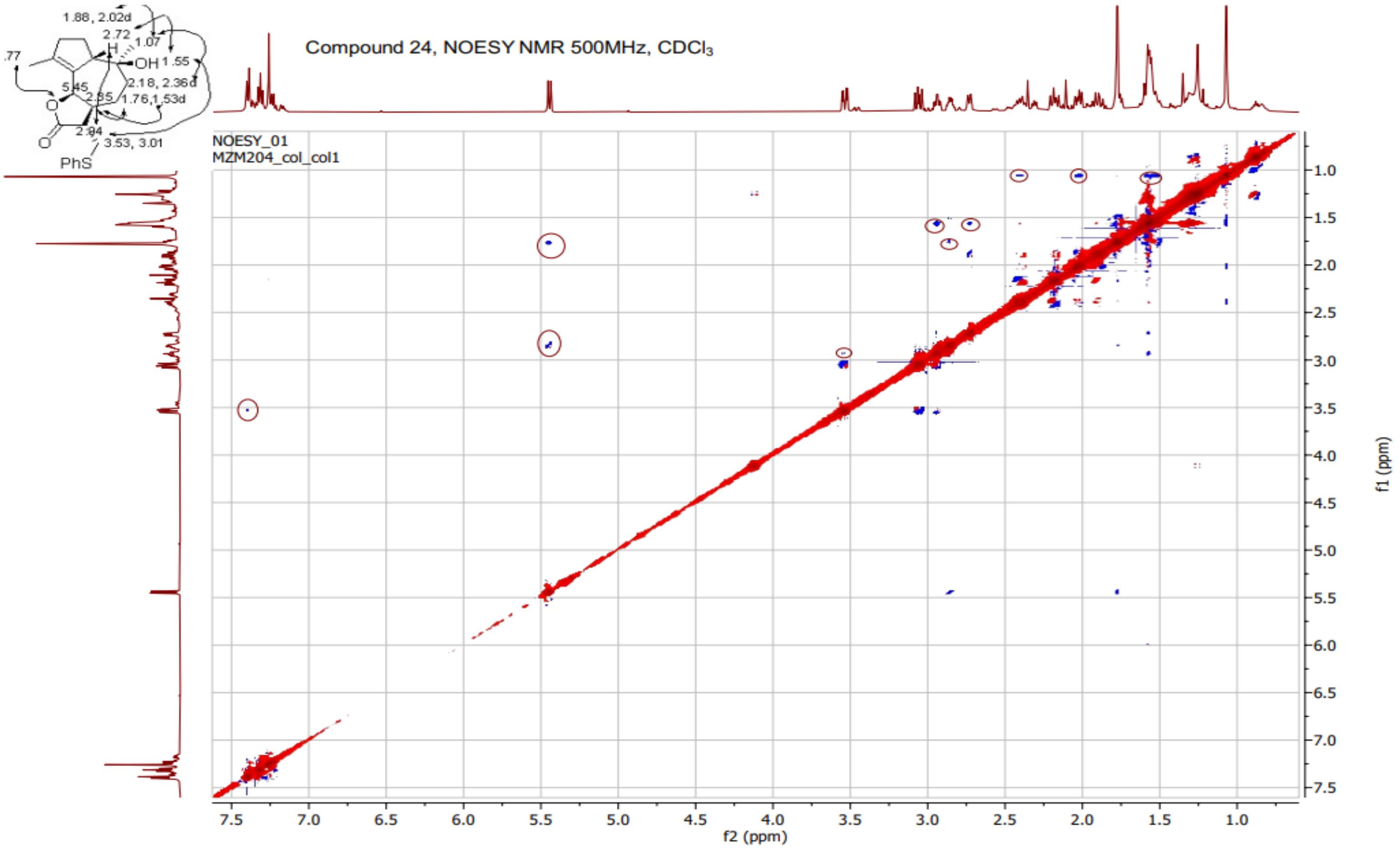
Compound 24, NOESY NMR 500MHz, CDCl3
NOESY_01
MZM204_col_col1
PhS
f2 (ppm)
f1 (ppm)

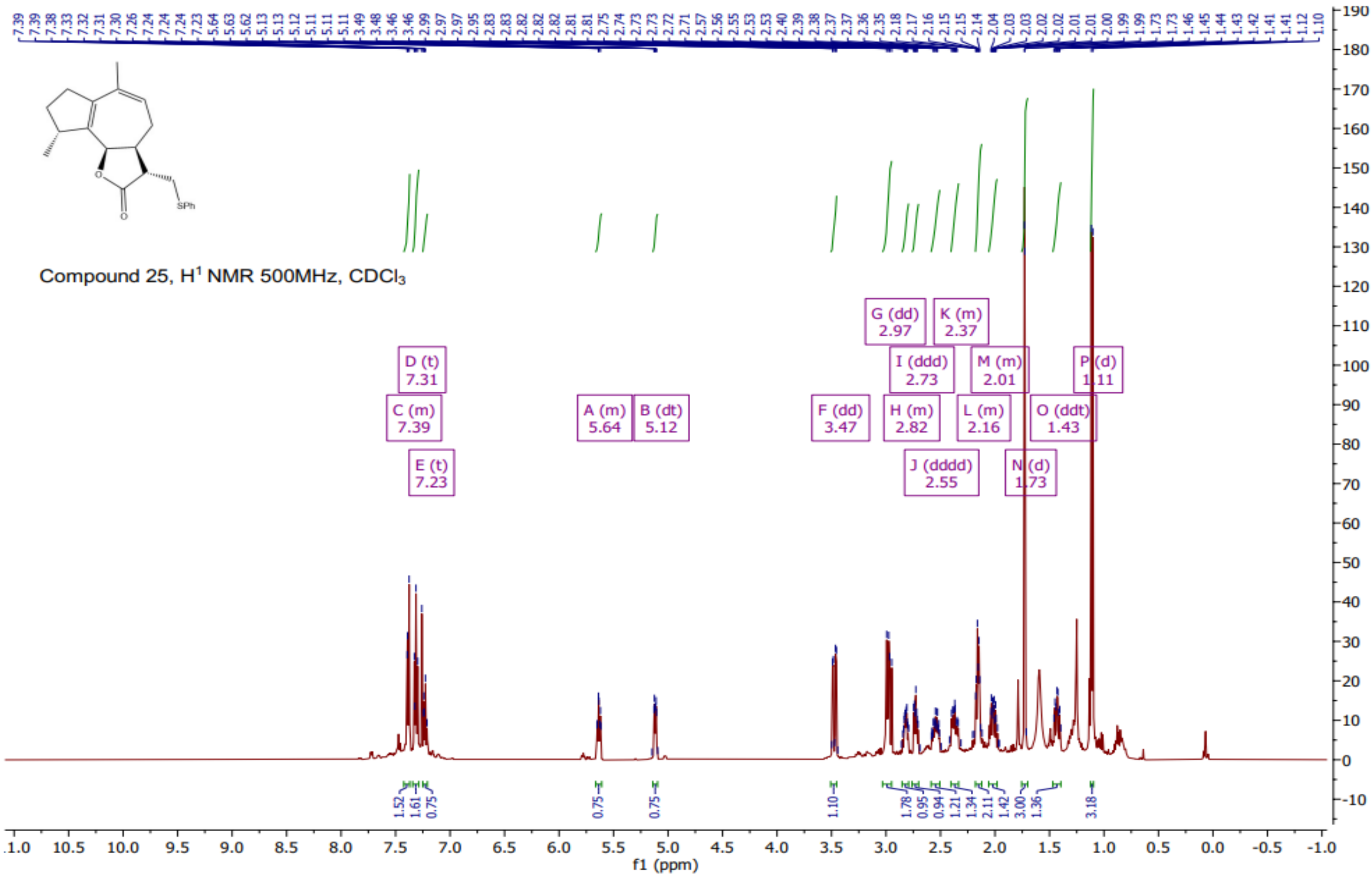
Compound 25, H¹ NMR 500MHz, CDCl3
SPh
A (m) 5.64
B (dt) 5.12
C (m) 7.39
D (t) 7.31
E (t) 7.23
F (dd) 3.47
G (dd) 2.97
H (m) 2.82
I (ddd) 2.73
J (dddd) 2.55
K (m) 2.37
L (m) 2.16
M (m) 2.01
N (d) 1.73
O (ddt) 1.43
P (d) 1.11
f1 (ppm)

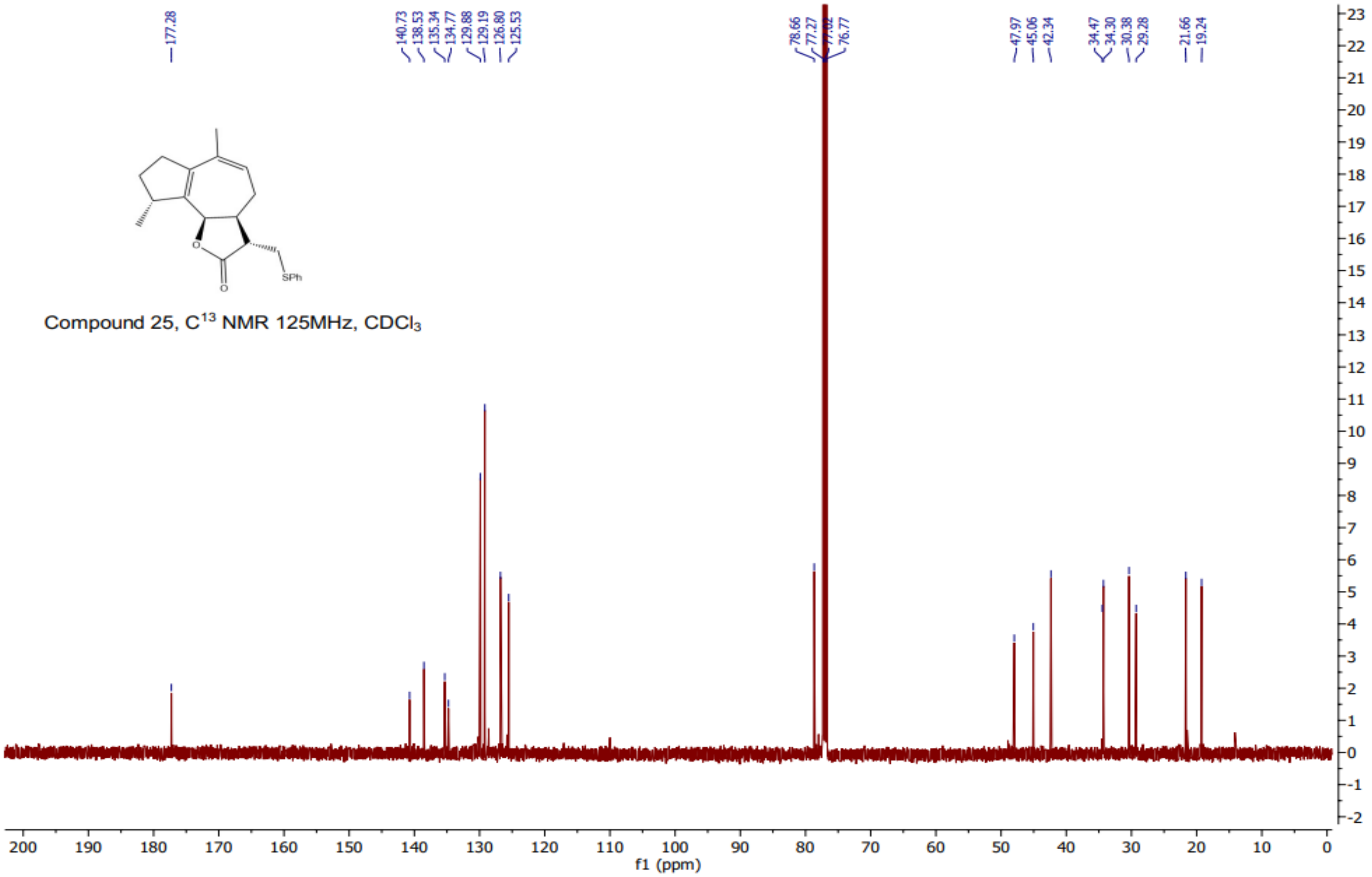
177.28
140.73
138.53
135.34
134.77
129.88
129.19
126.80
125.53
78.66
77.27
77.02
76.77
47.97
45.06
42.34
34.47
34.30
30.38
29.28
21.66
19.24
SPh
Compound 25, C13 NMR 125MHz, CDCl3
f1 (ppm)

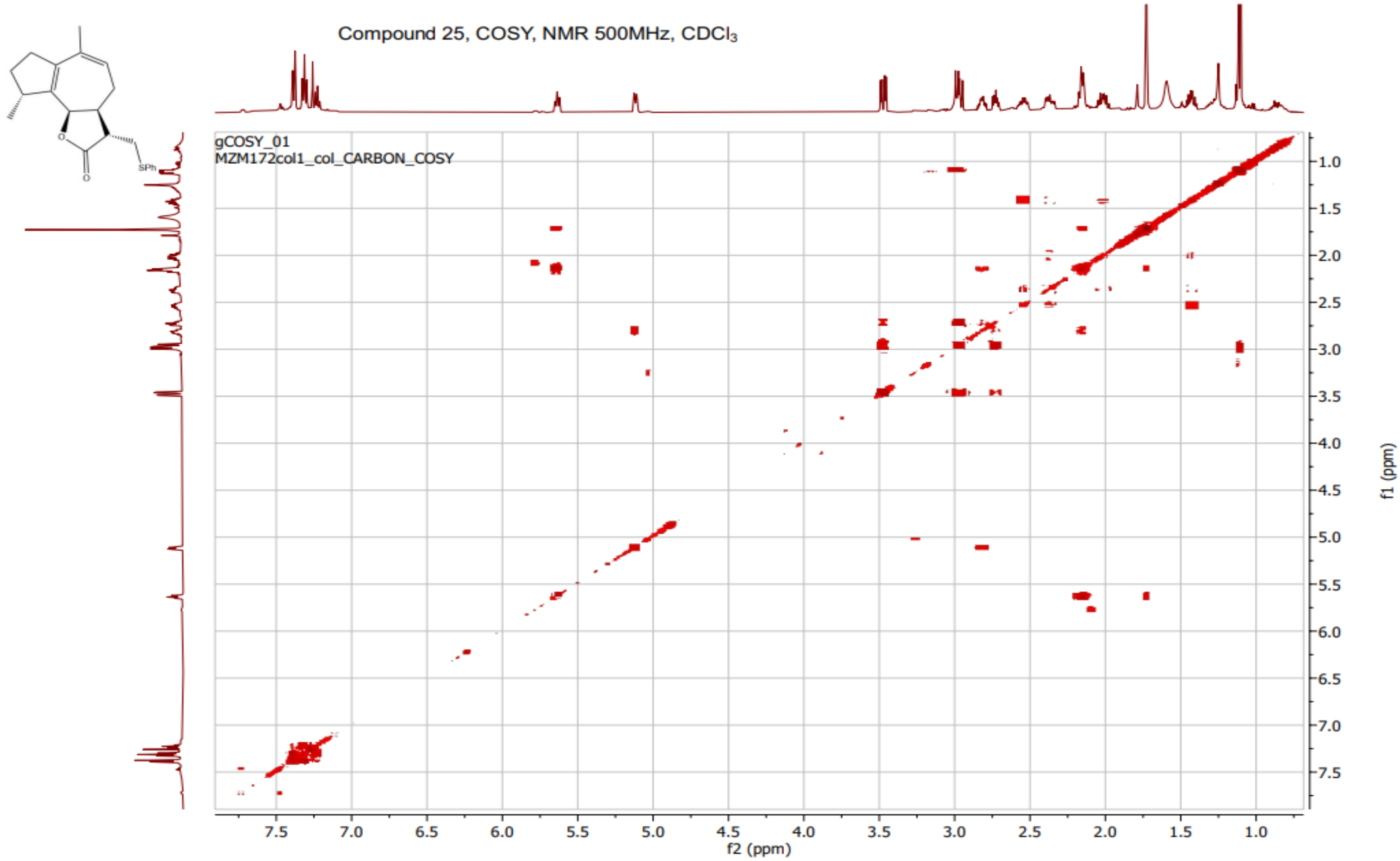
Compound 25, COSY, NMR 500MHz, CDCl3
SPh
gCOSY_01
MZM172col1_col_CARBON_COSY
f2 (ppm)
f1 (ppm)

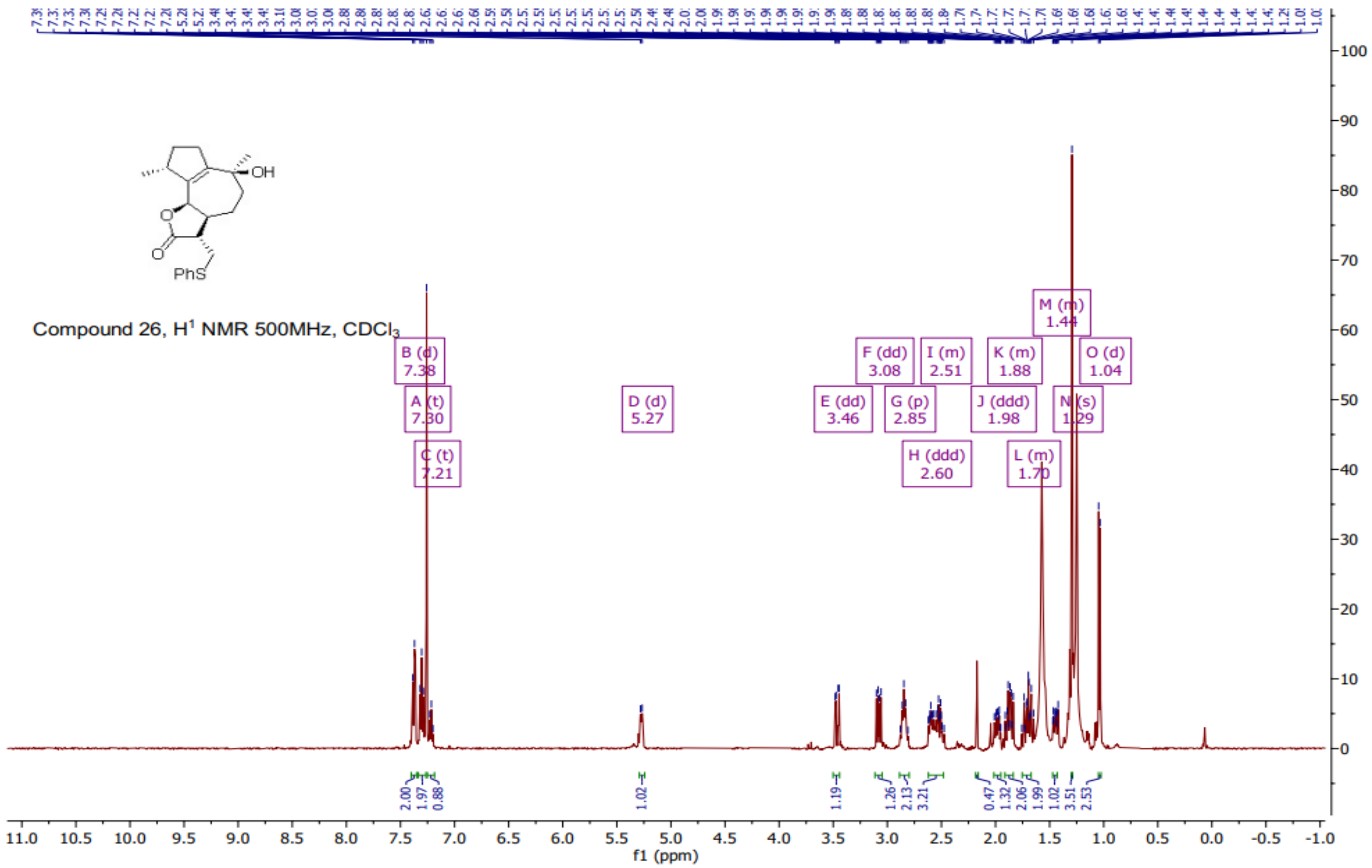


Compound 26, H[1] NMR 500MHz, $CDCl_3$

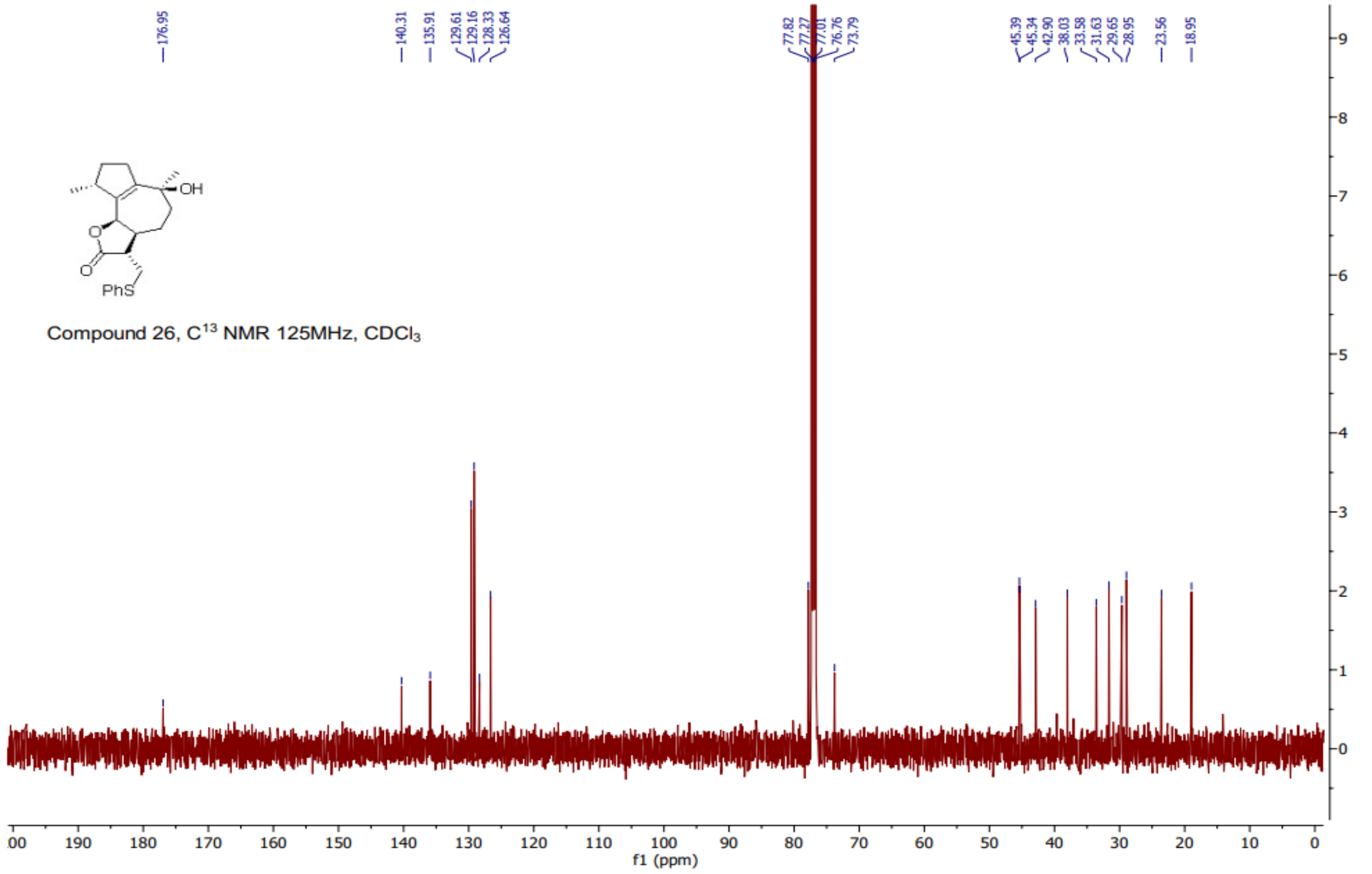


Compound 26, C[13] NMR 125MHz, $CDCl_3$

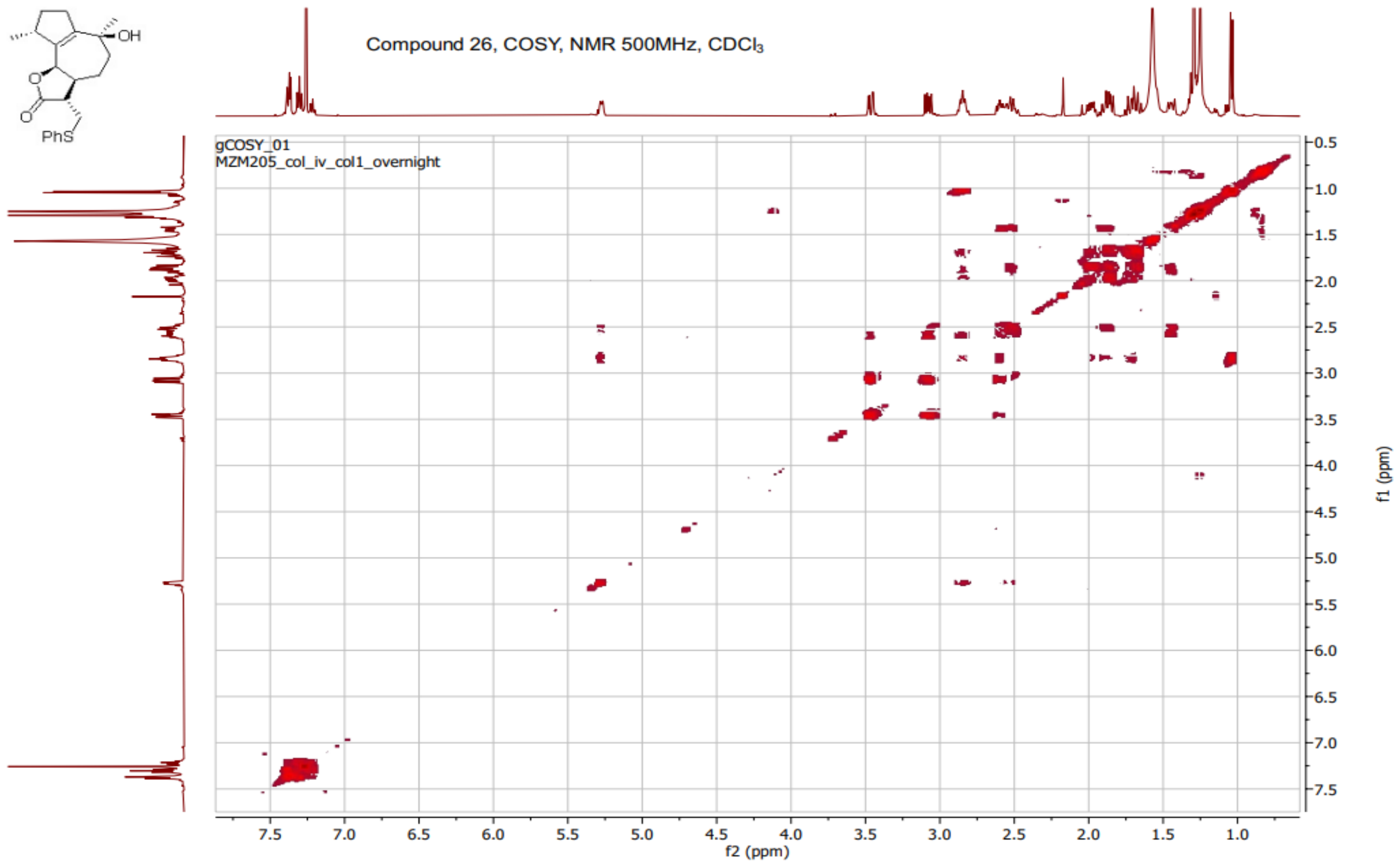
OH
O
O
PhS
Compound 26, COSY, NMR 500MHz, CDCl3
gCOSY_01
MZM205_col_iv_col1_overnight
f2 (ppm)
f1 (ppm)

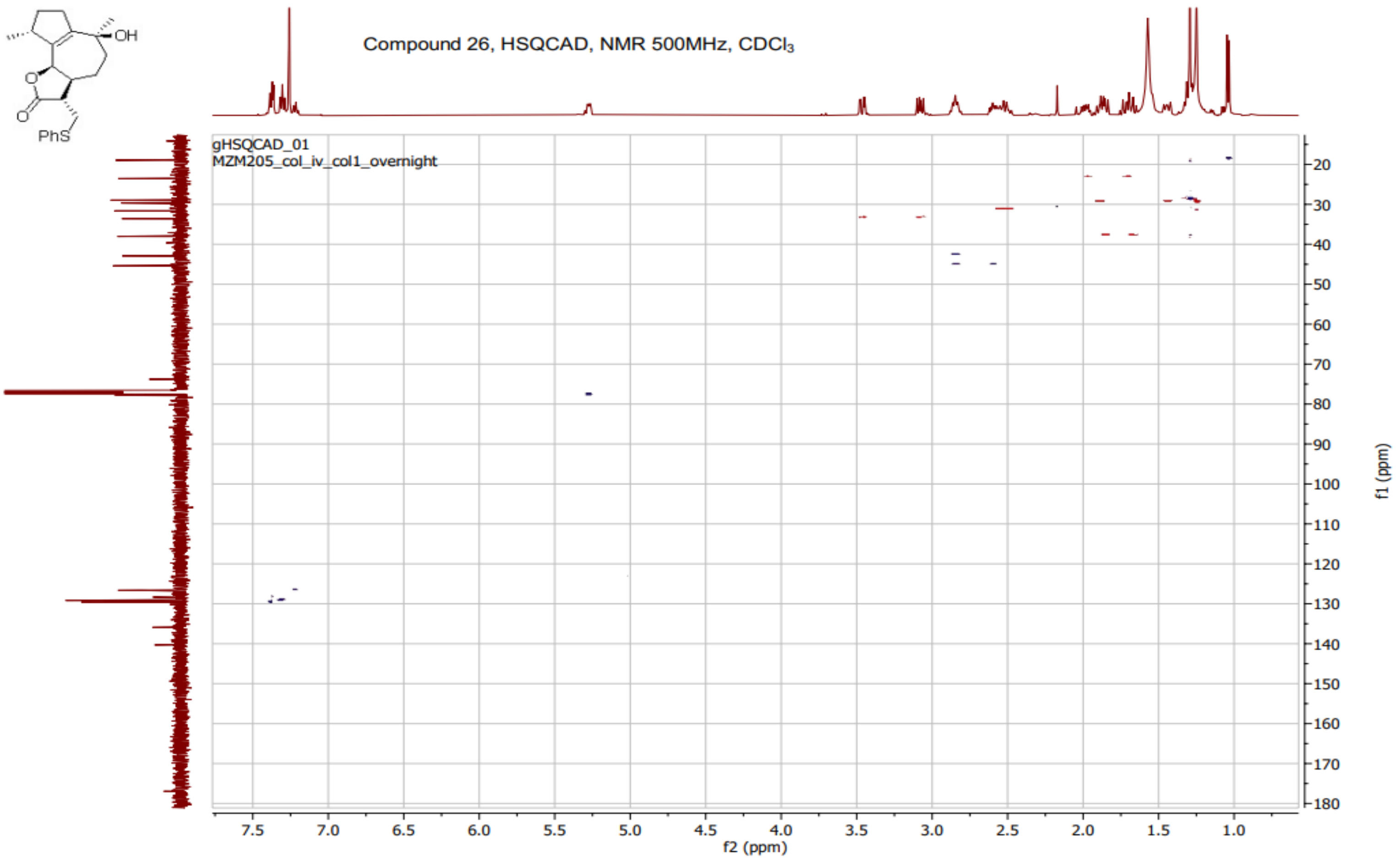
OH
O
O
PhS
Compound 26, HSQCAD, NMR 500MHz, CDCl3
gHSQCAD_01
MZM205_col_iv_col1_overnight
f2 (ppm)
f1 (ppm)

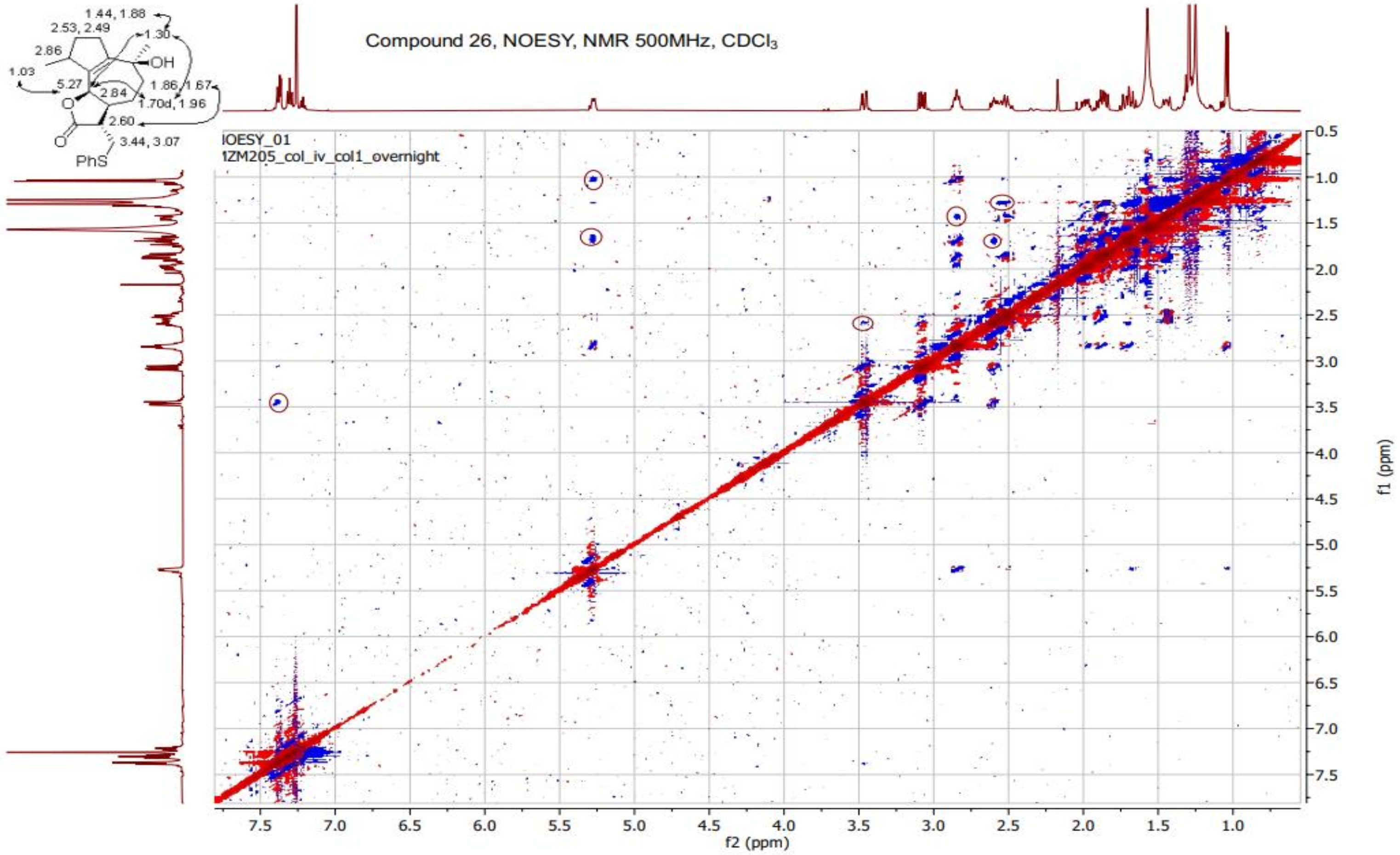
Compound 26, NOESY, NMR 500MHz, CDCl$_3$
NOESY_01
MZM205_col_iv_col1_overnight
f2 (ppm)
f1 (ppm)
PhS
OH

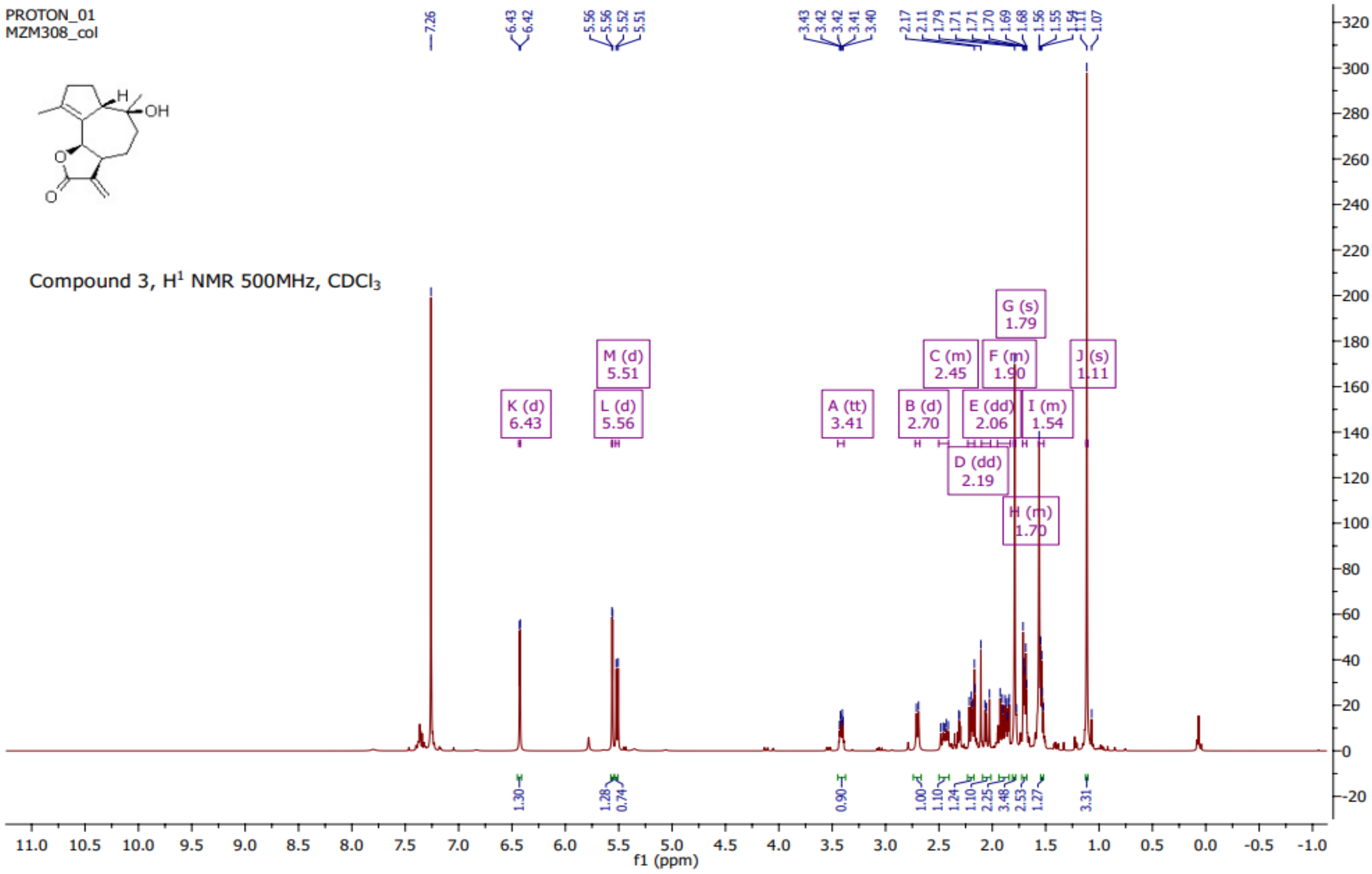
PROTON_01
MZM308_col
Compound 3, H$^1$ NMR 500MHz, CDCl$_3$
OH
f1 (ppm)
K (d) 6.43
L (d) 5.56
M (d) 5.51
A (tt) 3.41
B (d) 2.70
C (m) 2.45
D (dd) 2.19
E (dd) 2.06
F (m) 1.90
G (s) 1.79
H (m) 1.70
I (m) 1.54
J (s) 1.11

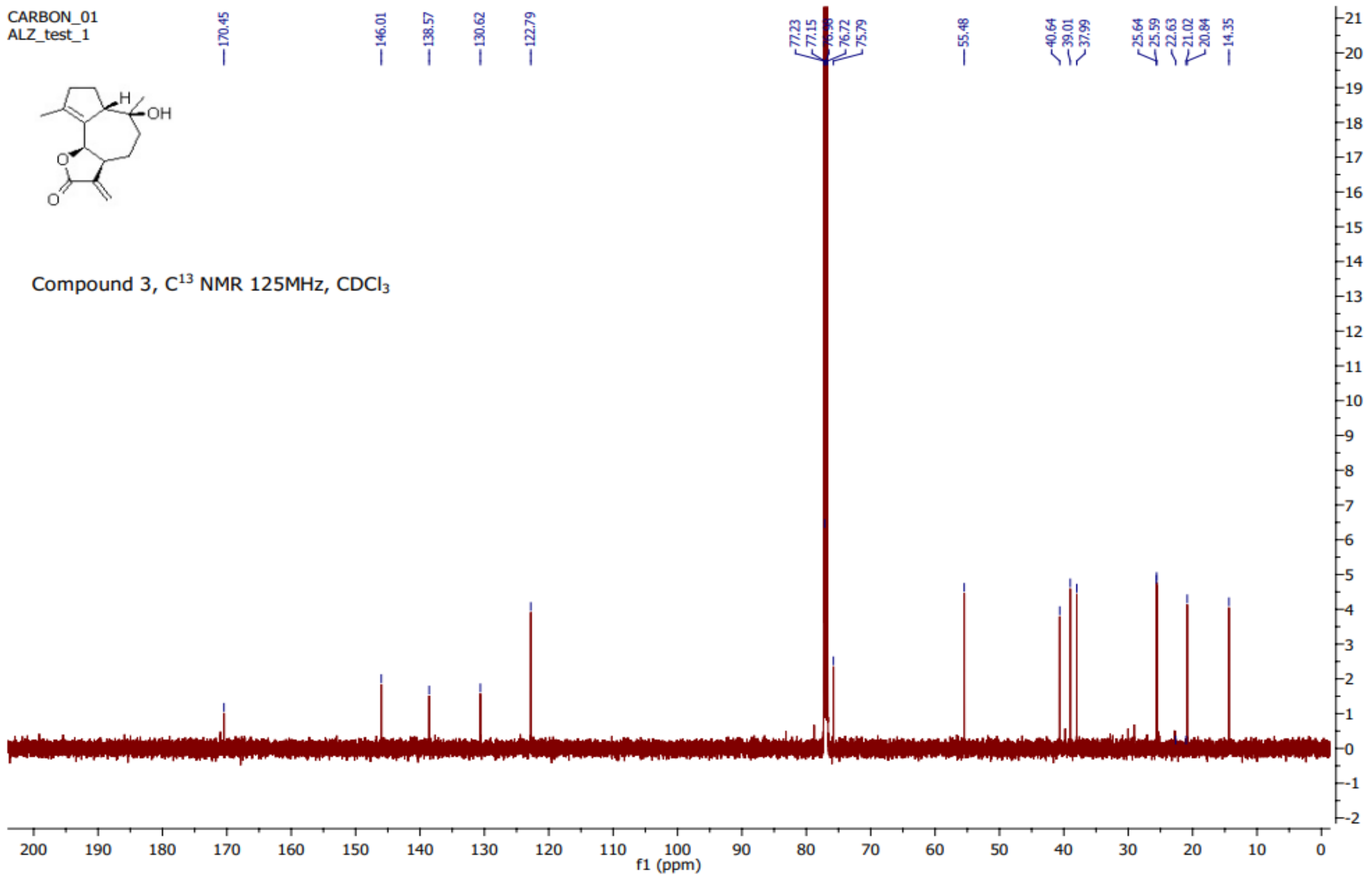
CARBON_01
ALZ_test_1
OH
O
O
H
Compound 3, C13 NMR 125MHz, CDCl3
170.45
146.01
138.57
130.62
122.79
77.23
77.15
76.72
75.79
55.48
40.64
39.01
37.99
25.64
25.59
22.63
21.02
20.84
14.35
f1 (ppm)

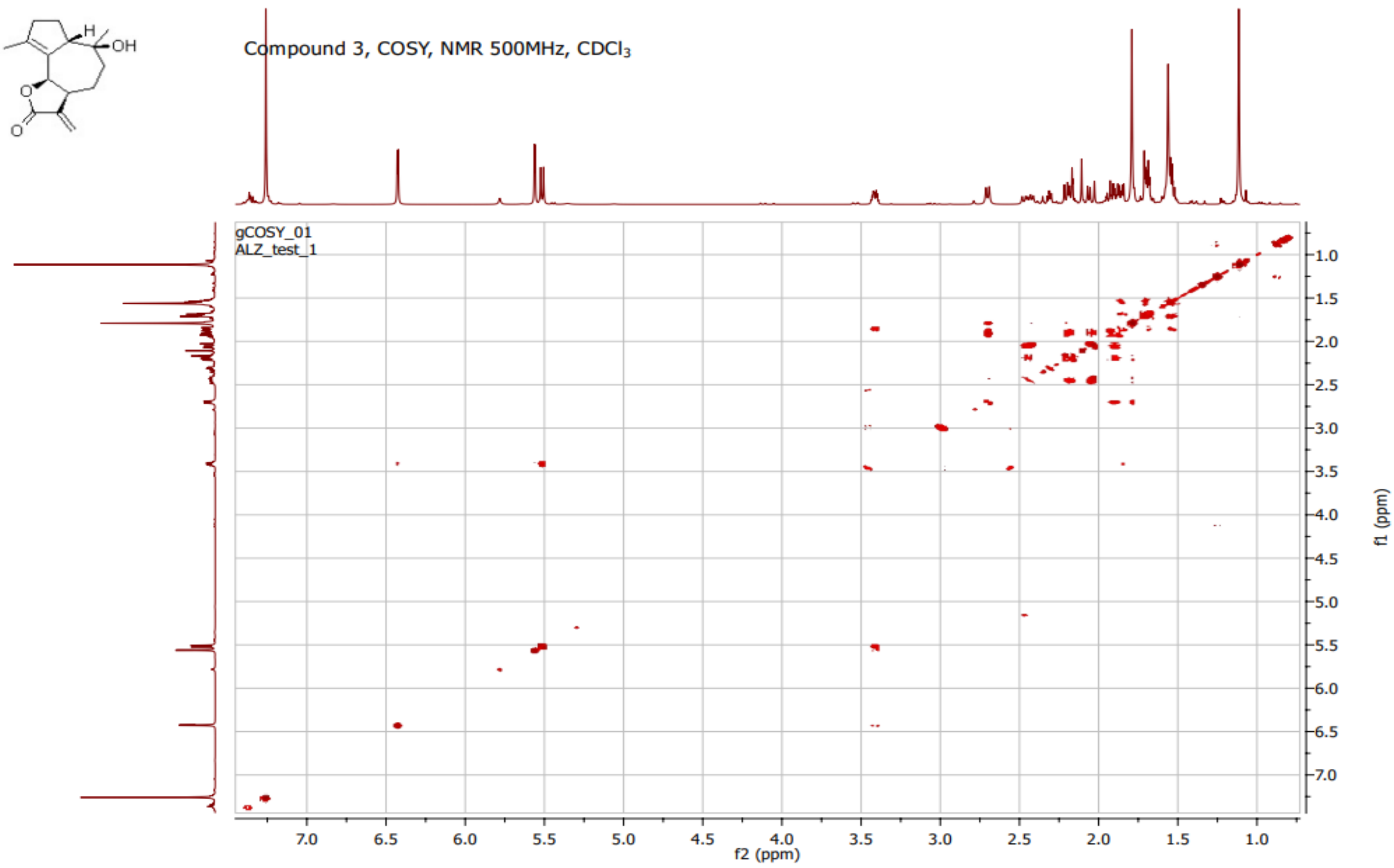
Compound 3, COSY, NMR 500MHz, CDCl3
gCOSY_01
ALZ_test_1
OH
O
O
H
f2 (ppm)
f1 (ppm)

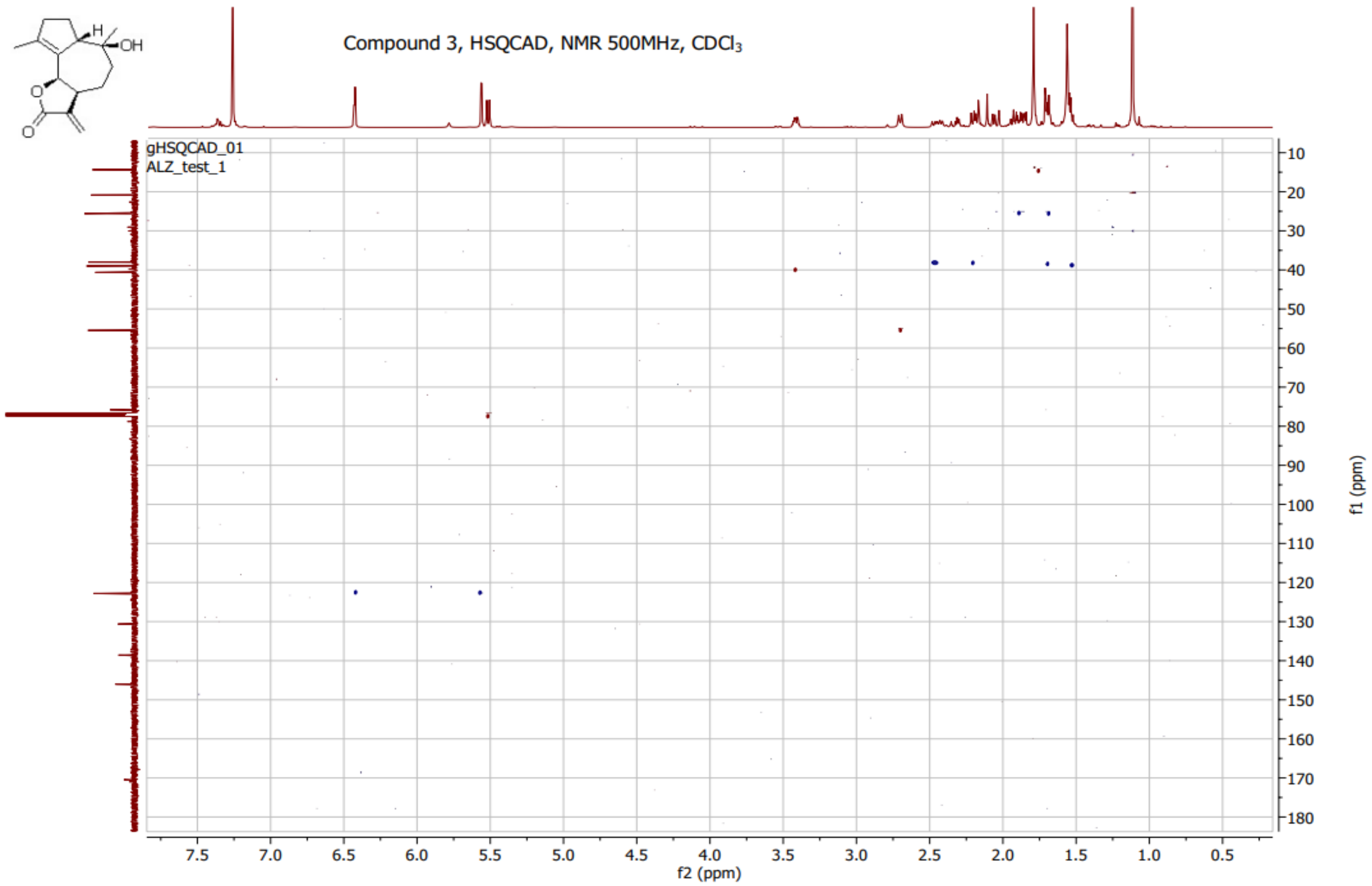

OH
O
O
Compound 3, HSQCAD, NMR 500MHz, CDCl3
gHSQCAD_01
ALZ_test_1
f2 (ppm)
f1 (ppm)


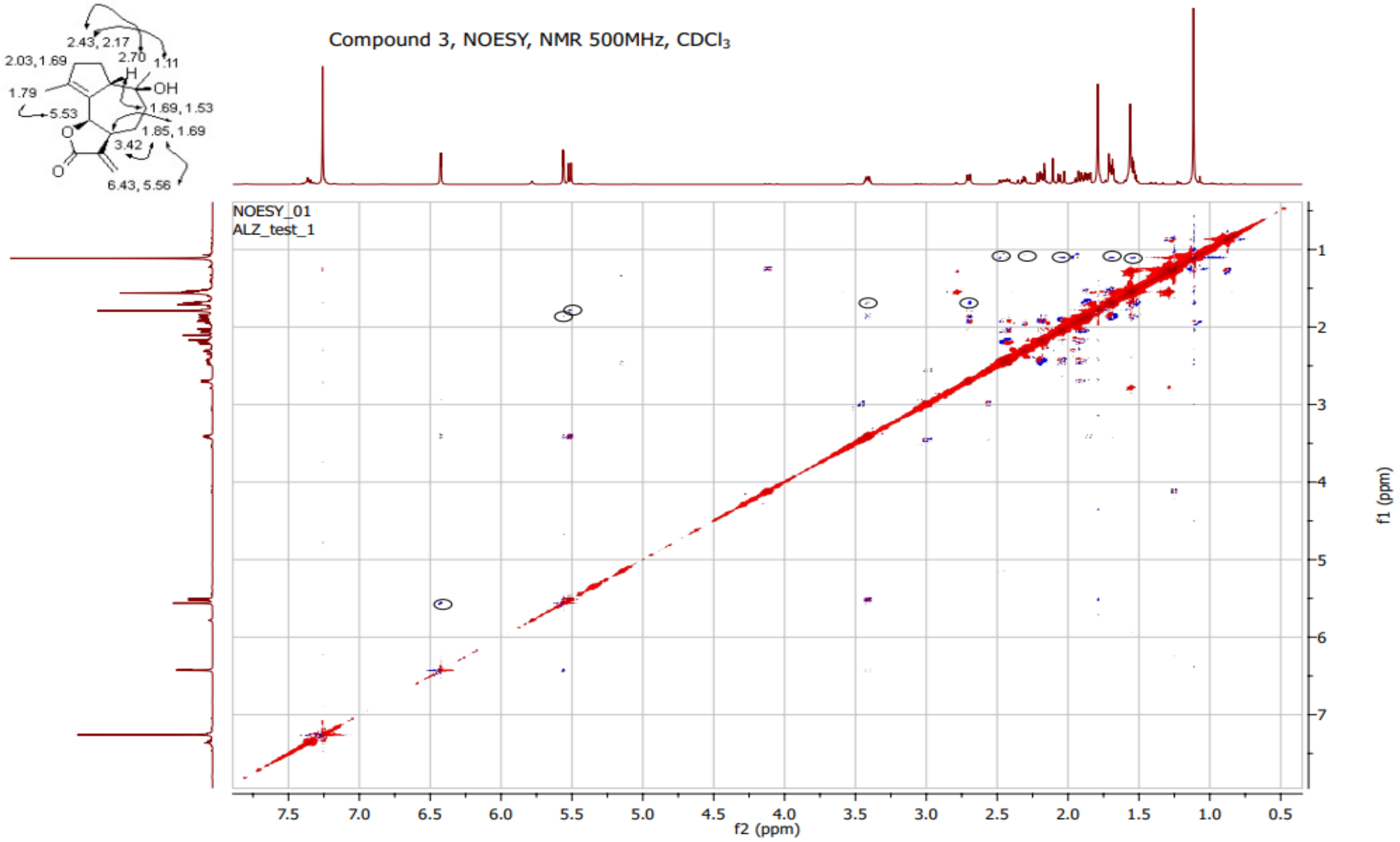

2.43, 2.17
2.70
1.11
2.03, 1.69
1.79
OH
5.53
1.69, 1.53
1.85, 1.69
3.42
6.43, 5.56
Compound 3, NOESY, NMR 500MHz, CDCl3
NOESY_01
ALZ_test_1
f2 (ppm)
f1 (ppm)

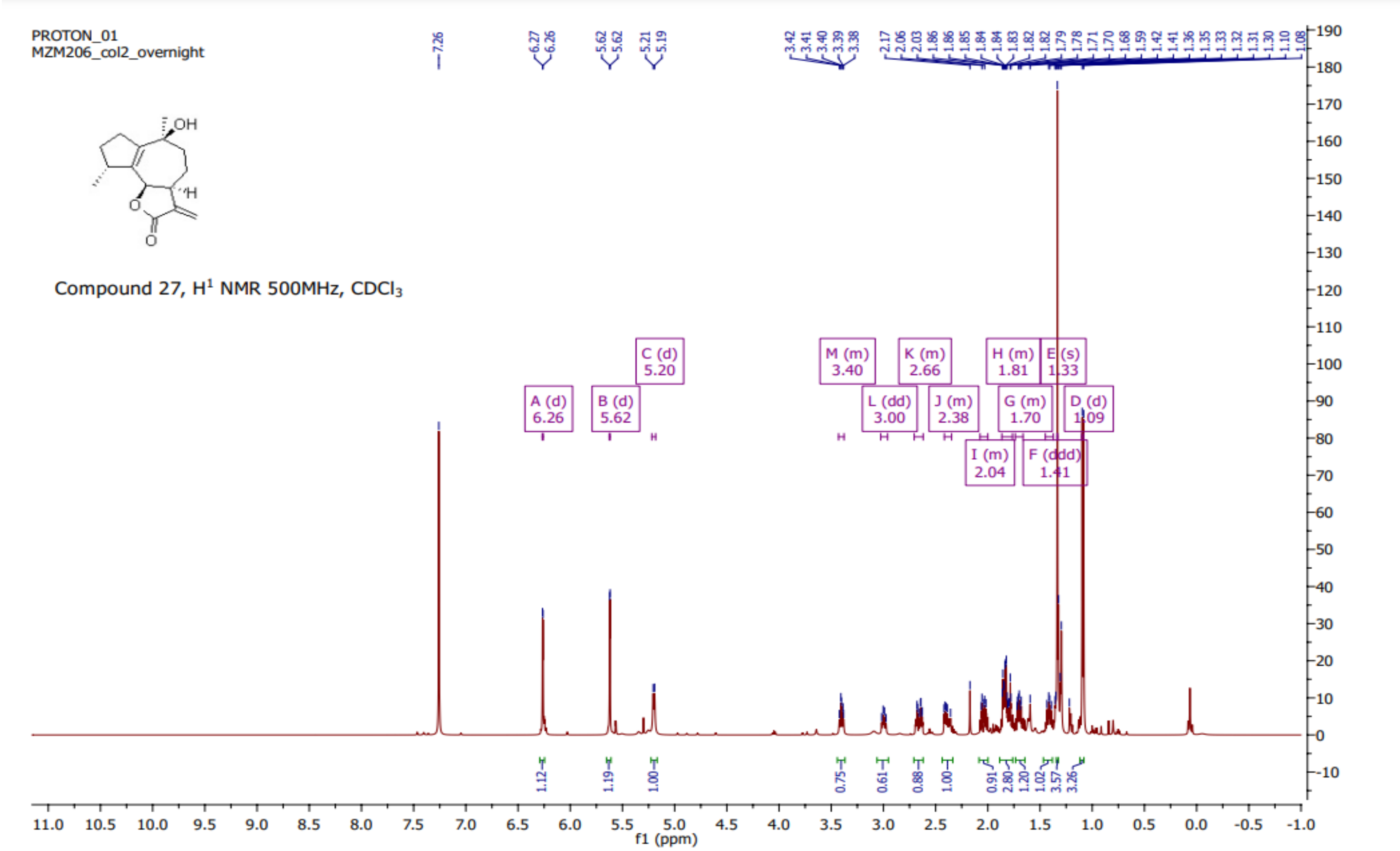
PROTON_01
MZM206_col2_overnight
OH
H
O
O
Compound 27, H1 NMR 500MHz, CDCl3
7.26
6.27
6.26
5.62
5.62
5.21
5.19
3.42
3.41
3.40
3.39
3.38
2.17
2.06
2.03
1.86
1.86
1.85
1.84
1.84
1.83
1.82
1.82
1.79
1.78
1.71
1.70
1.68
1.59
1.42
1.41
1.36
1.35
1.33
1.32
1.31
1.30
1.10
1.08
A (d) 6.26
B (d) 5.62
C (d) 5.20
M (m) 3.40
L (dd) 3.00
K (m) 2.66
J (m) 2.38
I (m) 2.04
H (m) 1.81
G (m) 1.70
F (ddd) 1.41
E (s) 1.33
D (d) 1.09
1.12
1.19
1.00
0.75
0.61
0.88
1.00
0.91
2.80
1.20
1.02
3.57
3.26
f1 (ppm)

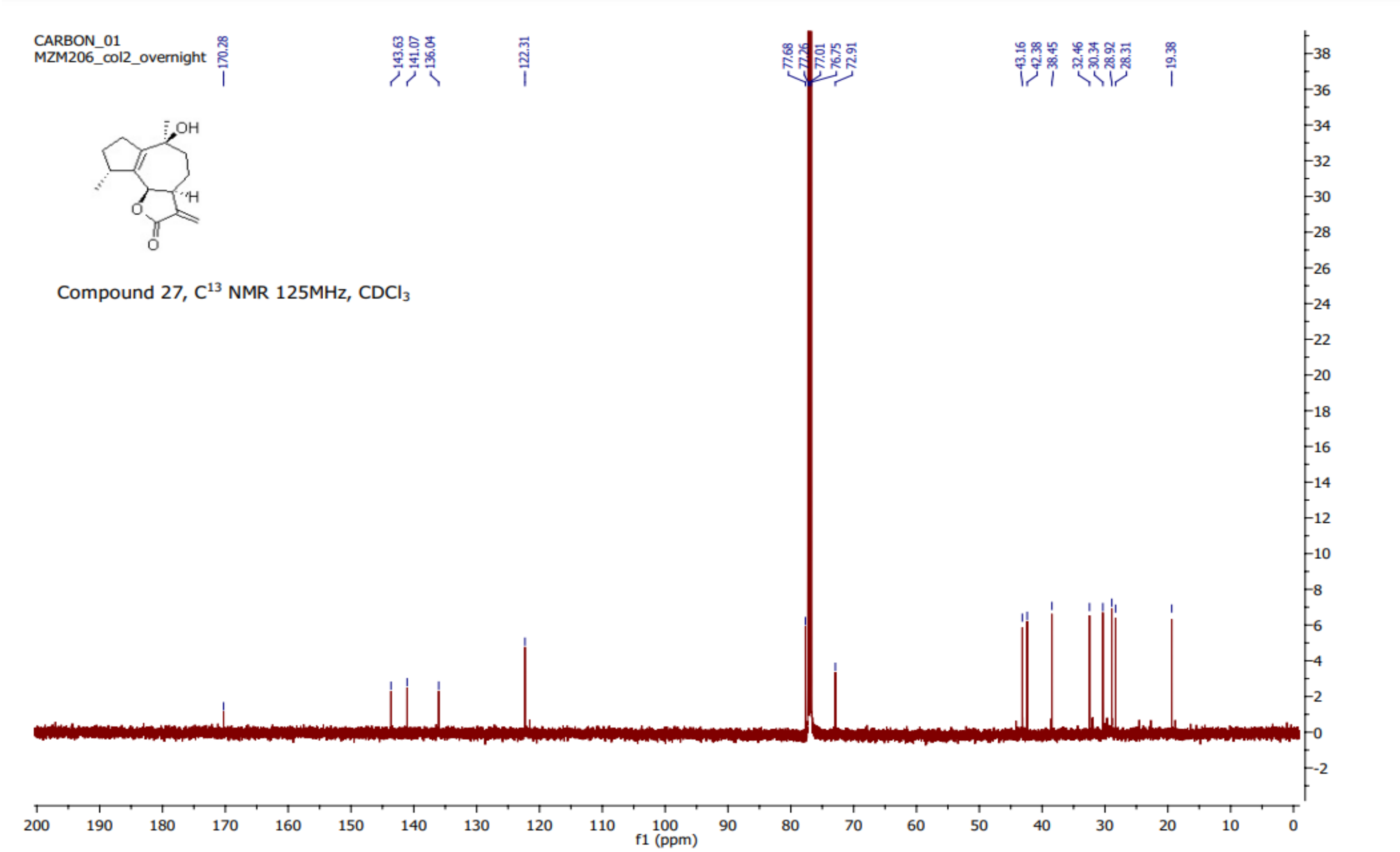
CARBON_01
MZM206_col2_overnight
170.28
143.63
141.07
136.04
122.31
77.68
77.26
77.01
76.75
72.91
43.16
42.38
38.45
32.46
30.34
28.92
28.31
19.38
OH
H
O
O
Compound 27, C13 NMR 125MHz, CDCl3
f1 (ppm)

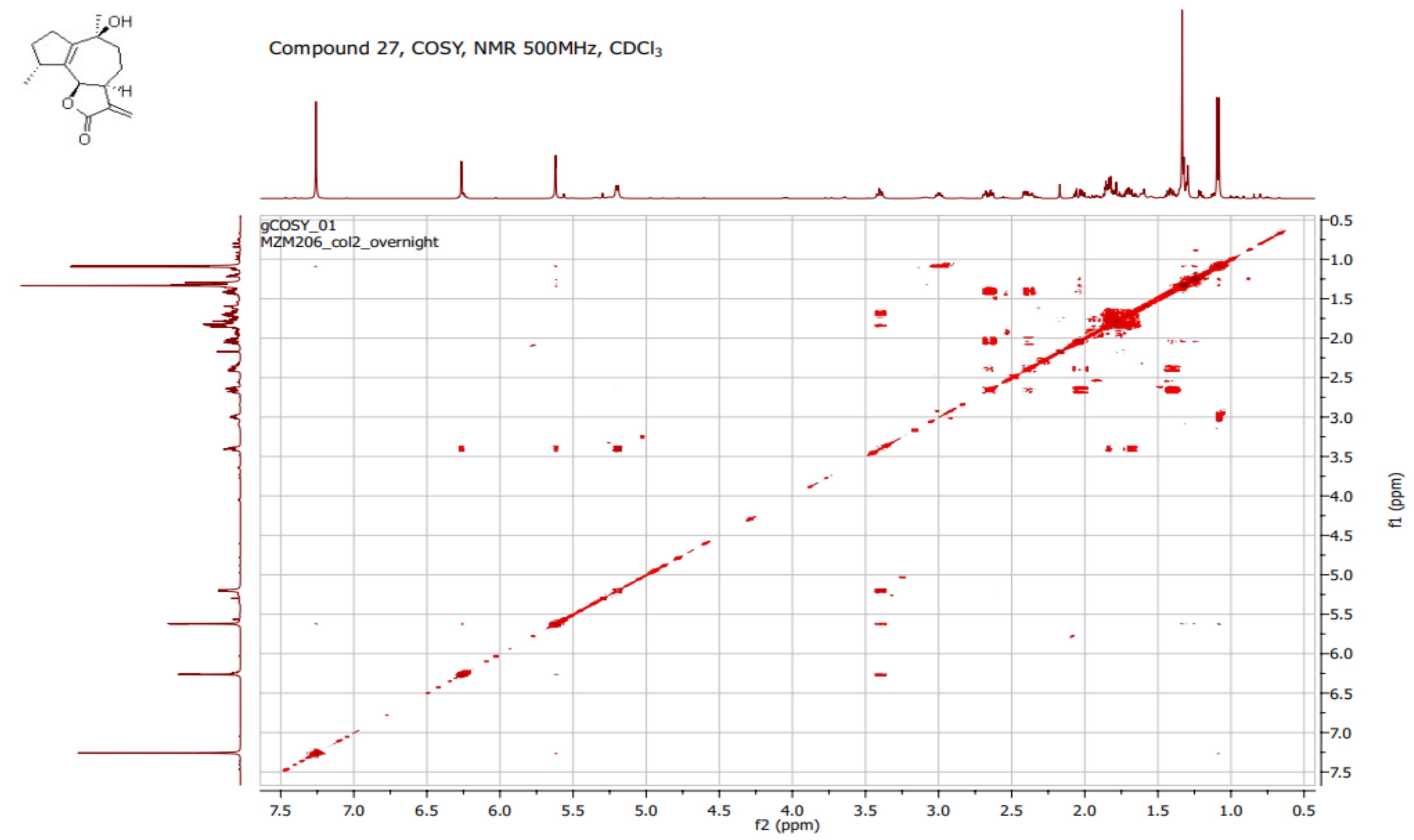

OH
H
O
O
Compound 27, COSY, NMR 500MHz, CDCl3
gCOSY_01
MZM206_col2_overnight
f2 (ppm)
f1 (ppm)


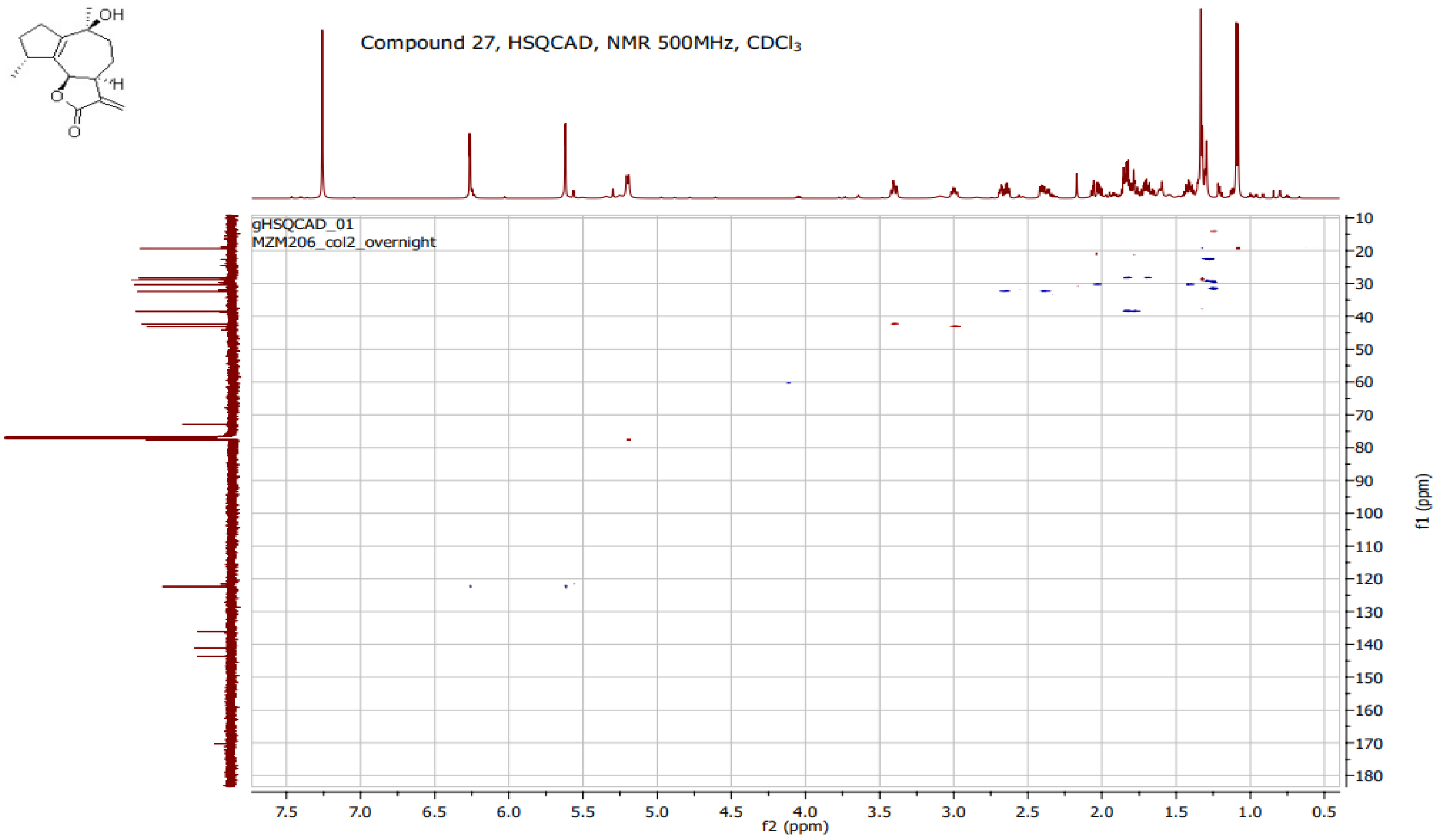

OH
H
O
O
Compound 27, HSQCAD, NMR 500MHz, CDCl3
gHSQCAD_01
MZM206_col2_overnight
f2 (ppm)
f1 (ppm)

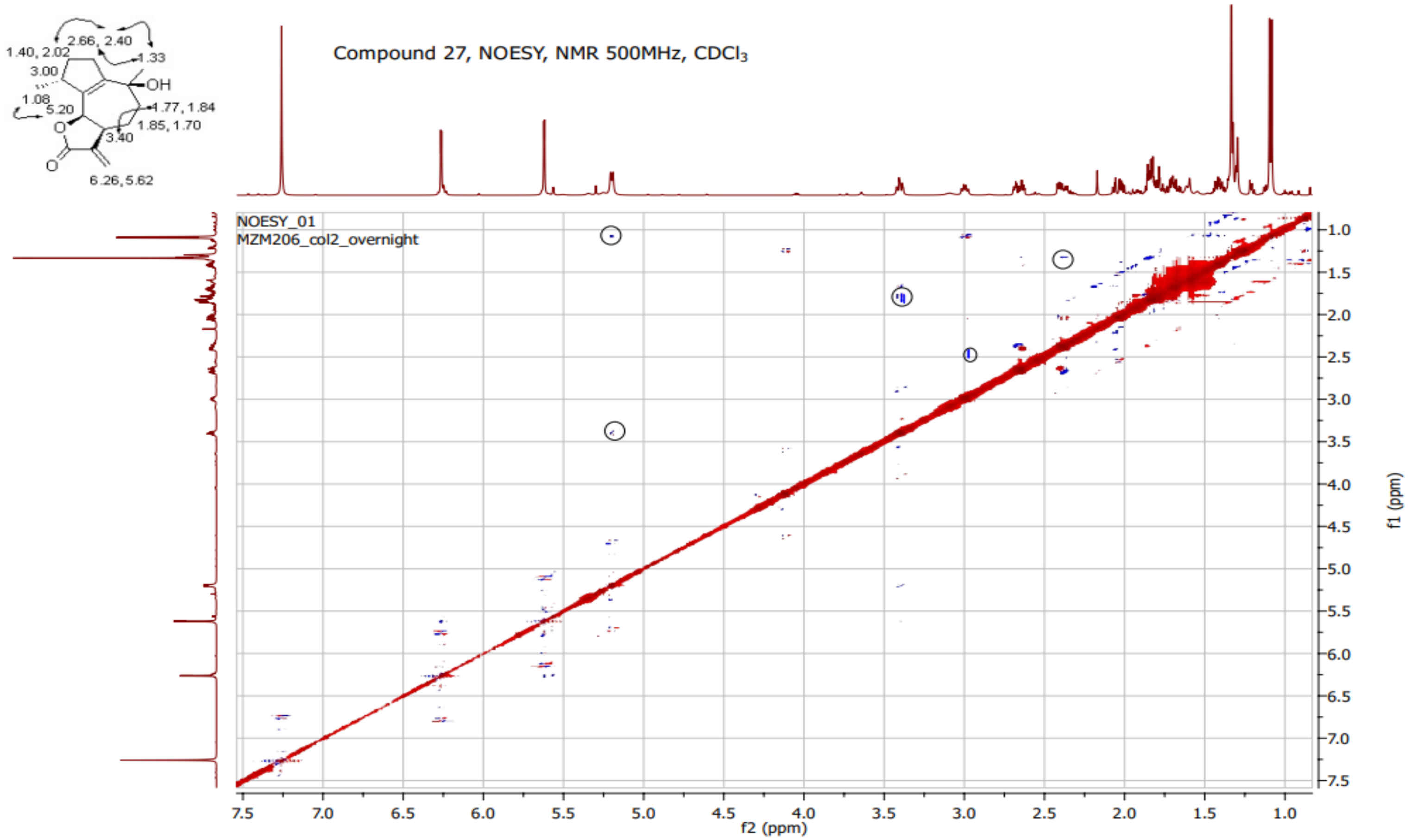
Compound 27, NOESY, NMR 500MHz, $CDCl_3$
NOESY_01
MZM206_col2_overnight
f2 (ppm)
f1 (ppm)

## 6. References


(1) Kourgiantaki, M.; Demertzidou, V. P.; Zografos, A. L. Short Scalable Route to *Apiaceae* Sesquiterpene Scaffolds: Total Synthesis of 4- *Epi* -Epiguaidiol A. *Org Lett* 2022, *24* (46), 8476–8480. https://doi.org/10.1021/acs.orglett.2c03215.

(2) Hegde, S. G.; Vogel, M. K.; Saddler, J.; Hrinyo, T.; Rockwell, N.; Haynes, R.; Oliver, M.; Wolinsky, J. The Reaction of Hypochlorous Acid with Olefins. A Convenient Synthesis of Allylic Chlorides. *Tetrahedron Lett* 1980, *21* (5), 441–444. https://doi.org/10.1016/S0040-4039(00)71427-4.

(3) Kornblum, N.; Jones, W. J.; Anderson, G. J. A NEW AND SELECTIVE METHOD OF OXIDATION. THE CONVERSION OF ALKYL HALIDES AND ALKYL TOSYLATES TO ALDEHYDES. *J Am Chem Soc* 1959, *81* (15), 4113–4114. https://doi.org/10.1021/ja01524a080.

(4) Bal, B. S.; Childers, W. E.; Pinnick, H. W. Oxidation of α,β-Un Saturated Aldehydes. *Tetrahedron* 1981, *37* (11), 2091–2096. https://doi.org/10.1016/S0040-4020(01)97963-3.

(5) Demertzidou, V. P.; Kourgiantaki, M.; Zografos, A. L. Expanding Natural Diversity: Tailored Enrichment of the 8,12-Sesquiterpenoid Lactone Chemical Space through Divergent Synthesis. *Org Lett* 2024, *26* (22), 4648–4653. https://doi.org/10.1021/acs.orglett.4c01374.

(6) Chen, D.; Evans, P. A. A Concise, Efficient and Scalable Total Synthesis of Thapsigargin and Nortrilobolide from ( *R* )-(−)-Carvone. *J Am Chem Soc* 2017, *139* (17), 6046–6049. https://doi.org/10.1021/jacs.7b01734.
